# Yoga vs. physical therapy vs. education for chronic low back pain in predominantly minority populations: study protocol for a randomized controlled trial

**DOI:** 10.1186/1745-6215-15-67

**Published:** 2014-02-26

**Authors:** Robert B Saper, Karen J Sherman, Anthony Delitto, Patricia M Herman, Joel Stevans, Ruth Paris, Julia E Keosaian, Christian J Cerrada, Chelsey M Lemaster, Carol Faulkner, Maya Breuer, Janice Weinberg

**Affiliations:** 1 Department of Family Medicine, Boston University School of Medicine and Boston Medical Center, 1 Boston Medical Center Place, Dowling 5 South, Boston, MA 02118, USA; 2 Group Health Research Institute and Department of Epidemiology, University of Washington, 1730 Minor Avenue, Suite 1600, Seattle, WA 98101, USA; 3 School of Health and Rehabilitation Sciences, University of Pittsburgh, 4028 Forbes Tower, Pittsburgh, PA 15260, USA; 4 RAND Health Unit, RAND Corporation, 1776 Main Street, Santa Monica, CA 90407, USA; 5 Boston University School of Social Work, 264 Bay State Road, Boston, MA 02215, USA; 6 Art & Soul Yoga Studio, 220 Pearl Street, Cambridge, MA 02139, USA; 7 Santosha School of Yoga, 14 Bartlett Avenue, Cranston, RI 02905, USA; 8 Department of Biostatistics, Boston University School of Public Health, 801 Massachusetts Avenue, Crosstown, 3rd floor, Boston, MA 02118, USA

**Keywords:** Complementary and Alternative Medicine, Cost effectiveness, Low back pain, Physical therapy, Randomized controlled trial, Yoga

## Abstract

**Background:**

Chronic low back pain causes substantial morbidity and cost to society while disproportionately impacting low-income and minority adults. Several randomized controlled trials show yoga is an effective treatment. However, the comparative effectiveness of yoga and physical therapy, a common mainstream treatment for chronic low back pain, is unknown.

**Methods/Design:**

This is a randomized controlled trial for 320 predominantly low-income minority adults with chronic low back pain, comparing yoga, physical therapy, and education. Inclusion criteria are adults 18–64 years old with non-specific low back pain lasting ≥12 weeks and a self-reported average pain intensity of ≥4 on a 0–10 scale. Recruitment takes place at Boston Medical Center, an urban academic safety-net hospital and seven federally qualified community health centers located in diverse neighborhoods. The 52-week study has an initial 12-week Treatment Phase where participants are randomized in a 2:2:1 ratio into i) a standardized weekly hatha yoga class supplemented by home practice; ii) a standardized evidence-based exercise therapy protocol adapted from the Treatment Based Classification method, individually delivered by a physical therapist and supplemented by home practice; and iii) education delivered through a self-care book. Co-primary outcome measures are 12-week pain intensity measured on an 11-point numerical rating scale and back-specific function measured using the modified Roland Morris Disability Questionnaire. In the subsequent 40-week Maintenance Phase, yoga participants are re-randomized in a 1:1 ratio to either structured maintenance yoga classes or home practice only. Physical therapy participants are similarly re-randomized to either five booster sessions or home practice only. Education participants continue to follow recommendations of educational materials. We will also assess cost effectiveness from the perspectives of the individual, insurers, and society using claims databases, electronic medical records, self-report cost data, and study records. Qualitative data from interviews will add subjective detail to complement quantitative data.

**Trial registration:**

This trial is registered in ClinicalTrials.gov, with the ID number: NCT01343927*.*

## Background

### Low back pain in low-income minority populations

Low back pain (LBP) is the most common cause of pain in the United States (US) [1,2], resulting in substantial morbidity [3], disability [4,5], and costs [6,7] to society. Chronic LBP (cLBP) lasting more than 12 weeks affects an estimated 5 to 10% of US adults [1,2,5]. The majority of cLBP patients are classified as having non-specific cLBP, i.e., there is no identifiable anatomic source for their pain [1]. Non-specific cLBP accounts for a majority of back-related health expenditures [8]. One-fourth of US adults experience LBP for at least one day over a three-month period [2]. LBP accounts for 34 million office visits annually by family physicians and primary care internists [3]. Annual direct costs for LBP care in the US are more than $50 billion [6] and indirect costs (e.g., productivity) are estimated to be even larger [9]. Back pain patients incur up to 75% more medical expenditures than patients without back pain [6,7]. Back injury is the leading and most expensive cause of workers’ compensation claims [4,5].

Although LBP prevalence in US whites, blacks, and Hispanics is similar [2], racial and ethnic disparities in access and treatment exist [3,6,10,11]. Medical expenditures for LBP in minorities are 30% lower than for whites [6]. Minorities with LBP receive less patient education [3], narcotic prescriptions [3,10], back surgery [11], specialty referrals [12], and intensive rehabilitation for occupational back injuries [13]. Reasons for disparities may include lack of adequate health insurance, lower income, and less education. Attitudes and beliefs of providers and patients may also play a role. For example, physicians may assess a person’s pain differentially based on race [11]. A history of racial discrimination experienced or perceived by a minority individual can also be associated with greater levels of back pain [14]. Few intervention studies for LBP have targeted minority populations. Although several studies have demonstrated racial and socioeconomic disparities in LBP treatment and outcomes [3,6,10,11,13], there is a large need for LBP intervention trials which specifically target minority underserved populations.

Although there is a range of conventional pharmacologic, non-pharmacologic, and surgical procedures used for non-specific cLBP, most patients report only modest or moderate relief, at best. Commonly, management for non-specific cLBP includes advice to remain physically active, education on back self-care, medication, and physical therapy (PT) [15]. Patient satisfaction with the effectiveness of conventional cLBP treatment is relatively low [16]. Thus, there is a substantial need for research to identify more helpful therapies.

### Yoga for low back pain

Yoga is increasingly popular in the US [17-19]. In 2007, 6.1% of people reported practicing yoga in the past year, an increase from 5.1% in 2002 [20]. Although yoga’s popularity has increased, its use among minorities and individuals with lower income or education is less common [17,18]. Being non-white, less educated, and having poor health status are all independent factors associated with less yoga use [21]. Data from the 2007 National Health Interview Survey show yoga use was 6.9% in whites vs. 3.2% in blacks; 14.6% in individuals with graduate degrees vs. 2.4% of those with a high school degree or equivalent; and 9.8% of individuals in the highest income quartile vs. 4.6% of individuals in the lowest quartile [21]. Minorities and people with low socioeconomic status are more likely to have undertreated back pain and are also less likely to use practices such as yoga. If complementary therapies, such as yoga, for back pain are to be rigorously studied, trials need to target all affected populations including low-income minorities.

Five large (n = 90–313) [22-26] and five smaller randomized controlled trials (RCTs) (n = 20–60) [27-31] support yoga’s effectiveness for reducing pain and improving function in adults with cLBP. Our pilot RCT of 30 adults recruited from two federally qualified community health centers in Boston demonstrated the feasibility of recruiting low-income minorities for a yoga LBP trial [28]. Our subsequent trial of 95 adults with cLBP recruited from Boston Medical Center (BMC) and five affiliated community health centers showed that both once-weekly and twice-weekly yoga classes for 12 weeks were similarly effective for reducing pain and improving back related function in a mostly low-income diverse population [26]. Reviews and meta-analyses [32-34] and practice guidelines from the American Pain Society and the American College of Physicians [35] support yoga as an evidence-based treatment for cLBP with at least moderate benefit. However, no studies to date have compared yoga’s effectiveness to PT, the most commonly non-pharmacologic reimbursable treatment physicians recommend [36].

### Physical therapy for low back pain

Physical therapists evaluate patients with musculoskeletal disorders and administer a range of interventions including stretching, strengthening, aerobic conditioning, and manipulation. Other modalities used by physical therapists include application of ice and heat, ultrasound, and transcutaneous electrical nerve stimulation (TENS). Although exercise therapy for cLBP can be conducted in many formats and settings, it most commonly occurs through a physician referral to a physical therapist [37,38]. A substantial proportion of patients with LBP are referred to PT [36,37], especially by primary care doctors and orthopedists [38]. Most PT visits are from physician referral because insurance companies typically reimburse the physical therapist only if prescribed by a physician [38]. Analyses of the National Ambulatory Medical Care Survey show 22% of patients with mechanical LBP seen by primary care physicians are referred to PT [36]. Data from the Medical Expenditure Panel Survey determined that the annual mean expenditures on PT per respondent with spine problems increased from $115 in 1997 to $129 in 2005, and an estimated $4.3 billion total was spent on PT for spine problems in 2005 [7]. Back problems comprise a significant portion of the conditions physical therapists commonly treat. A national survey of outpatient PT practices found that 26% of visits were for LBP [38]. Data also suggest disparities in access to PT. Individuals with higher education are more likely to receive PT and less likely if they have Medicaid [36]. PT is the most common non-pharmacologic referral for cLBP made by physicians. Therefore, physicians, patients, and insurers considering a new therapy will want to know how it compares in effectiveness to established treatments such as PT.

There are several evidence-based clinical guidelines for the treatment of cLBP which provide guidance for physical therapists. The American Pain Society/American College of Physicians issued a clinical practice guideline finding good evidence that exercise therapy has a moderate effect for cLBP [35]. Due to inconsistent or poor quality evidence, they were unable to recommend several passive therapies commonly used by physical therapists such as TENS or ultrasound [35]. The American Physical Therapy Association’s Low Back Pain Clinical Practice Guidelines as well as several European guidelines all found strong evidence for trunk coordination, strengthening, and endurance exercises [39-42]; systematic reviews and meta-analyses support these guidelines. In a Cochrane review of 43 cLBP trials, Hayden et al. found strong evidence that exercise therapies are as effective or more effective compared to other conservative treatments [43,44]. Using Bayesian multivariable random-effects meta-regression techniques, they concluded that the most effective exercise therapy strategy for improving cLBP was supervised, individually-tailored, high-dose stretching and muscle strengthening exercise programs with home practice [44].

### Long-term adherence to treatment

Many cLBP patients have longstanding pain. For example, 80% of patients in our previous studies of predominantly low-income minorities with cLBP had back pain lasting more than one year; 21% reported back pain for more than nine years [26,28]. However, few non-pharmacologic intervention studies for cLBP have included ongoing structured maintenance components beyond an initial 8–16-week intervention period. In 43 non-pharmacologic RCTs systematically reviewed by Chou and Huffman [35], only one exercise study contained a formal exercise maintenance program for participants [45]. Optimizing long-term outcomes for cLBP will likely require an ongoing chronic disease management approach. Therefore, cLBP studies need to evaluate not only long-term follow-up, but long-term models designed to maintain clinical effectiveness and support patient adherence.

### Cost effectiveness

Little is known regarding the cost effectiveness of many of the conventional and complementary therapies used for cLBP [46-50]. Only one study has examined the economic impact of yoga for cLBP; using data from a UK multi-centered RCT, Chuang et al. found that yoga yielded an incremental cost-effectiveness of 13,606 British pounds per Quality-Adjusted Life Year (QALY) [51]. Indirect costs for cLBP (predominantly change in work productivity in most populations [52]) are large and estimated to be greater than direct costs [9,53-57]. Given the usefulness of cost effectiveness data to health policy and other decision makers, building cost effectiveness analyses into comparative effectiveness trials for cLBP is critical [47,58].

### Specific aims

Evidence from multiple studies supports a moderate benefit in cLBP for yoga as well as exercise therapy individually delivered by a physical therapist [35,43,59]. Education, in the form of physician advice and handouts, are a common part of primary care provided to patients with cLBP [15]. However, no studies to date have done a head-to-head comparison of the effectiveness of yoga, PT, and education for cLBP. To ultimately reduce disparities in cLBP for minority populations, patients, providers, and health insurers need to know how evidence-based complementary therapies, such as yoga, compare in effectiveness to more well-established treatments such as PT and education. If yoga is superior to education and has similar effectiveness as PT, but costs less and has greater adherence, the potential therapeutic and economic implications would be substantial. Alternatively, if yoga is inferior, this information will help guide better treatment decisions and reduce unnecessary expenditures on inferior treatments.

The present study (“Back to Health”) is a 52-week randomized controlled trial of once-weekly yoga classes, individually-delivered PT, and education for cLBP in 320 individuals from predominantly low-income minority backgrounds recruited from BMC and affiliated community health centers. The trial starts with an initial 12-week Treatment Phase followed by a 40-week Maintenance Phase. Back to Health has the following three specific aims:

1. In the 12-week Treatment Phase, compare the effectiveness between a structured protocol of one yoga class per week, an individually-delivered structured PT protocol established around evidence-based clinical guidelines, and an educational book on self-care for cLBP.

2. In the 40-week Maintenance Phase, compare the effectiveness between patients participating in a structured yoga maintenance program, a structured PT maintenance program, or no structured maintenance program.

3. Determine the cost-effectiveness of yoga, PT, and education for adults with cLBP at 12 weeks, 6 months, and one year from three perspectives: society, third-party payer, and the participant.

## Methods

### Study Design

**Figure 1:**
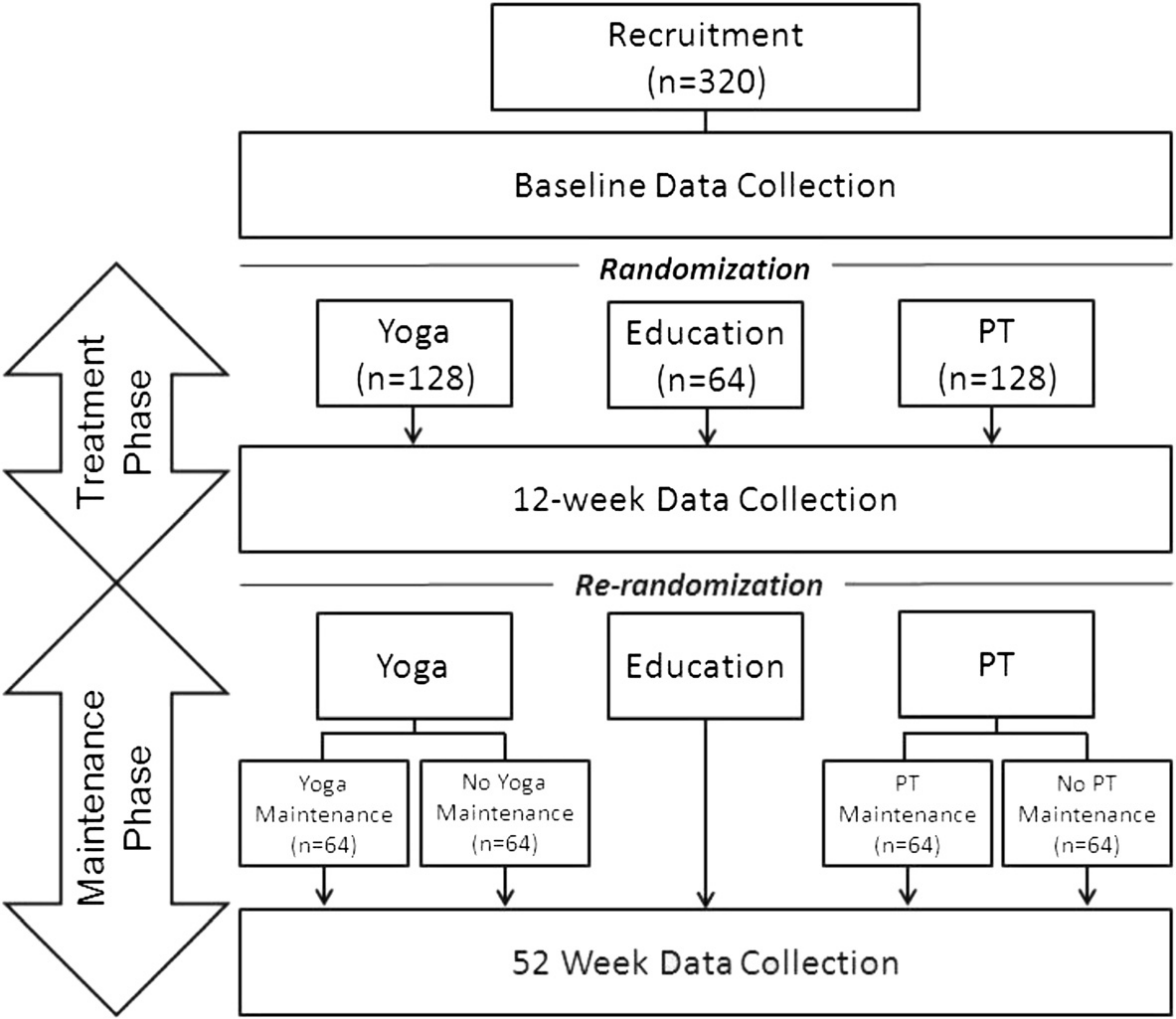
**Study flow diagram.** The study will recruit 320 participants with chronic low back pain from Boston Medical Center and surrounding affiliated federally qualified community health centers. Participants are randomized after baseline data collection to yoga classes, physical therapy (PT), or education through a self-care book in a 2:2:1 ratio. This year-long study is divided into a 12-week Treatment Phase and subsequent 40-week Maintenance Phase. Yoga and PT participants who have attended at least one intervention session during the Treatment Phase are re-randomized at 12 weeks into a structured maintenance intervention or no structured maintenance intervention. For the participants, we term the no structured maintenance intervention as “Home Practice” only. Education participants continue through the study without any re-randomization.

We use a single-blinded RCT study design. The trial is 52 weeks long and divided into two distinct parts: the initial 12-week Treatment Phase followed by the 40-week Maintenance Phase. For the initial 12-week Treatment Phase, participants are randomized in a 2:2:1 ratio into three treatment groups: yoga, PT, or education delivered through a self-care book [60] (Figure 1). The study co-primary endpoints are mean pain intensity over the previous week measured on an 11-point numerical rating scale (0 to 10, where 0 = no pain and 10 = worst possible pain) [61,62] and back-related function measured using the 23-point modified Roland Morris Disability Questionnaire (RMDQ; 0 to 23, where higher scores indicate worse back-related function) [63,64]. We hypothesize that yoga will be non-inferior to PT, and that both yoga and PT will be superior to education.

For the subsequent 40-week Maintenance Phase, there are five separate groups. At the end of the Treatment Phase, yoga participants are re-randomized in a 1:1 ratio to either a structured ongoing maintenance yoga program or no structured maintenance. Similarly, PT participants are re-randomized in a 1:1 ratio to either a structured ongoing maintenance PT program or no structured maintenance. Yoga and PT participants randomized to no structured maintenance are encouraged to continue with home practice. Education participants are not re-randomized and are encouraged to continue to review and follow the recommendations of their educational materials. For the Maintenance Phase, we hypothesize: i) maintenance yoga will be non-inferior to maintenance PT; ii) maintenance yoga will be superior to no yoga maintenance; iii) maintenance PT will be superior to no maintenance PT; and iv) maintenance yoga and maintenance PT will both be superior to education.

In addition, we collect data on medical utilization, out-of-pocket direct and indirect medical costs, implementation costs, and health-related quality of life to compare the cost-effectiveness of yoga, PT, and education from three perspectives: society, third-party payer, and the participant. We hypothesize that yoga will be more cost-effective than PT. Individual interviews using a semi-structured interview guide will be analyzed using qualitative methods. This study was approved by the Boston University Medical Campus (BUMC) Institutional Review Board.

### Study sites

Participants are recruited from the patient base of eight sites within the city of Boston that serve a diverse predominantly low-income population. Of these eight sites, one is BMC, a large academic medical center safety net hospital. The other sites are federally qualified community health centers in the Boston area (Codman Square Health Center, Dorchester House Multiservice Center, Upham’s Corner Health Center, Greater Roslindale Medical and Dental Center, Dimock Community Health Center, South Boston Community Health Center, and South End Community Health Center). Each health center has a physician who is a designated study site champion. Their role is to facilitate recruitment, assist with study logistics, and liaise with the principal investigator (PI) and study staff.

Yoga classes are offered at BMC and the different community health centers. The PT intervention is delivered by physical therapists at two sites of New England Physical Therapy Plus, Inc. (NEPT Plus), a private network of PT clinics throughout Boston, and from the Boston Medical Center Physical Therapy Department. For participants who are assigned to PT, they choose to receive the PT intervention from either NEPT Plus or BMC Physical Therapy depending upon convenience of location.

### Inclusion and exclusion criteria

Tables 1 and 2 list the inclusion and exclusion criteria as well as the rationale for each criterion. Inclusion criteria are as follows: 18 to 64 years old; current non-specific LBP persisting ≥12 weeks with average pain intensity ≥4 for the previous week on an 11-point numerical rating scale; and ability to speak and understand English. Non-specific LBP refers to the absence of clear symptoms and signs of specific anatomical causes (e.g., large herniated disk). Participants must also be willing to list comprehensive contact information for at least one (preferably two) friend, family member, or work colleague. Participants must be planning to stay in the area for at least one year. Exclusion criteria are as follows: specific causes of LBP including spinal canal stenosis, >Grade I spondylolisthesis, ankylosing spondylitis, moderate to severe scoliosis, malignancy, and fracture; having practiced yoga within the past 6 months; having received PT for back pain within the past 6 months; having read *The Back Pain Helpbook*[60] or *The Back Book*[65]; new cLBP treatment(s) started within the previous month or anticipated to begin in the next 3 months; previous back surgery; severe or progressive neurological deficits; sciatica pain equal to or greater than back pain; active or recent cervical radiculopathy; active or planned worker’s compensation, disability, or personal injury claims; perceived religious conflict; or any severe psychiatric or medical comorbidity that in the judgment of the PI would make participation unsafe or not possible.

**Table 1:** Inclusion criteria

**Inclusion criteria**	**Rationale**
18 to 64 years old	Chronic low back pain in older adults is more likely to have specific causes (e.g., spinal canal stenosis)
Current non-specific low back pain persisting ≥12 weeks	Condition studied is specifically chronic
Mean low back pain intensity for the previous week ≥4 on a 0 to 10 numerical rating scale (0 = no pain to 10 = worst possible pain)	Back pain severe enough to detect improvement and prevent against floor effects
English fluency sufficient to follow treatment instructions and answer survey questions	Fully informed consent and data collection
Willingness to list comprehensive contact information for at least one (preferably two) friend, family member, or work colleague who will always know how to contact the participant	Minimize loss to follow-up

**Table 2:** Exclusion criteria

**Exclusion criteria**	**Rationale**
Significant participation in yoga or physical therapy in the previous 6 months	Possible bias, confounding, or residual treatment effect
Has read *The Back Pain Helpbook* or *The Back Book* in the previous 6 months	
Has previously participated in our yoga or physical therapy studies	
New chronic low back pain (cLBP) treatments started within the previous month or anticipated to begin in the next 12 months	
Inability to understand English at a level necessary to understand treatment instructions and survey questions	Condition would make it difficult for fully informed consent and to follow intervention instructions
Known pregnancy	Pregnancy-related low back pain is different in etiology and time course than the target condition for the study, i.e., non-specific cLBP
Active or planned worker’s compensation, disability, or personal injury claims	Medico-legal concerns may bias participants’ incentive to improve or bias reporting of outcomes
Spinal canal stenosis	Back pain possibly due to, specific disease/condition(s)
Severe scoliosis	
Spondylolisthesis	
Ankylosing spondylitis	
Large herniated disk	
Sciatica pain equal to or greater than back pain	
Previous back surgery	
History of vertebral fracture	
Active or recent malignancy	
Active or recent constitutional symptoms	
Rheumatoid arthritis	Condition may overlap with symptoms of back pain and/or confound treatment effects
Severe fibromyalgia	
Other severe disabling chronic medical and/or psychiatric comorbidities deemed by the principal investigator on a case-by-case basis to prevent safe and/or adequate participation in the study (e.g., severe disabling heart failure or lung disease, active treatment for hepatitis B/C, psychosis)	Comorbid condition(s) that may pose inappropriate risk to safety or preclude compliance with interventions
Severe or progressive neurological deficits	
Active substance or alcohol abuse	
Plans to move out of the area in the next 12 months	Known barrier to full study participation
Perceived religious conflict with the yoga intervention	
Lack of consent	Research policy

### Recruitment

Participants are recruited predominantly through the sites using a multi-pronged strategy. This strategy has been successfully employed in past studies to recruit a predominantly low-income minority population [26,28].

1. Generate lists of patients aged 18 to 64 with a diagnosis of LBP (International Classification of Diseases, Ninth Revision codes 724.2 and 724.5) in the electronic medical records from BMC and affiliated community health centers. We then send a recruitment letter and study flyer to each patient.

2. Posting study flyers in clinic waiting rooms and exam rooms and in surrounding neighborhoods.

3. Emails, letters, electronic medical record alerts, and in-service presentations to BMC and community health center providers and staff about the study. Providers can then suggest to their patients who may be eligible for the trial to contact the study staff via telephone or email.

4. Disseminate information about the study to the BUMC community through electronic communications and signage.

5. Local community newspaper ads.

6. Staff tables with study information at health centers and events in the surrounding community such as farmer’s markets, neighborhood festivals, and health fairs.

The screening and enrollment process involves three parts: i) verbal consent and completion of a telephone eligibility screening; ii) in-person informational meeting with research staff; and iii) administration of informed consent and signing written consent form.

Eligibility screening for a potential participant takes place over the telephone by research staff using a questionnaire. The research staff member asks the potential participant for verbal consent before proceeding with screening questions. If there is any uncertainty on whether a participant is eligible, we obtain consent to review their medical records and/or contact their physician for more information. Based upon this initial screen, if an individual appears to be eligible for participation, he or she is asked to meet with research staff for an individual or group information session. This meeting takes place at BMC or one of the community health centers and takes approximately 45 minutes. The information session includes a short PowerPoint presentation about the study and the treatments, following which interested participants meet with research staff one-on-one. The research assistant reviews the study intervention and potential risks, benefits, and alternatives to participation. Additionally, the research assistant discusses expectations of study participants and the time commitment involved. It is made clear to the potential participant that he or she can withdraw from consideration at any time. The potential participant is asked frequently throughout the enrollment process if he or she has any questions. All questions are answered fully to the potential participant’s satisfaction. It is stressed that participation or withdrawal from the study will not impact their ability to receive their usual medical care in any way. For those interested in joining the study, we obtain informed consent verbally and written consent by signing the written consent form.

Prior to baseline survey administration and randomization, blinded study staff administer an eligibility verification checklist to assure that participants are still eligible to enroll in the study. This is to account for the potential time gap between consent and baseline survey administration (maximum 120 days).

### Randomization for 12-week Treatment Phase

Randomization occurs after administering the baseline survey. We use the randomization procedure built into our study management system (StudyTRAX™, Macon, GA, USA) to randomize each enrolled participant using a 2:2:1 ratio to yoga, PT, and education, respectively. Permuted variably-sized block randomization with block sizes of 5, 10, and 15 are used.

### 12-week Treatment Phase interventions

The study interventions start within one week of baseline data collection and randomization. All participants throughout the entire 52-week study can continue to receive routine medical care including doctor visits and pain medication.

#### Hatha yoga

The hatha yoga intervention is structured and reproducible. Originally, it was developed by an expert panel led by the PI in 2007 and used in a pilot study of 30 participants with cLBP [28]. It was further refined in 2011 in a study of 95 participants comparing once-weekly and twice-weekly yoga classes for 12 weeks [26]. Both class frequencies were similarly effective for reducing pain and improving back-related function. Due to the convenience and lower cost of once-weekly compared to twice-weekly classes, a once-weekly 75-minute yoga class was chosen for the current study.

Each class begins with *svasana* (a relaxation exercise), yoga breathing exercises (*pranayama*), and a brief discussion of yoga philosophy (Table 3). The class proceeds with yoga postures (*asanas*). Yoga breathing is emphasized throughout. The class ends with *svasana*. The 12 weeks are divided into four 3-week segments (Table 4). Each segment is given a theme (e.g., “Listening to the Wisdom of the Body”). Participants are frequently advised to go slowly and carefully. The degree of difficulty of postures learned increases with each segment. For each segment, the participants gradually learn a sequence of 15–19 poses. The protocol provides variations and uses various aids (e.g., chair, yoga strap, yoga block) to accommodate a range of physical abilities. A variety of world music is used during the classes. Participants are strongly encouraged to do yoga home-practice for 30 minutes daily on days which they do not attend yoga class. To facilitate home practice, participants receive a DVD of the protocol, a guidebook describing and depicting the protocol (Additional file 1), and a yoga mat, strap, and block.

**Table 3:** Standard yoga class format

**Curriculum elements**	**Time (min)**
Check in with participants	3
Lesson introduction and yoga philosophy	3
Relaxation exercise	3
Breathing exercise	4
Yoga postures	55
Closing relaxation	5
Closing	2
Total time	75 minutes

Teachers can exercise flexibility by incorporating the introduction and yogic philosophy into the check in, beginning relaxation, and/or breathing exercises. Some elements can be combined such as the relaxation and breathing exercises. The poems and readings at the end of the yoga instructor manual can be used throughout the class to support the particular themes and yogic philosophy for that week.

**Table 4:** Twelve-week standardized hatha yoga protocol

**Yoga posture (*Asana)***	**Classes incorporating posture by segment**				**Total classes incorporating posture**
	**Segment 1 weeks 1–3**	**Segment 2 weeks 4–6**	**Segment 3 weeks 7–9**	**Segment 4 weeks 10–12**	
	**Opening to something greater**	**Listening to the wisdom of the body**	**Engaging your power**	**Bringing it home**	
*Svasana* relaxation and breathing exercises	✓	✓	✓	✓	12
Knee to chest*	✓	✓	✓	✓	12
Knee together twist*	✓	✓	✓	✓	12
Pelvic tilt*	✓	✓	✓		9
Cat and cow pose (and modifications)*	✓	✓	✓		9
Chair pose (and modifications)*	✓	✓	✓		9
Shoulder opener*	✓	✓	✓	✓	12
Crescent moon (and modifications)*	✓	✓	✓		9
Mountain pose (and modifications)*	✓	✓	✓	✓	12
Chair twists, standing and seated	✓	✓	✓	✓	12
Child’s pose*	✓		✓	✓	9
Cobra (and modifications)*	✓	✓	✓	✓	12
Bridge pose* (with and without support)	✓	✓	✓	✓	12
Reclining cobbler*	✓	✓	✓		9
Downward facing dog (and at wall)*	✓	✓	✓	✓	12
Triangle pose (with and without the wall)		✓	✓	✓	9
Locust pose*	✓	✓			6
Sphinx*		✓	✓	✓	9
Standing forward bend at wall*		✓	✓	✓	9
Warrior pose*			✓	✓	6
Extended leg pose*		✓	✓	✓	9
Sun salutations				✓	3
Baby dancer pose*				✓	3
Spinal rolls				✓	3
*Svasana* integrative relaxation	✓	✓	✓	✓	12

The hatha yoga protocol developed for chronic low back pain patients consists of 12 weekly 75-min yoga classes divided into four 3-week segments. Each segment has a theme. The exercises for each segment are indicated in the table. Each class begins and ends with a relaxation exercise. The protocol provides for modifications of poses to accommodate different abilities.

*Exercises included in the DVD provided to participants for home practice.

Classes usually have 1–3 yoga teachers and no more than 4–5 participants per yoga teacher. This ensures adequate individual attention from the teacher, maximizes safety and effectiveness, and allows flexibility in yoga teacher scheduling based on the study’s needs. In the event that only one or two participants appear for a given class, the class still occurs and is not cancelled. In the event of unusual or extenuating circumstances (e.g., a participant misses multiple classes due to an emergency), we may attempt to set up a special make-up individual class so the participant can “catch up” and successfully rejoin his or her group class as soon as possible. However, these are rare; one-on-one instruction in the yoga group is not planned or routine.

All yoga teachers undergo a 12-hour training on the protocol delivered by a senior yoga instructor. All teachers receive and follow a yoga instructor manual (Additional file 2). A senior yoga instructor or the PI observe a sample of yoga classes and assure the protocol is appropriately being implemented using a checklist. Audits occur on 10% of yoga classes in each cohort.

#### Physical therapy

**Figure 2:**
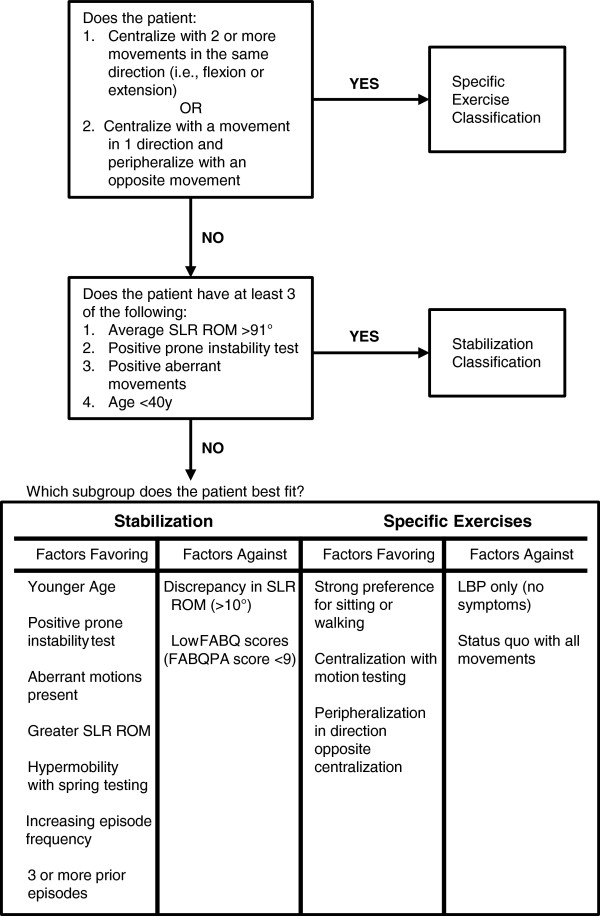
**Physical therapy intervention: the treatment-based classification algorithm.** Information from the history and examination is used to place chronic low back pain participants into one of two subgroups based on the pattern of signs and symptoms. Treatment is then based upon the participant’s subgroup classification. Participants’ classification is reassessed at each visit with appropriate adjustments to recommended exercises made. Participants are also classified according to the Fear Avoidance Belief Questionnaire (FABQ). PT participants who score ≥29 on the FABQ-W subscale receive *The Back Book* which provides brief cognitive behavioral education aimed at lowering fear avoidance. Physical therapists reinforce these points during treatment sessions and the participant’s exercise prescription is graded.

We used evidence-based evaluation and treatment methods for the PT intervention [39,66-68]. Evidence indicates classifying patients based on clinical characteristics and tailoring the management strategy will improve the effectiveness of the PT intervention. Information from the history and examination is used to place cLBP patients into one of two subgroups based on the pattern of signs and symptoms. Treatment is then based upon the participant’s subgroup classification (Figure 2). Physical therapists receive approximately 12 hours of training prior to participating in the study. Training includes an online web-based modular curriculum, in-person training led by A.D., J.S., and other senior physical therapists experienced in the protocol, review of a PT training manual (Additional file 3), and practice on non-study patient volunteers. Each PT participant has an initial intake evaluation by a physical therapist and is classified into one of two mutually exclusive subgroups: Specific Exercise or Stabilization. Within the Specific Exercise group, there are “flexion” and “extension” subgroups. Specific Exercise flexion participants receive lumbar flexion exercises. Specific Exercise extension participants receive lumbar extension exercises. Stabilization participants receive exercises to strengthen core trunk muscles (Table 5) [69]. Participants’ classification is reassessed at each visit with appropriate adjustments to recommended exercises made. Since the evidence suggests that manipulation be used primarily for acute LBP episodes, manipulation is not part of our treatment protocol [39]. In addition, passive modalities such as heat, ice, ultrasound, and TENS are not employed. Weekly completion of the Modified Oswestry Disability Index (MODI; range of possible scores 0 to 50, with higher scores indicating greater disability) is used internally by PT staff to monitor participant progress.

**Table 5:** Physical therapy stabilization exercise protocol

**Primary muscle group**	**Exercises**	**Criteria for progression**
*Transversus abdominus*	Abdominal bracing	30 repetitions with 8 s hold
	Bracing with heel slides	20 repetitions per leg with 4 s hold
	Bracing with leg lifts	20 repetitions per leg with 4 s hold
	Bracing with bridging	30 repetitions with 8 s hold, then progress to 1 leg
	Bracing with standing	30 repetitions with 8 s hold
	Bracing with standing row exercise	20 repetitions with 6 s hold
	Bracing with walking	10 minutes with cycles of 8 s hold and 10s rest
*Erector spinae-multifidus*	Quadraped arm lifts with bracing	30 repetitions with 8 s hold on each side
	Quadraped leg lifts with bracing	30 repetitions with 8 s hold on each side
	Quadraped alternative arm & leg lifts w/ bracing	30 repetitions with 8 s hold on each side
*Quadratus lumborum*	Side support with knees flexed	30 repetitions with 8 s hold on each side
	Side support with knees extended	30 repetitions with 8 s hold on each side
*Oblique abdominals*	Side support with knees flexed	30 repetitions with 8 s hold on each side
	Side support with knees extended	30 repetitions with 8 s hold on each side

Each participant randomized to the PT arm is also classified according to the Fear Avoidance Belief Questionnaire (FABQ). The FABQ measures the degree to which someone avoids physical activity due to fear of pain or injury [70]. High FABQ scores are associated with poor prognosis [71]. This can be mitigated through cognitive behavioral education delivered by the physical therapist and through a booklet (*The Back Book*) [65,72]. PT participants who score ≥29 on the FABQ Work subscale [73] receive *The Back Book*[65], which provides brief cognitive behavioral education aimed at lowering fear avoidance. The main points are reinforced by the physical therapists during treatment sessions. In addition, the participant’s exercise prescription is graded (i.e., it is slowly increased in frequency and intensity when the participant meets pre-specified targets) [74].

**Figure 3:**
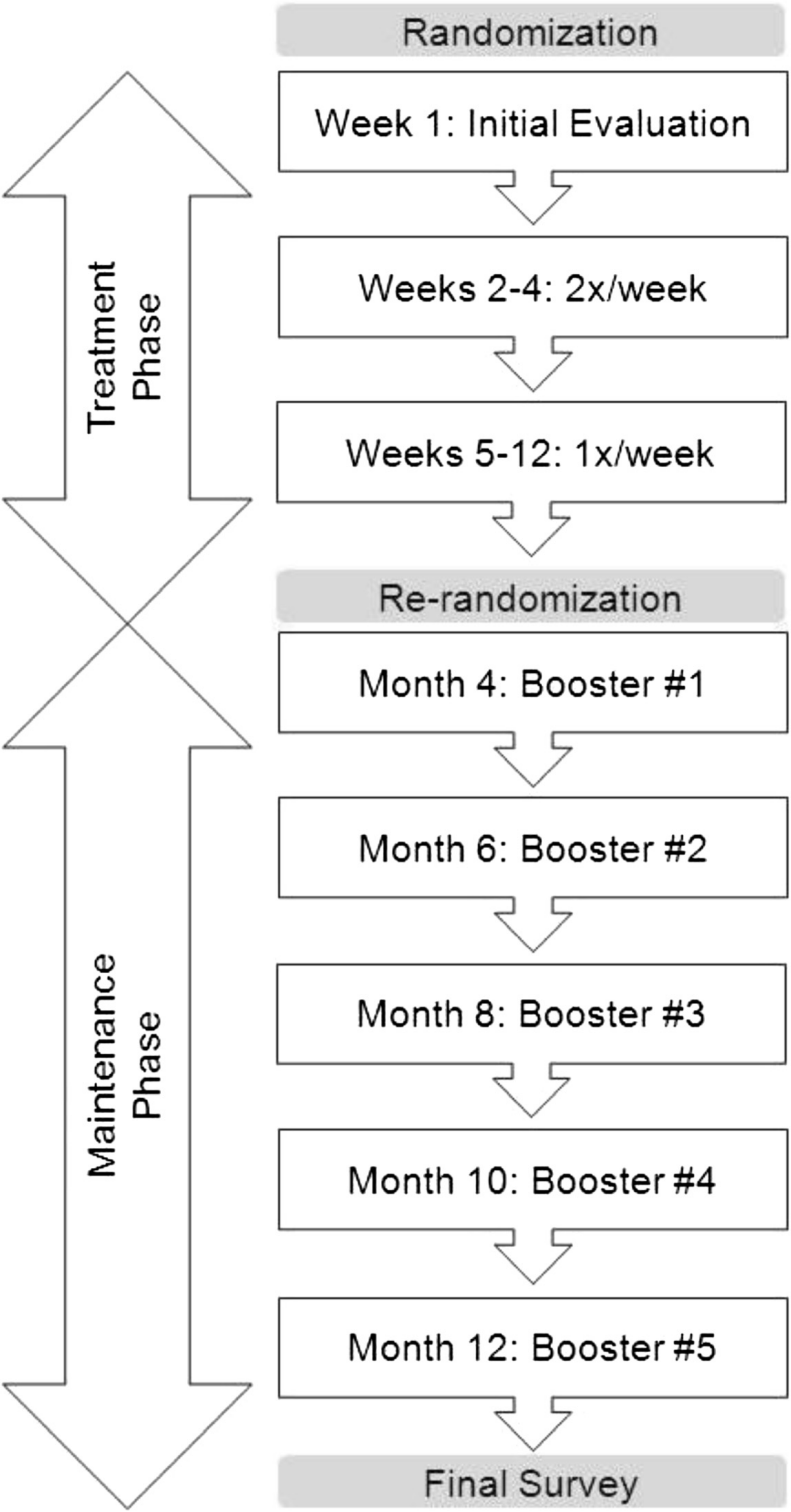
**Physical therapy intervention timeline.** Participants randomized to the physical therapy arm are offered a high dose, individually-tailored exercise program by physical therapists. A total of 15 individual 60-minute sessions over the course of the initial 12-week Treatment Phase is offered. Each session is divided into approximately 30 minutes of working directly with a physical therapist followed by up to 30 minutes of a supervised aerobic exercise routine. The first visit consists of an initial comprehensive evaluation for classification. Participants re-randomized at 12 weeks into a structured physical therapy maintenance program are offered five booster sessions scheduled at months 4, 6, 8, 10, and 12. The structure and content of booster sessions is similar to the Treatment Phase where therapists classify patients according to the Treatment Based Classification algorithm, review home practice, assess progress with the Modified Oswestry, review and perform recommended exercises, and provide encouragement and guidance for further home practice. PT participants randomized into the no maintenance (a.k.a. home practice) group do not receive booster sessions; however, they are encouraged to continue with their home practice exercises.

PT participants receive a high dose, individually-tailored, physical therapist-supervised exercise program with home practice. PT participants receive a total of 15 sessions of 60 minutes each during the 12-week Treatment Phase of the study according to a schedule shown in Figure 3. Sessions last 60 minutes and are divided into approximately 30 minutes of working directly with an individual physical therapist followed by up to 30 minutes of a supervised aerobic exercise routine. Recommendations for home exercise are 30 minutes on days when there is no PT session. Participants receive written materials and supplies (strap, mat) for home practice.

In the PT literature, 15 sessions in 12 weeks is considered a high dose of PT for LBP [44]. It allows participants to thoroughly learn the principles of the Stabilization exercise regimen and the aerobic routine, and carry over the exercises to a home program. Furthermore, 15 sessions is a high dose when compared to average numbers of PT visits for back pain in most practice settings (<10) [75].

To assess fidelity to the study protocol, PT treatment flow sheets are maintained by the physical therapists for each participant (Additional file 3). These are regularly reviewed by A.D. and J.S.; specific feedback to therapists are then provided during regularly scheduled conference calls and/or via email.

#### Education

Individuals randomized to the education group receive *The Back Pain Helpbook*[60], a 224-page educational book that encourages strategies for self-care including an exercise program, lifestyle modification, and tips for managing flare-ups (Table 6). In addition, they receive an assignment sheet outlining specific chapters to read over the course of the 12-week Treatment Phase. Participants also receive at 3, 6, 9, and 12 weeks 1 to 2 page newsletters written at a 6^th^ grade level that highlight the main points from the assigned chapters (Additional file 4). This book has been used successfully in previous cLBP studies for educational purposes [22,76].

**Table 6:** ***The Back Pain Helpbook*** * table of contents

Chapter 1	A self-assessment	3
Chapter 2	Back pain and you	15
Chapter 3	Reversing the downward spiral of back pain	39
Chapter 4	Effectively managing your back pain	47
Chapter 5	Managing flare-ups and emergencies	53
Chapter 6	Working with doctors and other health professionals	59
Chapter 7	Medicines for controlling back pain	65
Chapter 8	Physical methods of pain control	75
Chapter 9	Pain control through mind-body techniques	93
Chapter 10	Handling the effects of pain on thoughts and emotions	97
Chapter 11	Recognizing depressive illness when you have back pain	107
Chapter 12	A balanced approach to physical activity	113
Chapter 13	The comfort zone: key to good posture and body mechanics	119
Chapter 14	Stretch to prevent pain and stiffness	141
Chapter 15	Exercises for building strength and endurance	153
Chapter 16	Feeling better through aerobic activities	163
Chapter 17	Staying active in an inactive world	169
Chapter 18	Solutions for sleep problems	177
Chapter 19	Strengthening your relationships	185
Chapter 20	Intimacy and sex	193
Chapter 21	Back pain and your job	199
Chapter 22	Final thoughts on feeling and doing better	209
Appendix	The American Chronic Pain association’s ten steps for dealing with pain	215
Index		219

*The Back Pain Helpbook, [60].

### Randomization for 40-week Maintenance Phase

At the completion of the 12-week Treatment Phase, all participants who were initially randomized to the yoga arm and have attended at least one yoga class are re-randomized in a 1:1 ratio to either a structured ongoing maintenance yoga program for 40 weeks or no structured maintenance. Similarly, all participants who were initially randomized to the PT arm and have attended at least one PT session during the 12-week Treatment Phase are re-randomized in a 1:1 ratio to either a structured ongoing maintenance PT program for 40 weeks or no structured maintenance. Yoga and PT participants that did not go to any sessions during the 12-week Treatment Phase do not continue into the Maintenance Phase. Education participants continue into the Maintenance Phase without any re-randomization. They are encouraged to continue to review and follow the recommendations of their educational materials. Re-randomization of yoga and PT participants into their new treatment arms for the Maintenance Phase is done by the study biostatistician (J.W.) who has no contact with participants, knowledge of any participant identifying information, or other involvement in enrollment or randomization processes. Each participant eligible for the maintenance phase was assigned a random number using the random uniform function in SAS. For yoga and PT separately, the lower half of random numbers were assigned to maintenance and the larger half of numbers to no maintenance. Participants are notified of their re-randomization group after their 12-week survey by unblinded study staff. A password protected spreadsheet on the study’s network drive is used to document when and how participants are notified of their re-randomization assignment.

### 40-week Maintenance Phase interventions

#### Yoga maintenance

Yoga participants randomized into the structured yoga maintenance are encouraged to attend drop-in 75-minute yoga classes once per week for 40 weeks. These classes are also offered at the different study sites and are separate from Treatment Phase classes. Maintenance Phase classes have a higher participant-to-instructor ratio, usually no greater than 10:1. The structure of the drop-in classes is similar to the structure of the Treatment Phase classes. The relaxation, breathing, and posture exercises for drop-in classes are drawn from the Treatment Phase protocol (Table 4) according to instructor discretion and participant preference. In addition, they are encouraged to continue with their yoga home practice. Yoga participants randomized into no structured maintenance group are not invited to attend the drop-in yoga classes. Instead, they are advised to continue with their home practice and given a list of appropriate community non-study yoga classes which they are encouraged to attend. Daily practices of yoga and other exercises are recorded using home practice logs in both maintenance and no maintenance groups.

#### Physical therapy maintenance

PT participants randomized into the structured PT maintenance group receive five “booster sessions” scheduled at months 4, 6, 8, 10, and 12 (Figure 3). The structure and content of booster sessions is similar to sessions in the Treatment Phase. Therapists classify patients according to treatment-based classification, review home practice, assess progress with the MODI, review and perform recommended exercises, and provide encouragement and guidance for further home practice. PT participants randomized into the home practice group do not receive booster sessions; however, they are encouraged to continue with their home practice exercises. Daily practices of PT and other exercises are recorded using home practice logs in both maintenance and no maintenance groups.

The rationale for choosing five PT maintenance or booster sessions is based on reasonable and customary insurance coverage for PT in the Boston area. A typical annual maximum PT insurance benefit is 20 sessions [77]. Thus, participants randomized to PT and the PT structured maintenance program receive 20 sessions over the course of the one year study (i.e., 15 during the Treatment Phase and an additional five sessions in the Maintenance Phase).

### Data collection

There are six data collection points: baseline, 6, 12, 26, 40, and 52 weeks. Throughout the entire study, participants in all treatment arms are asked to complete their surveys in-person at pre-set times at their nearby community health center or BMC. The paper survey administration is proctored only by study staff blinded to participants’ treatment arm. If participants are unable to complete the surveys in-person, they may complete any post-baseline survey over the phone with a blinded research assistant. All paper surveys are double entered into StudyTRAX™ by blinded research assistants. Any inconsistencies between the two data entries are identified and reconciled.

#### Outcome measures

Table 7 shows the data collection schedule. The co-primary outcome measures are: i) average pain intensity in previous week on an 11-point numerical rating scale where 0 is no pain and 10 is the worst pain possible, and ii) back-related function using the 23-point modified RMDQ. Secondary outcomes include pain medication use in the previous week, health-related quality of life measured using the Short Form-36 Questionnaire (SF-36) [78], employment status [79], overall improvement (7-point Likert scale, 0 = extremely worsened to 6 = extremely improved), and patient satisfaction with treatment (5-point Likert scale, 1 = very satisfied to 5 = very dissatisfied) [80]. Exploratory outcomes include: i) FABQ [70]; ii) depression symptoms measured using the Patient Health Questionnaire 8 [81]; iii) anxiety symptoms measured using the Generalized Anxiety Disorder 7 [82]; iv) Pittsburgh Sleep Quality Index [83]; v) Pain Self-Efficacy Questionnaire [84]; vi) Perceived Stress Scale [85]; and vii) Coping Strategy Questionnaire [86].

**Table 7:** Content of baseline and follow-up assessments

**Measures**	**Baseline**	**6 weeks**	**12 weeks**	**26 weeks**	**40 weeks**	**52 weeks**
**Baseline information**						
Socio-demographics	x					
Expectations and preferences	x					
Comorbidities	x					
**Primary outcomes**						
Low back pain score	x	x	x	x	x	x
Roland Morris disability (RMDQ)	x	x	x	x	x	x
**Secondary outcomes**						
Pain medication use	x	x	x	x	x	x
Work productivity	x	x	x	x	x	x
Health-related quality of life (SF-36)	x	x	x	x	x	x
Global improvement		x	x	x	x	x
Satisfaction with treatment	x	x	x	x	x	x
**Treatment-related information**						
Cost diary		x	x	x	x	x
Adverse events		x	x	x	x	x
Other LBP treatments	x	x	x	x	x	x
**Exploratory outcomes and potential covariates**						
Fear Avoidance Beliefs (FABQ)	x	x	x	x	x	x
Exercise	x	x	x	x	x	x
Alcohol, drug, smoking	x		x			x
Height & weight	x		x			x
Pain Self-Efficacy (PSEQ)	x		x			x
Pittsburgh Sleep Quality (PSQI)	x		x			x
Depression (PHQ-8)	x		x			x
Anxiety (GAD-7)	x		x			x
Coping Strategies (CSQ)	x		x			x
Perceived Stress (PSS)	x		x			x

Socio-demographics collected at baseline include age, sex, marital status, race/ethnicity, religion, country of origin, years in US, primary language, highest level of education completed, and household annual income. Information on potential covariates is collected at baseline: i) LBP history (duration, frequency, previous cLBP treatments including PT and complementary and alternative medicine treatments); ii) expectation for each intervention’s ability to help their LBP, measured on an 11-point scale at baseline where 0 = not at all helpful and 10 = extremely helpful [87]; iii) participants’ preference for randomization to PT, yoga, or education; iv) tobacco, alcohol, and substance use; v) height and weight; and vi) comorbidities. Information on participant use of concomitant interventions for LBP (type, frequency, and duration) is collected throughout the study period.

#### Survey administration procedures

As with the baseline data collection, 6- and 12-week questionnaires are administered in-person at pre-set times. Outcome data (primary, secondary, and other measures as listed in Table 7) are collected at 6 and 12 weeks. In addition, participants provide cost data and rate their global improvement since the study began using a 7-point Likert scale.

Outcome data are also collected during the Maintenance Phase at 26, 40, and 52 weeks. As with the previous data collection periods, the questionnaires are administered by blinded study staff at scheduled data collection times (or by phone if in-person is not possible). Global improvement and cost data are also collected at these time points.

#### Cost effectiveness

We use a multi-method approach to collect cost data at 6, 12, 26, 40, and 52 weeks. Since our interventions may influence other common comorbidities of cLBP (e.g., depression [88] and obesity [89]), we measure total medical utilization, not only back-related utilization. Direct medical costs are measured, which consist of the cost of i) implementing the interventions themselves and ii) ongoing medical utilization during and after the intervention. Intervention implementation costs (e.g., non-study-specific staff hours, materials, facility use) are captured from study records and valued at their actual costs. Ongoing medical utilization including visits, hospitalizations, tests, radiology, and medications are taken directly from claims data, the BUMC Clinical Data Warehouse integrated databases, and electronic medical record systems. Direct medical costs are valued at their actual costs to the community health centers and BMC. Any non-insurance reimbursed medical utilization (e.g., acupuncturists, chiropractors, out-of-pocket back-related expenses) are obtained from a cost questionnaire adapted from previous studies [90] and are valued at the reported actual price paid by participants. The validity of the cost questionnaire is supported by a take-home cost diary [91]. The diary acts as a prompt for participants between data collection points to note visits to health practitioners, other health-related expenses, and non-medical direct costs (e.g., travel to yoga classes, childcare). This combination approach takes advantage of the fact that a diary can better capture a broad range of day-to-day costs, but a questionnaire can capture costs in a more consistent format with lower patient burden [92-94]. Direct non-medical costs for this study mainly consist of participants’ actual travel costs to the yoga and PT interventions, any childcare costs during those sessions, and the cost of any sessions attended after the Treatment Phase for patients not randomized to receive structured maintenance yoga or PT. These non-medical costs are valued at the reported actual price paid by participants. Unpaid childcare is valued at an average wage rate [95]. Indirect costs (i.e., lost productivity) for employed participants are calculated as the number of lost productive hours multiplied by a national average cost of employment for each participant’s general job category [96]. Lost productivity costs for those not in the workforce is assumed to be captured by their report of overall quality of life [97]. Lost productivity costs for those looking for work will be considered in sensitivity analyses. QALYs will be calculated based on the Short Form-based 6-Dimensional Measure of Health-Related Quality of Life, which is estimated from the results of the SF-36 using a previously developed algorithm [98,99].

#### Adverse events

The same strategy for collecting adverse event data is implemented across all study arms. Participants are instructed in their study introduction packets to contact the study staff immediately if they believe they have experienced an adverse event that may be a result of their involvement in the study. All participants have 24-hour emergency contact information for the PI and a member of the research staff. Furthermore, all data collections (i.e., 6, 12, 26, 40, and 52 weeks) include questions on whether the participant believes he or she incurred any possible intervention-related adverse events. Unblinded study staff and the PI follow-up on all these reports, as necessary. For this reason the PI may need to be unblinded to the treatment assignment for select participants with adverse events.

#### Policies and procedures

Detailed policies and procedures used by study staff to implement and operationalize recruitment, screening, enrollment, data collection, and adverse event reporting can be found in Additional file 5. These include multiple activities done by research staff to enhance treatment adherence including flexible yoga class schedules, weekly reminder calls, and education check-in calls. The BMC research team meets weekly and reviews the previous week and cumulative data on recruitment, enrollment, retention, and data collection. This approach allows the team to identify potential problems and rapidly initiate possible solutions. This quality improvement process allows for continuous monitoring of participants’ engagement with the study and discussion of any circumstances that deserve added attention.

#### Qualitative interviews

Participants are invited for an interview after the completion of the Treatment Phase based on their individual schedule and willingness to participate. The purpose of collecting qualitative data is to gain subjective insight and detail from participants to complement quantitative data. All participants sign a written consent form prior to the interview. Interviews are approximately 30 to 60 minutes long. Interviewers are not well known to the participants. A semi-structured interview guide (Additional file 6) is used to elicit responses regarding the following: i) motivations and expectations regarding the decision to join the study and participation in the interventions; ii) barriers and facilitators to treatment adherence and home practice; and iii) positive and negative experiences, perceptions, and effects of their participation in the study (e.g., changes in back pain, mood, stress). Interviews are audio-recorded and transcribed verbatim (excluding name and other identifiable information).

### Data analysis

Intention-to-treat (ITT) and per-protocol analyses will be performed for all effectiveness analyses with the ITT analysis considered as the primary method. The ITT analysis will include all patients that participated in the intervention regardless of adherence. Every effort will be made to minimize missing data and participant drop-out or loss to follow-up during the study period. We have accounted for possible drop-out or loss to follow-up by increasing the sample size by 20% in all groups. The “last value carried forward” approach will be used to fill in missing outcome variables; this approach reflects an analysis of the last known value of the outcomes and is thought to be conservative as far as the impact on the treatment effect. If the amount of missing data for outcomes or potential confounders is substantial (i.e., >10%), we will perform an analysis based on multiple imputation for comparison. We will also examine the pattern of missing data in order to discern whether the missing data mechanism could be non-ignorable. A per-protocol analysis will include only those participants who are considered to be adherent to the protocol as defined below.

#### Preliminary analysis

The success of randomization to study group will first be examined. The groups will be compared on baseline demographics and clinical characteristics using analysis of variance (ANOVA) or χ^2^ tests as appropriate. Variables that differ, using α = 0.1 level of significance, across the study groups are potential confounders and will be adjusted for in all subsequent analyses.

#### Analyses of Treatment Phase data

The primary hypothesis is that yoga is non-inferior to PT for our co-primary endpoints: reduction in back pain intensity and improvement in back-related function. First, a one-sided α = 0.05 level two-sample *t*-test will be used to determine if yoga is non-inferior to PT for the co-primary outcomes of pain and function. The mean differences between groups with corresponding standard errors, confidence intervals, and *P* values will be reported. While pain and RMDQ outcomes may have a tendency to be skewed, we are examining the change from baseline to week 12 in these scales as primary outcomes which are expected to have more normally distributed values. However, underlying assumptions of the *t*-test and regression models, including normality, will be examined. If important violations are found, then alternative methods will be explored including non-parametric methods. If any imbalance between groups is found in the preliminary analysis of the success of randomization, these variables will be used in regression models to adjust results, with change from baseline to week 12 with pain or function as the outcome, and treatment group and other potential confounders as predictors.

The following variables will be considered as possible effect modifiers for the relationship between treatment group and the outcomes of pain and function: study site, expectation of helpfulness, preference for treatment assignment, and depression. A multiplicative interaction term between treatment and the possible effect modifier of interest will be included in a regression model. If a significant interaction occurs, we will examine whether it is quantitative (e.g., two subgroups differ in the magnitude of effect, but the effect for both are in the same direction) or qualitative (i.e., two subgroups differ in the direction or interpretation of effect) in nature. We will also examine the secondary outcome of pain medication use during the previous week assessed at 12 weeks. Logistic regression with indicators for treatment and adjustment for possible confounders, including baseline medication use, will be used to compare medication usage rates between the yoga and PT groups. Medication subtypes including non-steroidal anti-inflammatory drugs, acetaminophen, opiates, muscle relaxants, and others will also be compared. Odds ratios with corresponding confidence intervals and *P* values will be reported.

We also hypothesize that both the yoga and PT groups will show superior back-pain co-primary outcomes (pain reduction and back-related function) compared to the education group at 12 weeks. A two-sided α = 0.05 level two-sample *t*-test will be used to determine if yoga is superior to education for the co-primary outcomes of pain and function. Similarly, a two-sided α = 0.05 level two-sample *t*-test will be used to determine if PT is superior to education for the co-primary outcomes of pain and function. The mean differences between groups with corresponding standard errors, confidence intervals, and *P* values will be reported. If any imbalance between groups is found in the preliminary analysis of the success of randomization, these analyses will be adjusted using regression models with change from baseline to week 12 with pain or function as the outcome and treatment group or other potential confounders as predictors. For comparing pain medication use between the yoga, PT, and education intervention arms, we will use logistic regression as described above.

We will compare the following secondary outcomes using either linear or logistic regression, as appropriate, with treatment and potential confounders as predictors: overall improvement, patient satisfaction, and health-related quality of life.

#### Analysis of Maintenance Phase data

For the Maintenance Phase, we hypothesize that i) maintenance yoga will be non-inferior to maintenance PT; ii) maintenance yoga will be superior to no yoga maintenance; iii) maintenance PT will be superior to no maintenance PT; and iv) maintenance yoga and maintenance PT will both be superior to education. To test our hypotheses, we will perform longitudinal analyses on the pain and function outcomes incorporating all measurements across the study period, including baseline and 6, 12, 26, 40, and 52 weeks. We will perform a longitudinal profile analysis to compare the pattern of change in outcomes over the study period with a five-part treatment variable (yoga maintenance, yoga no maintenance, PT maintenance, PT no maintenance, education), time, and their interaction as predictors in the model. The initial model will assume an unstructured covariance to account for the correlation between repeated measures on an individual. A simpler model may be used for the covariance or the model for the means over time if found to be appropriate. No missing data will be replaced in these longitudinal analyses. All available patient data can be included and the analysis will be considered to be unbiased under a “missing at random” mechanism. Additional subgroup longitudinal analyses can be done using only data from those participants who were 12-week completers. We will also compare the change in pain medication usage over time using a generalized estimating equation approach or non-linear mixed effects model to account for the repeated measures of a dichotomous outcome. This approach will parallel the longitudinal analyses for continuous outcomes described above.

#### Adherence and per-protocol analyses

For the 12-week Treatment Phase, adherence is defined as follows: ≥75% attendance to recommended yoga classes (i.e., 9 or more); ≥73% attendance to PT sessions (i.e., 11 or more); self-reported completion of three-fourths or more of the assigned educational materials.

For the 40-week Maintenance Phase, adherence is defined as follows: ≥75% attendance to maintenance yoga classes for structured maintenance yoga participants and ≥4 (80%) maintenance PT sessions for structured maintenance PT participants. There is no formal adherence definition for the three other groups: no structured maintenance yoga, no structured maintenance PT, or education. However, for these groups, we collect self-report data on attendance to any non-study yoga/exercise/gym classes and home practice during the Maintenance Phase.

Per-protocol analyses will include those participants adherent to their assigned protocol. If a yoga participant starts PT, a PT participant practices yoga, or an education participant starts PT or yoga, that participant would also be excluded from the per-protocol analysis.

Additional sensitivity analyses will be performed to explore the potential role of concomitant interventions as follows:

•Analyze participants who were adherent with the protocol as defined above but also exclude any participants who received any operative procedures during the study (i.e., epidural steroid injection or back surgery).

•Analyze participants who were adherent with the protocol as defined above but excluding anyone who started new treatments for back pain (e.g., medication, chiropractic, acupuncture, massage).

•Create an ordinal variable for the number of different non-protocol treatments a participant uses during the study period and analyze participants who were adherent with the protocol as defined above while adjusting for this non-protocol treatment variable.

#### Cost effectiveness analyses

From the society perspective, a cost-utility analysis will be performed by comparing the incremental societal costs of one treatment arm over another (direct medical and non-medical costs plus productivity costs) to incremental effectiveness in terms of change in QALYs [52]. This is designated as the “reference case” and can be used by policy makers for the broad allocation of health resources [97,100]. For the third-party payer perspective, incremental costs will only include direct medical costs (direct intervention costs plus ongoing direct medical utilization costs) and will be compared to the incremental QALY impact. This analysis will help determine whether it makes economic sense for yoga to be a reimbursable service for this population. For the participant perspective, cost-effectiveness will be determined by comparing participants’ incremental out-of-pocket costs (travel, childcare, over-the-counter medications, use of non-reimbursed healthcare practitioners, co-payments, cost of yoga or PT sessions attended after the 12-week Treatment Phase by those not randomized to a structured maintenance programs) to their incremental QALY impacts. A bootstrap methodology will be used to estimate confidence intervals [101], and one-way sensitivity analyses will be performed to determine the robustness of our estimates to different assumptions such as to the wage rates used to value productivity [102]. Combined LBP and non-LBP-related healthcare utilization will be used for all of these analyses. Sensitivity analyses using LBP-related utilization only will also be performed. All cost effectiveness analyses will use the ITT principle.

For the Treatment Phase, the PT protocol calls for all PT participants to receive 15 total sessions in 12 weeks. Adherence to the PT protocol is defined as completing 11 or more sessions. However, it is conceivable that in a “real world” practice situation, many clinicians would discharge a patient after fewer sessions if, in the therapist’s clinical judgment, there had been substantial clinical improvement and the participant reached all desired exercise targets. To address this, we will conduct additional sensitivity analyses to explore whether the comparative cost effectiveness of PT vs. yoga would be substantially different if physical therapists stopped treating the patient if clinical improvement was reached prior to the end of the 12 weeks and 15 sessions. PT implementation costs for this sensitivity analysis would be limited to only those PT sessions provided up to and including a visit where improvement was reached. For the purpose of this sensitivity analysis, we will *a priori* define the time point for improvement for a PT participant to be when they meet both of the following criteria: a reduction of ≥6 points in the participant’s MODI score from baseline (six points is widely considered the minimal clinically significant improvement in the MODI [103]) and successfully able to complete all recommended exercises at the targeted number of repetitions as displayed in Table 5.

#### Analysis of safety

The incidence of adverse events, both overall and for specific events, will be compared between groups using a χ^2^ or Fisher’s exact test, as appropriate. Any participant attending at least one session of yoga or PT will be included in that group for purpose of analysis.

#### Exploratory analyses

We will also examine an alternative definition of “success” at 12 weeks, defined as ≥30% reduction in pain from baseline and ≥30% improvement in function from baseline [104]. Logistic regression will be used with “success” as the outcome and treatment and potential confounders as covariates. We will also compare the following exploratory outcomes using either linear or logistic regression, as appropriate, with treatment and potential confounders as predictors: fear-avoidance beliefs (FABQ), sleep quality, perceived stress, anxiety, depression, coping skills, self-efficacy, and use of non-study cLBP treatments.

#### Qualitative analyses

Initial transcriptions will be read to draft short interview summaries. To minimize coder drift and assure similar use of codes, an initial coding list will be developed based upon initial interview responses and experiences from our previous studies [26,28]. Two research assistants will draft a summary of predominant themes, and then refine and update the code list. Every third interview will be double coded. As line-by-line coding of transcripts progresses, the research team will meet regularly to update the code list and assure codes are being utilized reliably. Atlas.ti qualitative data analysis software will be used to manage the coding process. Ultimately, line-by-line codes will be grouped into larger categories and themes. Themes will be developed after reviewing code frequencies and merging the highest code yields into distinct thematic categories. Frequency of occurrence will be a means to determine the salience of a code.

#### Sample size considerations

For the co-primary endpoints of change in pain or function from baseline to week 12, we assume a one-sided α error = 0.05. If we assume a minimal clinically significant difference of 2.0 points for the 11-point pain intensity scale, then using a 1.0 point non-inferiority margin for pain is reasonable [103]. If we assume 3.0 points on the 23-point RMDQ scale is a minimal clinical significant difference, then choosing a non-inferiority margin of 1.5 for function is reasonable [105]. A common standard deviation of change in all groups is 2.5 points for the 11-point pain intensity scale and 4.35 points for the 23-point RMDQ function scale [24,26].

Using these assumptions, 108 participants in both the yoga and PT group would provide 90% power to detect whether yoga is truly non-inferior to PT in respect to effect on pain. Similarly, 107 participants in both the yoga and PT group would provide 81% power to detect whether yoga is truly non-inferior to PT in respect to effect on function. Using these sample sizes, we would be able to detect at least a 1.4 unit difference in pain or at least a 2.4 difference in function with 90% power for superiority of yoga or PT compared to education. We inflated these sample sizes in order to protect against an estimated dropout rate of 20%. With this adjustment, the target sample size for the yoga and PT groups is 128 participants each. Using a yoga:PT:education 2:2:1 randomization scheme, we need a total of 320 participants (128 yoga, 128 PT, 64 education). Thus, for the co-primary outcomes of pain and function at 12 weeks, we have adequate power to determine non-inferiority between yoga and PT and superiority of yoga and PT to education*.*

We also anticipate adequate power for the longitudinal analyses of the Maintenance Phase data. Incorporation of repeated measures over time increases power by increasing the “effective sample size,” which is a function of the number of participants and the number of total measurements (0, 6, 12, 26, 40, and 52 weeks). Participants who drop-out or who have missing data can still be included in these analyses.

### Compensation to participants

Participants receive honoraria for participating in the study in the form of store gift cards (e.g., Target, CVS). We give the cards to participants after they complete each survey, according to the following schedule: baseline $50, 6 weeks $50, 12 weeks $100, 26 weeks $50, 40 weeks $50, 52 weeks $100. Each participant interviewed for the qualitative analyses also receives a gift card worth $25. We also encourage participants to update their contact information regularly throughout the study if it changes by giving $5 gift cards for providing verifiable updated contact information (e.g., new cell phone number). Participants receive study interventions (yoga classes, PT sessions, educational materials) and materials (e.g., yoga supplies, PT home practice materials, education book) free of charge.

## Trial status

The study is currently recruiting participants.

## Abbreviations

ANOVA: Analysis of variance; BMC: Boston Medical Center; BUMC: Boston University Medical Campus; cLBP: Chronic low back pain; FABQ: Fear Avoidance Belief Questionnaire; ITT: Intention-to-treat; LBP: Low back pain; MODI: Modified Oswestry Disability Index; NEPT: New England Physical Therapy Plus, Inc.; PI: Principal investigator; PT: Physical therapy; QALY: Quality-adjusted life year; RCT: Randomized controlled trial; RMDQ: Modified Roland-Morris Disability Questionnaire; SF-36: Short Form-36 Questionnaire; TENS: Transcutaneous electrical nerve stimulation.

## Competing interests

The authors declare that they have no competing interests.

## Authors’ contributions

All authors made substantial contributions to the conception or design of the protocol described in this manuscript. Specifically, RBS conceived of the study and its design and coordination. KJS assisted with study design. AD and JS designed and participated in drafting the physical therapy protocol, training manuals, and home practice materials. PMH designed the cost effectiveness analysis. RP participated in the design of the qualitative components. JEK drafted study policies and procedures, and participated in the design of qualitative analyses. CJC developed data management and quality assurance protocols. CML scripted and produced the yoga home practice videos. CF and MB participated in the design of the yoga protocol and drafting of the yoga teacher and participant manuals. JW designed the quantitative data analysis plan and participated in study design. All authors helped draft or critically revise the manuscript for important intellectual content. All authors read and approved the final manuscript.

## Authors’ information

RBS is Associate Professor of Family Medicine at Boston University School of Medicine, Associate Professor of Epidemiology at Boston University School of Public Health, and Director of Integrative Medicine in the Department of Family Medicine at Boston Medical Center. KJS is a Senior Scientific Investigator at the Group Health Research Institute, epidemiologist, and clinical trialist with a focus on complementary medicine practices for low back pain. AD is Professor and Chairman of the Department of Physical Therapy at the University of Pittsburgh, School of Health and Rehabilitation Sciences. He helped develop the original treatment-based classification system for low back pain. PMH is a senior behavioral scientist at the RAND Corporation, resource economist, and licensed naturopathic doctor. JS is a chiropractic physician and doctoral student at the University of Pittsburgh Department of Physical Therapy. RP is Associate Professor of Social Work at the Boston University School of Social Work. She is a mixed methods researcher and director of the Family Therapy Certificate Program. JEK is an associate project manager at New England Research Institutes. CJC is a doctoral student at University of Southern California in Health Psychology. CML is a graduate student at Boston University in Medical Sciences and International Public Health. CF is a certified Iyengar yoga teacher with over 15 years of experience. MB is the founder of the Santosha School of Yoga in Providence, Rhode Island. JW is Professor of Biostatistics, Mathematics and Statistics at Boston University School of Public Health.

## Supplementary Material

Additional file 1 **Yoga Participant Guide.** A guidebook given to participants describing and depicting the yoga exercises for assistance during home practice. Click here for file

Additional file 2 **Yoga Teacher Training Manual.** A training and reference manual that includes a description of the yoga protocol, guidelines for teaching the exercises, and details of each yoga posture with associated modifications as necessary. Click here for file

Additional file 3 **Physical Therapy Training Manual.** A training and reference manual that includes a description of the physical therapy protocol and instructions for using the treatment-based classification algorithm. Click here for file

Additional file 4 **Education Newsletters.** One- to two-page newsletters mailed to education participants at 3, 6, 9, and 12 weeks written at a 6^th^ grade level that highlight main points from the assigned chapters of *The Back Pain Helpbook*[60]. Click here for file

Additional file 5 **Study Policies and Procedures Manual.** Comprehensive manual for research staff for operationalizing different study activities, including recruitment, enrollment, treatment interventions, data collection, qualitative research, and adverse events. Click here for file

Additional file 6 **Qualitative Interview Guides.** Semi-structured interview guides used to facilitate individual qualitative interviews with participants from each treatment arm. Click here for file
