# Supplementary material for: Yoga vs. physical therapy vs. education for chronic low back pain in predominantly minority populations: study protocol for a randomized controlled trial
Source: Trials. 2014 Feb 26;15:67. doi: 10.1186/1745-6215-15-67 (PMC3944007; doi:10.1186/1745-6215-15-67)
Supplement: Additional file 1 — Yoga Participant Guide. A guidebook given to participants describing and depicting the yoga exercises for assistance during home practice. [file 1745-6215-15-67-S1.pdf]

Participant Guidebook

Back to Health

A Study Comparing Yoga, Physical Therapy & Education for Chronic Low Back Pain

Table of Contents

Welcome……..……..3

Study Contact Information…………………………………………………………………………………...……………………….4

Tips to make the most out of your experience with the *Back to Health* study…………………………………………………..…. 5

How to Set up Your Home Practice…………………………………………………………………………………...………….…6

How to Fill Out Your Home Practice Log………………………...……………………………………...…………………..……………….7

Yoga Poses for Home Practice
Segment 1: Opening to Something Greater…………………………………………………………………………………….…8
Segment 2: Listening to the Body…………………………………………………………………………………......…. 12
Segment 3: Engaging your Power……………………………………………………………………………………...…...16
Segment 4: Bringing it Home………………………………………………………………………………………………..20

Yoga Pose Descriptions
Baby Dancer Pose…………………………………………………………………………………………………...…..24
Bridge Pose………..25
Cat/Cow Pose……………………………………………………………………………………………………...…..26
Chair Pose……...27
Chair Twist Pose: Standing…………………………………...………………………………………………..…………28
Chair Twist Pose: Seated………………………………………………………………………………………………….29
Child's Pose……….30
Cobra Pose……………………………………………………………………………………………………..…….31
Crescent Moon Pose…………………………………………………………………………………………...……….32
Downward Facing Dog Pose……………………………………………………………………...…………………….33
Extended Leg Pose…………………………………………………………………………..…………………………34
Knees to Chest Pose…………………………………………………………………...………………………….35
Knees Together Twist Pose………………………………………...………………………………………………….36
Locust Pose………………………………………………………...……………………………………………….37

© Boston Medical Center

Yoga Pose Descriptions con’t

Modified Chair Pose……………………………………………………………………………………………....…………………….38
Mountain Pose………………………………………………………………………………………………………..……………39
Pelvic Tilt Pose……………………………………………………………………………………..……………………..………..40
Reclined Chest Opener Pose………………………………………………………………………………….………………………41
Reclining Cobbler Pose……………………………………………………………………………...………………………….42
Svasana……..…………………...43
Shoulder Opener Pose……………………………………………………………………………....…………………………..44
Sphinx Pose……….45
Spinal Rock Pose……………………………………………………………………………..……...…………………………46
Standing Forward Bend at Wall Pose……………………………..…………………………...……………………………….47
Supported Bridge Pose……………………………..……………………………………...…………………………………….48
Table Top with Leg Extended Pose……...………………………………………………………………………..……………….49
Triangle Pose……………………………..………………………………………………………………………………………….50
Triangle at Wall Pose…………………...…………………………………………………………………………………………….51
Wall Dog Pose…………………………..………………………………………………………………………………………………….52
Warrior I Pose……………………………..………………………………………………………………………………………….53
Warrior I at Wall Pose ……….54

Welcome

Back to Health is a study that compares yoga, physical therapy, and education for lower back pain. To best answer this question, we ask you to be as committed as possible to the study. Please make your best effort to come to all the yoga classes, fill out all the questionnaires honestly, and practice yoga at home. We do realize that emergencies or unavoidable conflicts happen. If you cannot make a class, or have any concerns about the study, please contact the study staff.

We planned the yoga classes specifically for chronic low back pain. The yoga teachers are all experts in this form of yoga. The classes are aimed at people who have done little or no yoga before. Experts have designed the classes to be as safe as possible for people with chronic low back pain. Over 12 weeks, you will be gradually taught how to do the yoga exercises. Your safety is very important. If you feel that you have any worsened pain or injury during the study please let your yoga teacher or a member of our study team know as soon as possible. Please feel free to call us at **617.414.6248** if you have any questions or concerns.

We recommend all participants practice 30 minutes per day on days when you do not have class. This guidebook was created to assist you in your yoga practice. It will help you recreate what you are learning in class as you begin your home practice. All the poses you learn in class are described in this Guidebook along with helpful diagrams. Keep this booklet at home where you practice yoga.

© Boston Medical Center

Important Study Contact Information

If you cannot make a class or need to get in touch with a member of our study team, please call us at 617.414.6248

Other Study Contact Information,

Principal Investigator:

Robert B. Saper, MD MPH
Boston Medical Center, Department of Family Medicine
1 Boston Medical Center Place
Dowling 5 South
Boston, MA 02118
(617) 414-6276 (office), (617) 414-3345 (fax)
Email: Robert.Saper@bmc.org

Research Coordinators:

Research Co-coordinator: Chelsey Lemaster
617-414-6201 (office)
Research Co-coordinator: Daniel Do
617-414-4464 (office)
Treatment Coordinator: Shayna Egan
617-414-6248 (office)

Tips to make the most out of your experience with the *Back to Health* study

1. Wear loose comfortable clothing.

2. Practice 30 minutes every day you do not have yoga class.

3. Take off your shoes and socks for better grip and balance on the yoga mat.

4. Come to each class. If for any reason you need to miss a class, let the study staff know. We will try to help you make up the missed class.

5. Be sure to turn off cell phones and electronic devices during class and when you practice at home.

6. Do not eat before practicing yoga. It's best to practice on an empty stomach.

7. Ask for help whenever you have questions. The teachers are there to help you succeed.

8. Listen to your own body. Do not do anything that causes severe pain or you think may be unsafe.

9. Yoga is not a competition. Try not to compare yourself to others or judge yourself.

10. The use of the breath is what makes yoga different from other exercise. You should remember to take slow even yoga breaths with each pose.

11. If for any reason you cannot do a certain yoga exercise, let the teacher know. For each exercise there is an alternative or modification the teacher can show you.

12. Bring water if you would like.

© Boston Medical Center

How to set up your home practice

This book is designed to help you practice yoga at home. Home practice reinforces what you do in class. We suggest practicing at home for 30 minutes on the days you do not have class. Even though you are taking a class with others, your practice is your own – your experience is genuine and yours. Please bring any questions or concerns that come up during your home practice to your teacher when you have class. Here are a few suggestions to make your home yoga practice a success:

1. **Find space** at home where you can practice. If possible, leave your yoga items, such as your mat, block and strap, there. It needs to be large enough so you can stretch upwards as well as to the sides. Try to find a space that is free of clutter, quiet, and private.

2. **Pick a time** that you can be alone and not be interrupted. Often early morning or evening works best.

3. **Ask for support from your family and friends.** If you live with others, let them know about your need to practice yoga every day. Ask for their support. Ask them to please not interrupt you while you practice.

4. **Wear comfortable clothing** that you can move in and take your socks off for better balance and grip on your mat.

5. **Make your practice area personal and attractive**. Place a few items where you practice that have special meaning for you. Keep the space clean and attractive with objects like flowers or a candle. Play calming music to aid in relaxation.

© Boston Medical Center

How to fill out your home practice log

1. Begin filling out your weekly home practice log the day after class.
2. Simply check whether or not you have practiced yoga at home and for how many minutes each day.
3. You may wish to write comments about how your home practice went or to keep track of which day you have class.
4. Please bring your home practice log to class and hand it in each week.

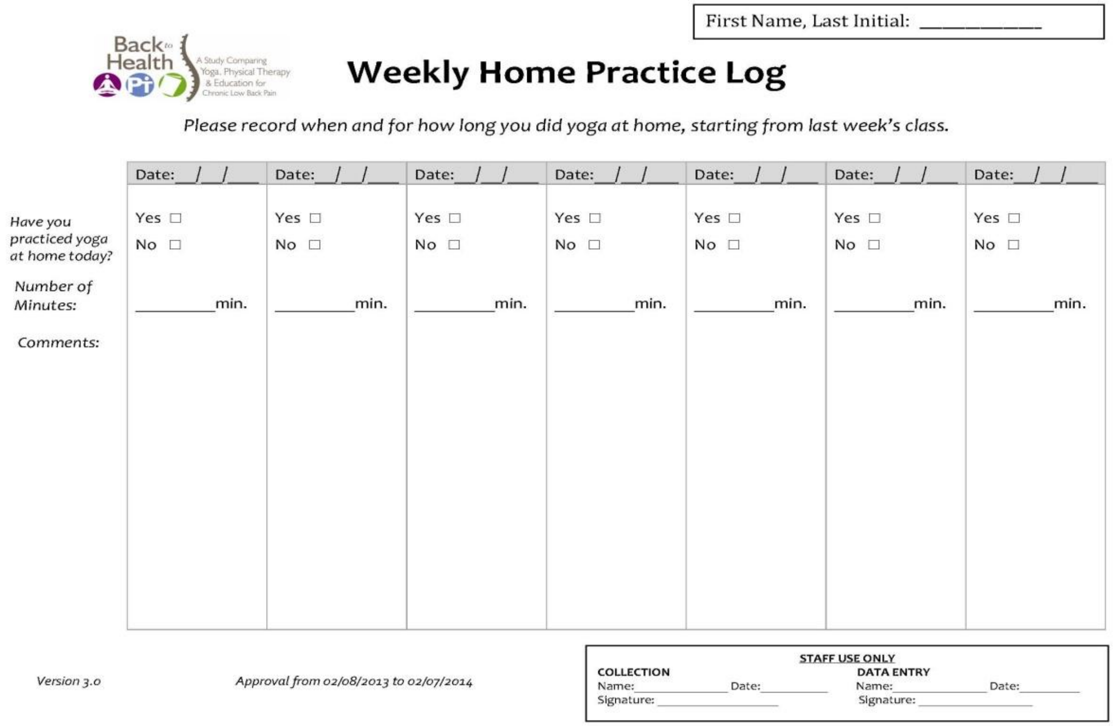

First Name, Last Initial: ___________

Back to Health — A Study Comparing Yoga, Physical Therapy & Education for Chronic Low Back Pain

Weekly Home Practice Log

Please record when and for how long you did yoga at home, starting from last week's class.

	Date:__/__/___	Date:__/__/___	Date:__/__/___	Date:__/__/___	Date:__/__/___	Date:__/__/___	Date:__/__/___
Have you practiced yoga at home today?	Yes ☐ No ☐	Yes ☐ No ☐	Yes ☐ No ☐	Yes ☐ No ☐	Yes ☐ No ☐	Yes ☐ No ☐	Yes ☐ No ☐
Number of Minutes:	________min.	________min.	________min.	________min.	________min.	________min.	________min.
Comments:							

Version 3.0 *Approval from 02/08/2013 to 02/07/2014*

STAFF USE ONLY
COLLECTION Name:__________ Date:________ Signature: __________
DATA ENTRY Name:__________ Date:________ Signature: __________

Yoga Poses for Home Practice

Segment One (Weeks 1-3): Opening to Something Greater

Please select a few of the following warm-ups to start your home practice.

Warm-Ups

1. Knees to Chest Pose
2. Knees Together Twist Pose
3. Pelvic Tilt Pose
4. Cat/Cow Pose
5. Mountain Pose
6. Modified Chair Pose
7. Shoulder Opener Pose
8. Modified Crescent Moon Pose
9. Chair Twist Pose: Standing

After the warm-ups, follow with the yoga postures.

Yoga Postures

1. Child's Pose
2. Locust Pose (Option 1 Only)
3. Sphinx Pose
4. Bridge Pose

Following the Yoga Postures, finish with these cool down postures.

Cool-Downs

1. Knees to Chest Pose
2. Knees Together Twist Pose
3. Reclining Cobbler Pose

End your practice with a short relaxation

Relaxation

1. Svasana

© Boston Medical Center

Segment One: Warm-Up Postures

Chose a few warm-ups to start your practice

Knees to Chest

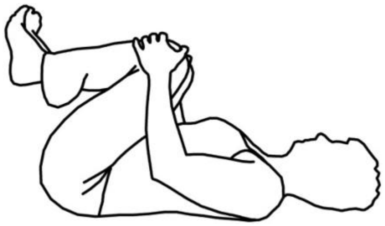

Knees Together Twist

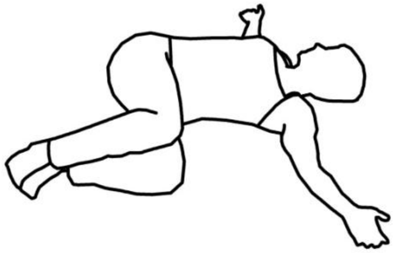

Pelvic Tilt

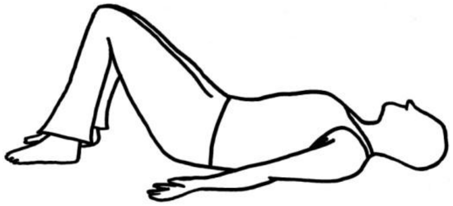

Cat/Cow

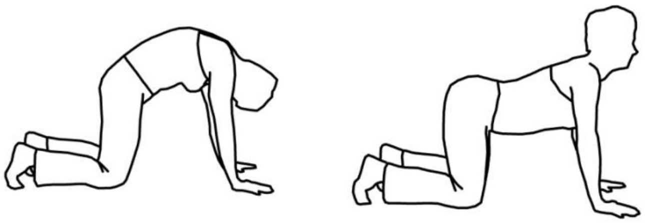

Mountain

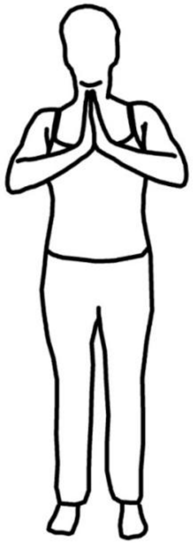

Modified Chair

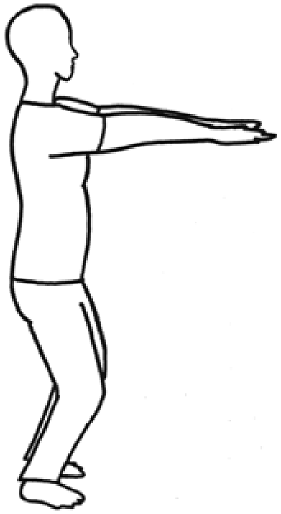

Shoulder Opener

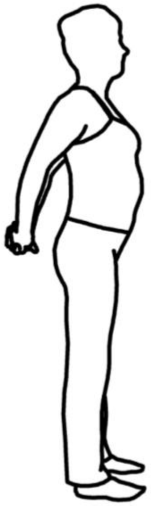

Modified Crescent Moon

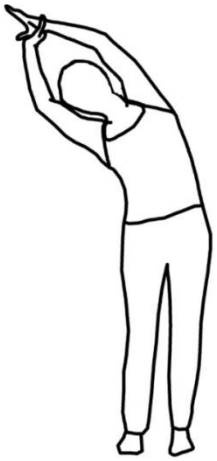

Chair Twist: Standing

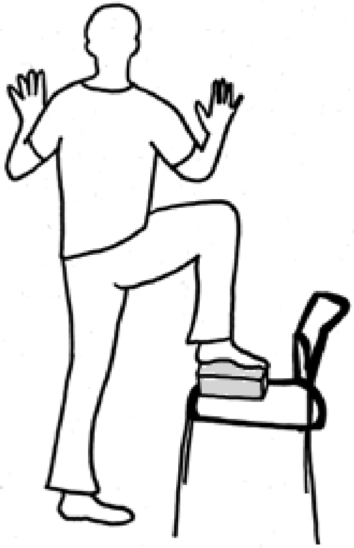

© Boston Medical Center

Segment One: Yoga Postures

1. Child's Pose

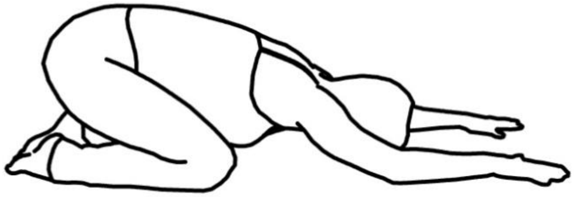

2. Locust Pose

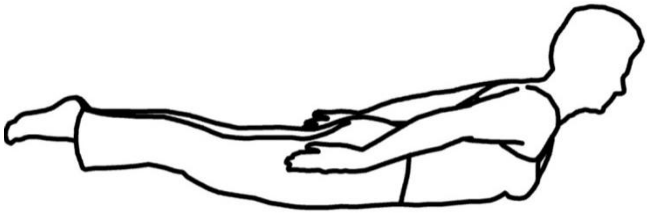

3. Sphinx Pose

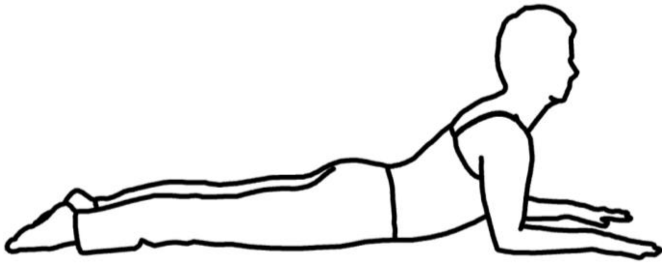

4. Bridge Pose

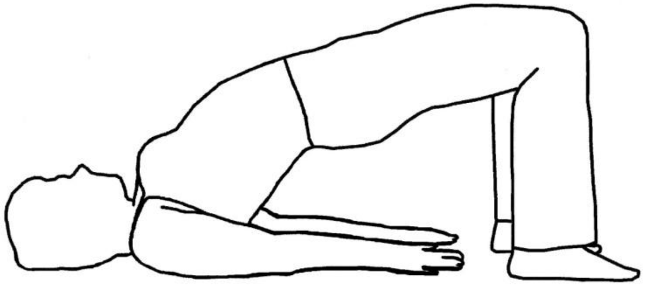

© Boston Medical Center

Segment One: Cool-Down Postures

Knees to Chest

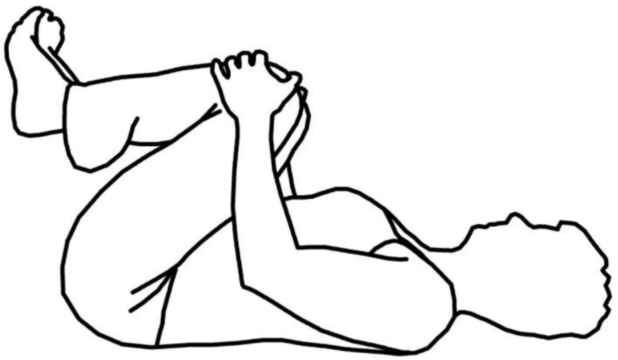

Knees Together Twist

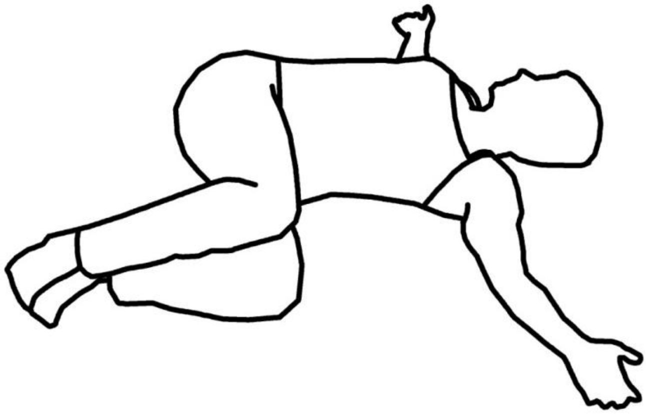

Reclining Cobbler

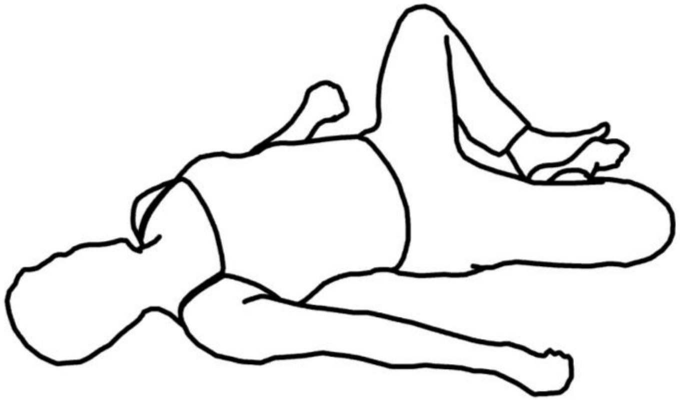

© Boston Medical Center

Segment Two (Weeks 4-6): Listening to the Body

Please select a few of the following warm-ups to start your home practice.

Warm-Ups

1. Knees to Chest Pose
2. Knees Together Twist Pose
3. Pelvic Tilt Pose
4. Cat/Cow Pose
5. Child's Pose
6. Mountain Pose
7. Modified Chair Pose
8. Shoulder Opener Pose
9. Modified Crescent Moon Pose
10. Chair Twist Pose: Seated

After the warm-ups, follow with the yoga postures.

Yoga Postures

1. Wall Dog Pose
2. Triangle At Wall Pose
3. Standing Forward Bend at Wall Pose
4. Locust Pose
5. Sphinx Pose
6. Cobra Pose
7. Bridge Pose

Following the Yoga Postures, finish with these cool down postures.

Cool-Downs

1. Supported Bridge Pose
2. Knees to Chest Pose
3. Extended Leg Stretch
4. Knees Together Twist
5. Reclining Cobbler Pose

End your practice with a short relaxation

Relaxation

1. Savasana

© Boston Medical Center

Segment Two: Warm-Up Postures

Chose a few warm-ups to start your practice

Knees to Chest

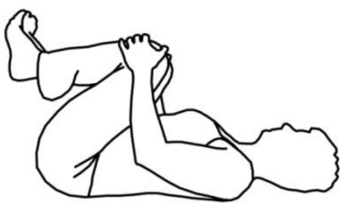

Knees Together Twist

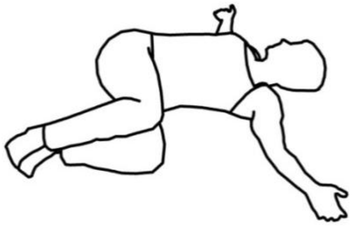

Pelvic Tilt

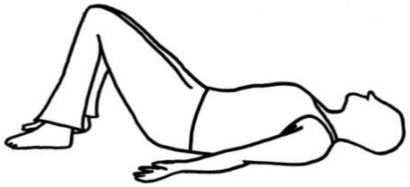

Cat/Cow

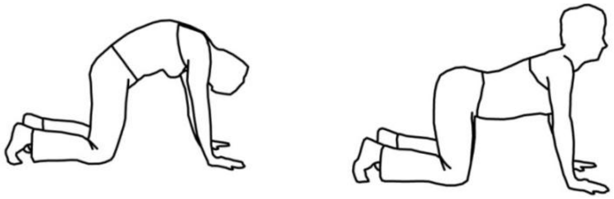

Child's Pose

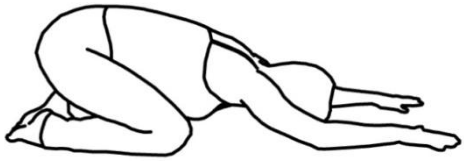

Mountain

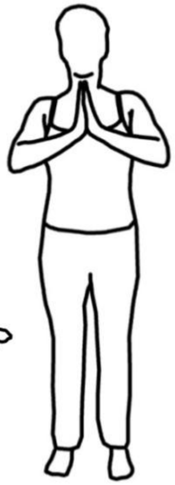

Modified Chair

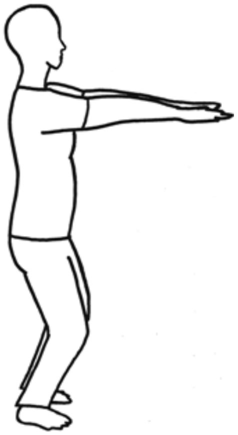

Shoulder Opener

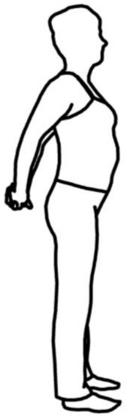

Modified Crescent Moon

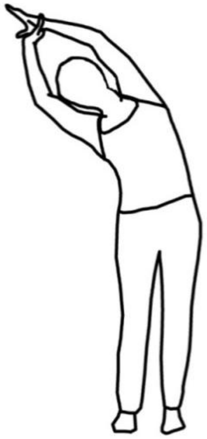

Chair Twist: Seated

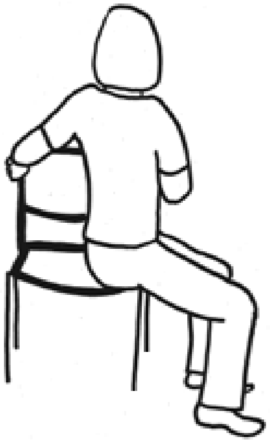

© Boston Medical Center

Segment Two: Yoga Postures

1. Wall Dog Pose

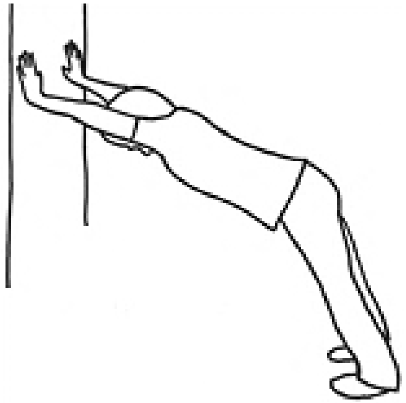

2. Triangle at Wall Pose

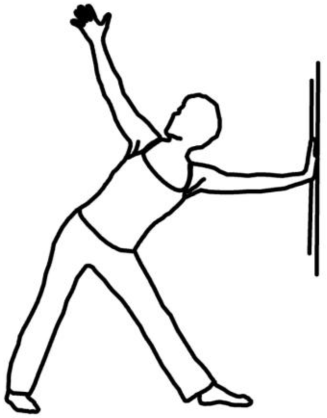

3. Standing Forward Bend at Wall

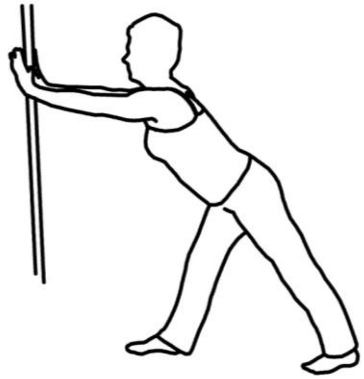

4. Locust Pose

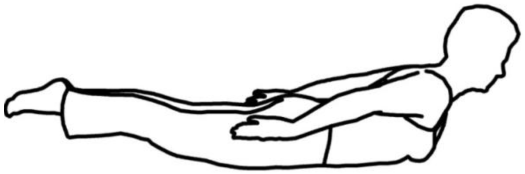

5. Sphinx Pose

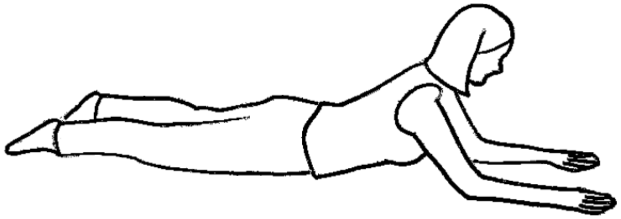

6. Cobra Pose

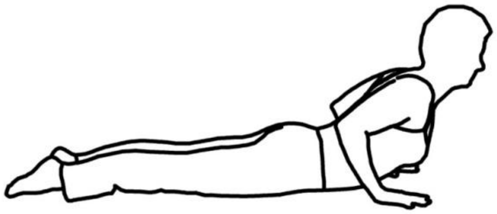

7. Bridge Pose

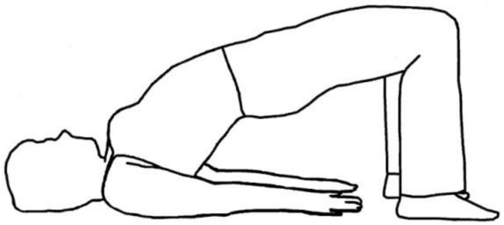

© Boston Medical Center

Segment Two: Cool-Down Postures

Supported Bridge Pose

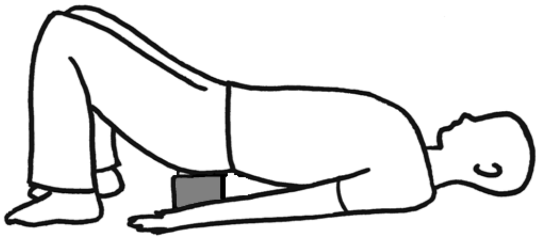

Knees to Chest Pose

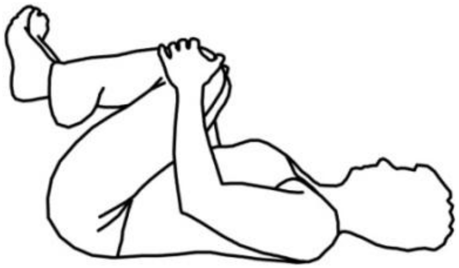

Extended Leg Stretch Pose

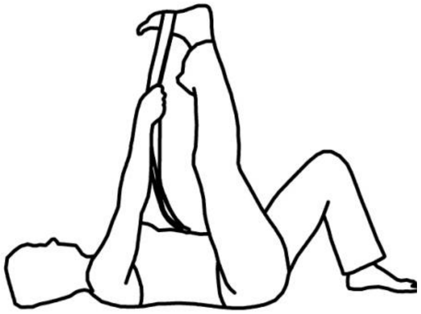

Knees Together Twist Pose

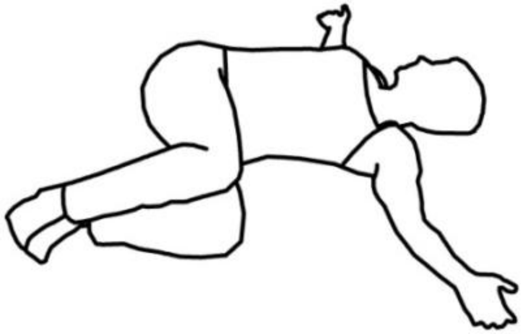

Reclining Cobbler Pose

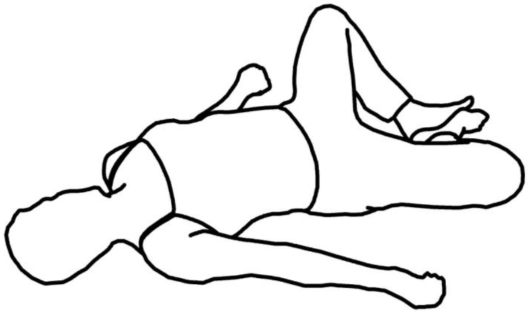

© Boston Medical Center

Segment Three (Weeks 7-9): Engaging Your Power

Please select a few of the following warm-ups to start your home practice.

Warm-Ups

1. Knees to Chest Pose
2. Knees Together Twist Pose
3. Table Top with Leg Extended Pose
4. Child's Pose
5. Mountain Pose
6. Shoulder Opener Pose
7. Modified Crescent Moon Pose

After the warm-ups, follow with the yoga postures.

Yoga Postures

1. Chair Pose
2. Wall Dog Pose
3. Triangle At Wall Pose
4. Standing Forward Bend at Wall Pose
5. Chair Twist Pose: Seated
6. Sphinx Pose
7. Cobra Pose
8. Bridge Pose

Following the Yoga Postures, finish with these cool down postures.

Cool-Downs

1. Extended Leg Stretch Pose
2. Knees to Chest Pose
3. Knees Together Twist
4. Reclining Cobbler Pose

End your practice with a short relaxation

Relaxation

1. Savasana

Segment Three: Warm-Up Postures

Chose a few warm-ups to start your practice

Knees to Chest

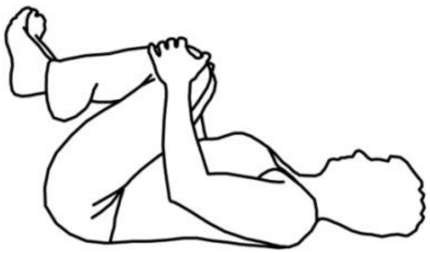

Knees Together Twist

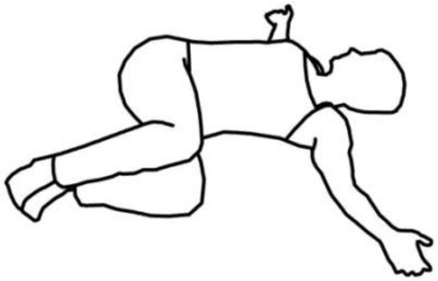

Table Top with Leg Extended

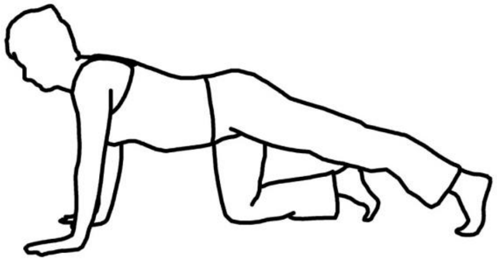

Child's Pose

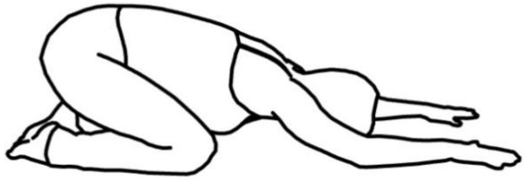

Mountain

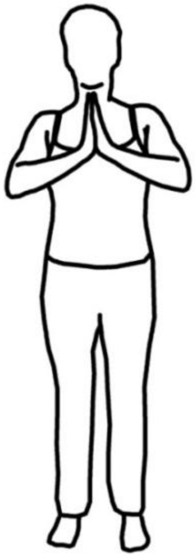

Shoulder Opener

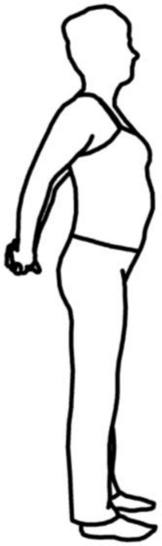

Modified Crescent Moon

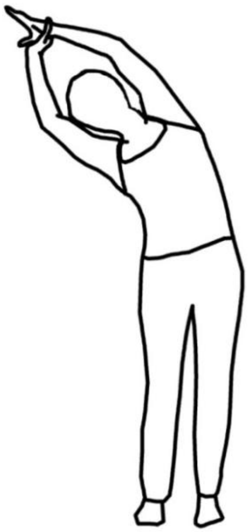

© Boston Medical Center

Segment Three: Yoga Postures

1. Chair Pose

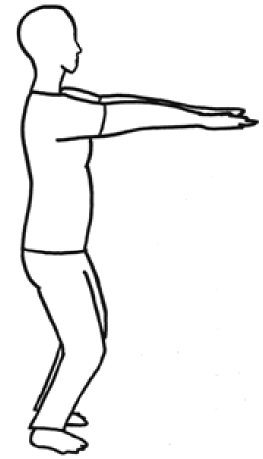

2. Wall Dog Pose

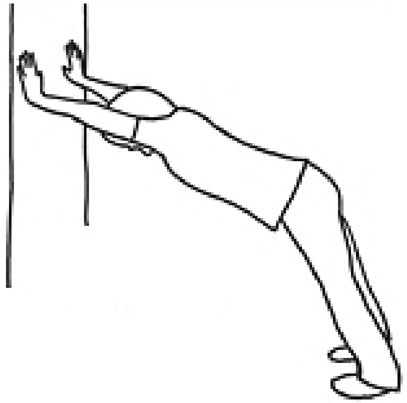

3. Triangle at Wall Pose

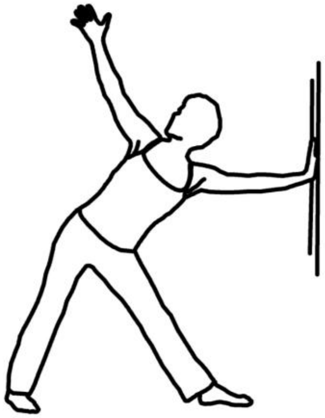

4. Standing Forward Bend at Wall Pose

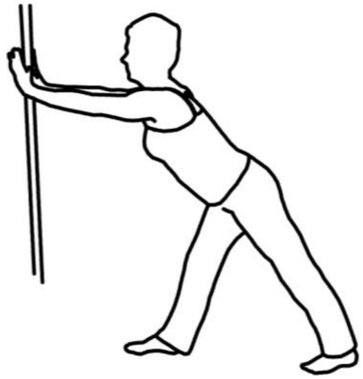

5. Chair Twist Pose: Seated

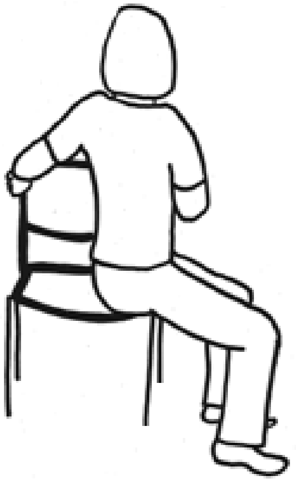

6. Sphinx Pose

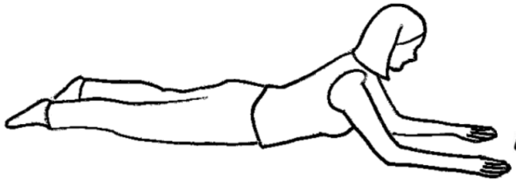

7. Cobra Pose

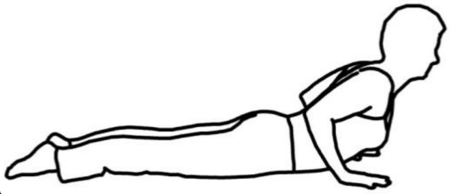

8. Bridge Pose

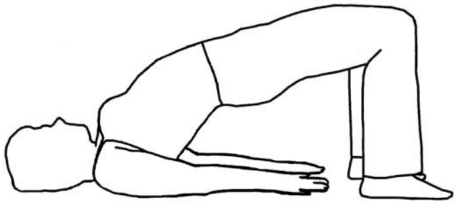

© Boston Medical Center

Segment Three: Cool-Down Postures

Extended Leg Stretch Pose

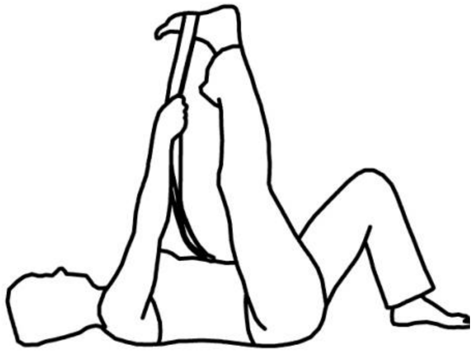

Knees to Chest Pose

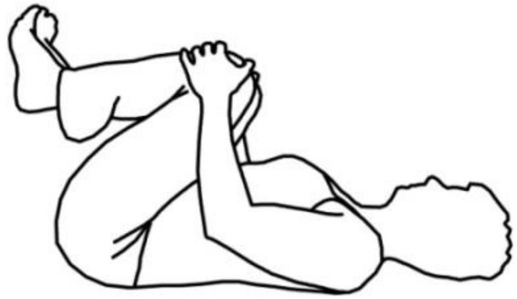

Knees Together Twist Pose

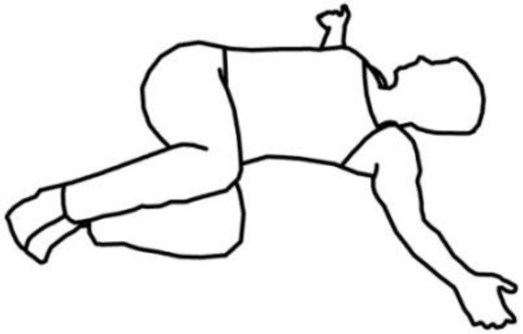

Reclining Cobbler Pose

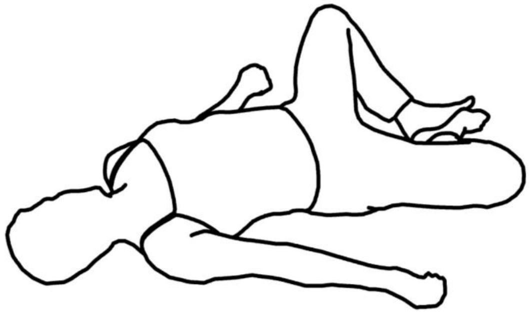

© Boston Medical Center

Segment Four (Weeks 10-12): Bringing it Home

Please select a few of the following warm-ups to start your home practice.

Warm-Ups

1. Knees to Chest Pose
2. Knees Together Twist Pose
3. Extended Leg Stretch Pose
4. Table Top with Leg Extended Pose
5. Mountain Pose
6. Shoulder Opener Pose

After the warm-ups, follow with the yoga postures.

Yoga Postures

1. Chair Pose
2. Warrior I Pose
3. Baby Dancer Pose
4. Standing Forward Bend at Wall Pose
5. Downward Facing Dog Pose
6. Locust Pose
7. Cobra Pose
8. Child's Pose

Following the Yoga Postures, finish with these cool down postures.

Cool-Downs

1. Knees to Chest Pose
2. Knees Together Twist
3. Reclining Chest Opener Pose
4. Reclining Cobbler Pose

End your practice with a short relaxation

Relaxation

1. Savasana

© Boston Medical Center

Segment Four: Warm-Up Postures

Chose a few warm-ups to start your practice

Knees to Chest

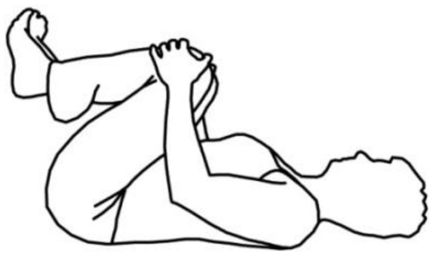

Knees Together Twist

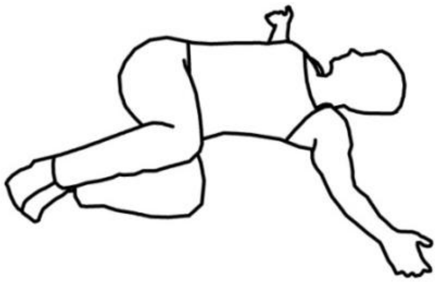

Table Top with Leg Extended

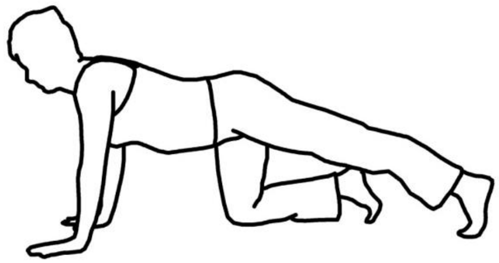

Extended Leg Stretch Pose

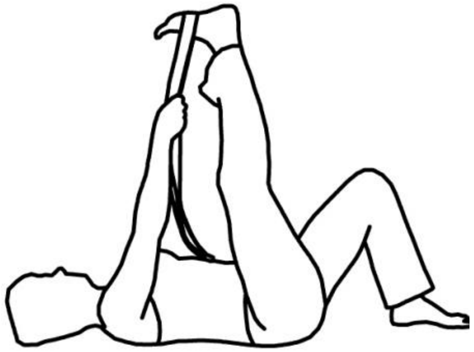

Mountain

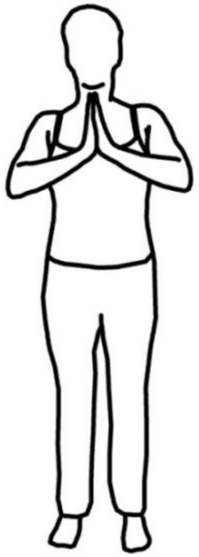

Shoulder Opener

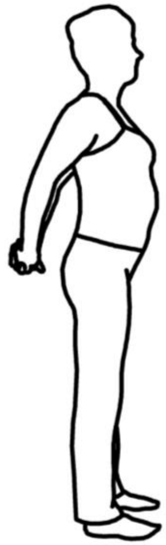

© Boston Medical Center

Segment Four: Yoga Postures

1. Chair Pose

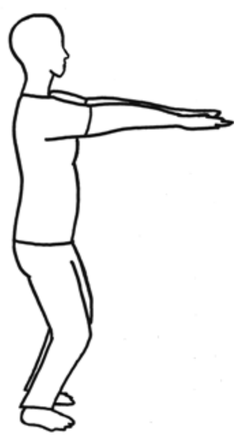

2. Warrior I Pose

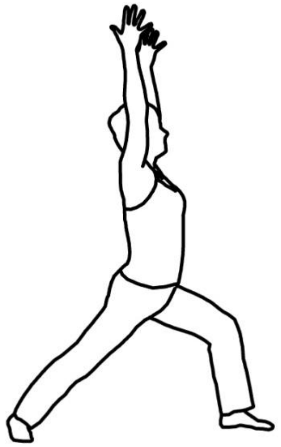

3. Baby Dancer Pose

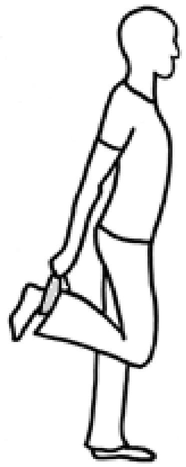

4. Standing Forward Bend at Wall Pose

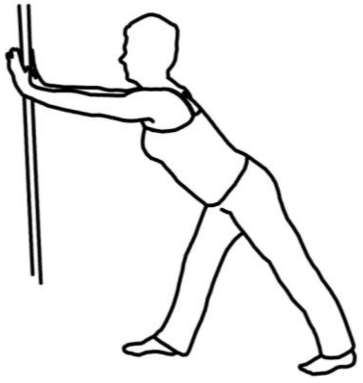

5. Downward Facing Dog Pose

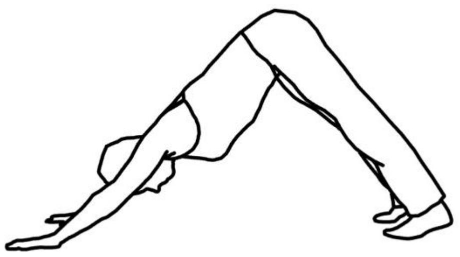

6. Locust Pose

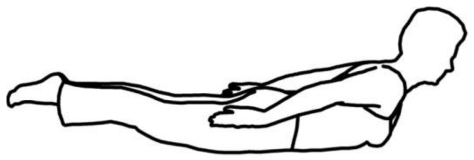

7. Cobra Pose

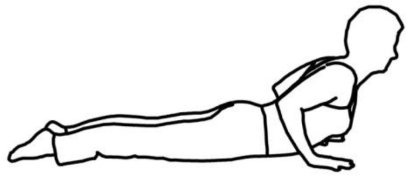

8. Child's Pose

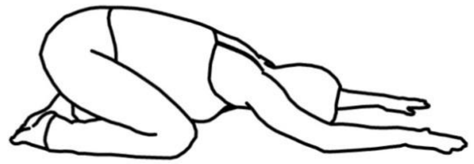

© Boston Medical Center

Segment Four: Cool-Down Postures

Knees to Chest Pose

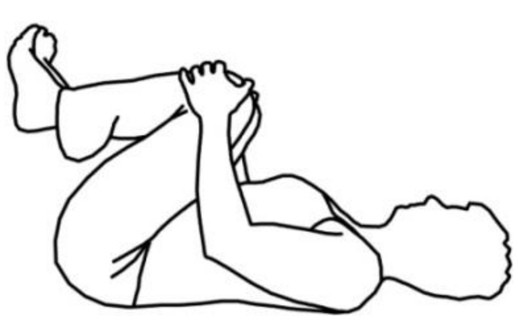

Knees Together Twist Pose

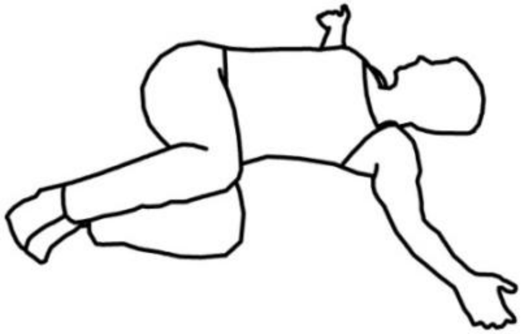

Reclining Chest Opener Pose

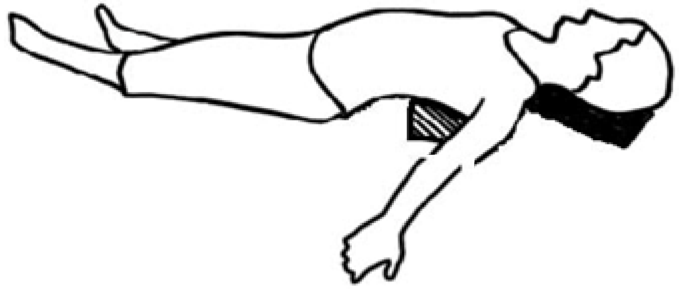

Reclining Cobbler Pose

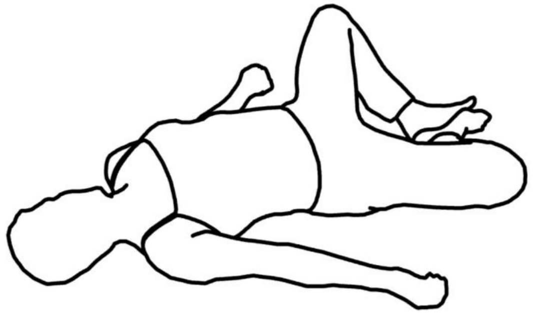

© Boston Medical Center

Baby Dancer Pose

Instructions for Yoga Pose

1. Start in Mountain Pose (p39) with the left side of your body against a wall and your left hand on the wall.
2. Shift your weight onto the left leg and bend your right knee so that your right foot is behind you.
3. Lift your right foot and hold either your foot or ankle with your right hand.
4. Raise your left hand while still touching the wall.
5. Release your right foot and come back to Mountain Pose.
6. Repeat on the other side, with the right side of your body against the wall.

Other Options for Pose

1. If you have difficulty reaching back far enough to hold your foot, use a yoga strap to help. Wrap the strap around the right foot and hold the other end of the strap with the right hand.
   To do the pose in a chair: Sit on edge of chair, take one foot back to the side of the chair and let the knee drop down to face the floor. If able, hold ankle or place a belt around ankle.

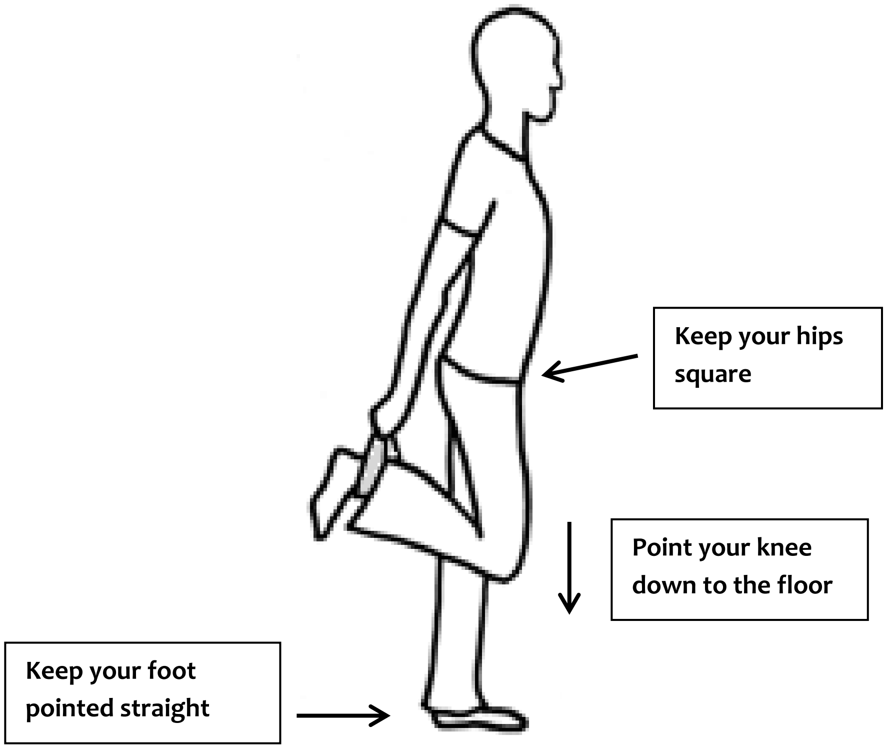

© Boston Medical Center

Bridge Pose

<u>Instructions for Yoga Pose</u>

1. Lie on your back with your knees bent and your feet as close to your hips as possible. Keep your knees, and feet parallel.
2. Press your lower back and feet into the mat and lift your tailbone up.
3. Keep lifting from the back of your thighs until your back is off of the floor.
4. Slowly roll back down to the mat starting with the upper back.

If the pose causes back pain, keep your back on floor and do gentle pelvic tilts.

<u>Other Options for Pose</u>

1. If there is pain in your knees, walk feet further away from hips.
2. Place a block between knees or a belt around thighs to keep thighs parallel.
3. To do the pose in a chair: With your hands on a chair seat or holding the arms of the chair, lean back and lift the hips up off the chair, making sure not to lift from the lower back but instead lift from backs of thighs. If you cannot put weight on your hands or arms, do Pelvic Tilt in a chair.

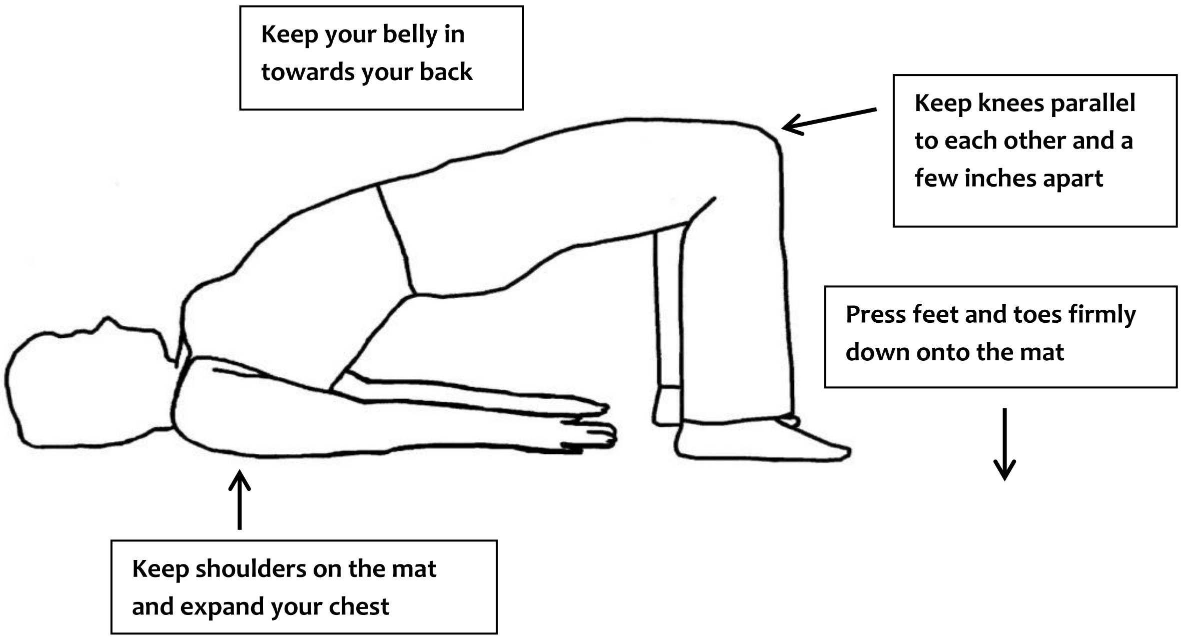

© Boston Medical Center

Cat/Cow Pose

Instructions for Yoga Pose

Cat Pose:

1. Begin on all fours with the back flat. Place your hands on the mat directly under the shoulders and your knees under your hips.
2. Press your hands into the mat and spread your fingers.
3. Exhale, lower your chin to your chest and round your back.

Cow Pose:

1. From Cat Pose, lift your head and your tailbone toward the ceiling.

To prevent injury, make sure to look forward or slightly down. Looking up to the ceiling *can* cause neck injury if the head bends too far backwards.

Other Options for Pose

1. If your wrists are uncomfortable, make a fist with both hands, thumbs facing down.
2. If your knees or hands are uncomfortable, roll the sides of the mat under your knees or the top of the mat under your hands.
3. To do the pose in a chair: Sit on a chair with your feet under your knees and knees hip width apart. Exhale, make your back round and tuck your chin toward your chest. Inhale and straighten your back. Exhale, gently lift head and chest up, slightly arching the back. Repeat several times. Rest, sitting straight up.

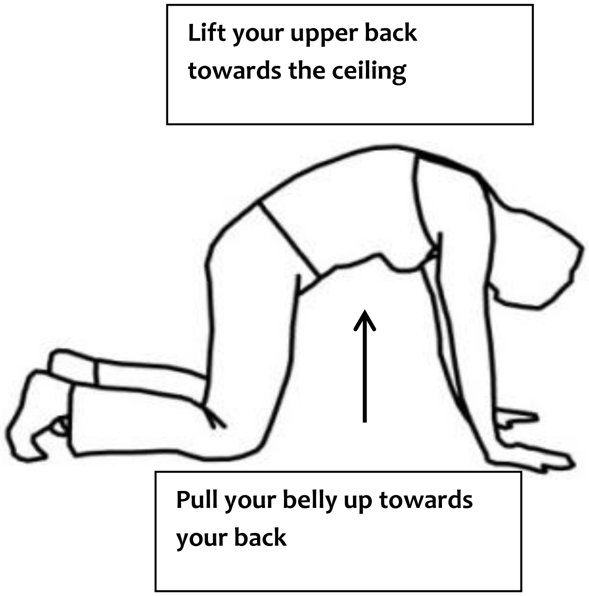

Cat

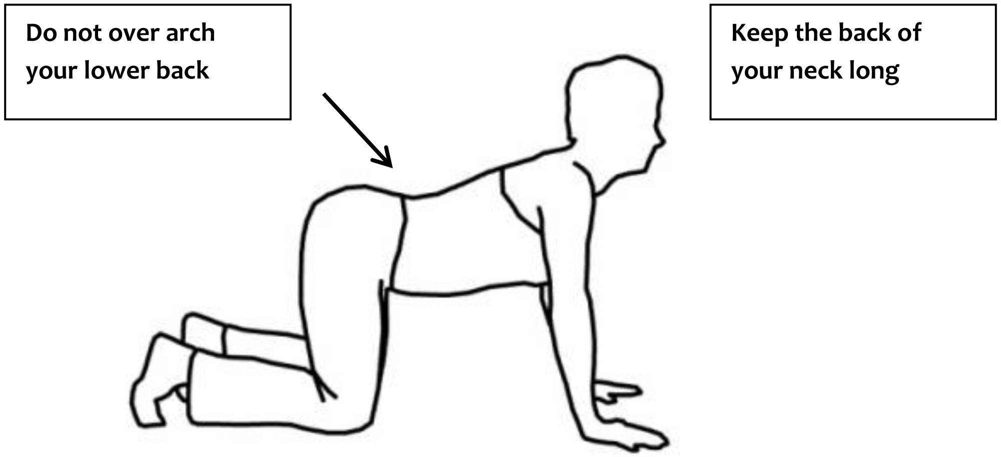

Cow

© Boston Medical Center

Chair Pose

<u>Instructions for Yoga Pose</u>

1. From Mountain Pose (p39), place your hands on your hips, and bend your knees as if you are about to sit in a chair.
2. Move your weight back onto your heels and bring your stomach in, while raising your above your head.
3. Straighten your legs and bring your arms back down.

<u>Other Options for Pose</u>

1. Keep your hands on your waist
2. Raise arms out in front to shoulder height
3. Stand with your back on a wall and your feet and knees together facing away from the wall. Bend your knees, making sure you're your knees are pointing over feet and press your lower back into wall. Slowly raise your arms up over your head.
4. Face the wall, using it to support you while you do Chair Pose with your fingertips on the wall.
5. To do the pose in a chair: Sit on the edge of a chair with your feet under your knees. Lean slightly forward, moving your body weight onto your feet. While remaining seated, raise arms over head, keeping your chest lifted and your tailbone moving down towards the sea of the chair.

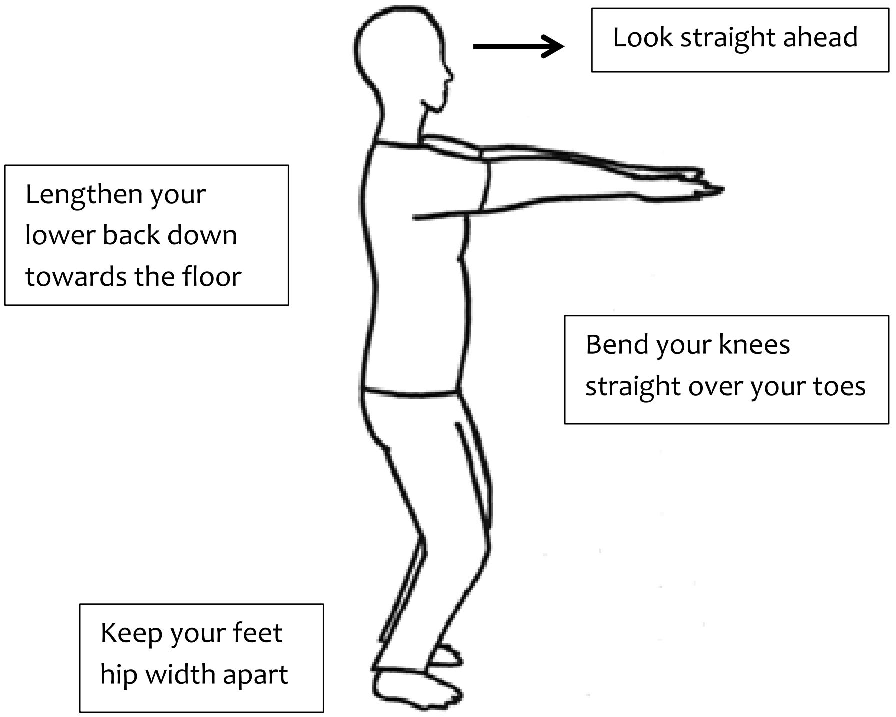

© Boston Medical Center

Chair Twist Pose: Seated

<u>Instructions for Yoga Pose</u>

1. Sit sideways on a chair so that the right side of the body is facing the back of the chair,
2. Sit on the edge of the chair so that the feet are firmly on the floor hips width apart.
3. Raise your arms up and turn to the right towards the back of the chair.
4. Bring your arms down, and place your hands on the back of the chair.
5. Relax and breathe.
6. Gently turning back to face forward.
7. Breathe and repeat on the left side.

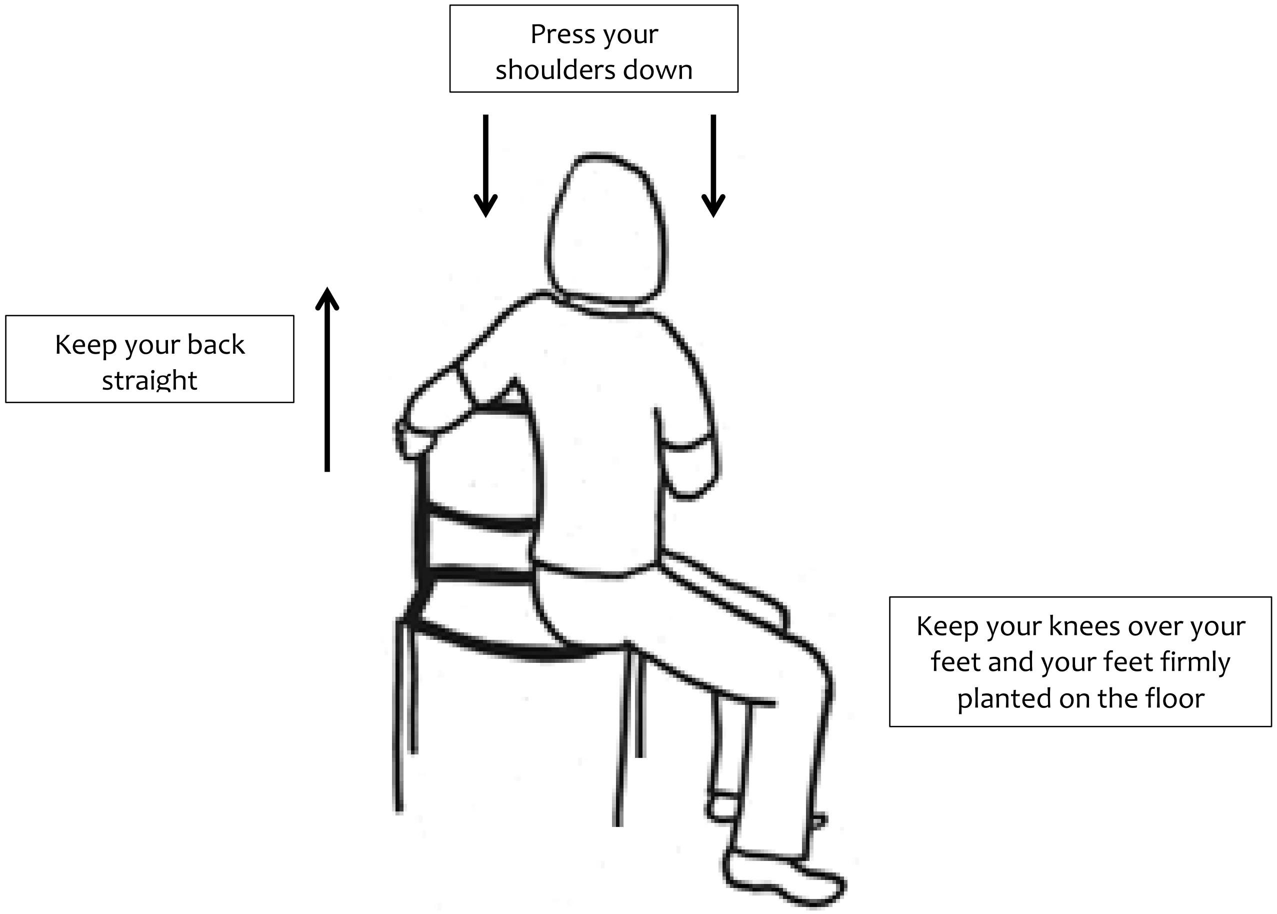

© Boston Medical Center

Chair Twist Pose: Standing

Instructions for Yoga Pose

1. Stand with the left side of the body against the wall and the right foot on a block on a chair or directly on the seat of the chair.
2. Turn and place your hands on the wall at the same height as your shoulders.
3. Press your hands into the wall and move your shoulders down.
4. Turn your body back to the chair and step your foot down.
5. Move the chair to the other side and repeat on with the left foot on the chair.

Other Options for Pose

1. For shorter people, place the foot directly on the chair seat, omitting the block under the foot.
2. With left hand hold right knee in place while turning.
3. Place a block between the wall and the outside of the knee.

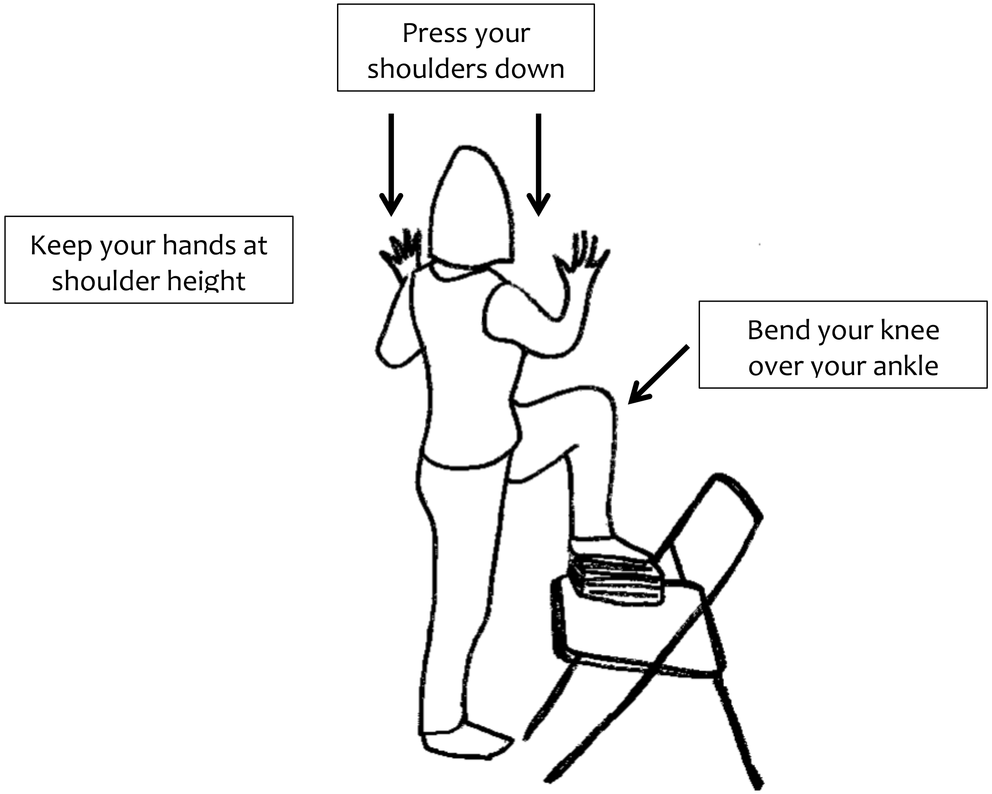

Child's Pose

<u>Instructions for Yoga Pose</u>

1. Start kneeling on the floor.
2. Moves knees apart from one another, while keeping your toes together.
3. Bend forward and bring your forehead towards the floor and sit back towards your feet while moving your arms overhead or to the side.

<u>Other Options for Pose</u>

1. If there is pain in your knee, place a folded blanket behind your knees.
2. If there is pain in your ankle, place a rolled blanket under your ankles.
3. If your feet cramp, keep your toes curled under your feet.
4. To do the pose in a chair: Sitting in a chair place your feet firmly on floor, hips distance apart. With hands on your thighs, palms down, slowly bend forward between your legs. Your hands can stay on your thighs or move to the floor or blocks.

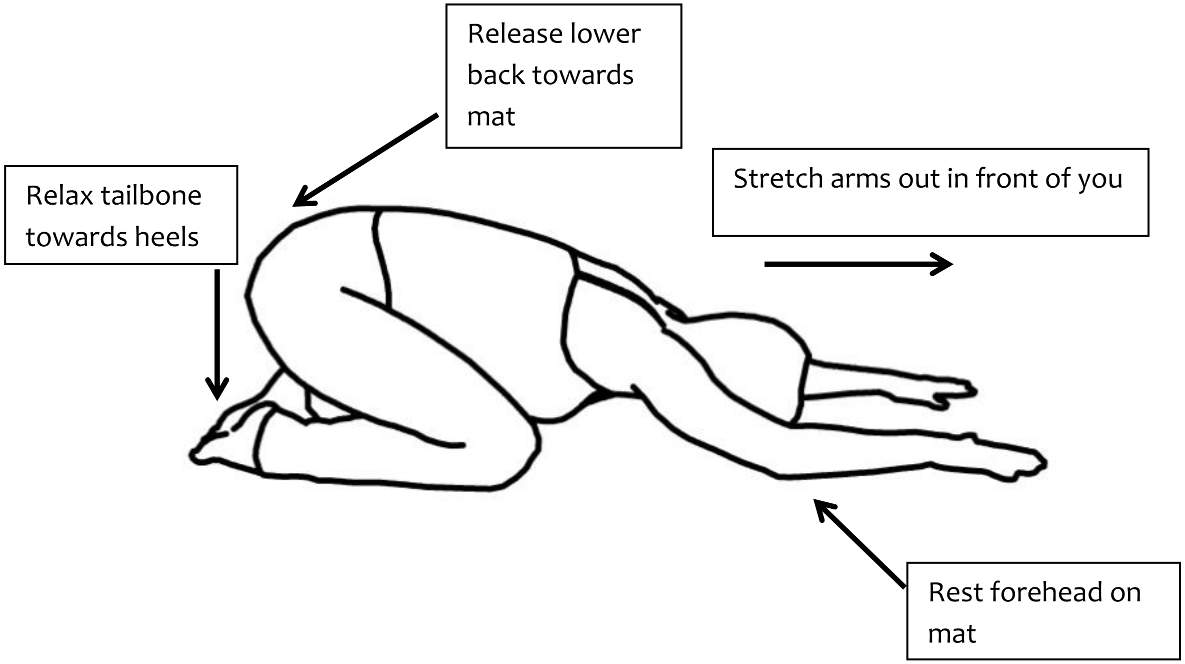

Cobra Pose

Instructions for Yoga Pose

1. Lie on your stomach with your chin or forehead on the mat and your hands at shoulder level with your palms face down on the mat.
2. Point your feet behind you with your toes touching the mat.
3. Press down into the mat with your hands and lift first your head and then chest. **Look forward and not up**.

To prevent injury, make sure to look forward or slightly down. Looking up to the ceiling can cause neck injury if the head bends too far backwards.

Other Options for Pose

1. To do the pose in a chair: Sit in a chair and place feet firmly on floor with your hands on your thighs and allow your shoulders to drop down and away from your ears. Roll your shoulders back to open your chest. Squeeze shoulder blades slightly together and pull stomach in towards your back.

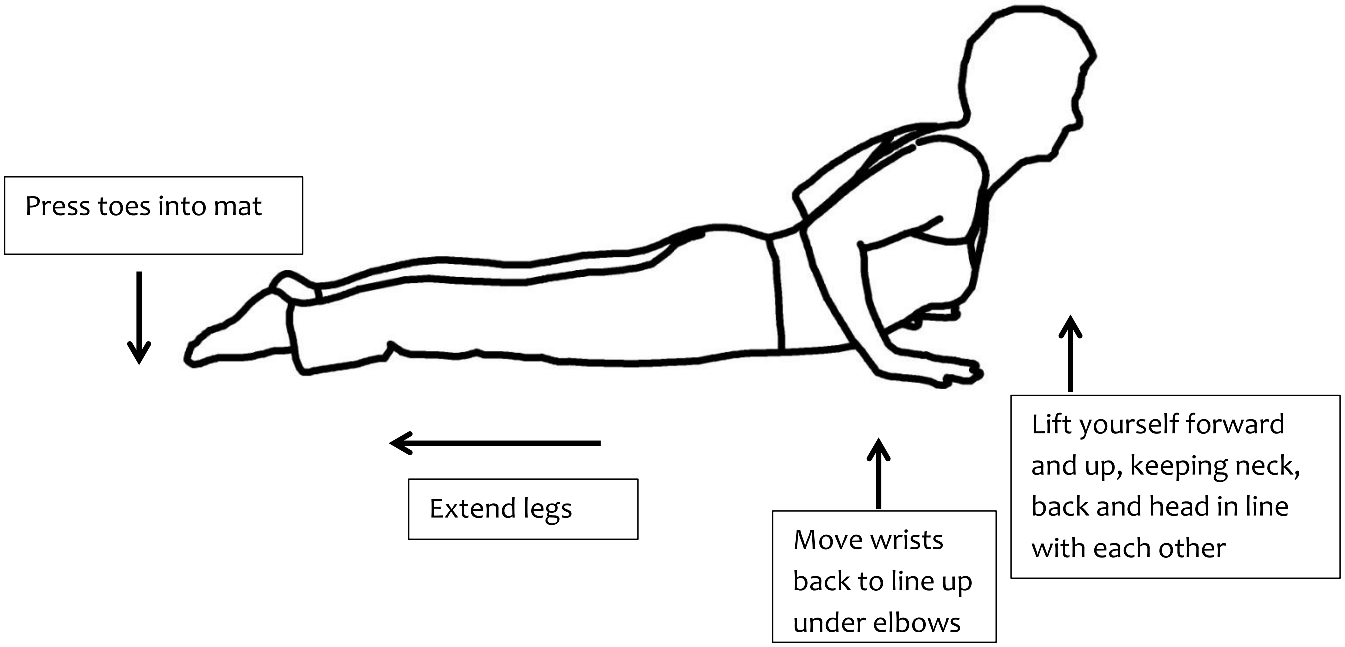

© Boston Medical Center

Crescent Moon Pose

Instructions for Yoga Pose

1. Start in Mountain Pose (p39) and move your arms up over your head.
2. Hold your left wrist with your right hand and bend slowly to the right.
3. Come back to the center and repeat on the other side, holding your right wrist with your left hand.

Other Options for Pose

1. While bending to the right, move your left arm up and over head toward the right with the palm facing the floor, keeping the right hand on the waist. Repeat on the left side.
2. Keep both hands on the waist while bending sideways.
3. To do the pose in a chair: Sit upright in a chair with back straight, move your arms over your head. Hold your left wrist with your right hand. Bend slowly to the right moving the left arm up and over to the right and then come back to the middle. Hold the right wrist with the left hand and bend to the left.

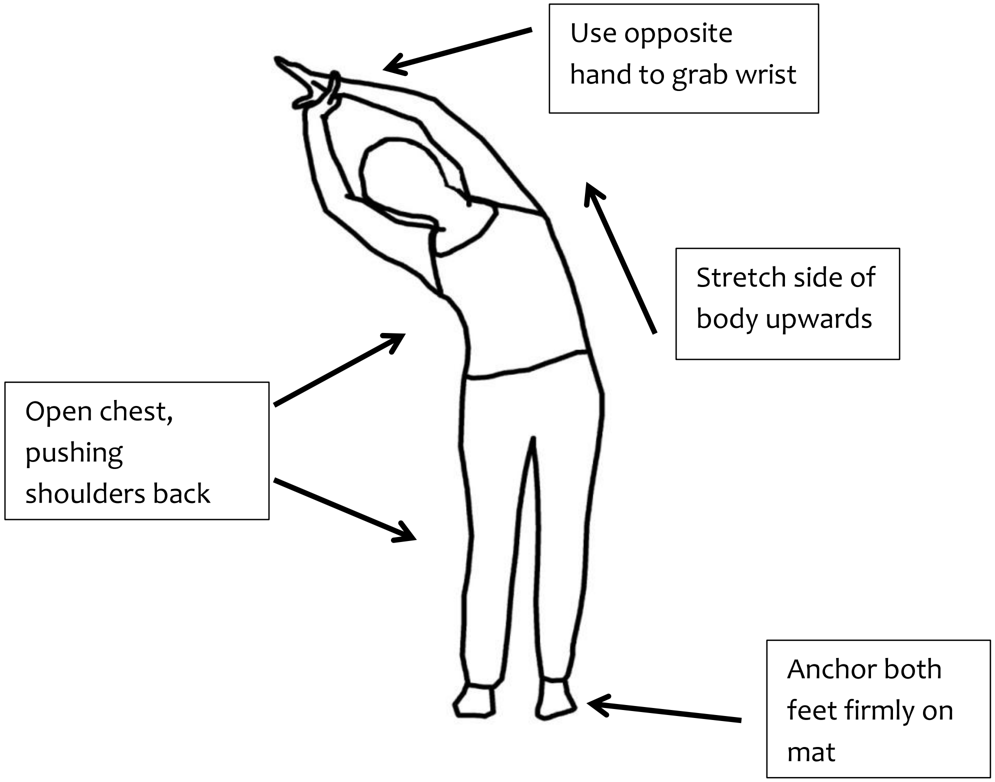

Downward Facing Dog Pose

Instructions for Yoga Pose

1. Start on your hands and knees with your hands directly under your shoulders and your knees directly under your hips.
2. Walk your hands forwards a few inches and press your palms into the mat.
3. Lift your hips and straighten your legs while pressing your heels down towards the floor.
4. Come back to your hands and knees.

Other Options For Pose

1. Wall dog (p52).
2. Place a chair at the wall. Bending over, hold the sides of the chair seat and step back until body is in a "V" shape. Place feet the same distance apart as hands.
3. To do the pose in a chair: Sit on edge of a chair, extend both legs out, hip width apart and raise both arms straight up over head, shoulder distance apart.

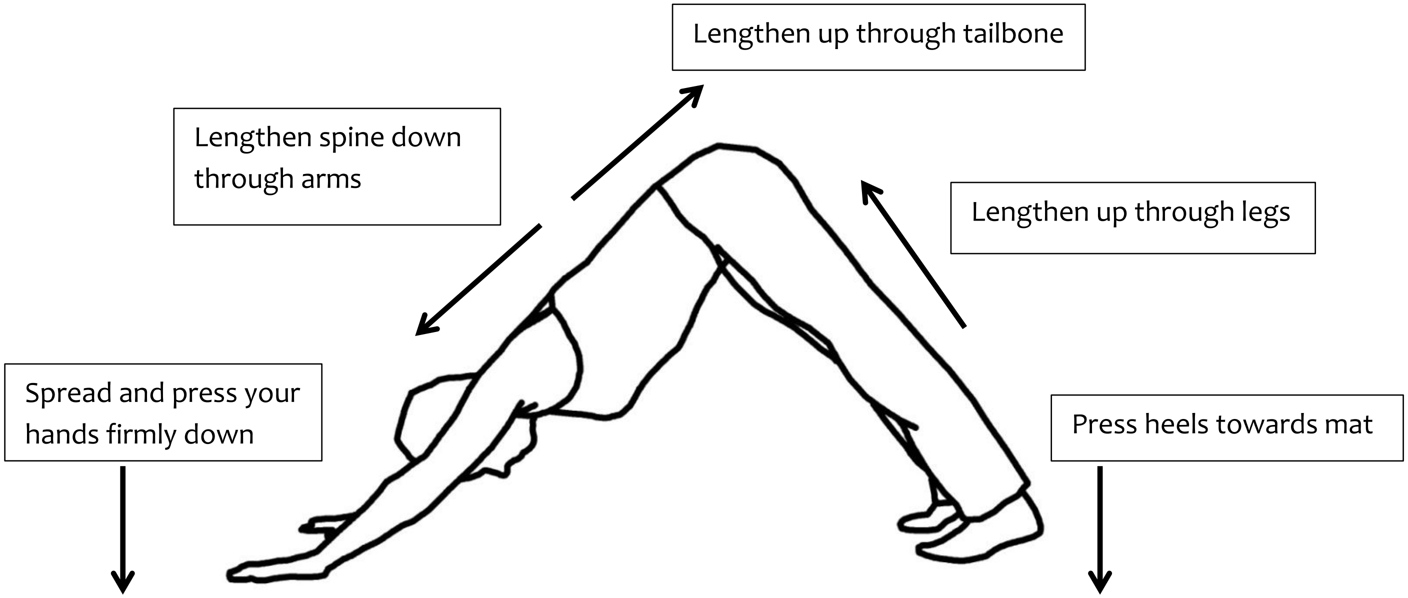

Extended Leg Pose

Instructions for Yoga Pose

1. Lying on your back with both knees bent, loop belt around the bottom of your right foot while holding both ends of the belt with your right hand.
2. Straighten your leg as much as possible.
3. Press your right foot up into the belt while gently pulling your arms down towards the floor.
4. Move the right leg out to the right side and let your left knee move to the left. Then bring your leg back straight.
5. Repeat, moving your leg out and up three times.
6. Bend your knee and take the belt off.
7. Repeat on the left leg.

Other Options for Pose

1. Bend and straighten your leg, without moving leg to the side, several times using a belt for support.
2. Lie on your back with your feet facing a wall or a chair with your knees bent, place one leg up on the wall or on the seat of a chair as straight as possible using the wall or chair for support.
3. To do the pose in a chair: Sit on a chair, with legs bent and feet on floor, place one leg straight up onto block or another chair seat.

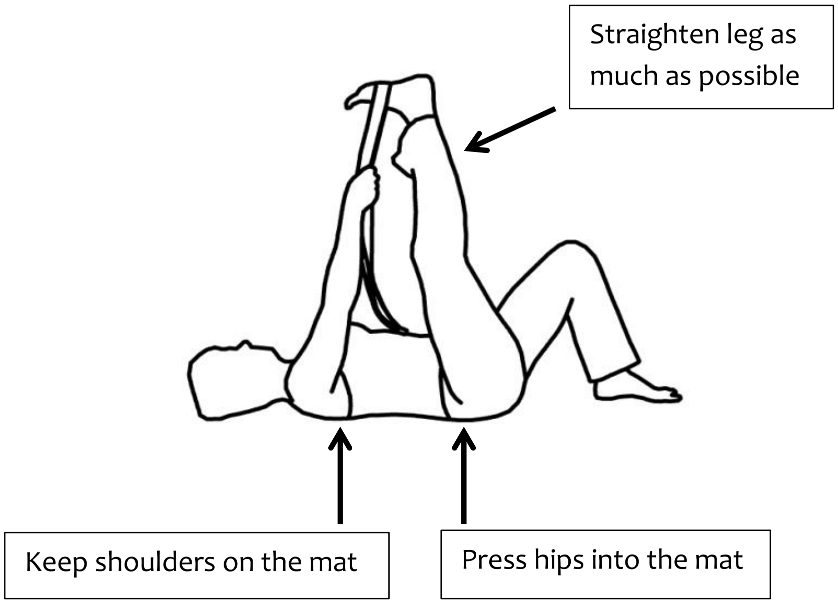

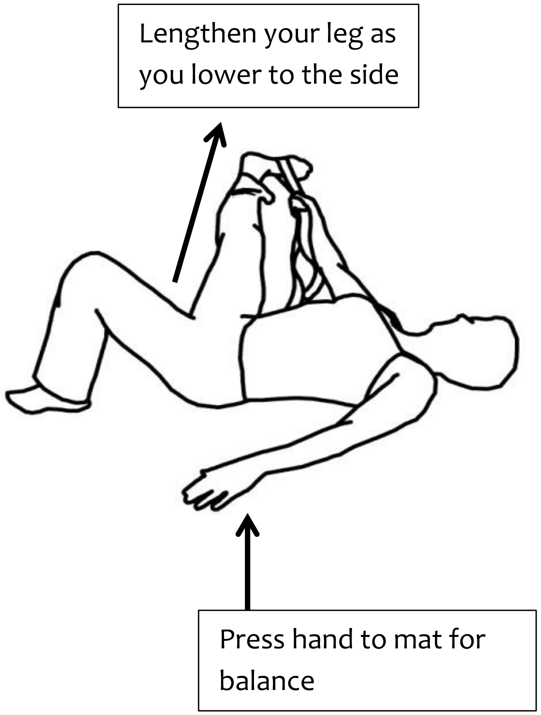

© Boston Medical Center

Knees to Chest Pose

Instructions for Yoga Pose

1. Lie on your back with your knees bent.
2. Lift one knee up to your chest while holding on to that leg around your shin or thigh.
3. Press your back into the ground.
4. Repeat with the other knee.
5. Repeat bringing both knees to your chest.

Other Options for Pose

1. Use a belt around your shin or thigh if you cannot reach them with your hands.
2. If you cannot bring your knees to chest with your hands or a belt, place feet on a wall or a chair.
3. To do the pose in a chair: Sitting in chair, raise one knee at a time, holding your leg up with your hands or a belt under thigh.

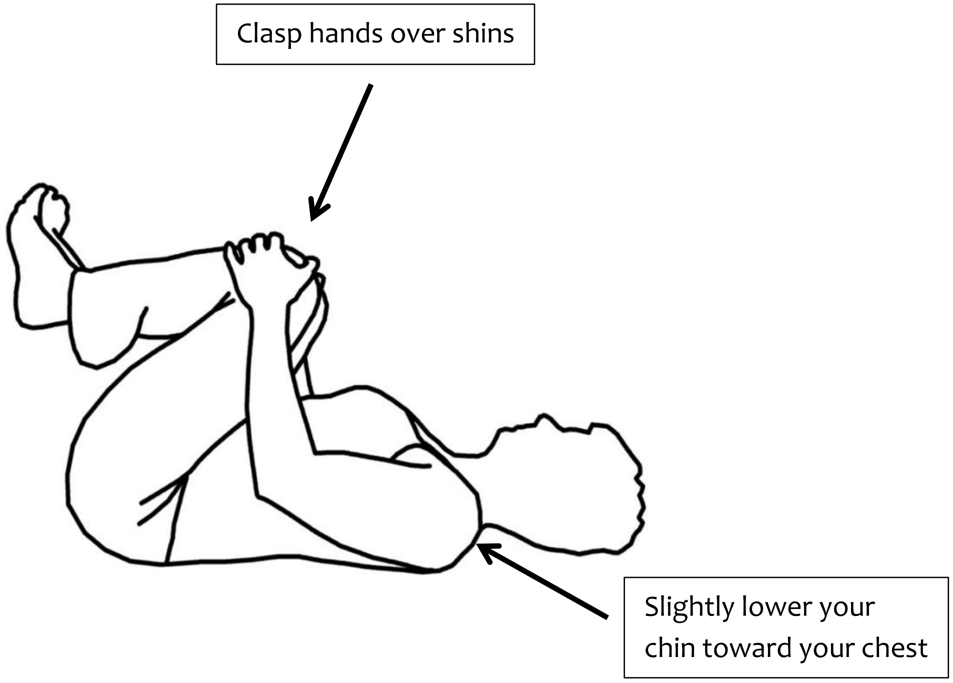

© Boston Medical Center

Knees Together Twist Pose

Instructions for Yoga Pose

For Warm-Up

1. Lie on your back
2. Bring both knees to your chest and then lower them towards the floor on the left, keeping your right shoulder on the mat.
3. Bring your knees back to the middle. Move your knees down towards the floor on the right, keeping your left shoulder on the mat.
4. Repeat three times moving between the left and right sides.

For Cool-Down

1. Lie on your back
2. Bring both knees to your chest and then lower them towards the floor on the left, keeping your right shoulder on the mat. Hold the pose.
3. Bring your knees back to the middle. Move your knees down towards the floor on the right, keeping your left shoulder on the mat. Hold the pose.
4. Come back to center.

Other Options for Pose

1. If your shoulder cannot stay down on the floor, place a blanket or block under your knees on each side so they do not go as far to the floor.
2. To do the pose in a chair: use the instructions for Chair Twist Pose: Seated (p27).

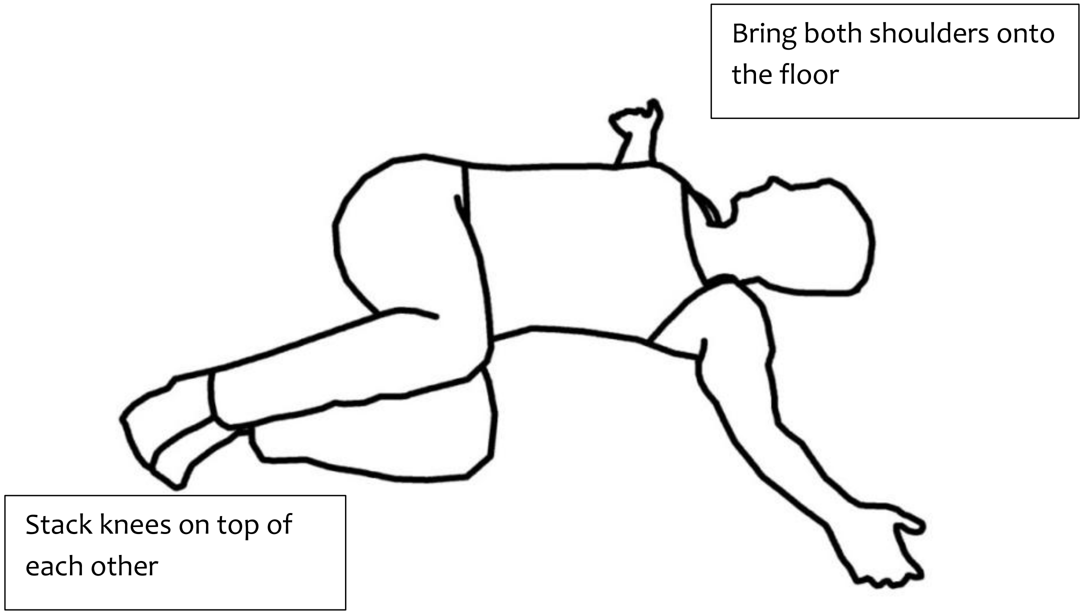

© Boston Medical Center

Locust Pose

Instructions for Yoga Pose

1. Lie on your stomach with your chin or forehead on the mat and your hands straight behind you palms facing up and your toes pointed away from you.
2. Lift your head, upper chest, arms and legs off the mat at the same time.
3. Release and lower your body back to the mat.

For the first few weeks use option 1 below.

To prevent injury, make sure to look forward or slightly down. Looking up to the ceiling can cause neck injury if the head bends too far backwards.

Other Options for Yoga Pose

1. Fold your arms in front of you and place head on back of hands. Raise one leg at a time. Then raise both legs together. Lower your legs and then with arms down by the sides of body and palms facing up, raise head, chest and arms, while pressing legs down.
2. Stand facing a chair: Bend forward and hold onto the seat of a chair. Extend one leg back , inhale, slowly raise the leg straight up off the floor. Exhale, bring it down. Repeat on the other side
3. To do the pose in a chair: Sitting in a chair, place feet firmly on floor with the arms by the sides and allow the shoulders to drop down away from the ears. Roll the shoulders back to open the chest.

Keep back of the neck long

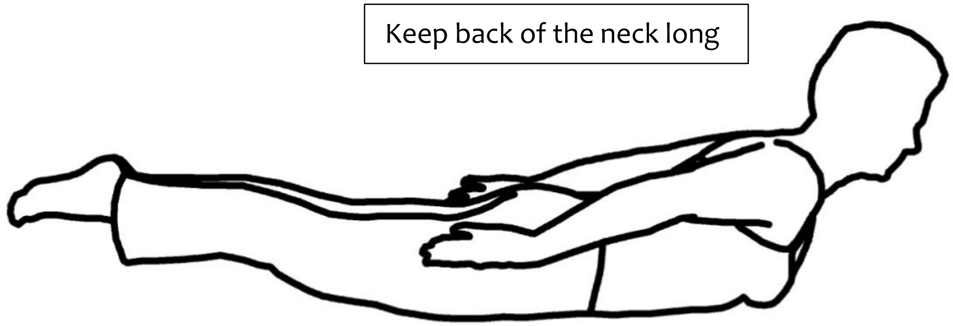

Rest on belly, ribs, and pelvis

© Boston Medical Center

Modified Chair Pose

Instructions for Yoga Pose

1. From Mountain Pose (p39), place your hands on your hips, and bend your knees as if you are about to sit in a chair.
2. Move your weight back onto your heels and bring your stomach in, while raising your arms out in front of you at shoulder level.
3. Straighten your legs and bring your arms back down.

Other Options for Pose

1. Keep your hands on your hips.
2. Stand with your back on the wall. Bend your knees and press your lower back into the wall. Slowly raise arms up over head.
3. To do the pose in a chair: Sit on the edge of the chair with your feet directly below your knees. Lean slightly forward, allowing yourself to bear some of your weight onto your feet. Raise your arms over head, keeping your chest lifted and back straight.

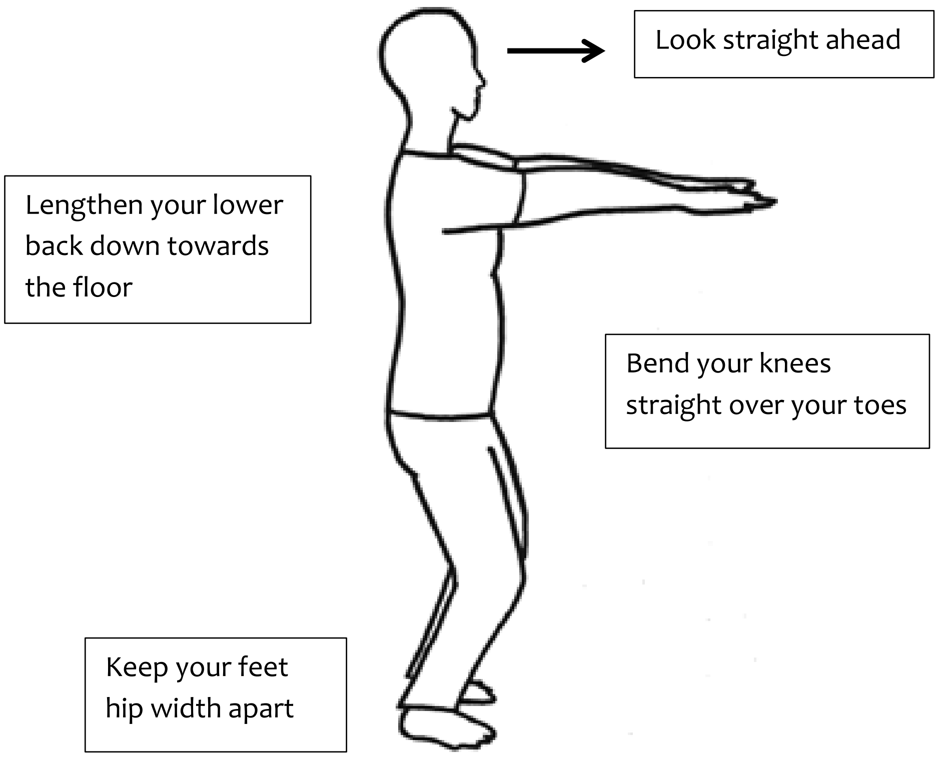

© Boston Medical Center

Mountain Pose

Instructions for Yoga Pose

1. Stand straight with your feet facing forward and hip distance apart. Let your weight bear equally on all parts of your feet.
2. Allow your back to stretch and lengthen while looking forward. Keep your head over your shoulders, your shoulders over your hips and your hips over your ankles.

Other Options for Pose

1. Stand with back against wall
2. Lie on floor with legs straight and feet pressing into wall. Arms down by sides, shoulders pressing down toward the floor.
3. To do the pose in a chair: Sit upright in a chair with back straight. With your hands on your hips, move your hips slightly forward and back to find the center. Keep your back straight so that your head and shoulders are in line with your hips. Move your arms straight down by the side of your body. Look straight ahead.

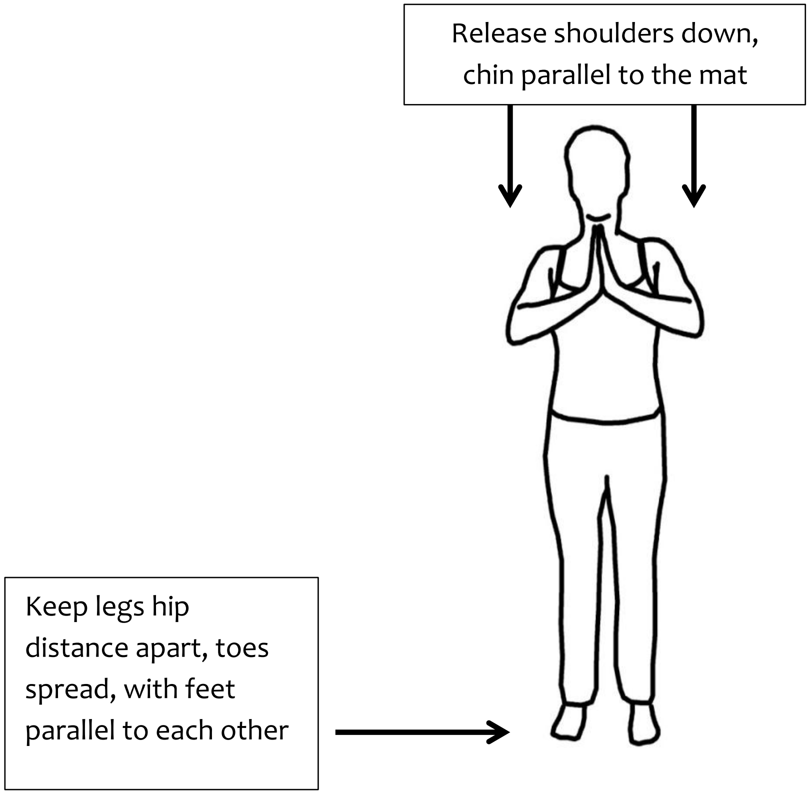

© Boston Medical Center

Pelvic Tilt Pose

<u>Instructions for Yoga Pose</u>

1. Lie on your back with your knees bent and the bottoms of your feet on the mat.
2. Extend your arms down towards your feet with your palms facing down.
3. Gently press your lower back into the mat.
4. Relax your back and then repeat several times.

<u>Other Options for the Pose</u>

1. Use a block between the knees to keep them closer together if your knees are moving out wider than hip distance.
2. Stand with back and head against wall, feet away from wall and slightly bent. Gently press your lower back into the wall.
3. To do the pose in a chair: Sit up straight in a chair with feet firmly planted on the floor, place a block length wise behind your back and gently press your lower back into the block. Breathe and sit back upright. Repeat several times.

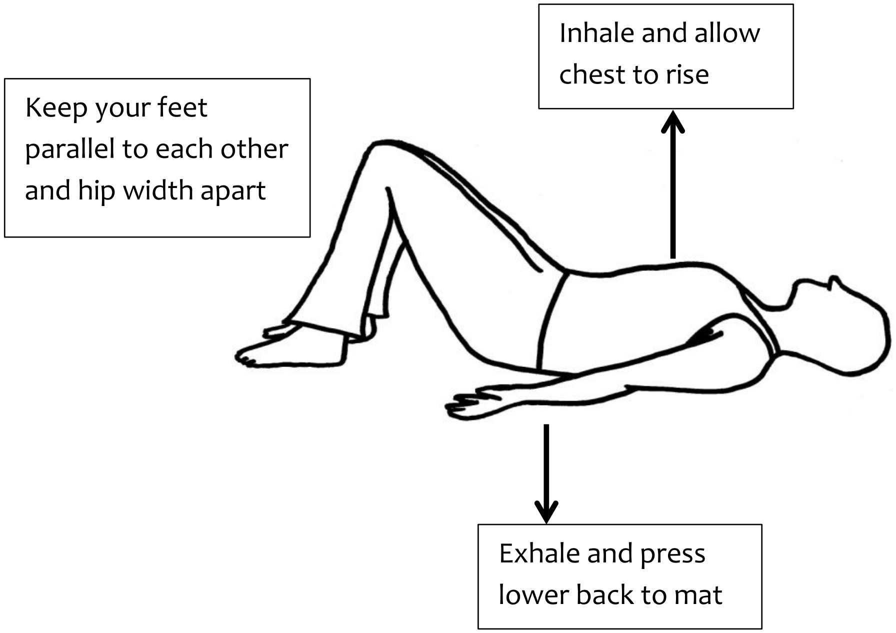

At Wall

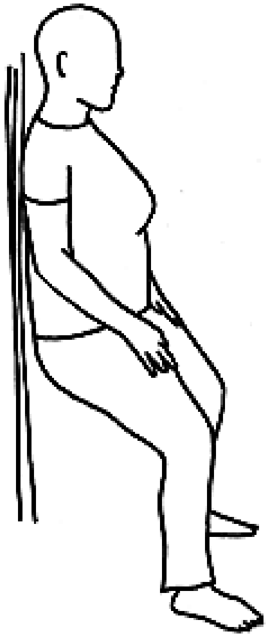

© Boston Medical Center

Reclined Chest Opener Pose

Instructions for Yoga Pose

1. Place a folded blanket horizontally across the mat. Lie down on your back with the blanket under your upper back.
2. If your neck is uncomfortable, place a blanket under your head.

Modifications:

1. For more of a chest opening use two blankets or a block. If you have lower back discomfort, bend your knees or come out of the pose.

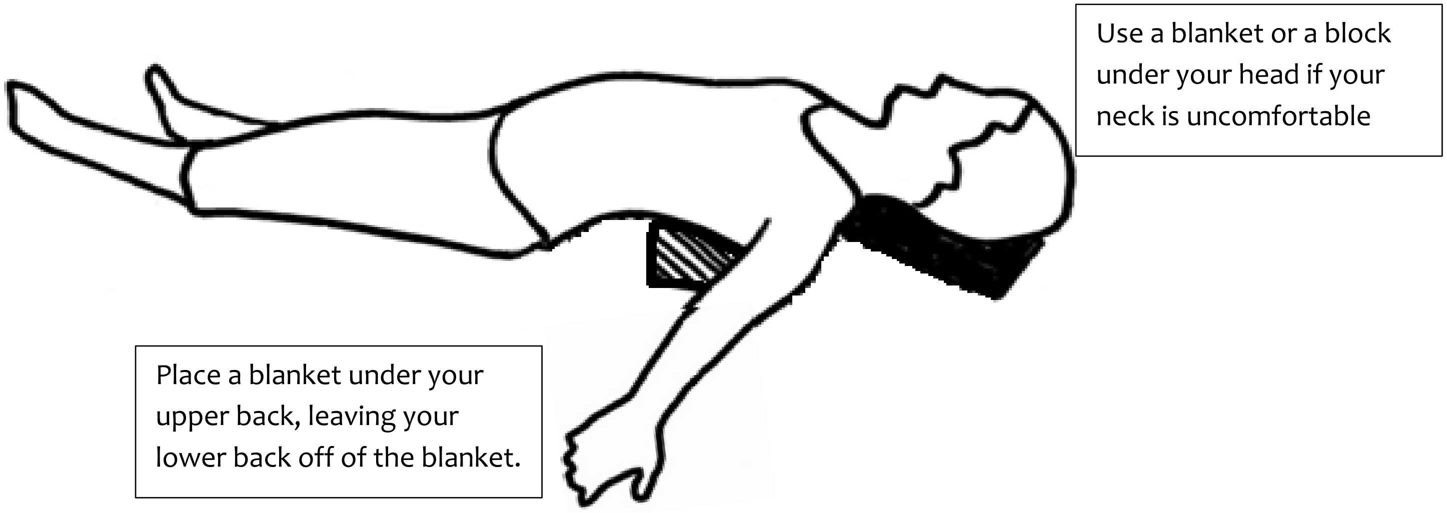

© Boston Medical Center

Reclining Cobbler Pose

Instructions for Yoga Pose

1. Lie on your back with your knees bent. Bring the bottoms of your feet together so your knees can open to the sides.
2. Place folded blankets or bolsters to either side of your thighs. Gently lower your knees to either side with the bottoms of your feet still touching.
3. Relax your shoulders and place your arms to the sides, palms up.

Other Options for Pose

1. If your lower back is uncomfortable, fold 1-2 blankets lengthwise to support length of spine from waist to head. Don't have the blankets right up to the tailbone, but rather have space from waist to tailbone.
2. If your back is uncomfortable after coming out of posture, lie flat on the floor and hug both knees to the chest, gently rocking side to side.

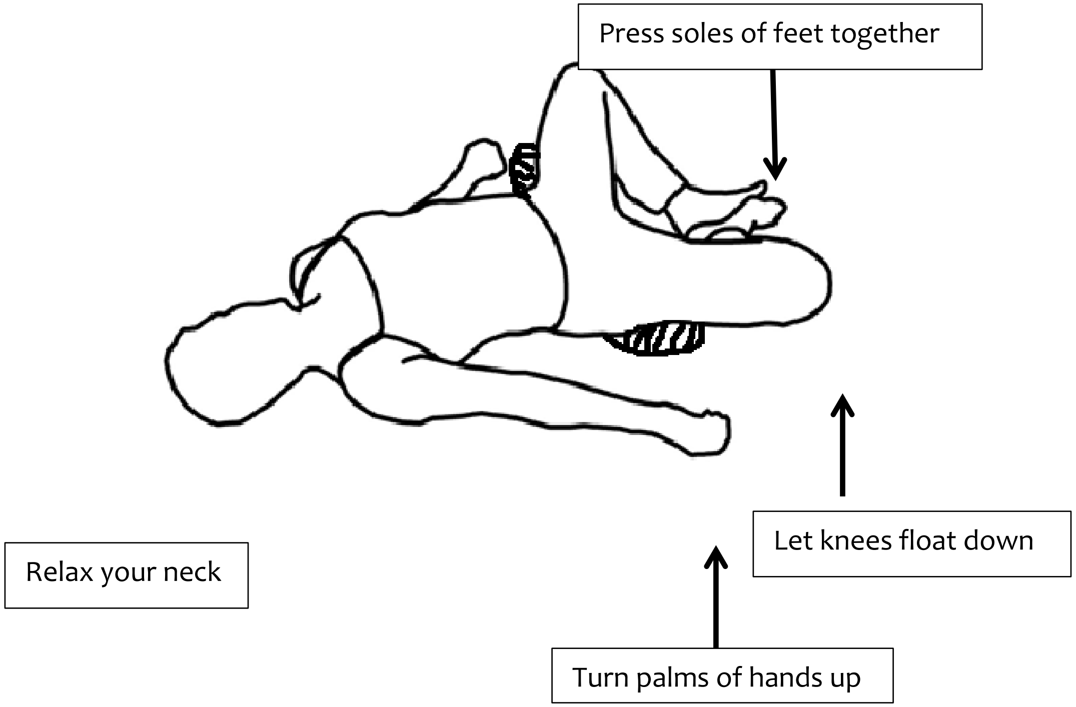

© Boston Medical Center

Svasana

<u>Instructions for Yoga Pose</u>

1. Lie on your back with your legs slightly wider than hip width and your arms slightly away from your body, palms facing up.
2. Relax your whole body including your face and let your body be heavy.
3. Relax in this pose at the end your practice for at least 5 minutes.

<u>Other options for pose</u>

1. Cover yourself with a blanket.
2. If your back is uncomfortable, place bolster or blanket under knees.
3. If your back is uncomfortable, place lower legs on a chair seat
4. Side relaxation: Lie on your side with a blanket under head for support, another blanket under your top arm and another supporting between knees.
5. Belly Relaxation: Lie on your belly, with your feet slightly turned in toward each other and with your hands or a blanket folded under your head.
6. To do this pose in a chair: Sit on a chair with your spine supported with a blanket or a bolster behind your back and a blanket over your legs.

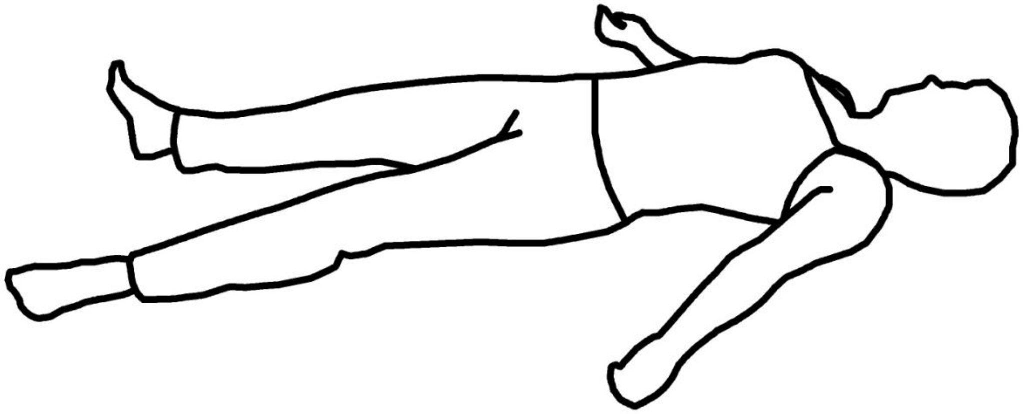

© Boston Medical Center

Shoulder Opener Pose

Instructions for Yoga Pose

1. From Mountain Pose (p39), place your hands behind your back and clasp your hands together.
2. Slowly raise your arms so there is a comfortable stretch. Do not bend forward.
3. Breathe and move your shoulders back towards each other.
4. Slowly lower your arms down and release your hands.

Other Options for Pose

1. If your hands cannot reach one another, use a belt between your hands.
2. To do the pose in a chair: Sit in a chair and clasp your hands behind your back. Slowly lift your arms and then lower them, releasing your hands.

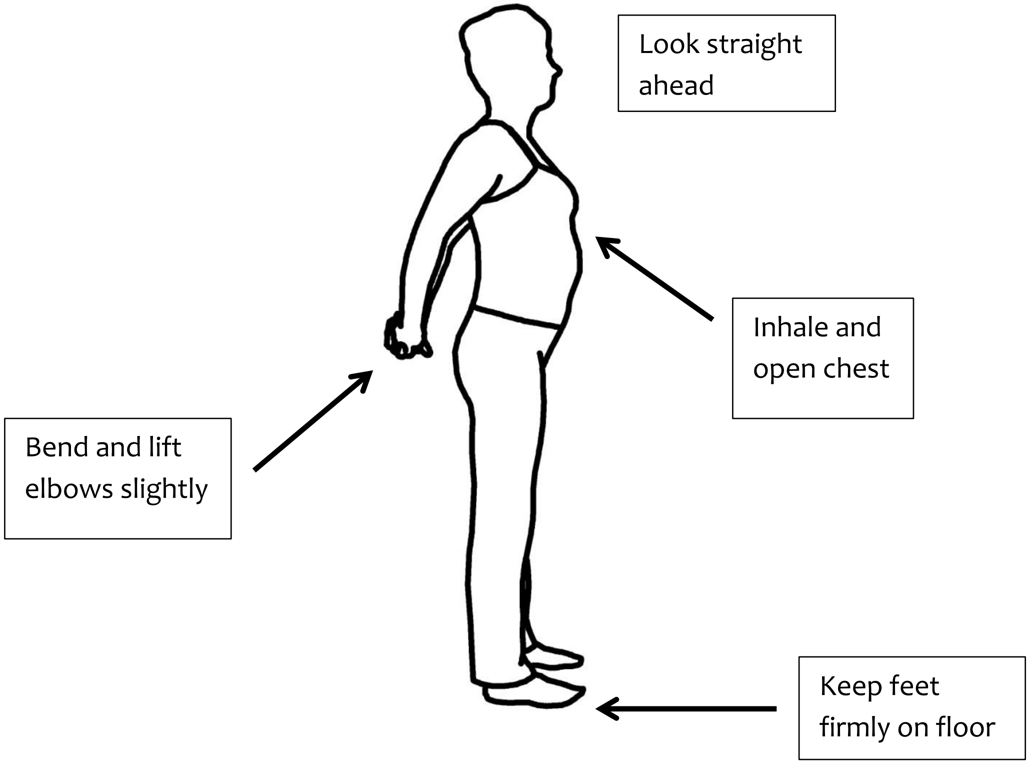

© Boston Medical Center

Sphinx Pose

Instructions for Yoga Pose

1. Lie on your stomach with your chin or forehead on the mat and your elbows under your shoulders, close to your body.
2. Point your legs behind you with your toes on the mat.
3. Press your forearms into the mat and lift your head and chest up.

To prevent injury, make sure to look forward or slightly down. Looking up to the ceiling *can* cause neck injury if the head bends too far backwards.

Other Options for Pose

1. To do the pose in a chair: Sit in a chair with feet firmly on the floor. With your hands on your thighs, allow shoulders to drop down away from your ears. Roll shoulders back to open chest. Move shoulder blades slightly towards each other and pull abdominal muscles in. Expand chest. Look forward.

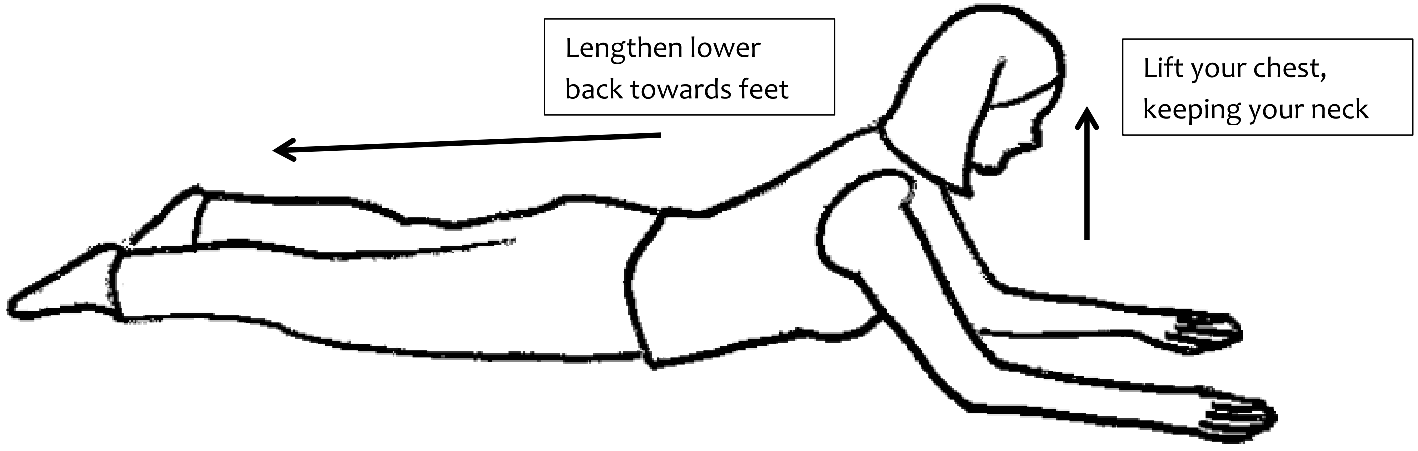

Spinal Rock Pose

Instructions for Yoga Pose

1. Lie on your back and bend your knees into your chest with your arms on the side of your body.
2. Gently swing your legs towards your head and then back to the floor.
3. Gently swing legs back and forth, rocking the lower back, lifting your hips off the ground if possible.
4. Repeat rocking motion several times, and then rest.

Other Options for Pose

1. Place an opened blanket down the length of the mat to cushion spine.
2. Lying on your back with knees bent, place feet on a wall or edge of a chair seat and lift one leg or both legs at a time, gently swinging the leg(s).
3. To do the pose in a chair: Sit in a chair, place feet under knees with knees hip width apart. Exhale, gently round spine and tuck chin toward chest. Inhale, straighten up. Repeat several times. Rest, sitting straight up.
4. If hips are lifting easily, being to roll up to sitting as the legs come back to the floor, keeping the chin tucked. If possible, roll back down, still keeping your chin tucked into your neck.

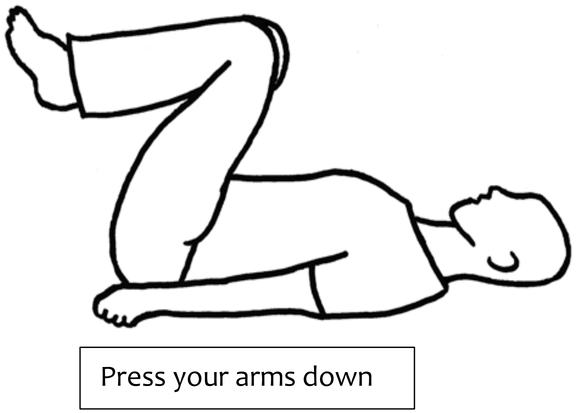

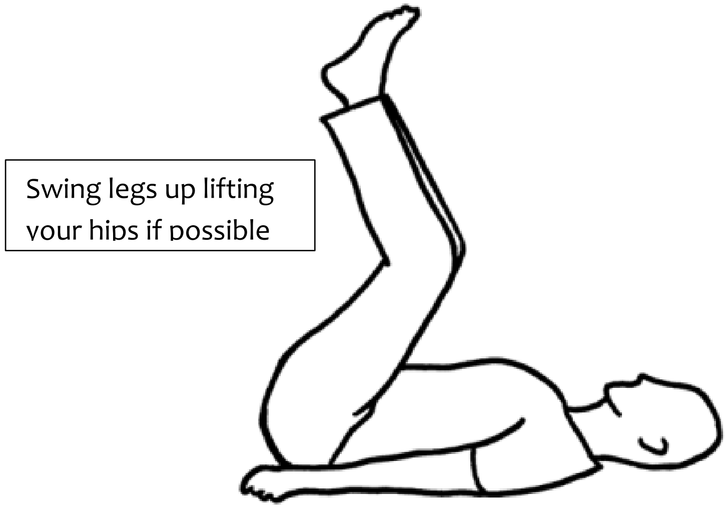

Standing Forward Bend at Wall Pose

Instructions for Yoga Pose

1. Start in Wall Dog (p52). Step your right leg forward towards the wall while stepping your left foot back away from the wall.
2. Lower your back so that it is as parallel to the floor as possible.

Other Options for Yoga Pose

1. Place your hands on a chair or table.
2. To do the pose in a chair: Sit in a chair and extend one leg straight forward with the heel on the floor and the other leg bent with the foot on the floor. Exhale, bend forward at the hips until there is a feeling of a stretch in the back of the leg. Inhale sit back up. Repeat and hold for 3 breaths. Repeat on other side.

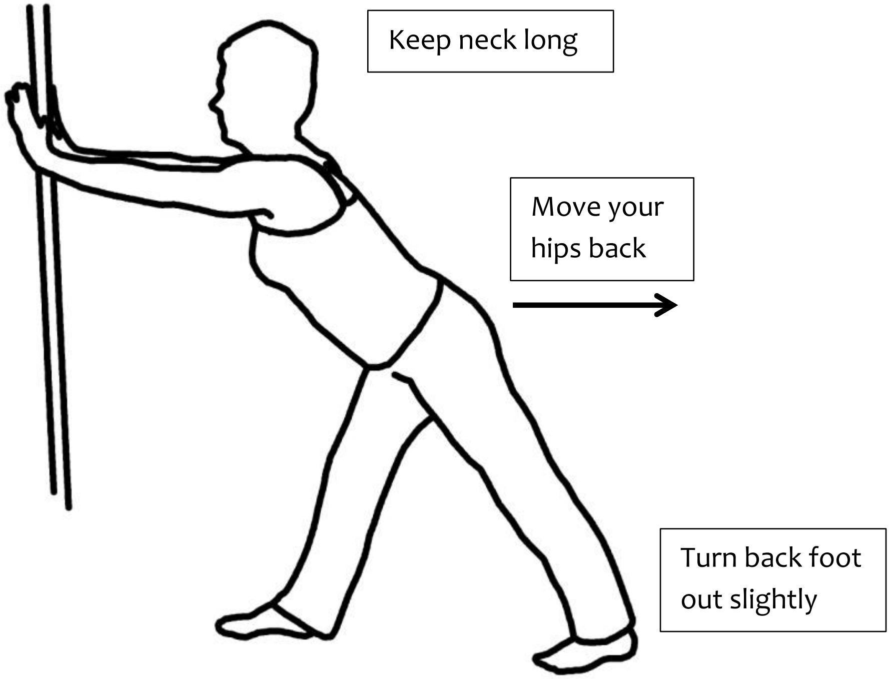

© Boston Medical Center

Supported Bridge

Instructions for Yoga Pose

1. Lie on your back with your knees bent and your feet as close to your hips as possible. Keep your knees, and feet parallel.
2. Lift your hips and place a block under your tailbone for support.
3. Keeping your hips rested on the block, lift your chest without lifting your head or shoulders.
4. Relax your arms on the floor, slightly out to the side.

Other Options for Pose

1. If there is pain in your knees, walk feet further away from hips.
2. To do the pose in a chair: Sit with a blanket or a bolster behind your back.

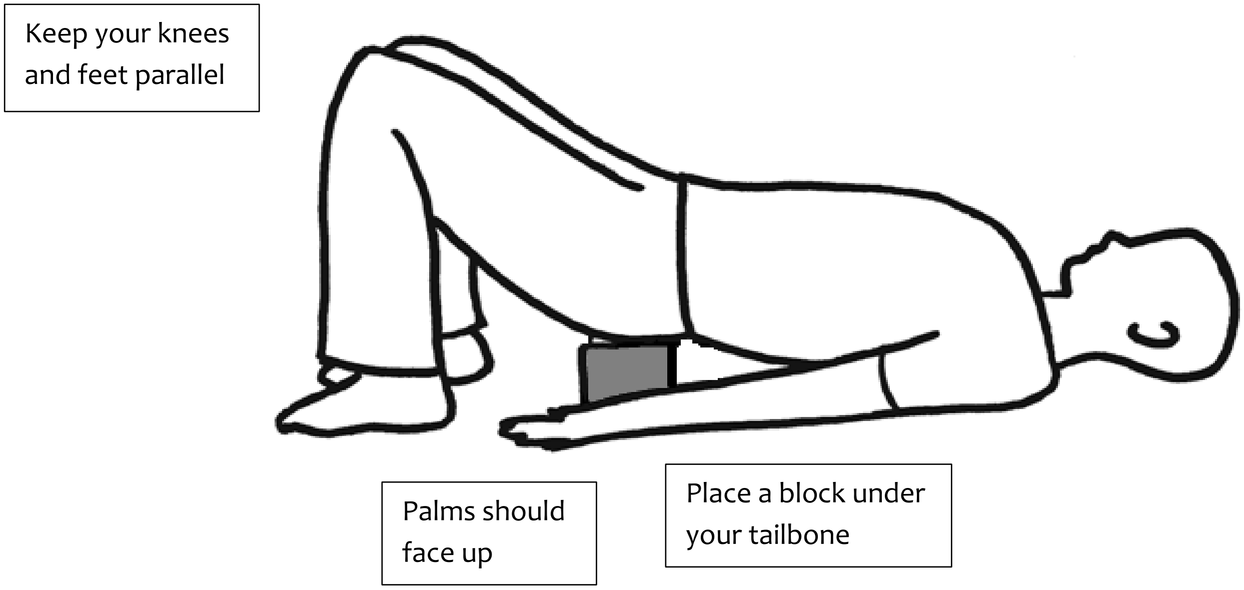

© Boston Medical Center

Table Top with Leg Extended Pose

Instructions for Yoga Pose

1. Start on your hands and knees with your back flat and level like a table. Move your right leg behind you with your leg straight and your toes on the floor.
2. Bring your leg back so your knee is on the mat.
3. Repeat of the other side, stretching your left leg behind you.

To prevent injury, make sure to look forward or slightly down. Looking up to the ceiling can cause neck injury if the head bends too far backwards.

Other Options for Pose:

1. If your wrist is uncomfortable, make a fist with both hands resting your weight on your knuckles with thumbs facing down.
2. If your knees or hands are uncomfortable, you can roll the sides of the mat under your knees or the top of the mat under your hands.
3. Rest your forearms on the mat instead of your hands.
4. Standing with hands on the back of a chair (bending forward) or against the wall, extend one leg at a time back with your toes down on the floor.
5. To do the pose in a chair: Sit on the edge of a chair, extend one leg out in front with the heel on the floor. Press down through the heel.

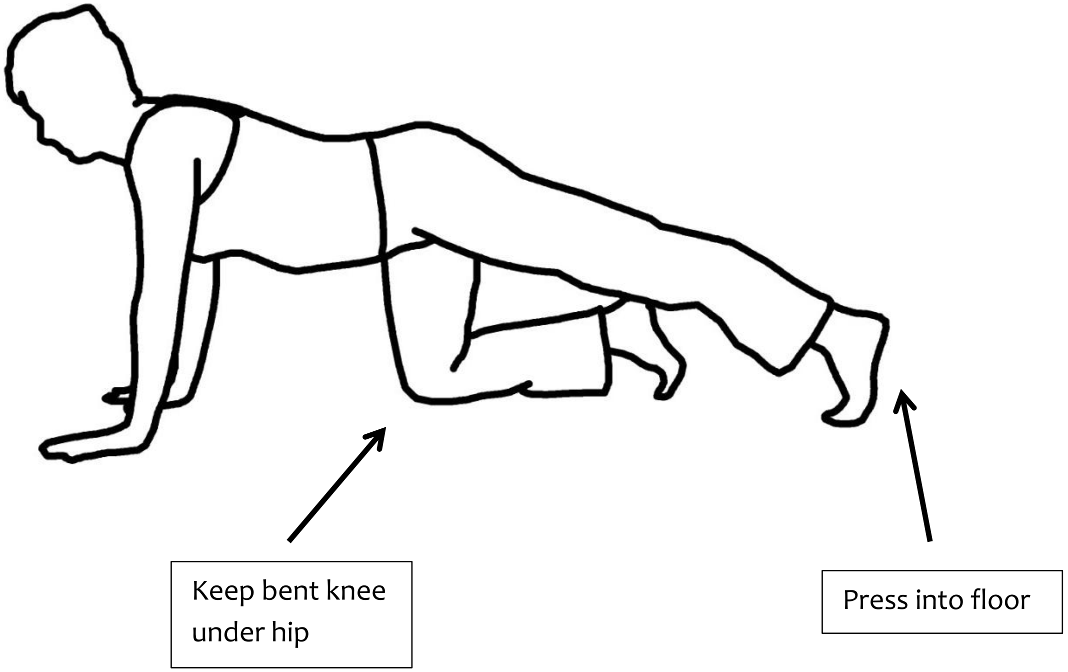

Triangle Pose

Instructions for Yoga Pose

1. Start in Mountain Pose (p39) and stretch your arms out at shoulder height with your feet facing forward away from your body.
2. Spread your feet so that your ankles are under your wrists.
3. Turn your right foot to the right and your left foot slightly to the right.
4. Bend at the right hip and place the right hand on the right leg as you move the left arm straight up.
5. Keep your legs, arms, and back straight.
6. Inhale and bring your arms and body back to center. Turn and repeat on the other side.

Other Options for Pose

1. Triangle at wall (p50)
2. Place right hand on a chair seat when bending to the right, and vice versa on left.
3. To do the pose in a chair: Sitting in a chair, extend right leg out to side turning the whole leg out 90 degrees with the toes pointing to the side wall. Your left leg should be bent with the foot on the floor. Bend to the right side at the hips, bending directly over your extended leg and place the right hand on the leg. Repeat on the other side.

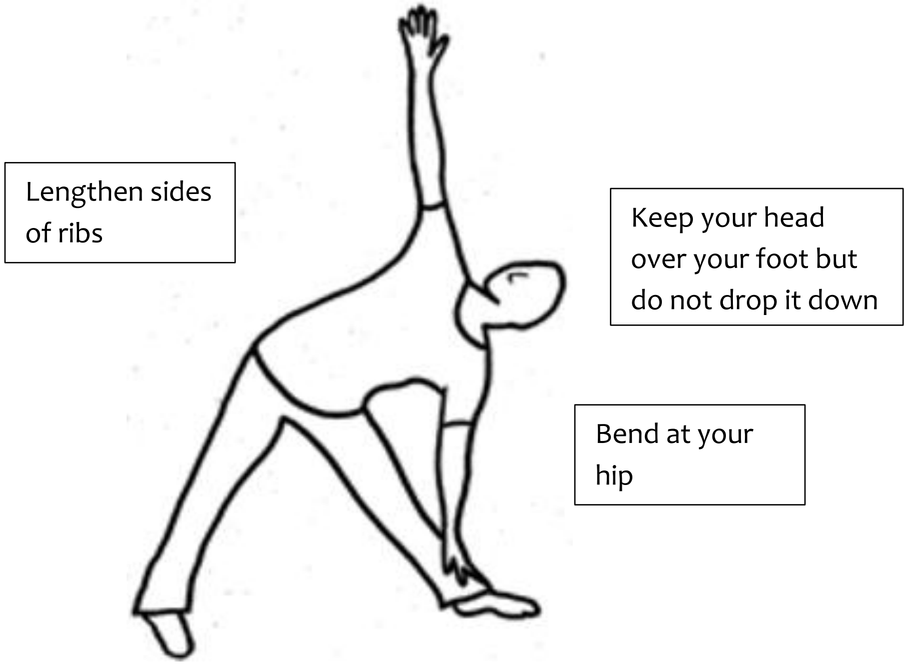

© Boston Medical Center

Triangle at Wall Pose

<u>Instructions for Yoga Pose</u>

1. Stand with the left side of your body facing the wall and your left hand on the wall.
2. Turn your left leg towards the wall with your toes close to or touching the wall.
3. Turn your right foot slightly in towards the wall.
4. Bend at your left hip towards the wall and slide your left hand up the wall.
5. Turn around and repeat on the other side.

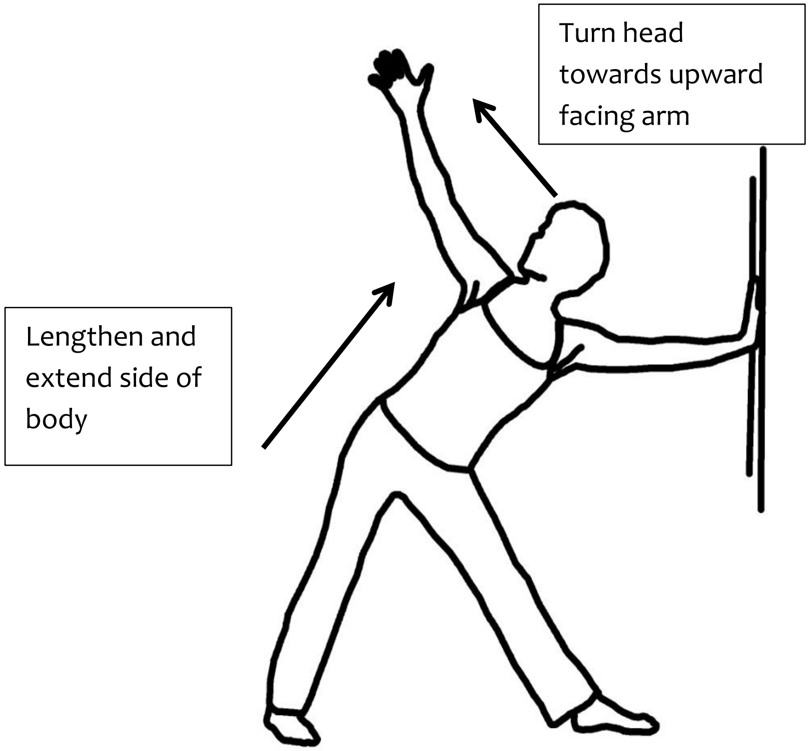

© Boston Medical Center

Wall Dog Pose

Instructions for Yoga Pose

1. Start facing the wall with your feet hip width apart.
2. Bend forward from your hips and place your hands on the wall. Move your hands up the wall so they are slightly higher than your shoulders.
3. Gently press your hands into the wall while keeping your arms and legs straight. Step back so your legs are angled back from your hips.
4. Bring your head between your arms and look down.

Other Options for Pose

1. To do the pose in a chair: Sit on edge of a chair, extend both of your legs out, hips weigh apart and raise both arms straight up over head, shoulder distance apart.

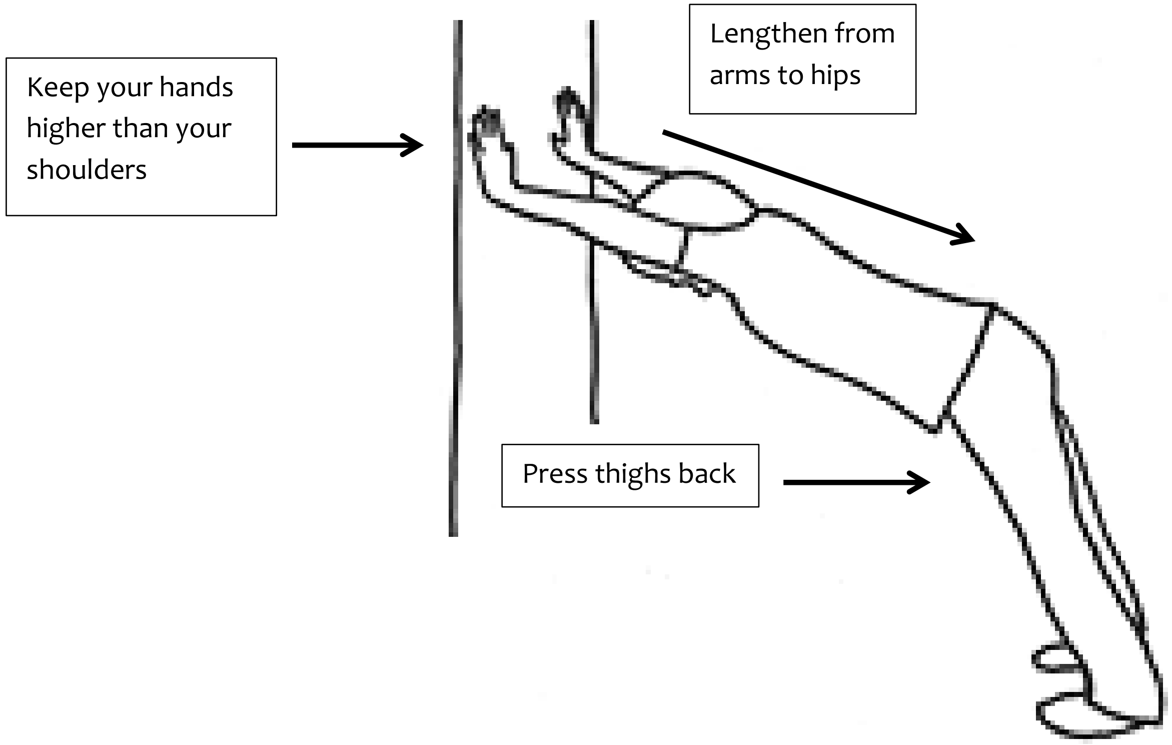

© Boston Medical Center

Warrior I Pose

Instructions for Yoga Pose

1. Start in Mountain Pose (p39) and step right foot back with your toes pointing forward.
2. Stretch your arms out to the sides at shoulder height with your palms facing down.
3. Turn your right heel inward while keeping the right leg straight.
4. Bend your left leg so your knee is over your left ankle. Keep your right leg straight.
5. Raise your arms straight up over your head next to your ears.
6. Bring your arms down and step back to a standing position. Repeat on the other side.

Other Options for Pose

1. Move your back foot slightly to the side, away from the center of the body to make a wider stance if you feel unsteady.
2. Place hands on a chair in front of you to steady yourself.
3. Warrior I at Wall (p54).
4. To do the pose in a chair: Using a chair without arms, sit sideways on the chair with your right leg bent so that your knee is over your ankle and your left leg is extended behind you. Facing sideways on the chair, raise your arms up over head and lift chest. If raising the arms up cause back pain, keep your hands on the hips.

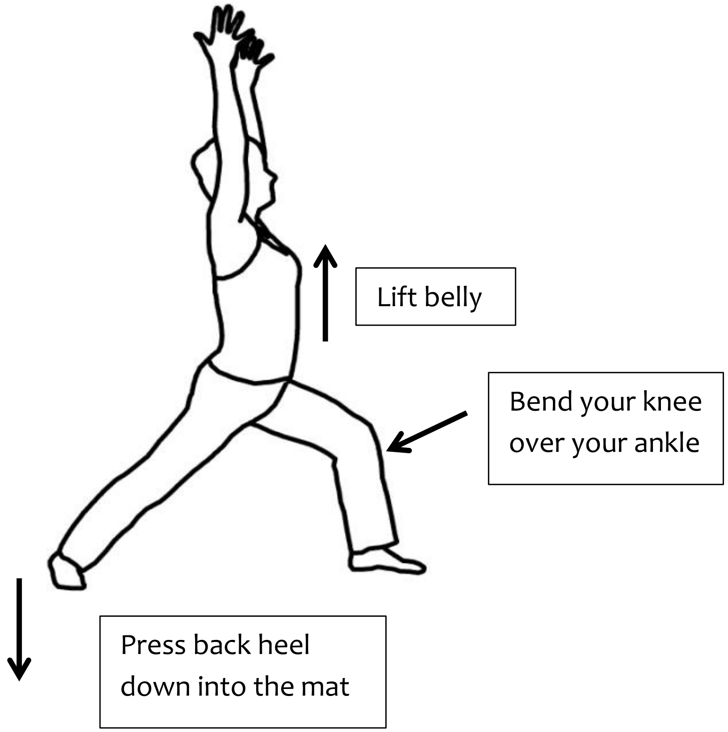

© Boston Medical Center

Warrior I at Wall Pose

<u>Instructions for Yoga Pose</u>

1. Start facing the wall with your hands on the wall at shoulder height, arms straight. Step your right foot up to the wall and your left leg back as far as possible, bend your right knee over your ankle.
2. Turn your left foot out slightly so there is a stretch in the back of your leg.
3. Straighten and move your right leg back. Repeat on the other side.

<u>Other Options for Pose</u>

1. Allow the back of the heel to come off floor
2. Place a block between knee and wall pushing knee into block.
3. Lean into wall, placing head on wall
5. To do the pose in a chair: Using a chair without arms, sit sideways on the chair with your right leg bent so that your knee is you’re your ankle and your left leg is extended behind you. Facing sideways on the chair, raise your arms up over head and lift chest. If the arms up cause back pain, keep the arms on the hips.

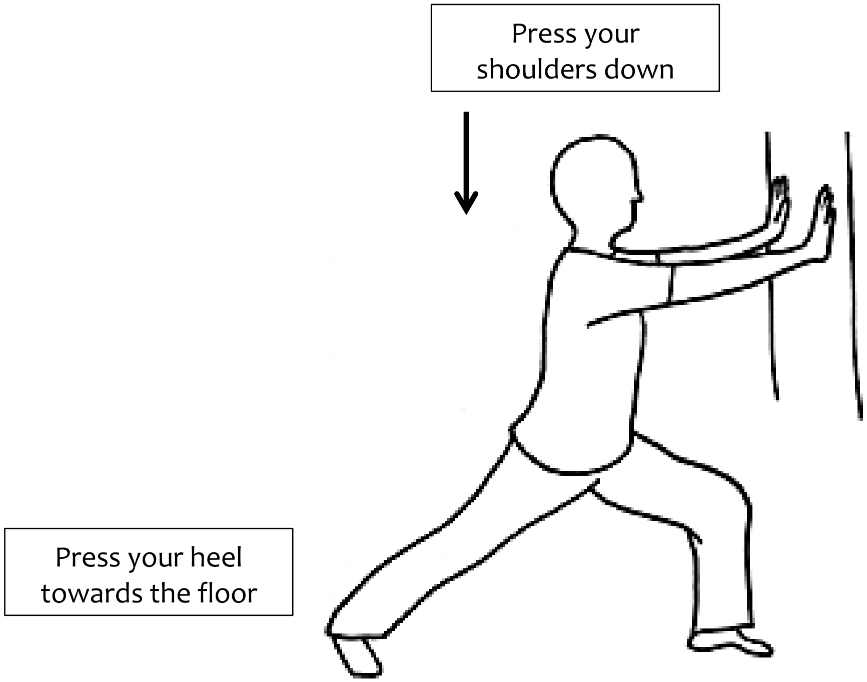

© Boston Medical Center
