# Supplementary material for: Yoga vs. physical therapy vs. education for chronic low back pain in predominantly minority populations: study protocol for a randomized controlled trial
Source: Trials. 2014 Feb 26;15:67. doi: 10.1186/1745-6215-15-67 (PMC3944007; doi:10.1186/1745-6215-15-67)
Supplement: Additional file 2 — Yoga Teacher Training Manual. A training and reference manual that includes a description of the yoga protocol, guidelines for teaching the exercises, and details of each yoga posture with associated modifications as necessary. [file 1745-6215-15-67-S2.pdf]

Yoga Teacher Training Manual

Back to Health

A Study Comparing Yoga, Physical Therapy & Education for Chronic Low Back Pain

Table of Contents for Yoga Teacher Training Manual

Introduction from PI	3
Low Back Pain	5
Yoga for Low Back Pain	7
Yoga Teacher Roles, Responsibilities and Logistics	14
Teacher Roles and Responsibilities	15
Teacher Assistant Roles and Responsibilities	16
Boston Medical Center Yoga for Chronic Low Back Pain Intervention	18
Back to Health: An Overview	19
Segments, Themes and Objectives	20
Individual Class Format	22
Segment 1: Opening to Something Greater	
Week 1	26
Week 2	28
Week 3	31
Segment 2: Listening to the Body	
Week 4	32
Week 5	34
Week 6	36
Segment 3: Engaging Your Power	
Week 7	38
Week 8	40
Week 9	42
Segment 4: Bringing it Home	
Week 10	44
Week 11	46
Week 12	48
Guidelines for Teaching Specific Yoga Poses	50
Baby Dancer Pose	51
Bridge Pose	52
Cat/Cow Pose	53
Chair Pose	54
Chair Twist Pose: Seated	55
Chair Twist Pose: Standing	56
Child's Pose	57
Cobra Pose	58
Crescent Moon Pose	59
Downward Dog Pose	60
Extended Leg Pose	61
Flat Back Forward Bend	62
Forward Bend Pose	63
Knees to Chest Pose	64
Knees Together Twist	65
Locust Pose	66

Modified Chair Pose 67
Mountain Pose 68
Mountain Pose with One Leg Lifted 69
Pelvic Tilt Pose 70
Reclined Chest Opener Pose 71
Reclining Cobbler Pose 72
Shoulder Opener Pose 73
Sphinx Pose 74
Standing Forward Bend with Wall Assist Pose 75
Sun Salutations 76
Supported Bridge Pose 77
Table Top with Leg Extended Pose 78
Triangle Pose 79
Toe Taps 80
Triangle at Wall Pose 81
Wall Dog Pose 82
Warrior I Pose 83
Warrior I at Wall Pose 84
Guidelines for Teaching Relaxation Exercises 85
Preparing the Body for Relaxation 86
Svasana 87
Suggestions for Relaxation Exercises 88
Guideline for Teaching Yoga Breathing Exercises by Week 91
Weeks 1-2 92
Weeks 3-5 93
Weeks 6-9 94
Weeks 10-12 95
Poems By Week 96
Week 1 97
Week 2 98
Week 3 99
Week 4 100
Week 5 101
Week 6 102
Week 7 103
Week 8 104
Week 9 105
Week 10 106
Week 11 107
Week 12 108
Bibliography 111
Appendix I: Supplementary Readings and Poems 114
Appendix II: Standardized Hatha Yoga Protocol 117
Acknowledgments 118

© Boston Medical Center
Program for Integrative Medicine & Health Care Disparities
Back to Health Trial

Introduction from Principal Investigator

Welcome to the Back to Health Study! Back to Health is an NIH-funded randomized controlled trial (RCT) that compares the effectiveness of three different treatments for chronic low back pain (CLBP): yoga, physical therapy, and education. CLBP affects 5-10% of U.S. adults annually and costs over $50 billion per year in direct health care expenditures. Individuals from low-income, minority backgrounds are disproportionately impacted by CLBP due to disparities in access and treatment. Several recent studies suggest yoga as an effective treatment for CLBP. Yoga may also have other relevant benefits for CLBP patients, such as improved mood, stress reduction and lower health care costs. Multiple CLBP studies also support a moderate benefit for exercise therapy individually-delivered by a physical therapist, a reimbursed and well-established treatment to which physicians refer 22-38% of their low back pain patients. Education about self-care for back pain has also been tested in studies and has been found to be helpful. Education can be in various forms, such as provider counseling, written materials and support. However, no studies have directly compared yoga, physical therapy and education for the treatment of CLBP in any population—low-income, minority, or otherwise. To ultimately reduce disparities in CLBP for minority populations, patients, providers, and health insurers need to know how well-established treatments such as physical therapy (PT) compare to complementary therapies such as yoga and commonly used self-care approaches such as education. The Back to Health Study was designed to address this important question. Back to Health is a comparative effectiveness randomized controlled trial (RCT) for people from predominantly low-income minority backgrounds with CLBP. We will compare three treatment groups:

1. A standardized exercise clinical evidence-based therapy protocol individually delivered by a physical therapist
2. A standardized 12-week yoga protocol, delivered in a class format
3. An education program that includes a comprehensive book on evidence-based self-care approaches for CLBP

The major outcomes of interest in the study are back pain intensity and function. Medication use, quality of life, psychological parameters, and cost effectiveness are some of the other important outcomes that will be measured.

We have designed a hatha yoga protocol for the study. An expert panel in 2006-07 developed the yoga protocol by consensus and discussion after a systematic review of the lay and scientific literature on yoga and low back pain. Panel members had experience in several styles of hatha yoga including Anusara, Ashtanga, Iyengar, and Kripalu. One member had special expertise in leading yoga programs for minority women. The protocol was used in a 2007 pilot study of yoga compared to usual care for 30 patients with CLBP. It was further refined through a 2012 Yoga Dosing Study, where 95 participants were randomized to either once weekly or twice weekly yoga classes. Learning how to teach the protocol and adhering to the protocols described herein are essential for the study to be valid and the results generalizable.

© Boston Medical Center

In order to give your best to the study, it is critical to take care of yourself by taking the time to eat properly, get enough sleep and take time for yourself. Make sure you have time before class to prepare yourself so you are ready to give each class your best. Reading this manual in completion and making sure you know the lessons, postures and modifications will help you to guide participants safely through the study. Make sure to keep a balance of teaching the words from this manual with silence, allowing for silence during the class will guide participants into a deeper experience. The participants in this study may have complicated physical and psychological medical histories, so keep their needs in mind at all times during the classes. Give your support to each participant to guide them through beginning their own yoga practice and continuing on with home practice beyond the course of the study.

As a member of the Yoga Team, your participation in the study is extremely appreciated. Your commitment to providing the best possible yoga intervention to the study participants is critical for the success of the study. We acknowledge that yoga teachers' preferences and teaching styles may vary significantly, and it may be difficult sometimes to follow a proscribed treatment protocol when you may think someone would benefit from something different. For the purposes of this study, however, we ask you to please try to follow this protocol as closely as possible. As questions about the protocol, study, or logistics arise, please feel free to speak with anyone involved in the study including myself and the Research Coordinator.

Again, thank you for your enthusiasm and dedication to the Back to Health Study

Sincerely,

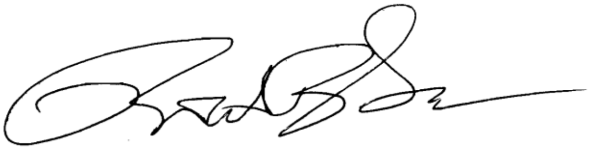

Robert B. Saper, MD MPH
Principal Investigator
Robert.Saper@bmc.org
(617) 414-6276

© Boston Medical Center
Program for Integrative Medicine & Health Care Disparities
Back to Health Trial

Low Back Pain

Low back pain (LBP) is the most common cause of pain in the United States, resulting in substantial morbidity, disability and cost to society. Approximately one-fourth of U.S. adults experience LBP at least one day over a three-month period. LBP accounts for 34 million office visits annually by family physician and primary care internists. Annual direct costs for LBP care in the U.S. are more than $50 billion and indirect costs (e.g. productivity) are estimated to be even greater. Back pain patients incur up to 75% more medical expenditures than patients without back pain. Back injury is the leading and most expensive cause of workers' compensation claims.

Health Disparities and Low Back Pain

The deleterious impact of LBP for people from low-income minority backgrounds is greater due to disparities in treatment and access. Although LBP prevalence in U.S. amongst whites, blacks, and Hispanics is similar, racial and ethnic disparities in access and treatment exist. Medical expenditures for LBP in minorities are 30% lower than for whites. For example, minorities with LBP receive less patient education, narcotic prescriptions, back surgery, specialty referrals, and intensive rehabilitation for occupational back injuries. Reasons for disparities may include lack of adequate health insurance, lower income, and less education, all factors associated with increased risk and severity of back pain. Attitudes and beliefs of providers and patients may also play a role. For example, physicians may assess a white patient's pain to be greater than a black patient's pain. However, the patient may perceive the opposite: the black patient may perceive her own pain greater than the white person perceives her pain. A history of racial discrimination experienced or perceived by a minority individual can also be associated with greater levels of back pain. Few intervention studies for LBP targeting minority populations have been conducted. *Although several studies have demonstrated racial and socioeconomic disparities in LBP treatment and outcomes, there is a large need for LBP intervention trials which specifically target minority underserved populations.*

Non-Specific Chronic Low Back Pain

CLBP lasting more than 12 weeks affects an estimated 5-10% of U.S. adults. Physicians identify a definite anatomic source for the back pain in only a small minority of patients. Examples include a large herniated disc, spinal canal stenosis, or vertebral compression fracture. The majority of patients however are classified as having non-specific chronic low back pain. Non-specific CLBP accounts for a majority of back-related health expenditures. The chronicity of pain for some LBP patients is marked. For example, 100% of our pilot subjects reported CLBP for > 1 year and one-third reported CLBP for >7 years. However, few non-pharmacologic intervention studies for CLBP have included ongoing structured maintenance components beyond an initial 8-16 week initial intervention period. In 43 non-pharmacologic RCTs systematically reviewed by Chou, only one exercise study contained a formal continuing exercise program for participants. To keep chronic

© Boston Medical Center

diseases such as diabetes and asthma well-controlled requires a chronic disease management model with ongoing evaluation and care. Similarly, optimizing long-term outcomes for CLBP will likely also require an ongoing chronic disease management approach. Therefore CLBP studies need to evaluate not only long-term follow-up, but long-term models designed to maintain clinical effectiveness and support patient adherence.

Treatment for Non-specific CLBP

Although there are a range of conventional pharmacologic, non-pharmacologic, and surgical procedures used for non-specific CLBP, most patients report only modest or moderate relief at best. In 2007 Chou and Huffman performed a systematic review and meta-analysis of non-pharmacologic treatments for back pain and authored evidence-based joint clinical practice guidelines on behalf of the American College of Physicians and American Pain Society for acute, sub-acute, and chronic low back pain. They recommended initial management for non-specific CLBP should include advice to remain physically active and education on back self-care. If necessary medication with good evidence for benefitting CLBP, such as acetaminophen or non-steroidal anti-inflammatory drugs, can be judiciously used. When needed, the guidelines recommend several conventional non-pharmacologic therapies including exercise therapy and cognitive-behavioral therapy.

Nonetheless, patient satisfaction with the effectiveness of conventional CLBP treatment is relatively low. Thus, there is a substantial need for research to identify more helpful therapies.

© Boston Medical Center

Yoga for Low Back Pain

Yoga
The National Center for Complementary and Alternative Medicine (NCCAM) classifies yoga as a mind-body therapy. Mind-body therapies encompass physical practices purported to affect the mind and conversely, mental practices that can impact the body. Yoga originated over 2000 years ago in India as a system of physical, moral, and spiritual principles and practices. Hatha yoga is the branch of traditional yoga that grew popular in the U.S. in the 1960s and consists of three core components: physical postures (*asanas),* breathing techniques (*pranayama*), and meditation. Multiple variations or schools of hatha yoga have arisen, such as Iyengar, Viniyoga, Ashtanga, Anusara, and Kripalu. These styles all use most of the basic hatha yoga postures, but vary in the intensity, pace, and selection of they are practiced.

Patterns of Yoga Use in the United States
Yoga is increasingly popular in the U.S. Our study of national prevalence of yoga use in 1998 was the first of its kind and found 3.8% of U.S. adults used yoga in the previous year. Twenty-one percent listed back pain as a reason for yoga use, with a majority believing it was very or somewhat helpful. We also reported that 2002 use increased to 5.1% which has subsequently grown to 6.1% in 2007 (13.1 million). One-third of 2007 yoga users who used yoga for a specific medical condition used it for back pain (Personal communication, Maria Chao, DrPH, MPA). Although yoga's popularity has increased, use among minorities and individuals with lower income or education is less common. Data from the 2007 National Health Interview Survey show yoga use in whites was 6.3% vs. 3.3% in African Americans; 6.6% in non-Hispanics vs. 2.9% in Hispanics; 9.5% in college-educated individuals vs. 1.9% of individuals who did not enter college; 8.6% of individuals in the highest income quartile vs. 4.9% of individuals in the lowest quartile. Minorities and people with low socioeconomic status are more likely to have undertreated back pain and also less likely to use practices such as yoga. If complementary therapies such as yoga for back pain are to be rigorously studied, trials need to target all affected populations including low income minorities.

Studies of Yoga for CLBP
Karen Sherman, PhD, a consultant on this project, randomized 101 participants to 12 weeks of yoga classes based on the Viniyoga style, 12 weeks of exercise classes, or education with a back pain self-help book. Most participants were white, middle income, college-educated with mild to moderate CLBP. No group was superior at 12 weeks for the symptom bothersomeness score. For back-related function at 12 weeks measured by the Roland Morris Disability Questionnaire, yoga was superior to education and exercise). However, for function at 26 weeks, yoga was superior to education only. Symptom bothersomeness for yoga at 26 weeks was also superior to education as well as exercise. Yoga participants reported statistically less pain medication use at 26 weeks than with exercise or education. Based upon Sherman's

study, the American College of Physicians/ American Pain Society practice guideline lists yoga as having fair evidence for a moderate benefit for CLBP. More recently Williams compared 24 weeks of biweekly Iyengar yoga to usual care in a RCT of 90 insured patients with CLBP. Yoga participants had greater reductions in pain and disability as measured by the Oswestry Disability Index than controls at 24 weeks. Depression scores also significantly decreased more in the yoga group than controls. Sherman is conducting a larger NCCAM-funded trial (5U01AT003208) comparing yoga, group therapeutic exercise classes, and usual care in a predominantly white middle class working HMO population. If yoga is found to be effective, physical (e.g., flexion), physiologic (e.g., neuroendocrine), and psychological (e.g., mental focus, mental stress) factors will be explored as possible mechanisms. Lastly, David Torgerson of the University of York in Great Britain has completed recruitment for a multi-site pragmatic trial of 313 adults recruited form general practices randomized to 12 weeks of yoga or usual care.

Despite several promising published and ongoing studies of yoga for CLBP, none to date have targeted minority lower income populations nor compared yoga's effectiveness to what physicians most commonly recommend, i.e., exercise therapy individually administered by physical therapists.

© Boston Medical Center

The Back to Health Study Design

The Back to Health Study is a 52-week comparative effectiveness randomized controlled trial of individually delivered PT, once per week yoga classes, and an educational book on self-care for CLBP in 320 individuals from predominantly minority backgrounds recruited from Boston Medical Center and affiliated community health centers. The 52 week trial starts with an initial 12 week Treatment Phase followed by a 40 week Maintenance Phase.

For the 12-week Treatment Phase, participants are randomized in a 2:2:1 ratio into (1) a standardized evidence-based exercise therapy protocol individually delivered by a physical therapist and supplemented by home practice; (2); a standardized once per week hatha yoga class supplemented by home practice; and (3) education delivered through a self-care book. The study co-primary endpoints are mean pain intensity over the previous week measured on an 11-point numerical rating scale and back-specific function measured using the 23-point modified Roland Morris Disability Questionnaire. Yoga participants receive yoga materials (e.g., mat, block, strap), a participant guide manual, and a DVD to help them with home practice

For the 40-week Maintenance Phase, yoga participants will be re-randomized in a 1:1 ratio to either structured ongoing yoga classes once per week or a continued home practice without ongoing structured yoga classes. The home practice group will have access to the home practice materials received during the Treatment Phase. The home practice group will also receive a list of community yoga classes available.

The Study Flow Diagram on the next page illustrates the overall study design:

© Boston Medical Center

Back to Health Study Flow

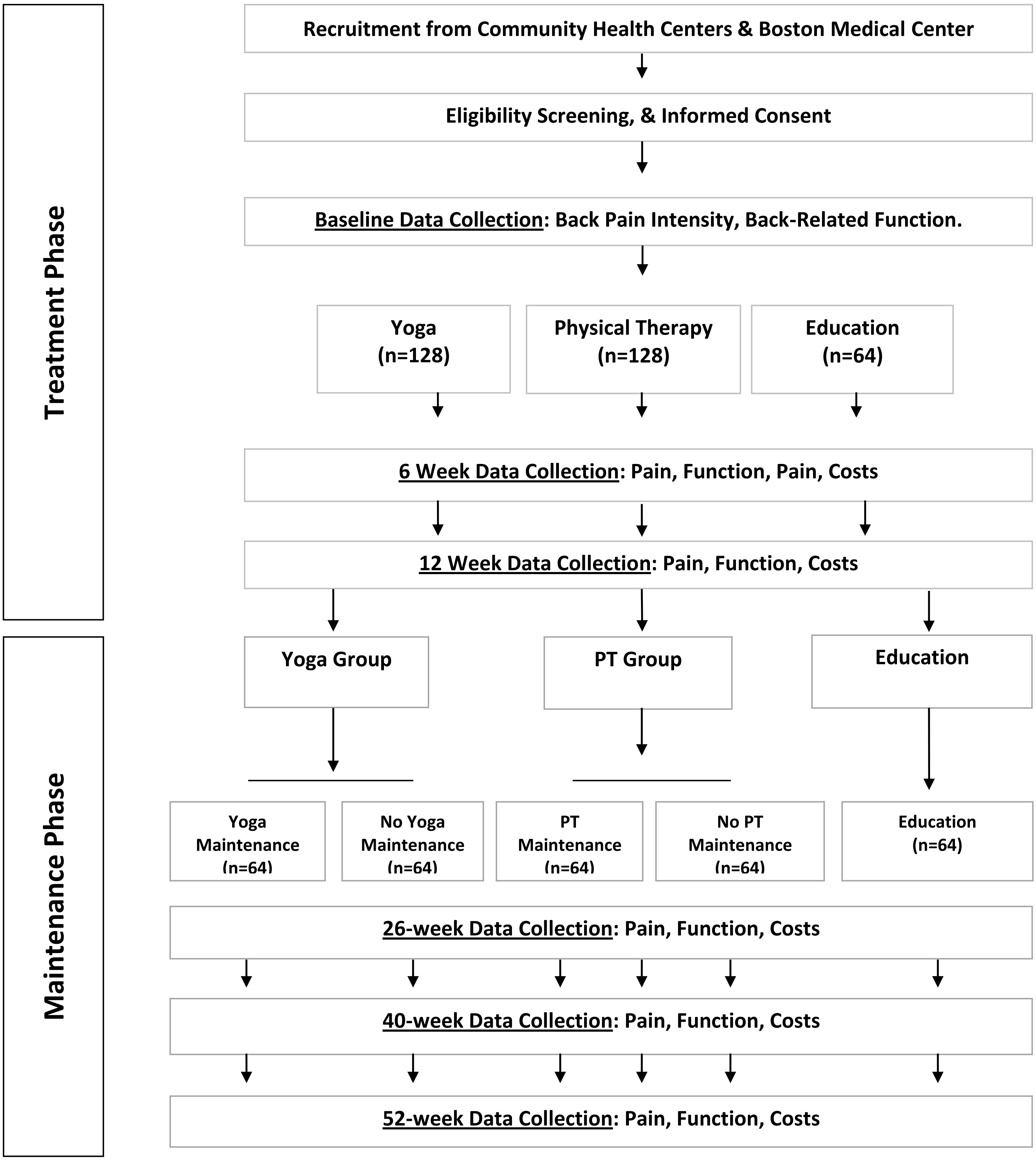

© Boston Medical Center

Yoga Teacher Training

Study-Specific Training

All yoga teachers and yoga teacher assistants on the Yoga Study Team have substantial professional experience in treating patients and specifically those with low back pain. Training specifically for participating on the Yoga Study Team therefore is mostly focused on learning the specific treatment protocols used. These may be more or less familiar to each yoga teacher depending upon their own educational background and professional experience. Study-specific training each yoga teacher must complete to participate in the study include:

1. Reading this training manual thoroughly.
2. Attending study-specific trainings on the protocol led by previously trained yoga teachers.
3. Training in Human Subjects Protection. Federal laws governing the conduct of human subject research mandate that all study personnel having contact with study participants undergo training in human subjects protection. Human Subjects Protection training can be achieved in one of two ways:

    1. Completing an online training sponsored by the National Institutes of Health. This takes approximately 2 hours.

    2. Participating in an in-person training offered at Boston University Medical Campus. These are offered periodically and can be from 2-4 hours in length.

Information about NIH Research Certification and how to obtain certification can be found under the "Research Certification" section, at the following website: http://www.bumc.bu.edu/ocr/certification/

National Institutes of Health

Online training through the National Institutes of Health (NIH) can be obtained through the following website: http://phrp.nihtraining.com

Boston University Medical Campus Clinical Research

Boston University Medical Campus also hosts in-person training offered in the Evans Bio-Medical Research Building (650 Albany Street, 7th floor, room 714). An RSVP is required to attend a seminar by either calling the Office of Clinical Research or by emailing irbtemp@bu.edu.

The contact information for the Office of Clinical Research and the on-site training schedule can be found at the following website:

Yoga Teacher Training

http://www.bumc.bu.edu/ocr/certification/training/

Please contact one of the following Back to Health Study research staff for further assistance:

Chelsey Lemaster, Research Coordinator
Chelsey.lemaster@bmc.org
617.414.6201

Daniel Do, Research Coordinator
Daniel.do@bmc.org
617.414.4464

Data Collection

The vast majority of "data" for this study will be collected by the study staff and not the yoga instructors. Data collection by the research staff usually occurs *before* the onset of the yoga class scheduled for that day and is conducted in such a way to minimize any interference to the class. However, we do ask participants to fill out a daily Home practice log and submit the log to the yoga teachers on a weekly basis. Yoga teachers and study staff should encourage participants to complete logs truthfully.

The research team will collect data at the following time points:

- Baseline (Week 0)
- Week 6
- Week 12
- Week 26
- Week 40
- Week 52

Overview of Yoga Classes in the Maintenance Phase

After the completion of the 12 week Treatment Phase, all participants who were initially randomized to the yoga arm and have attended at least 1 class during the Treatment Phase, will be re-randomized in a 1:1 ratio to either a structured ongoing maintenance yoga program for 40 weeks or no maintenance yoga program. Similarly, the physical therapy participants will be placed into either a structured ongoing PT maintenance yoga program for 40 weeks or no maintenance yoga program. The education participants will be encourage to continue to review and follow the recommendations of their educational materials.

Yoga participants randomized into the structured yoga maintenance group will be asked to attend drop in yoga classes which will occur once per week for 40 weeks. In addition, structured yoga maintenance participants will be encouraged to continue with their yoga home practice. Yoga participants randomized into the non-structured yoga maintenance group will not be asked or allowed to attend the drop-in yoga class; however they will be encouraged to continue with their home practice and attend community yoga classes if desired.

© Boston Medical Center

Yoga Teacher Roles, Responsibilities & Logistics

© Boston Medical Center

Yoga Teacher Roles and Responsibilities

Yoga teachers (YTs) will be assisted in each class by yoga teacher assistants (YTAs). YTAs are recently certified yoga teachers who will assist the YTs with class logistics (e.g. setting up the yoga class, collecting home practice logs, handing out gift cards) and providing assistance to participants during the class (e.g. helping participants with adjustments and handing out props). YT roles and responsibilities are outlined as follows:

Before Each Class (arrive at least 15 minutes early):

- Check in with the YTA to share any relevant information learned about participants as well as any other issues or concerns.

On the First Day of Class (Arrive 45 minutes early):

- Welcome participants into the space and introduce yourselves.
- Inform participants about logistics (e.g. bathrooms, where to put their items), taking off shoes, not eating or drinking in class, cleaning their mats, etc. Ask them to please turn off cell phones during class.
- Assure participants about confidentiality within the group, letting them know they can talk to others outside of class about their own experience, but not about the other participants.

During Class:

- Position yourself so that participants can see you and you can see them during class.
- Assist participants with props and provide support to participants when needed.

After Class:

- Use your Teacher Notes to communicate with the study staff about supplies, questions, or concerns. Put any forms in the locked form box in the Yoga Supplies Bin. The study staff collects these forms each week and will respond to your request

(Note: for more urgent issues, especially if you have a concern regarding a participant's safety or possible injury, please do not hesitate to contact Dr. Saper, Principal Investigator or the Treatment Coordinator on their cell phones directly).

Yoga Assistant Teacher Roles and Responsibilities

Before Each Class (arrive at least 15 minutes early):

- Check in with the Yoga Teacher to share any relevant information learned about participants as well as any other issues or concerns.
- Put up any signs about turning off cell phones, directions, restrooms etc.
- Turn on lights, clear space, and set up the props (mats, belts, blocks, blankets). Make sure there is sufficient room around each mat for the assistant to be able to walk around and model postures. Depending upon the health center, you may need to clear more furniture than others.
- Have attendance sheet, pens, and extra Weekly Home Practice Logs near the door and in the same place each week.
- Be sure everyone signs the attendance sheet legibly and remind participants to note if contact information has changed.
- Make sure the attendance sheet is dated and that all participants present are recorded.
- Create a separate area for participants' belongings away from the yoga space.
- Place index cards with participants' first names next to their mat. Place participants who need extra help closer to the teacher or near a wall for support during postures.

On the First Day of Class (Arrive 45 minutes early):

- Welcome participants into the space and introduce yourselves.
- Have one chair behind each mat so that participants can sit down before getting down onto mat.

During Class:

- Assist participants with props and provide support to participants when needed.

After Class:

- Share cleaning materials with participants and show them how to clean their mats. Store yoga supplies and leave the space as it was found.
- Place the Weekly Home Practice Logs into locked form box.
- Both the yoga teachers and yoga assistants should complete the Yoga Teacher Class Notes

Sickness and Absence Policy

Foreseeable Absences

Your commitment to be present and teach at **all** assigned yoga class is critical for the participants and success of the study. However, we do understand there may be unusual or unforeseen circumstances necessitating that you miss class. Should you need to be absent from a class, please notify the Yoga Team Coordinator (contact information below) **at least one week prior to the class date.** She will send a substitute request email to the participating yoga teachers to arrange for an alternate. If no one has responded within 48 hours, she will follow up with phone calls to find a fill-in.

Sickness

If you are ill and unable to teach a class, please notify the Yoga Team Coordinator **as soon as possible** (preferably **24 hours in advance** if possible). She will try to arrange for a substitute teacher for your class.

Emergency

In the rare event of an emergency the day of a class, please call **Yoga Coordinator** at 617-414-6248. Please do **not** use email or voicemail in the event of an emergency.

Contact Information

Yoga Team Coordinator
Office: 617-414-6248

© Boston Medical Center

Boston Medical Center Yoga for Chronic Low Back Pain Intervention

© Boston Medical Center

Back to Health: An Overview

People are encouraged to participate in this study if they are experiencing chronic low back pain and wish to achieve a higher level of functioning and minimization or alleviation of pain. The hatha yoga curriculum described in this teacher's manual was originally developed in 2007 by an expert panel led by the Principal Investigator, Dr. Saper, and used successfully in a pilot study of 30 predominantly low income minority participants with chronic low back pain. (Saper et al, Alt Ther Health Med 2009). In the fall of 2012, the yoga curriculum was further refined in the Yoga Dosing Study, a randomized controlled trial of 95 participants with chronic low back pain, comparing 12 weeks of once weekly vs. twice weekly classes. The Dosing Study found both class schedules similarly effective for improving back pain and function. Thus, for the current Back to Health study, once per week protocol is being used. Additional minor changes have been made to the protocol based on yoga teacher and participant feedback from the Dosing Study.

These yoga classes for chronic low back pain are different from regular yoga classes in that the curriculum includes standardized format and teaching directives that can be replicated and delivered by any qualified yoga teacher. The treatment phase is 12 weeks long. Participants in the yoga group will take one 75 minute yoga class per week. The twelve weeks are divided into four segments. Each segment has a name and lasts 3 weeks. The segments build upon what participants have learned in previous segments. Each segment has a set of specific poses, breathing exercises and yoga philosophies. The goal of the four segments is to teach basic hatha yoga postures in a carefully calibrated sequence that supports healing of the back. The four segments are entitled: *Opening to Something Greater, (Segment 1)*, *Listening to the Body (Segment 2)*, *Engaging Your Power (Segment 3)* and *Bringing It Home (Segment 4)*.

The Four Segments

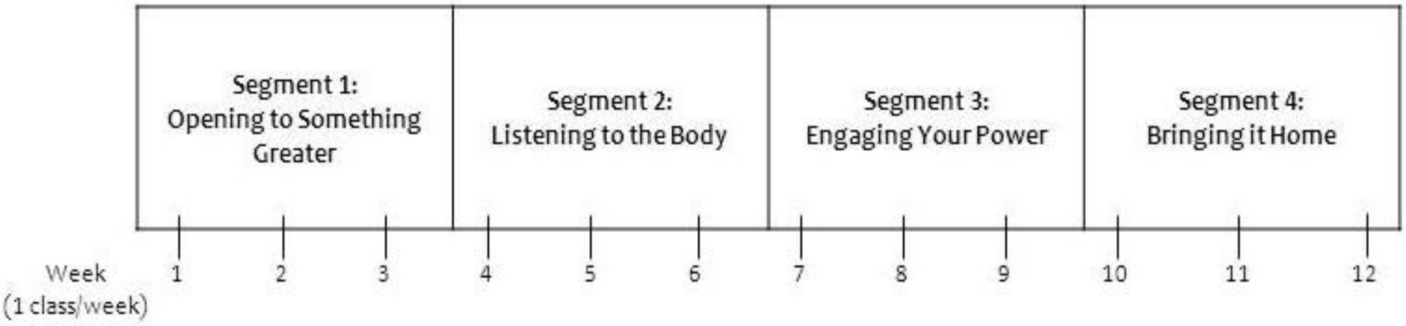

Segment 1: Opening to Something Greater (Weeks 1-3)

In this first segment, participants are introduced to yoga and the possibility of change in their back pain and change in themselves. They are taught how controlled breath work is the foundation for yoga. Participants practice a variety yoga breathing exercises (e.g. awareness of normal breath and evening out the breath in the lungs). They are introduced to intentional relaxation and practice several poses such as: Child’s Pose, Cobra Pose, Bridge Pose, Knees to Chest, Knees Together Twist and Reclining Cobbler Pose. Each week highlights briefly an aspect of yoga philosophy (definition of yoga, non-violence, honest). How the yoga philosophy concept applies both to yoga practice and daily life is emphasized.

Segment 2: Listening to the Body (Week 4-6)

Participants are taught how to listen to their individual bodies and to develop self-compassion. They are encouraged to begin building strength in standing postures, developing stability in balancing poses, stretching in gentle backbends, and learning to use the muscles to support the spine—all elements to help their low back pain. Participants are taught to use their experience on the mat to create a foundation of balance in their lives by trusting their own experience and listening to their bodies. Participants continue with postures learned in the first segment and learn new postures such as Wall Dog Pose, Triangle at Wall Pose, and Standing Forward Bend at

Wall. Yoga philosophy principles highlighted in Segment 2 include purity, cleanliness, gratitude and discipline

Segment 3: Engaging Your Power (Weeks 7-9)

Participants are challenged to apply the strength and self-awareness that they have started to discover in more challenging standing, twisting, and back bending postures. They are encouraged to focus on the strength and mobility in their low back as they try more difficult postures such as Warrior I at Wall Pose and Chair Pose. Participants are introduced to the yogic concepts of self-awareness, self-acceptance and moderation.

Segment 4: Bringing it Home (Weeks 10-12)

Participants perform more challenging postures including Downward Facing Dog Pose and Baby Dancer Pose. The notions of balance, self-compassion and community are presented in this segment.

© Boston Medical Center
Program for Integrative Medicine & Health Care Disparities
Back to Health Trial

Individual Class Format

Each class throughout the 12 weeks has a similar semi-standard format:

Curriculum Elements	Time (min)
Check In with Participants	3
Lesson Introduction and Yoga Philosophy	3
Yoga Postures	55
Closing Relaxation	5
Closing	2
Total Time:	75

*The order of these three sections is flexible, though they should always occur at the beginning of the class after checking in with participants. Teachers should use their own discretion for the sequence of these elements in each class. These three sections should be 10 minutes in total, with equal time divided between philosophy, breathing and relaxation. Be mindful of any information garnered while checking in with participants to help you decide the order of these sections.

Check-in With Participants	5 minutes

Check-in with participants takes places at the beginning of each yoga class. It is mandatory that teachers check in with each participant. The yoga teacher should ask participants how their yoga practice is proceeding. Participants should be particularly encouraged to share both progress and any setbacks or injuries from their yoga home practice. Encourage participants to let the teacher know if they are experiencing any new symptoms during or after the class. During the check-in, ask participants openly on how yoga and home practice are going. Create an atmosphere of safety and acceptance so participants can express both positive and negative experiences. Acknowledge and validate all responses without judgment. Comment as needed.

Individual Class Format

Lesson Introduction and Yoga Philosophy	3 minutes

Lesson Introduction and Yoga Philosophy are intended to familiarize participants with the philosophical principles of yoga. Yoga philosophy provides the participants a framework for health and wellness while promoting awareness and self-discipline. Teachers utilize poems, readings, and commentaries related to the philosophical principles to inspire the participants to practice greater self-care. The suggested readings for each week are in this manual. However, teachers are welcome to bring in appropriate readings of their choosing that speak to the relevant topic. The manual give "talking points" that can be used either verbatim or as suggestions to guide teachings. During the study we cover the following topics:

- Week 1 (Define Yoga)
- Week 2 (The Practice of Non-violence)
- Week 3 (Truth and Honesty)
- Week 4 (Purity/Cleanliness)
- Week 5 (Contentment/Gratitude)
- Week 6 (Discipline/Will Power)
- Week 7 (Study and Awareness)
- Week 8 (Acceptance/Letting Go)
- Week 9 (Moderation/Not Greed)
- Week 10 (Balancing Effort and Ease)
- Week 11 (You are What You Practice)
- Week 12 (We're all in This Together)

Please see a summary of the 12 principles on pg. 109.

© Boston Medical Center
Program for Integrative Medicine & Health Care Disparities
Back to Health Trial

Individual Class Format

Relaxation Exercise	3 minutes

Relaxation is a crucial component of the program, providing participants with regularly occurring opportunities to renew, restore and recharge with yoga. Participants are encouraged to set aside actions, thoughts, stress, worries and the events of the day and focus on the present moment in class. Different relaxation techniques are used, including muscle relaxation and body scan. There is a menu of relaxation exercises that yoga teachers may choose from listed in this manual. Yoga teachers may use the relaxation exercises provided in the manual or a variation of their own choosing. Please employ a variety of relaxation techniques throughout the course of the 12 weeks.

Breathing Exercise	4 minutes

There is class time specifically dedicated to teaching breathing techniques in each session. Over the course of the 12 week curriculum breath awareness, the 3- part breath, rhythmic deep breathing, and the ocean (Ujjayi) breath are taught. Teachers and Teacher Assistants should model and demonstrate all breathing techniques. Yogic breathing techniques must be emphasized with every posture as well as during breathing exercises.

Yoga Postures	53 minutes

The yoga postures have been selected for their safety and potential to help chronic low back pain. They gradually increase in difficulty over the 12-week intervention. Support the participants through individual attention, yoga props as needed, and modifications to accommodate individual needs. The postures include both warm-ups, which prepare the participants' bodies and minds for the yoga postures by moving during the postures, and cool-downs. During cool-downs, the postures are intended to transition participants from more strenuous poses in the yoga postures

© Boston Medical Center

to the closing relaxation (Savasana). In cool-downs, participants will be instructed to hold postures while inhaling and exhaling, instead of breathing through movements.

Closing Relaxation	5 minutes

The closing relaxation is led by the Yoga Teacher while the participants are in Savasana. The closing relaxation provides participants an opportunity to integrate the different experiences of yoga practice. The closing relaxation will be less structured than the opening relaxation. This part of the practice is meant to draw participants into a deeper state of relaxation. At the beginning of the study, yoga teachers should give clear guidance to participants throughout the relaxation section. As the study progresses, allow for more silence to enter into the practice.

Closing	2 minutes

The purpose of the closing is to allow time for any announcements regarding the study. On a regular basis, teachers and teacher assistants should remind participants to turn in their Weekly Home Practice Logs and to encourage them to continue their home practice. Say good bye to everyone *by name*, making eye contact.

© Boston Medical Center

Segment 1: Opening to Something Greater
Week 1

Check In With Participants	5 minutes

- Introduce teacher and teaching assistant
- Take attendance, learning how to pronounce names
- Welcome participants to yogic intervention to learn techniques to alleviate back pain
- Share goals and ask for their commitment to program

*Lesson Introduction and Yoga Philosophy	3 minutes

Define Yoga
- Union of the body, mind, breath and soul
- Quieting the mind
- Being in the present moment
- Physical exercise strengthening and toning the body and mind, bringing about a balanced, peaceful state, reducing stress and suffering
- Accepting the joys and sorrows of life.

*Relaxation	3 minutes

- Prepare the body for relaxation as described on pg. 86
- Use the menu of relaxation exercises listed on pg. 88 to guide participants or incorporate a relaxation exercise from your own practice. Include a variety of relaxation exercises throughout the 12 weeks of the study.

*Breathing Exercise	4 minutes

Awareness of Normal Breath
1. Breathing through the nose with the mouth closed, notice the feel of the air as it comes into your body and then goes out. Be aware of your body breathing in, saying to yourself, "I am breathing in." Be aware of your body breathing out, saying, I am breathing out." Repeat.
2. Don't try to control the breath, just be aware of your body breathing without any effort on your part.

Yoga Postures	53 minutes

1. Knees to chest
2. Knees together twist
3. Pelvic tilt
4. Mountain
5. Shoulder stretches 1 & 2
6. Crescent Moon, one arm up
7. Modified Chair with hands on hips
8. Chair twist, standing
9. Child's pose in chair

© Boston Medical Center
Program for Integrative Medicine & Health Care Disparities
Back to Health Trial

10. Wall Dog
11. Modified Locust with one leg up at a time
12. Sphinx
13. Bridge
14. Supported Bridge
15. Knees together twist
16. Knees to chest

Closing Relaxation	5 minutes

- Savasana
- Use this time to guide participants through a less structure relaxation period. Assist participants to enter a deeper state of relaxation. Give participants specific guidance on how to do deep relaxation.

Closing	2 minutes

- Praise participants for their yoga in class.
- Remind participants to practice yoga at home for 30 minutes per day and to fill out Weekly Home Practice Logs.
- Remind participants to be aware of their breath during their yoga practice and also throughout the day.

© Boston Medical Center
Program for Integrative Medicine & Health Care Disparities
Back to Health Trial

Segment 1: Opening to Something Greater

Week 2

Check In With Participants	5 minutes

- Ask participants about what came up for them since last class re: yoga intervention, practicing at home, and keeping practice logs.
- Discuss setting up a realistic practice schedule

*Lesson Introduction and Yoga Philosophy	3 minutes

The Practice of Non-Violence

- Not harming ourselves or others through actions, words, or thoughts
- Accepting and nurturing ourselves, treating ourselves and others with compassion and kindness.
- Discuss the difference between unhealthy harmful pain and healthy discomfort in poses; learning the difference between challenging ourselves without over straining our bodies.

*Relaxation	3 minutes

- Prepare the body for relaxation as described on pg. 86
- Use the menu of relaxation exercises listed on pg. 88 to guide participants or incorporate a relaxation exercise from your own practice. Include a variety of relaxation exercises throughout the 12 weeks of the study.

*Breathing Exercise	4 minutes

- Continue practicing "Awareness of Normal Breath," now using the steps outlined below:
    1. Notice the feel of the air as it comes into your body and then goes out. Where do you feel the breath in your body the most?
    2. Notice the temperature of the air as it comes into the nose at the nostrils and again as the air goes out. Does the temperature of the air change from the inhale to the exhale?
    3. Notice the quality of the breath. Is the breath bumpy or smooth, light or heavy, starting and stopping?
    4. Notice the normal length of the inhale and exhale. Is the inhale longer or shorter than the exhale?
    5. These questions don't need answers, they are to focus attention and continue normal breath awareness.

Yoga Postures	53 minutes

1. Knees to chest
2. Knees together twist
3. Pelvic tilt
4. Mountain

© Boston Medical Center
Program for Integrative Medicine & Health Care Disparities
Back to Health Trial

5. Shoulder stretches 1 & 2
6. Crescent Moon, one arm up
7. Modified Chair with hands on hips
8. Chair twist, standing
9. Child's pose in chair
10. Wall Dog
11. Modified Locust with one leg up at a time
12. Sphinx
13. Bridge
14. Supported Bridge
15. Knees together twist
16. Knees to chest

Closing Relaxation	5 minutes

- Savasana
- Use this time to guide participants through a less structure relaxation period. Assist participants to enter a deeper state of relaxation. Give participants specific guidance on how to do deep relaxation.

Closing	2 minutes

- Praise participants for their yoga in class.
- Remind participants to practice yoga at home for 30 minutes per day and to fill out Weekly Home Practice Logs.
- Remind participants to be aware of their breath during their yoga practice and also throughout the day.

© Boston Medical Center

Segment 1: Opening to Something Greater

Week 3

Check In With Participants	5 minutes

- Ask participants how practice went, what challenges they encountered in practice, what feels realistic, and if they can they set a new goal for practice this week.

*Lesson Introduction and Yoga Philosophy	3 minutes

Truth and Honesty

- Being honest with ourselves and others; not taking what doesn't belong to us.
- To look at ourselves honestly, we need to accept and have compassion for our weaknesses.
- When we look at ourselves honestly, we may find that we may need to do certain poses differently than everyone else in the class, depending on what is safe and healthy for us in that moment.

*Relaxation	3 minutes

- Prepare the body for relaxation as described on pg. 86
- Use the menu of relaxation exercises listed on pg. 88 to guide participants or incorporate a relaxation exercise from your own practice. Include a variety of relaxation exercises throughout the 12 weeks of the study.

*Breathing Exercise	4 minutes

1. Review normal breath awareness.
2. Breathing in, synchronize the chest expanding upward and outward at the same time. Breath out quietly, emptying the lungs slowly. Continue consciously observing and feeling the flow of the breath and the slow expansion and contraction of the breath.

Yoga Postures	53 minutes

1. Knees to chest
2. Knees together twist
3. Pelvic tilt
4. Cat/Cow
5. Mountain
6. Shoulder stretches 1 & 2
7. Crescent Moon, one arm up
8. Modified Chair with hands on hips
9. Chair twist, standing
10. Child's pose in chair
11. Wall Dog
12. Modified Locust with one leg up at a time

13. Sphinx
14. Cobra
15. Bridge
16. Supported Bridge
17. Knees together twist
18. Knees to chest

Closing Relaxation	5 minutes

- Savasana
- Use this time to guide participants through a less structure relaxation period. Assist participants to enter a deeper state of relaxation. Give participants specific guidance on how to do deep relaxation.

Closing	2 minutes

- Remind participants to enjoy their at-home yoga practice.
- Remind them to bring in weekly home practice logs.

Segment 2: Listening to the Body

Week 4

Check In With Participants	5 minutes

- Query participants about what they are noticing since beginning yoga. Do not prompt for positive responses though acknowledge and validate responses without judgment.

*Lesson Introduction and Yoga Philosophy	3 minutes

Purity/Cleanliness

- Yoga postures and breathing exercises cleanse the body on the inside, removing toxins and tension, bringing in the fresh flow of oxygen.
- Yoga clears the body and mind of clutter, opening us up to new possibilities, and bringing clarity and freshness to the body and mind.
- Our body is our temple or sacred space. Keep it clean by being aware of what we put into it (e.g. healthy food), what kind of company we keep (e.g. healthy relationships), and what we do (e.g. healthy balanced of work, exercise, and leisure).

*Relaxation	3 minutes

- Prepare the body for relaxation as described on pg. 86
- Use the menu of relaxation exercises listed on pg. 88 to guide participants or incorporate a relaxation exercise from your own practice. Include a variety of relaxation exercises throughout the 12 weeks of the study.

*Breathing Exercise	4 minutes

Introduce "3-Part Breath": Belly, lower chest and upper chest.

- Teach participants the location of the diaphragm muscle and how it helps the lungs expand and contract.
    1. Place hands on the lower belly and naval area. Relax belly and breathe normally with easy, comfortable breaths. Notice if there is any movement under the hands. The belly should rise on the inhale and fall on the exhale.
    2. Place hands on the lower side ribs (floating ribs). With the belly relaxed, gently try to breathe in so that the ribs move outward and expand into the hands. Breathe out so the ribs move and contract inwards. Breathe out, moving lower ribs out into hands and back in like an accordion—going in and out.
    3. Place hands on upper chest, under collarbones. Notice if the upper chest has any movement while breathing. The upper chest should rise with the inhale and fall with the exhale. Be careful not to tense neck and shoulders on inhale.
    4. Now combine all three parts of the breath. As you inhale, expand the belly, then the lower side ribs and then the upper chest. As you exhale, allow the upper chest to fall, the lower ribs to contract and the belly to fall.

5. Take normal, slow, smooth, easy breaths, breathing in until there is a full feeling. Breathe out until there is an empty feeling. Discuss how the torso is like a glass that fills from the bottom to the top when it is filled with water. In the same way the lungs fill from the bottom to the top with air. Exhale, emptying the lungs slowly from the top to the bottom, from upper chest down to belly.

Yoga Postures	53 minutes

1. Knees to chest
2. Knees together twist
3. Pelvic tilt
4. Cat/Cow
5. Mountain
6. Shoulder stretches 1 & 2
7. Crescent Moon, one arm up
8. Modified Chair with hands on hips
9. Chair twist, standing
10. Child's pose in chair
11. Wall Dog
12. Modified Locust with one leg up at a time
13. Sphinx
14. Cobra
15. Bridge
16. Supported Bridge
17. Knees together twist
18. Knees to chest

Closing Relaxation	5 minutes

- Savasana
- Use this time to guide participants through a less structure relaxation period. Assist participants to enter a deeper state of relaxation. Give participants specific guidance on how to do deep relaxation.

Closing	2 minutes

- Praise participants for their progress and success in yoga. Ask participants to notice if they see more opportunities for gratitude in their lives this week.
- Remind participants to practice each day and come to each class with the Weekly Home Practice Log and to fill out their Cost Diaries at home.

Segment 2: Listening to the Body

Week 5

Check In With Participants	5 minutes

- Query participants about what they're noticing since beginning yoga (mental/emotional and physical).
- Discuss how with home practice is going (successes and challenges).

*Lesson Introduction and Yoga Philosophy	3 minutes

Contentment/Gratitude

- A feeling of not wanting or needing anything else in this moment; of being at peace, feeling whole, and being grateful for what we have right now.
- Being content is a practice, something to cultivate. It's easiest to cultivate when we're feeling good and our life is going well. With steady practice we will be able to draw on our reservoir of contentment when life isn't going well.
- Focusing on what we <u>can</u> do in a pose, not what we <u>cannot</u> do.

*Relaxation	3 minutes

- Prepare the body for relaxation as described on pg. 86
- Use the menu of relaxation exercises listed on pg. 88 to guide participants or incorporate a relaxation exercise from your own practice. Include a variety of relaxation exercises throughout the 12 weeks of the study.

*Breathing Exercise	4 minutes

- Teach participants the location of the diaphragm muscle and how it helps the lungs expand and contract.
  1. Place hands on the lower belly and naval area. Relax belly and breathe normally with easy, comfortable breaths. Notice if there is any movement under the hands. The belly should rise on the inhale and fall on the exhale.
  2. Place hands on the lower side ribs (floating ribs). With the belly relaxed, gently try to breathe in so that the ribs move outward and expand into the hands. Breathe out so the ribs move and contract inwards. Breathe out, moving lower ribs out into hands and back in like an accordion—going in and out.
  3. Place hands on upper chest, under collarbones. Notice if the upper chest has any movement while breathing. The upper chest should rise with the inhale and fall with the exhale. Be careful not to tense neck and shoulders on inhale.
  4. Now combine all three parts of the breath. As you inhale, expand the belly, then the lower side ribs and then the upper chest. As you exhale, allow the upper chest to fall, the lower ribs to contract and the belly to fall.
  5. Take normal, slow, smooth, easy breaths, breathing in until there is a full feeling. Breathe out until there is an empty feeling. Discuss how the torso is like a glass that fills from the bottom to the top when it is filled with water. In the same way the lungs fill from the bottom to the top with air. Exhale, emptying the lungs slowly from the top to the bottom, from upper chest down to belly.

Segment 2: Listening to the Body

Week 5

Yoga Postures	53 minutes

1. Knees to chest – alternate knees then both, moving in and out with breath
2. Knees together twist – moving back and forth with breath
3. Pelvic tilt
4. Toe taps -pelvic tilt with feet off floor
5. Table Top with leg extended – lift leg up and down slowly
6. Child's pose
7. Mountain
8. Shoulder openers 1, 2, & 3
9. Crescent Moon
10. Modified Chair – arms out parallel to floor
11. Standing chair twist
12. Wall Dog
13. Triangle at wall
14. Standing forward bend at wall
15. Modified Locust
16. Sphinx
17. Cobra
18. Down Dog
19. Bridge
20. Extended leg stretch #1
21. Knees together twist
22. Supported Bridge
23. Knees to chest
24. Reclined cobbler

Closing Relaxation	5 minutes

- Savasana
- Use this time to guide participants through a less structure relaxation period. Assist participants to enter a deeper state of relaxation. Give participants specific guidance on how to do deep relaxation. Begin to allow more silence to enter the closing relaxation. Allow participants the time and space in the silence to enter a deeper state of relaxation.

Closing	2 minutes

- Reiterate importance of filling out logs and practicing on days when there is no class.
- Remind participants that next week is a data collection week and that they will be receiving a call from the study staff with instructions.

Segment 2: Listening to the Body

Week 6

Check In With Participants	5 minutes

- Find out what's been working and what hasn't in the participants' yoga practice
- Discuss the difficulty of practice.
- Discuss the challenge of learning to listen to their bodies and have self-compassion.

*Lesson Introduction and Yoga Philosophy	3 minutes

Discipline/Will Power

- We need discipline, will power, and patience to stay with our practice of yoga and our goals in life.
- It takes patience and will power to change our behavior for a well-balanced life.
- With patience and will power, discipline can be practiced in small steps, working up to bigger goals just like with difficult yoga poses. Start with part of the pose, and with steady practice, we can move toward the whole pose.

*Relaxation	3 minutes

- Prepare the body for relaxation as described on pg. 86
- Use the menu of relaxation exercises listed on pg. 88 to guide participants or incorporate a relaxation exercise from your own practice. Include a variety of relaxation exercises throughout the 12 weeks of the study.

*Breathing Exercise	4 minutes

- Introduce "Lengthening each Exhale"
    1. Discuss how lengthening the exhale helps us relax, release tension, and stress.
    2. Review synchronizing and making the breath even.
    3. Start with normal breathing, then exhale quietly until the lungs feel empty but without pressing down on the abdomen.
    4. Inhale normally through the nose.
    5. Exhale slowly, deeply, steadily, until the lungs feel empty.
    6. Continue with normal inhales and slow, deep, steady, and conscience exhales, lengthening each exhale comfortably.

Yoga Postures	53 minutes

1. Knees to chest – alternate knees then both, moving in and out with breath
2. Knees together twist – moving back and forth with breath
3. Pelvic tilt
4. Toe taps -pelvic tilt with feet off floor
5. Table Top with leg extended – lift leg up and down slowly
6. Child's pose
7. Mountain
8. Shoulder openers 1, 2, & 3
9. Crescent Moon

10. Modified Chair – arms out parallel to floor
11. Standing chair twist
12. Wall Dog
13. Triangle at wall
14. Standing forward bend at wall
15. Modified Locust
16. Sphinx
17. Cobra
18. Down Dog
19. Bridge
20. Extended leg stretch #1
21. Knees together twist
22. Supported Bridge
23. Knees to chest
24. Reclined cobbler

Closing Relaxation	5 minutes

- Savasana
- Use this time to guide participants through a less structure relaxation period. Assist participants to enter a deeper state of relaxation. Give participants specific guidance on how to do deep relaxation. Begin to allow more silence to enter the closing relaxation. Allow participants the time and space in the silence to enter a deeper state of relaxation.

Closing	2 minutes

- Remember to say good bye to everyone *by name*, making eye contact.
- Remind participants to enjoy home practice this week and to bring back weekly logs next week.

Segment 3: Engaging Your Power
Week 7

Check In With Participants	5 minutes

- Query participants about how they are doing this week.
- Notice who might need modifications and extra support with the slightly more challenging postures in this lesson.

*Lesson Introduction and Yoga Philosophy	3 minutes

Study and Awareness
- Self-awareness through self-examination, reflection, and the study of inspiring people and writings.
- When doing a pose, reflect on yourself in the pose. See if you are straining or relaxed. Make adjustments and changes as needed. With awareness, you can work toward changing old habits that are no longer healthy and cultivate new healthier behaviors.
- Know which poses help you feel better, and which poses do not.

*Relaxation	3 minutes

- Prepare the body for relaxation as described on pg. 86
- Use the menu of relaxation exercises listed on pg. 88 to guide participants or incorporate a relaxation exercise from your own practice. Include a variety of relaxation exercises throughout the 12 weeks of the study.

*Breathing Exercise	4 minutes

"Lengthening each Inhale and Exhale"
1. Review normal breath awareness.
2. Inhale normally through the nose.
3. Exhale slowly and steadily until the lungs feel empty.
4. Discuss how lengthening the exhale helps to relax us.

Yoga Postures	53 minutes

1. Knees to chest
2. Knees together twist
3. Pelvic tilt
4. Toe taps -pelvic tilt with feet off floor
5. Cat/cow
6. Table Top standing with leg extended, hands on chair seat – lift leg up and down slowly
7. Mountain
8. Shoulder openers 1, 2, & 3
9. Crescent Moon
10. Chair
11. Wall Dog
12. Triangle at wall

13. Warrior at wall
14. Mountain, lift one leg, balance at wall
15. Standing forward bend with hands on chair seat or blocks, back heel at wall
16. Seated chair twist
17. Locust
18. Down Dog
19. Child's pose
20. Bridge
21. Extended leg stretch #1 & 2
22. Knees together twist
23. Supported Bridge
24. Knees to chest
25. Reclined Cobbler

Closing Relaxation	5 minutes

- Savasana
- Use this time to guide participants through a less structure relaxation period. Assist participants to enter a deeper state of relaxation. Give participants specific guidance on how to do deep relaxation. Begin to allow more silence to enter the closing relaxation. Allow participants the time and space in the silence to enter a deeper state of relaxation.

Closing	2 minutes

- Remind participants to allow their bodies to lead them to the postures that feel good to their bodies during home practice.
- Remind them to bring weekly home practice logs as well as fill out their Cost Diary at home.

Segment 3: Engaging Your Power

Week 8

Check In With Participants	5 minutes

- Ask participants about how following their bodies to a yoga pose felt last week.

*Lesson Introduction and Yoga Philosophy	3 minutes

Acceptance/Letting Go

- Accepting what is: we have no way of knowing what is a "blessing" or what is a "curse"
- Accepting ourselves as we are in this moment
- Letting go of anything disturbing your peace of mind in the present moment.
- Serenity prayer
- Each pose is a practice of acceptance and letting go.

*Relaxation	3 minutes

- Prepare the body for relaxation as described on pg. 86
- Use the menu of relaxation exercises listed on pg. 88 to guide participants or incorporate a relaxation exercise from your own practice. Include a variety of relaxation exercises throughout the 12 weeks of the study.

*Breathing Exercise	4 minutes

- Introduce "Lengthening each Inhale and Exhale"
  1. Relax with normal breathing
  2. Exhale fully
  3. Breathe in until you feel full, letting your ribs lift and spread as you breathe in.
     a. Notice that there is a slight natural pause at the end of an inhale before new exhale begins.
  4. Breathe out slowly, deeply, and steadily, without letting all the air out at once. Exhale gradually until you feel empty, maintaining the fullness of the lungs as long as possible. Notice that there is a slight natural pause at the end of the exhale before a new inhale begins.
  5. After several cycles of slow, deep, and steady breaths, begin to lengthen the breath by counting each inhale and exhale. Inhale, slowly counting, 1… 2… 3. Pause. Exhaling slowly counting 1… 2… 3. Pause. Have the participants take a normal breath in between each slow breath. Next, inhale counting slowly, 1, 2, 3, 4, pause, exhale, 1, 2, 3, 4, pause. Normal breath. Have participants choose to either repeat inhaling and exhaling for the count of 4, return to a count of 3, or lengthening to a count of 5. It is very important that the breath remains comfortable, never forcing or pushing it.

Yoga Postures	53 minutes

1. Knees to chest
2. Knees together twist

3. Pelvic tilt
4. Toe taps -pelvic tilt with feet off floor
5. Cat/cow
6. Table Top standing with leg extended, hands on chair seat – lift leg up and down slowly
7. Mountain
8. Shoulder openers 1, 2, & 3
9. Crescent Moon
10. Chair
11. Wall Dog
12. Triangle at wall
13. Warrior at wall
14. Mountain, lift one leg, balance at wall
15. Standing forward bend with hands on chair seat or blocks, back heel at wall
16. Seated chair twist
17. Locust
18. Down Dog
19. Child's pose
20. Bridge
21. Extended leg stretch #1 & 2
22. Knees together twist
23. Supported Bridge
24. Knees to chest
25. Reclined Cobbler

Closing Relaxation	5 minutes

- Savasana
- Use this time to guide participants through a less structure relaxation period. Assist participants to enter a deeper state of relaxation. Give participants specific guidance on how to do deep relaxation. Begin to allow more silence to enter the closing relaxation. Allow participants the time and space in the silence to enter a deeper state of relaxation.

Closing	2 minutes

- Remind participants to continue home practice, to bring weekly logs.

Segment 3: Engaging Your Power

Week 9

Check In With Participants	5 minutes

- Query participants about how they are feeling about their progress with yoga and their back pain.

*Lesson Introduction and Yoga Philosophy	3 minutes

Moderation

- Being in control of our desires; not having more or seeking more than we need.
- Aiming for mental and physical balance by practicing moderation in sleep, work, rest, leisure, food, exercise, and relationships.
- Better to do 10-15 minutes of yoga each day than a lot of yoga every once in awhile.

*Relaxation	3 minutes

- Prepare the body for relaxation as described on pg. 86
- Use the menu of relaxation exercises listed on pg. 88 to guide participants or incorporate a relaxation exercise from your own practice. Include a variety of relaxation exercises throughout the 12 weeks of the study.

*Breathing Exercise	4 minutes

Review "Lengthening each Inhale and Exhale," description found on pg. 83.

1. Relax with normal breathing
2. Exhale fully
3. Breathe in until you feel full, letting your ribs lift and spread as you breathe in. Notice that there is a slight natural pause at the end of an inhale before new exhale begins.
4. Breathe out slowly, deeply, and steadily, without letting all the air out at once. Exhale gradually until you feel empty, maintaining the fullness of the lungs as long as possible. Notice that there is a slight natural pause at the end of the exhale before a new inhale begins.
5. After several cycles of slow, deep, and steady breaths, begin to lengthen the breath by counting each inhale and exhale. Inhale, slowly counting, 1, 2, 3. Pause, exhaling slowly counting 1, 2, 3, pause. Have the participants take a natural breath in between each slow breath. Next, inhale counting slowly, 1, 2, 3, 4, pause, exhale, 1, 2, 3, 4, pause. Normal breath. Have participants choose to either repeat inhaling and exhaling for the count of 4, return to a count of 3, or lengthening to a count of 5. It is very important that the breath remains comfortable, never forcing or pushing it.

Yoga Postures	53 minutes

1. Knees to chest
2. Knees together twist
3. Pelvic tilt
4. Toe taps -pelvic tilt with feet off floor
5. Cat/cow

6. Table Top standing with leg extended, hands on chair seat – lift leg up and down slowly
7. Mountain
8. Shoulder openers 1, 2, & 3
9. Crescent Moon
10. Chair
11. Wall Dog
12. Triangle at wall
13. Warrior at wall
14. Mountain, lift one leg, balance at wall
15. Standing forward bend with hands on chair seat or blocks, back heel at wall
16. Seated chair twist
17. Locust
18. Down Dog
19. Child's pose
20. Bridge
21. Extended leg stretch #1 & 2
22. Knees together twist
23. Supported Bridge
24. Knees to chest
25. Reclined Cobbler

Closing Relaxation	5 minutes

- Savasana
- Use this time to guide participants through a less structure relaxation period. Assist participants to enter a deeper state of relaxation. Give participants specific guidance on how to do deep relaxation. Begin to allow more silence to enter the closing relaxation. Allow participants the time and space in the silence to enter a deeper state of relaxation.

Closing	2 minutes

- Remind participants to warm up at home before doing more energetic postures.
- Reiterate the importance of home practice and bringing necessary forms to class.

Segment 4: Bringing it Home

Week 10

Check In With Participants	5 minutes

- Ask participants about how their backs are feeling.

*Lesson Introduction and Yoga Philosophy	3 minutes

Balancing Effort and Ease

- In each pose strive for a balance of effort and ease, of doing and being.
- Life is a balancing act between not being too active or too passive.
- Through our yoga practice we are trying to balance our energy to be more calm, alert, light, and peaceful.

*Relaxation	3 minutes

- Prepare the body for relaxation as described on pg. 86
- Use the menu of relaxation exercises listed on pg. 88 to guide participants or incorporate a relaxation exercise from your own practice. Include a variety of relaxation exercises throughout the 12 weeks of the study.

*Breathing Exercise	4 minutes

Introduce "Ocean Breath" (Ujjayi Breath)

1. Inhales are made with a sibilant sound "sss" and exhales are made with an aspirate "hhh."
2. Exhaling through the nose, slightly close or contract the back of the throat to make a quiet "hhh" sound. Keep the back of the throat contracted while inhaling to quietly make the "sss" sound. To learn to make the sound, start with an exhale through the mouth. Try using one or several of these images to teach the sound:
    - Breathe out as you would to fog a mirror
    - Breathe like when you whisper
    - Breathe as you would if you were trying to see your breath on a cold day.
3. If the sound isn't coming, let it go. It will come on its own with practice.
4. Listening to the sound of the breath helps us stay focused.
5. Contracting the throat like this also allows you to slow the breath which deepens the relaxation response.

Yoga Postures	53 minutes

1. Knees to chest
2. Knees together twist
3. Extended leg stretch, #1 & 2
4. Toe taps -pelvic tilt with feet off floor
5. Table Top with leg extended – lift leg up and down slowly 3x's then hold for 3 breaths
6. Child's pose
7. Mountain

Segment 4: Bringing it Home

Week 10

8. Shoulder openers 1, 2, & 3
9. Sun salutations
10. Wall Dog
11. Triangle – back heel at wall
12. Warrior – back heel at wall
13. Standing forward bend – hand on chair, back heel at wall
14. Baby Dancer
15. Seated chair twist
16. Child's pose
17. Spinal rocks
18. Knees to chest
19. Extended leg stretch
20. Knees together twist
21. Reclined Chest opener
22. Supported Bridge
23. Reclined Cobbler

Closing Relaxation	5 minutes

- Savasana
- Use this time to guide participants through a less structure relaxation period. Assist participants to enter a deeper state of relaxation. Give participants specific guidance on how to do deep relaxation. Begin to allow more silence to enter the closing relaxation. Allow participants the time and space in the silence to enter a deeper state of relaxation.

Closing	2 minutes

- Encourage participants to take the feelings of ease and calm they find in class into other areas of their lives. Remind them of bringing Weekly Home Practice logs and filling out Cost Diaries at home.

Segment 4: Bringing it Home

Week 11

Check In With Participants	5 minutes

- Query participants about questions they may have about yoga and their yoga practice.

*Lesson Introduction and Yoga Philosophy	3 minutes

You are what you practice

- What you practice will determine what kind of person you are.
- The body is like a garden—practice cultivating what you would like to have more of.
- Practice friendliness and loving kindness towards yourself and others instead of anger and hatred.
- Practice compassion instead of judgment
- Practice joy for others' successes instead of jealousy and envy
- Practice forgiveness and acceptance of your own shortcomings and those of others.
- Practice yoga for a healthy back.

*Relaxation	3 minutes

- Prepare the body for relaxation as described on pg. 86
- Use the menu of relaxation exercises listed on pg. 88 to guide participants or incorporate a relaxation exercise from your own practice. Include a variety of relaxation exercises throughout the 12 weeks of the study.

*Breathing Exercise	4 minutes

Review "Ocean Breath" (Ujjayi Breath)

- Inhales are made with a sibilant sound "sss" and exhales are made with an aspirate "hhh."

1. Exhaling through the nose, slightly close or contract the back of the throat to make a quiet "hhh" sound. Keep the back of the throat contracted while inhaling to quietly make the "sss" sound. To learn to make the sound, start with an exhale through the mouth. Try using one or several of these images to teach the sound:
2. Breathe out as you would to fog a mirror
3. Breathe like when you whisper
4. Breathe as you would if you were trying to see your breath on a cold day.
5. Breathe so it sounds like a quieter Darth Vader.
6. If the sound isn't coming, let it go. It will come on its own with practice.
7. Listening to the sound of the breath helps us stay focused and also gives information about its quality, rhythm and pace.

Yoga Postures	53 minutes

1. Knees to chest
2. Knees together twist
3. Extended leg stretch, #1 & 2
4. Toe taps -pelvic tilt with feet off floor

Segment 4: Bringing it Home

Week 11

5. Table Top with leg extended – lift leg up and down slowly 3x's then hold for 3 breaths
6. Child's pose
7. Mountain
8. Shoulder openers 1, 2, & 3
9. Sun salutations
10. Wall Dog
11. Triangle – back heel at wall
12. Warrior – back heel at wall
13. Standing forward bend – hand on chair, back heel at wall
14. Baby Dancer
15. Seated chair twist
16. Child's pose
17. Spinal rocks
18. Knees to chest
19. Extended leg stretch
20. Knees together twist
21. Reclined Chest opener
22. Supported Bridge
23. Reclined Cobbler

Closing Relaxation	5 minutes

- Savasana
- Use this time to guide participants through a less structure relaxation period. Assist participants to enter a deeper state of relaxation. Give participants specific guidance on how to do deep relaxation. Begin to allow more silence to enter the closing relaxation. Allow participants the time and space in the silence to enter a deeper state of relaxation.

Closing	2 minutes

- Let participants know that next week is a data collection week and that they will be receiving reminder phone calls from study staff with instructions.

Segment 4: Bringing it Home

Week 12

Check In With Participants	5 minutes

- Share with participants how enjoyable it was teaching and watching their progress.
- Emphasize that this is <u>not</u> the end of the study.

*Lesson Introduction and Yoga Philosophy	3 minutes

Community

- We are all connected. We are all human beings. We all live on the same earth, receive warmth from the same sun, breathe the same air, drink the same water.
- We have formed a community through our practice of yoga.
- Allow the community to help support your yoga practice.

*Relaxation	3 minutes

- Prepare the body for relaxation as described on pg. 86
- Use the menu of relaxation exercises listed on pg. 88 to guide participants or incorporate a relaxation exercise from your own practice. Include a variety of relaxation exercises throughout the 12 weeks of the study.

*Breathing Exercise	4 minutes

Review "Ocean Breath" (Ujjayi Breath)

- Inhales are made with a sibilant sound "sss" and exhales are made with an aspirate "hhh."

1. Exhaling through the nose, slightly close or contract the back of the throat to make a quiet "hhh" sound. Keep the back of the throat contracted while inhaling to quietly make the "sss" sound. To learn to make the sound, start with an exhale through the mouth. Try using one or several of these images to teach the sound:
2. Breathe out as you would to fog a mirror
3. Breathe like when you whisper
4. Breathe as you would if you were trying to see your breath on a cold day.
5. Breathe so it sounds like a quieter Darth Vader.
6. If the sound isn't coming, let it go. It will come on its own with practice.
7. Listening to the sound of the breath helps us stay focused and also gives information about its quality, rhythm and pace.

Yoga Postures	53 minutes

1. Knees to chest
2. Knees together twist
3. Toe taps -pelvic tilt with feet off floor
4. Extended leg straight up with belt, moving leg side to side
5. Table Top with leg extended – lift leg up and down slowly 3x's then hold for 3 breaths
6. Child's pose

7. Mountain
8. Shoulder openers 1, 2, & 3
9. Sun Salutations
10. Triangle
11. Warrior
12. Standing forward bend – hand on chair or blocks
13. Baby Dancer
14. Child's pose
15. Spinal rocks
16. Knees to chest
17. Extended leg stretch
18. Knees together twist
19. Reclined Chest opener
20. Supported Bridge
21. Reclined Cobbler

Closing Relaxation	5 minutes

- Savasana
- Use this time to guide participants through a less structure relaxation period. Assist participants to enter a deeper state of relaxation. Give participants specific guidance on how to do deep relaxation. Begin to allow more silence to enter the closing relaxation. Allow participants the time and space in the silence to enter a deeper state of relaxation.

Closing	2 minutes

- Have each participant share what the yoga has done for them, and what they are taking home from the lessons. Thank everyone and tell them how great they were.

Guidelines for Teaching Yoga Poses

Baby Dancer Pose…………………………………………………………………….. 51
Bridge Pose…………………………………………………………………………..... 52
Cat/Cow Pose……………………………………………………………………...... 53
Chair Pose……………………………………………………………………………… 54
Chair Twist Pose: Seated………………………………………………………… 55
Chair Twist Pose: Standing…………………………………………………….. 56
Child's Pose………………………………………………………………………..... 57
Cobra Pose…………………………………………………………………………….. 58
Crescent Moon Pose……………………………………………………………….. 59
Downward Dog Pose……………………………………………………………….. 60
Extended Leg Pose………………………………………………………………… 61
Flat Back Forward Bend…………………………………………………………. 62
Forward Bend Pose…………………………………………………………………63
Knees to Chest Pose……………………………………………………………….. 64
Knees Together Twist………………………………………………………………65
Locust Pose…………………………………………………………………………… 66
Modified Chair Pose……………………………………………………………… 67
Mountain Pose……………………………………………………………………… 68
Mountain Pose with One Leg Lifted…………………………………………….. 69
Pelvic Tilt Pose………………………………………………………………………70
Reclined Chest Opener Pose……………………………………………………. 71
Reclining Cobbler Pose…………………………………………………………. 72
Shoulder Opener Pose…………………………………………………………….. 73
Sphinx Pose…………………………………………………………………………… 74
Standing Forward Bend with Wall Assist Pose……………………………… 75
Sun Salutations…………………………………………………………………….. 76
Supported Bridge Pose…………………………………………………………. 77
Table Top with Leg Extended Pose……………………………………………. 78
Triangle Pose………………………………………………………………………. 79
Toe Taps…………………………………………………………………………….. 80
Triangle at Wall Pose……………………………………………………………. 81
Wall Dog Pose……………………………………………………………………….. 82
Warrior I Pose………………………………………………………………………… 83
Warrior I at Wall Pose…………………………………………………………….. 84

Baby Dancer Pose

Description: Standing on the left leg, holding the right foot behind you with your hand while extending your left arm forward.

How to teach: Stand in Mountain Pose (pg.68) facing the left side of your body to the wall with your left hand on the wall. Find a steady point in front of you to focus on. This will help you balance. Gradually shift your weight to left the leg. Inhale and bend right knee so the right foot is behind you. Hold the right foot or ankle with the right hand. Stand firmly on the left leg, keeping it straight. Take a moment to balance yourself so that knees are in alignment with your hips. Hold here and breathe into the stretch from your abdominal muscles to the front of the thigh. If steady, inhale and raise the left hand up over head still on the wall. Slowly release bent leg to the floor. Come back to Mountain Pose. Repeat on opposite side.

Modifications:

1. If you have difficulty reaching back far enough to hold your foot due to tight shoulders or quads, use a yoga strap to help. For example, wrap the strap around the right foot and hold the other end of the strap with the right hand.
2. Chair: Sit on edge of chair, take one foot back to the side of the chair and let the knee drop down to face the floor. If able, hold ankle or place a belt around ankle.

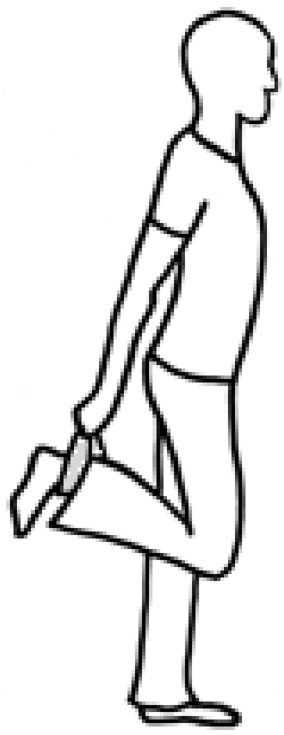

Bridge Pose

Description: Lying on back with knees bent, feet flat on mat, lift back up to make a bridge shape.

How to teach: Lying on back with knees bent, feet flat on mat and hip distance apart, walk feet in toward hips as far as possible. Keep thighs and feet parallel. Reach hands toward feet, grabbing either feet or sides of mat. Turn upper arms and shoulders down toward floor and tuck shoulders in toward each other. Inhale, press feet into the floor, lifting tailbone up. Keep lifting from backs of thighs (not from the lower back) until back is up off floor. Press arms down into floor and lift upper back up, bring chest toward chin. Exhale, relax neck. Slowly roll down back to the mat starting with the upper back, one vertebra at a time. Keep tailbone lifted while rolling down until body is all the way down. Rest back on the floor.

If the pose causes back pain, keep back on floor and do gentle pelvic tilts.

Modifications:

1. If pain in knees, walk feet further away from hips.
2. Place a block between knees or a belt around thighs to keep thighs parallel.
3. Chair: With the hands on chair seat or holding the arms of the chair, lean back and lift the hips up off the chair, making sure not to lift from the lower back but instead lift from backs of thighs. If you cannot put weight on hands or arms, do Pelvic Tilt in chair.

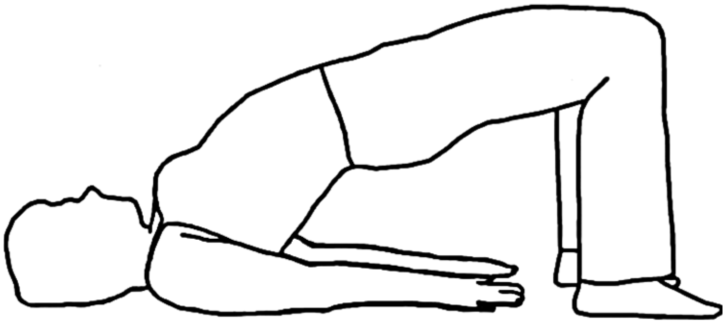

Cat/Cow Pose

Description: Table position with hands and knees on mat, lifting and lowering back into concave and convex shape.

How to teach:
Cat: Begin on all fours in table position. Place hands under shoulders and hips over knees. Press hands into mat. Exhale, tuck chin toward chest, tuck tailbone down toward floor, and round spine up, lifting abdomen up toward spine.

Cow: Inhale, lift top of the head, keeping back of neck lengthened. Drop the back down toward mat, lifting tailbone. Keep abdomen lifted up into body to prevent overarching in lower back.

To prevent injury, caution participants to not overextend the neck. Encourage them to keep the spine of the neck in line with the spine of the upper back. Also encourage participants to look forward or slightly down.

Modifications:

1. If flat hands cause wrist discomfort, make a fist with both hands resting weight on knuckles with thumbs facing down.
2. If participants need more cushioning under knees or hands, roll the sides of mat under knees or the top of mat under hands.
3. Chair: Sit on chair with feet under knees and knees hip width apart. Exhale, gently round the spine and tuck the chin toward the chest. Inhale and straighten up. Exhale, gently lift head and chest up, slightly arching the back. Repeat several times. Rest, sitting straight up.

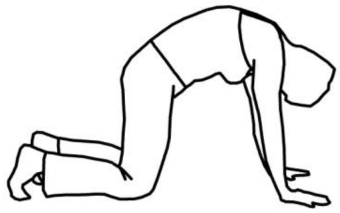

Cat

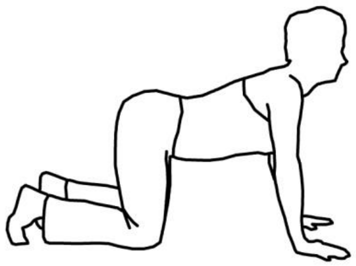

Cow

Chair Pose

Description: Standing with knees and hips bent, arms over head, and lowering the hips down as if going to sit in a chair.

How to teach: While in Mountain Pose (pg. 68), place hands on hips. Exhale, bend knees over feet while also bending at hips as if about to sit in a chair. Drop tailbone down toward the floor while lifting chest up. Move weight back into heels and draw abdomen in. Inhale, extend arms above the head. Exhale, move legs and hips back slightly to bring weight back on heels. Inhale, straighten legs and bring arms down to come back to Mountain Pose.

Modifications:

1. Keep hands on waist
2. Raise arms out in front to shoulder height
3. Stand with back on wall but feet and knees away from wall so that back is leaning on the wall with the feet and knees together (unless there is knee pain or balance issues, then separate). Bend knees, making sure that knees are pointing over feet and press lower back into wall. Slowly raise arms up over head.
4. Stand with feet hip width apart and 6-8 inches away from and facing the wall. Place your fingertips on the wall at shoulder height. Follow directions for Chair Pose.
5. Sitting on edge of chair with feet under knees. Lean slightly forward, taking body weight onto feet. While remaining seated, raise arms over head, keeping chest and back ribs lifted and tailbone moving down toward chair seat.

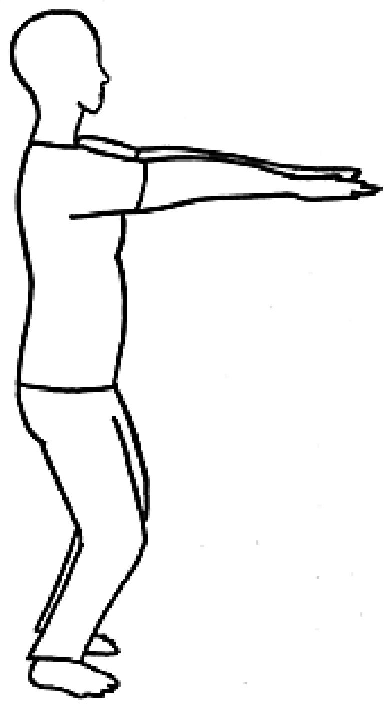

Chair Twist Pose: Seated

Description: Gently twisting while sitting in a chair.

How to teach: In an armless chair: Sit sideways so that the right side is facing the back of chair. Sit on the edge of the seat so that the feet are firmly on the floor hips width apart, thighs parallel. Raise arms up on an inhale and turn right toward the back of the chair on an exhale taking the arms down, hands onto the back of chair. Relax and breathe, gently lifting the spine on an inhale and turning on an exhale. Release hands and turn back to face forward. Repeat, turning to the left.

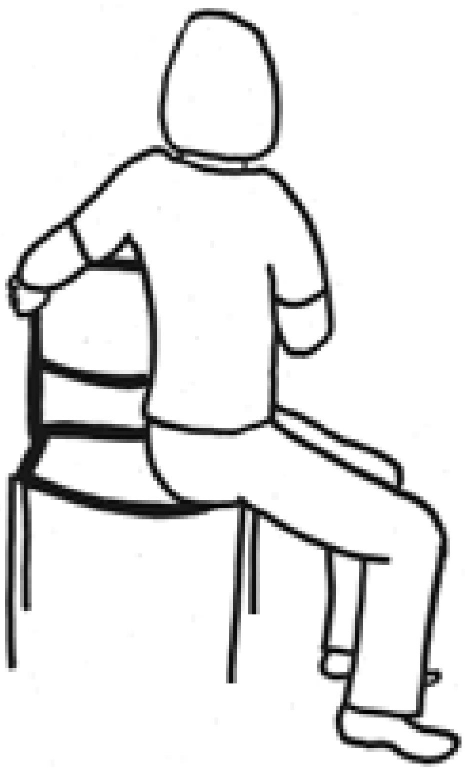

Chair Twist Pose: Standing

Description: Standing sideways to wall with one foot on the chair, hands on wall, twisting toward the wall

How to teach: Standing with the left side of the body against the wall, the left foot on a block on the chair, turn and place the hands on the wall level with the shoulders. Maintain the alignment of the knees, facing toward the feet and the outer knee aligned with the outer hip. Inhale, press the hands slightly down, moving the shoulders down and lengthening the spine upward. Relax in twist, lengthening the spine on the inhale and turning gently on the exhale for three breaths. Turn back to face chair and step foot down. Turn chair and body to face the other side toward the wall and repeat this side.

Modifications:

1. For shorter people, place the foot directly on the chair seat, omitting the block under the foot.
2. With right hand hold left knee in place while turning to stop the knee from turning towards the wall.
3. Place a block between the wall and the outside of the knee

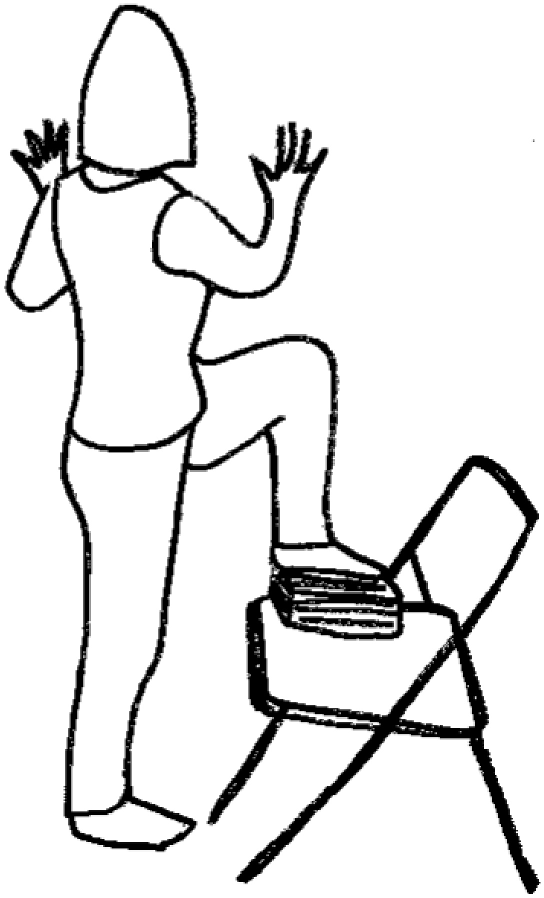

Child's Pose

Description: Kneeling on mat, torso flexed forward to mat, arms on mat close to the side of the body or extended over the head. Forehead on floor.

How to teach: Start by kneeling, then sit comfortably on heels. Take knees apart while keeping toes together. Bend forward bringing head to floor. Extend arms forward on the floor overhead or to the side.

Modifications:

1. If there is knee pain, place a folded blanket behind knees, between the shin and the thigh.
2. If there is ankle pain, place a rolled blanket under ankles.
3. If the feet cramp, keep toes curled under.
4. Chair: Sitting in a chair place feet firmly on floor. Separate legs, aligning feet with knees. With hands on thighs, palms down, slowly bend forward between legs. Hands can stay on thighs or move to the floor or blocks.

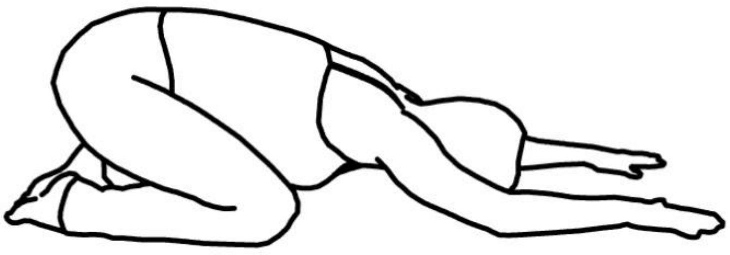

Cobra Pose

Description: Lying prone, lifting head and chest off of floor while gently arching upper back.

How to teach: Lie flat on stomach, with legs slightly wider than hip distance apart. Place tops of feet on the floor. Relax shoulders and put forehead on the mat. Place palms face down at armpit level, with fingers facing forward. Keep elbows bent at side. Inhale, push palms down against the floor and use back muscles to lift head and then chest, slowly, gazing forward. Keep shoulders moving down toward floor. Keep elbows bent. There should be no strain on the lower back. Exhale, as you lower the upper back and head down to return to beginning position.

To prevent injury, caution participants to not overextend the neck. Encourage them to keep the spine of the neck in line with the spine of the upper back. Also encourage participants to look forward or slightly down.

Modifications:

1. Sitting in a chair, place feet firmly on floor with hands on thighs and move shoulders down away from ears. Roll upper arms back to open chest. Squeeze shoulder blades slightly into each other and pull abdomen in toward spine.

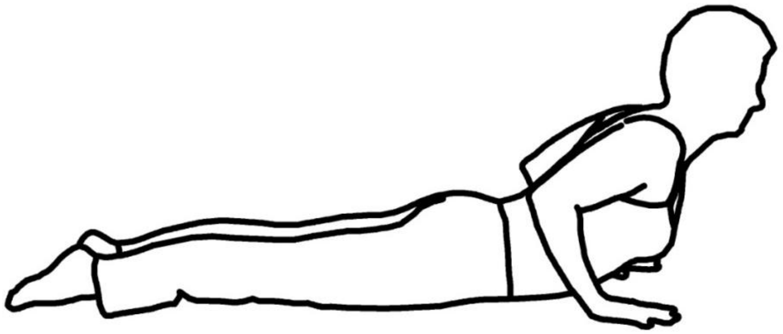

Crescent Moon Pose (Modified)

Description: Standing in Mountain Pose (p. 68), with arms overhead, bend slightly to each side. This pose is sometimes called Half Moon Pose. It is a variation of Mountain Pose.

How to teach: From Mountain Pose, inhale and extend arms up straight up over head. Hold the left wrist with the right hand. Exhale, bend slowly to the right, extending the left arm up and over to the right. Press down through the left foot to lengthen through the entire left side of the body. Inhale, come back up to Mountain. Hold the right wrist with the left hand and exhale, bend to the left.

Modifications:

1. While bending to the right, take left arm up and over head toward the right with the palm facing the floor, keeping the right hand on the waist. Same on the left side.
2. Keep both hands on the waist while bending sideways.
3. Chair: Sit upright in a chair with back straight, inhale and extend arms straight up over head. Hold the left wrist with the right hand. Exhale, bend slowly to the right extending the left arm up and over to the right. Inhale come back up. Hold the right wrist with the left hand, exhale, bend to the left.

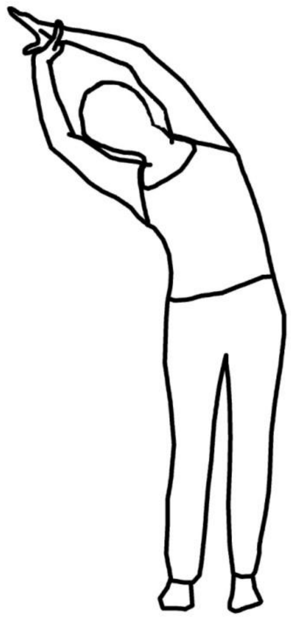

Downward Dog Pose

Description: This pose resembles an inverted V shape from table top.

How to teach: Start in Table Top Pose (p. 78). On hands and knees, walk hands in front of shoulders with hands shoulder distance apart. With knees in line with palms, move knees backwards. Inhale, lift hips up toward ceiling and straighten legs. Spread fingers and press them evenly on the floor. Exhale, stretch arms keeping elbows straight, lengthening arms, torso, and back toward legs. With legs straight, inhale, lift hips higher while pressing heels down toward the floor. Your upper arms should be alongside your ears, not your shoulders. Exhale, come back to Table Top on your hands and knees.

Modifications:

1. Wall dog
2. If hands are slippery, put thumb and index fingers against a wall with palms turned out and slightly wider than shoulders.
3. Place a chair at the wall. Bending over, hold the sides of the chair seat and step back until body is in a "V" shape. Place feet the same distance apart as hands.
4. Chair: Sit on edge of a chair, extend both legs out, hips weigh apart and raise both arms straight up over head, shoulder distance apart.

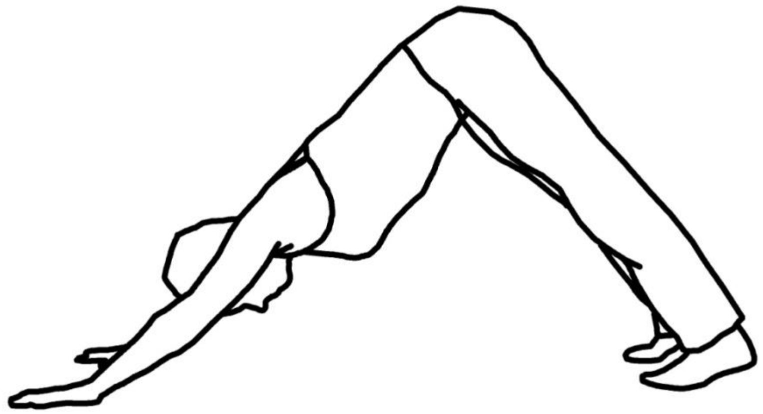

Extended Leg Pose

Description: Lying on back, stretch one leg in the air with belt over foot.

How to teach: With both knees bent, place belt on sole of right foot and straighten leg. Allow shoulders to relax down toward floor and keep tailbone on floor. Press right foot up overhead while pulling arms and shoulders down. Inhale, straighten leg. Take belt in right hand, bring left hand to floor. Exhale, move right leg out to right side and let left knee move out to the left. Inhale, bring leg back up straight. Repeat, moving leg out and up again three times, keeping leg straight and moving with each breath. End with leg straight up. Bend knee and take belt off and repeat on left leg.

Modifications:

1. Bend and straighten leg several times using a belt for support.
2. With leg straightened, circle leg in one direction and then in the other direction several times using a belt for support.
3. Lying on back at wall with feet facing wall, knees bent, place one leg up on wall as straight as possible using the wall for support.
4. Lying on back with feet facing chair seat, knees bent, rest right foot on edge of chair seat. Raise right leg up as straight as possible. Repeat on other side using the chair for support.
5. Chair: Sit on a chair, with legs bent and feet on floor, place one leg straight up onto block or another chair seat (or whatever height will give a comfortable stretch depending on ability).

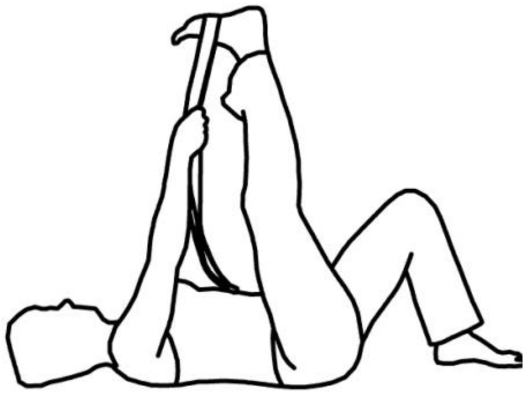

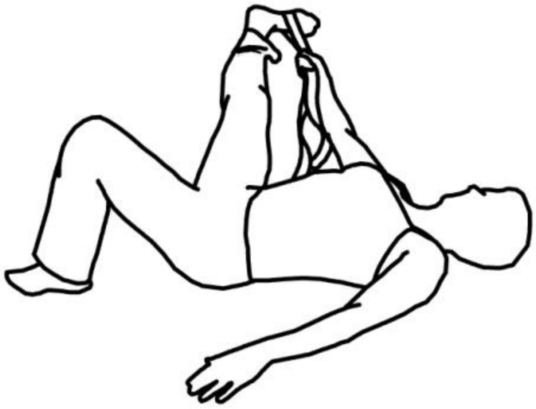

Flat Back Forward Bend

Description: Bending forward from hips with a flat back.

How to teach: From Mountain Pose (p. 68), stand with the feet a little wider that hip-width apart. With the hands on the hips, bend forward from the hips, keeping the back straight (back of the head stays in line with the tailbone). Place the hands under the shoulders on blocks or a chair seat. Keeping the weight balanced evenly in the feet and the legs straight and perpendicular to the floor, straighten the arms and press the hands down onto blocks or chair seat to lift the chest up (keep the back of the neck lengthened). Move the shoulders back toward the waist and the shoulder blades down into the body to feel the back muscles contracting and the front body lengthening.

Forward Bend Pose

Description: Bending forward with straight legs and arms, with the feet a little wider than hip-width apart and the hands on the floor by the feet, or on blocks or a chair under the shoulders.

How to teach: From Flat Back Forward Bend (p. 62), maintain the firmness in the legs as the chest and head move down toward the floor. The hands also come down lower to the floor.

Modifications:

1. Place hands on chair seat and bend one knee at a time to come down onto hands and knees.

Knees to Chest

Description: Alternate bringing one knee toward chest, then bringing both knees to chest.

How to teach: Start by lying on back with both feet on the floor. Inhale, lift one leg up to bring knee into chest. Hold leg around shin or thigh and on an exhale, gently bring knee in toward chest. Repeat twice on each side. Then, bring both knees into chest.

Modifications:

1. Place a belt around shin or thigh if unable to reach with hands.
2. If unable to bring knees to chest with hands or belt, place feet on a wall or chair.
3. Chair: Sitting in chair, raise one knee at a time, holding leg up with hands under thigh or belt.

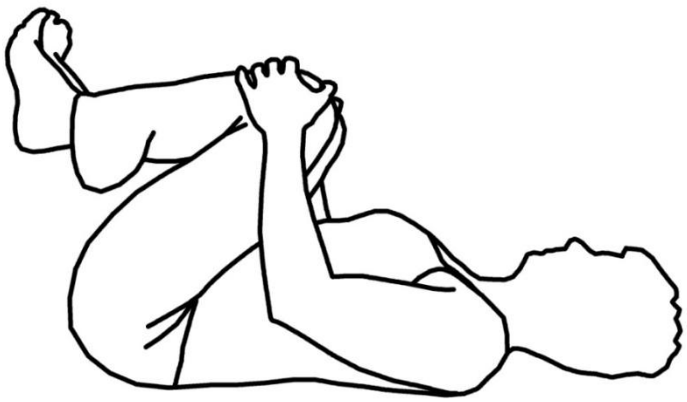

Knees Together Twist

Description: Lie on back with knees bent and feet on floor. Twist knees both to the left, then to the right.

How to teach: Start lying on back with feet on the floor, with knees together and bent. Lay arms out on the floor, palms facing up, and level with shoulders. Exhale, bring both knees down to the left, keeping shoulder down. Inhale, raise knees back to center. On next exhale, bring both knees down to the right, keeping left shoulder down on the floor. Repeat three times, moving with the breath.

Modifications:

1. If opposite shoulder cannot stay down on the floor, place a blanket(s) or block under knees on each side.

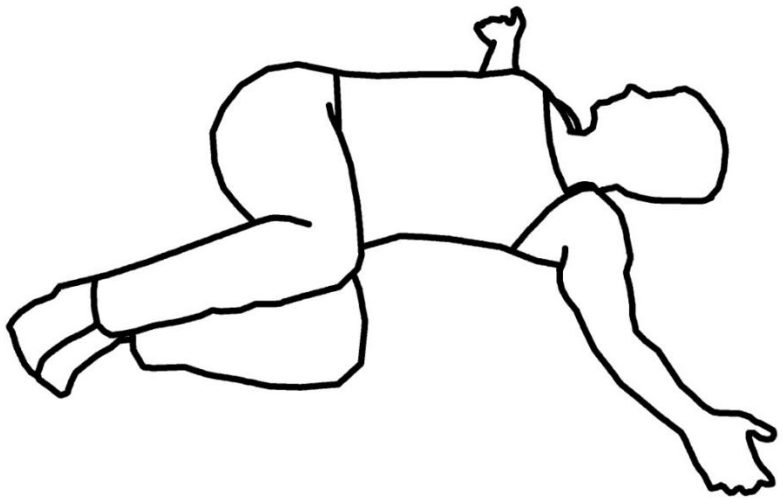

Locust Pose

Description: Lying prone, lifting legs, arms, upper chest, and head off the floor.

How to teach: Lie on belly with chin or forehead on the mat. Bring hands straight back by hips. Point big toes behind and away from you. Inhale and lift head, upper chest, arms and legs off the mat at the same time. Lightly press pubic bone into the mat to create more space in low back. To release, exhale as head, arms and legs come back to the floor.

To prevent injury, caution participants to not overextend the neck. Encourage them to keep the spine of the neck in line with the spine of the upper back. Also encourage participants to look forward or slightly down.

Modifications:

1. Fold your arms in front of you and place head on back of hands. Raise one leg at a time. Then raise both legs together. Lower legs and then with arms down by the sides of body and palms facing up, raise head, chest and arms, while pressing legs down.
2. Stand facing a chair: Bend forward and hold on the seat of a chair. Extend one leg back , inhale, slowly raise the leg straight up off the floor. Exhale, bring it down.
3. Chair: Sitting in a chair, place feet firmly on floor with the arms by the sides and move the shoulders down away from the ears. Roll the upper arms back to open the chest. Squeeze shoulder blades slightly into each other and pull abdomen in towards the spine. Extend arms straight back and behind.

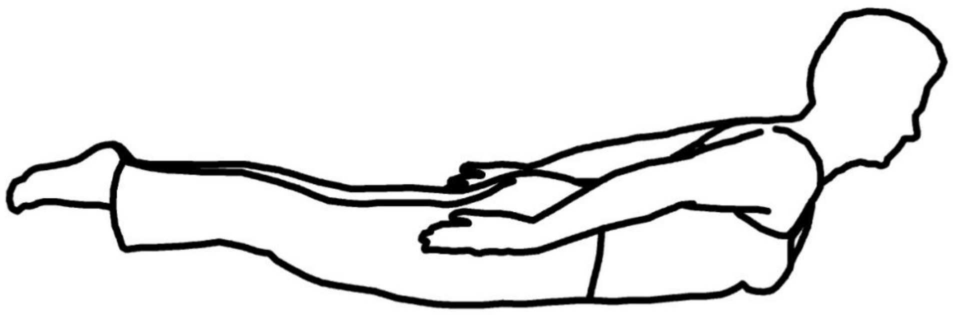

Modified Chair Pose

Description: Standing with knees and hips bent and lowering the hips down as if going to sit in a chair.

How to teach: While in Mountain Pose (p. 68), place hands on hip. Exhale, bend knees over feet while also bending at hips as if about to sit in a chair. Drop tailbone down toward the floor while lifting abdomen and chest up. Move weight back into heels and draw abdomen in. Inhale, raise arms out in front, in line with shoulders. Exhale, move legs and hips back slightly to bring weight back into heels. Inhale, straighten legs and bring arms down coming back to Mountain Pose. Come in and out moving with the breath.

Modifications:

1. Keep hands on waist
2. Stand with back on the wall, but feet and knees away from wall. Participants should now be leaning on the wall with back and with feet and knees together (unless there's knee pain or balance issues). Bend knees, making sure that knees are pointing over feet and press lower back into the wall. Slowly raise arms up over head and towards the wall.
3. Face the wall, stand with feet hip width apart, 6-8 inches from the wall. Place fingertips on the wall at shoulder height. Follow directions for Modified Chair Pose.
4. Chair: Sit on the edge of the chair with feet under knees. Lean slightly forward, allowing yourself to bear some of your weight onto your feet. While remaining seated, raise arms over head, keeping chest lifted and back straight, and tailbone moving down toward the chair seat.

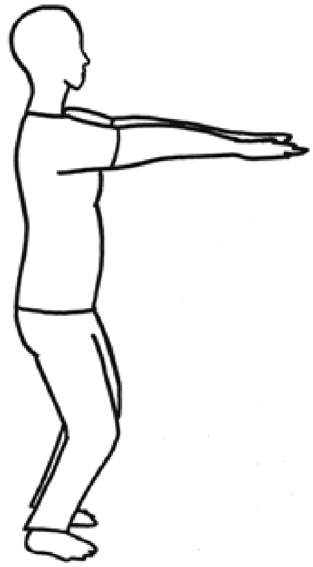

Mountain Pose

Description: Standing upright, tall, firm and balanced aligning body from feet to top of head.

How to teach: Stand upright with feet facing forward and hip width apart, balancing body weight evenly on the feet, from balls of feet to the heels and from inner to outer foot (or "on the four corners of feet, i.e. ball of big toe, ball of the little toe, inner heel and outer heel.) Align thighs and shins over ankles, hips over thighs, shoulders over hips, and ears over shoulders. Bring shoulder blades down into back, while lifting chest up. Lengthen neck with the head and look straight ahead. Extend arms down by the sides of body.

Modifications:

1. Stand with back against wall
2. Lie on floor with legs straight and feet pressing into wall. Arms down by sides, shoulders pressing down toward the floors. Elongate back of the neck.
3. Chair: Sit upright in a chair with back straight. Feel weight on the sitting bones (the two bones at the base of the pelvis) by leaning slightly to the right and the left. Find where the weight is even on both the right and left sitting bone. With hands on hips, slightly tip the hips forward and back, find the center so that the top of the hips are level with the floor. Align the top of the head over the hips so that the ears are over the shoulders, and the shoulders are over the hips. Extend the arms straight down by the side of the torso. Relax the shoulders down and lift the chest up. Look straight ahead with a relaxed face keeping the back of the neck long and the chin parallel to the floor.

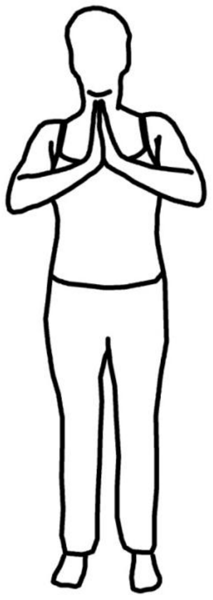

Mountain Pose with One Leg Lifted

Description: Mountain with one leg raise up in front

How to teach: Stand in Mountain pose, shift the weight to the left leg without letting the left hip push out to the side. Spread the left foot and feel the body weight come onto the left foot evenly across the inner and outer parts of the foot, and evenly from the front to the back of the foot. Feel the body weight heavy into the full circle of the heel bone. Pull the thighs up, feeling the muscles of the legs tightening around the thighbone. Standing tall and firmly on the left leg, slowly raise the heel of the right leg up off the floor. If stable, take the foot off the floor, raising the right leg up in front, balancing on the left leg. Repeat other side.

Modifications:

1. With a chair in front facing backward, hold onto the back of chair
2. Hand(s) on wall

Pelvic Tilt Pose

Description: Lying on back with knees bent and feet on floor in line with hips, arms down by sides of torso.

How to teach: Lying on back, bend knees bringing feet in toward lower pelvis, thighs parallel to each other. Extend the arms down toward the feet, palms downward on the mat and relax the shoulders. Inhale, relax maintaining the natural curve of the lower back. Exhale, gently press the lower back into the floor flatting the natural curve and slightly lifting the lower pelvis up toward the ceiling. Inhale, relax and return to the natural curve of the back. Repeat, several times keeping the shoulders and head relaxed. There will be a gentle rocking motion of the pelvis.

Modifications:

1. Use a block between the knees to keep them parallel to each other if the knees are splaying out wider than hips.
2. Stand with back and head against wall, feet away from wall and slightly bent. On exhale, gently press lower back into wall, relax on inhale.
3. Sitting up straight in a chair with feet firmly planted on the floor, exhale, gently press lower back toward the back of the chair, slightly rounding the lower back. On inhale, sit back upright. Repeat several times.

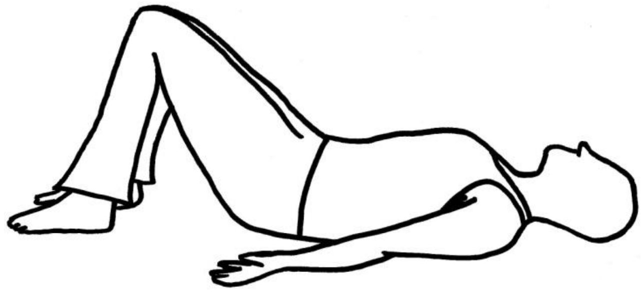

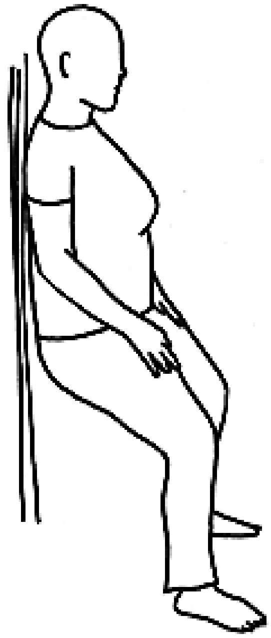

Reclined Chest Opener Pose

Description: lying prone with blanket or block under upper back.

How to teach: Place a narrowly folded blanket horizontally across the mat so that it will be under the upper back (the blanket comes under the bottom of the arms when the arms are stretched out on the floor sideways shoulder height.) Lie down in Savasana with the back of the head on the floor. The shoulders are not on the blanket.

Modifications:

1. If neck is uncomfortable, place a blanket under the head. For more of a chest opening use two blankets or a block. If there is lower back discomfort, bend knees or come out of the pose.

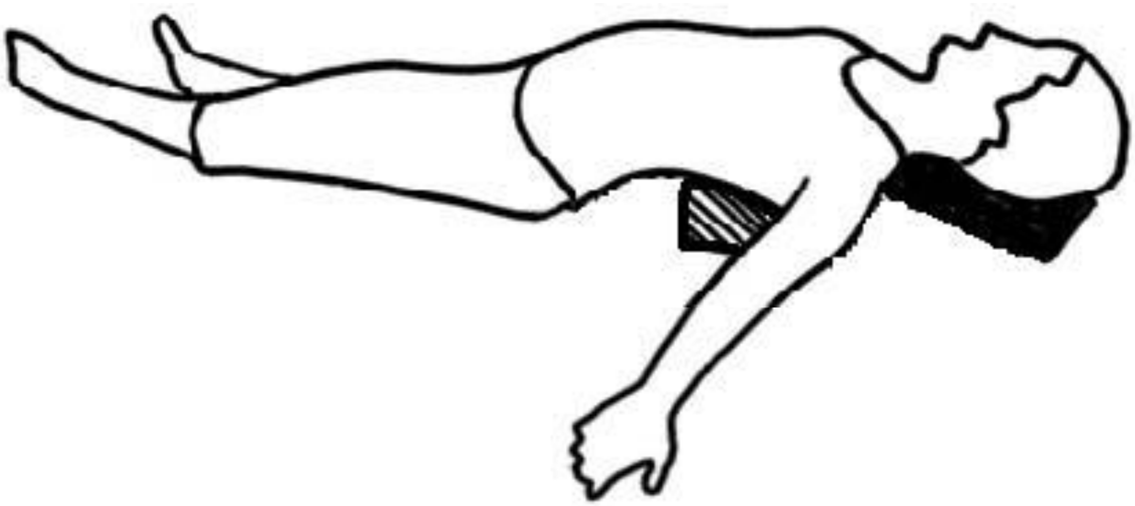

Reclining Cobbler Pose

Description: Lying on back, arms straight and palms up, slightly out to the sides with the palms facing up toward the ceiling, bend knees, place feet together on mat, lower knees away from each other toward mat, bring soles of feet together.

How to teach: Begin by lying on back with knees bent. Bring soles of feet together so knees can open to the sides. Place folded blankets, blocks, or bolsters under outer thighs for support. Make sure the pose is comfortable and there is no strain in the hips or inner thighs. Breathe and relax in posture. To come out, take hands and gently move knees together.

Modifications:

1. For lower back discomfort, fold 1-2 blankets lengthwise to support length of spine from waist to head. Don't have the blankets right up to tailbone, but rather have space from waist to tailbone.
2. If there is back discomfort after coming out of posture, lie flat on the floor and hug both knees to the chest, gently rocking side to side.

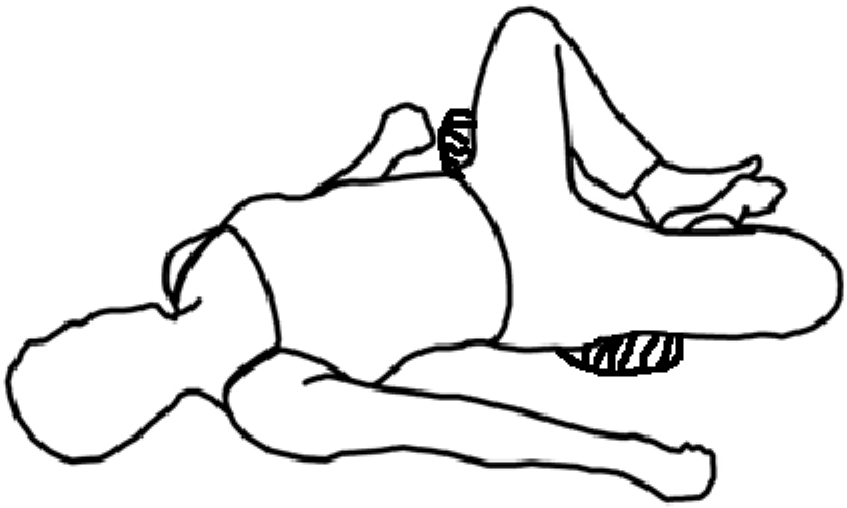

Shoulder Opener Poses

Description: Standing, feet pointing forward and hip distance apart, fingers clasped behind hips, arms extended back without dropping chest.

How to teach: From Mountain Pose (pg.68), clasp hands behind back and slowly raise arms backwards to a comfortable stretch, making sure not to collapse chest. Continue to breathe and draw shoulder blades back toward each other. Slowly lower arms down and release hands.

Modifications:

1. For very tight shoulders, instead of clasping hands together, use a belt to allow a wider distance between hands.
2. Chair: All of the poses can also be completed while sitting in a chair

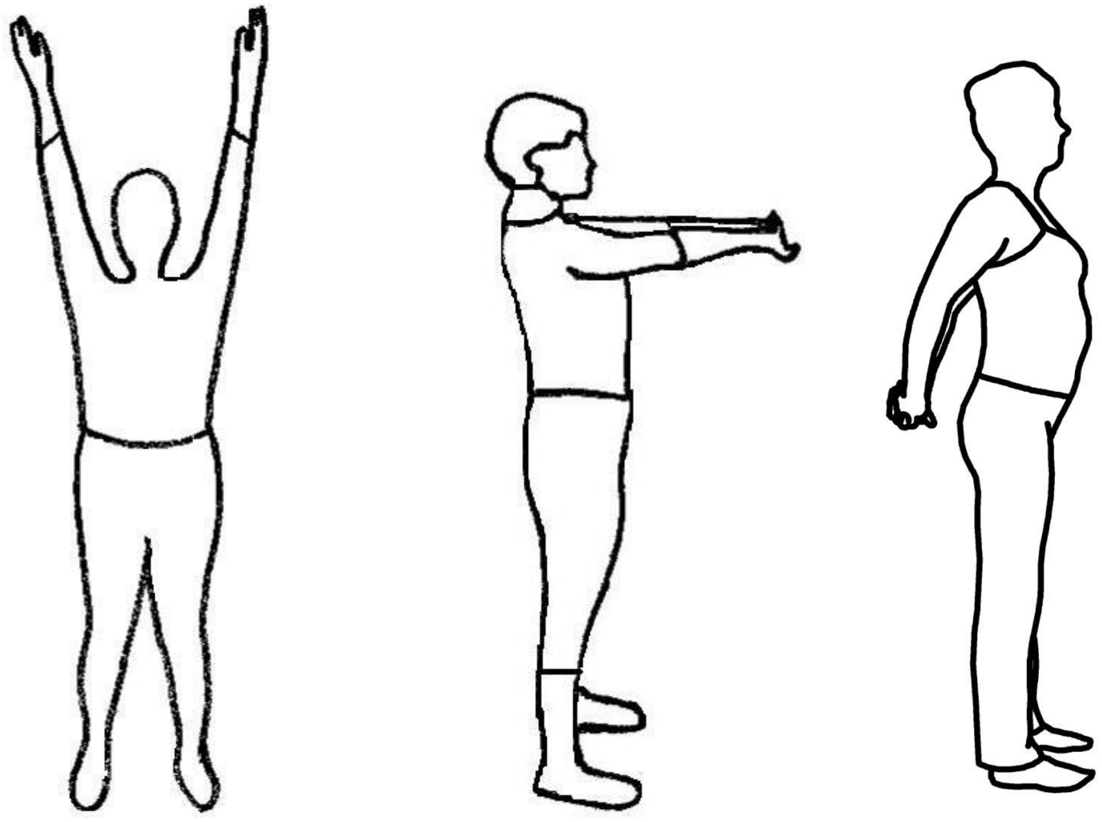

Sphinx Pose

Description: Lying prone, rest on forearms, stomach, and legs.

How to teach: Lying on stomach place elbows shoulder distance apart, slightly in front of shoulders, fingers pointing straight ahead. Press forearms firmly down while lifting head and chest up. While inhaling, move shoulders down and pull them back, bringing chest forward. Exhale, release to the floor.

To prevent injury, caution participants to not overextend the neck. Encourage them to keep the spine of the neck in line with the spine of the upper back. Also encourage participants to look forward or slightly down.

Modifications:

1. Chair: Sitting in a chair with feet firmly on the floor. With hands on thighs allow shoulders to drop down away from ears. Roll upper arms back to open chest. Move shoulder blades slightly towards each other and pull abdominal muscles in. Lift head and chest slightly up.

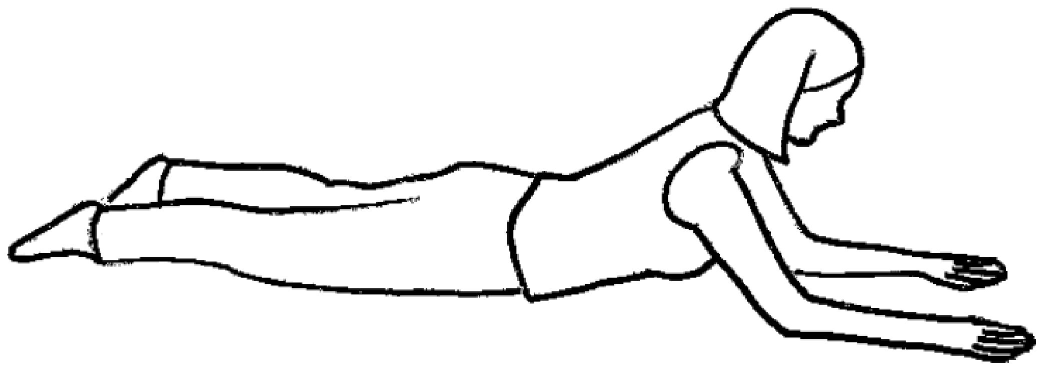

Standing Forward Bend at Wall Pose

Description: Standing facing the wall, bend forward with one leg forward and hands on the wall.

How to teach: From Wall Dog Pose (pg.82), step right leg forward toward the wall (about one foot forward). Step left foot back away from the wall (about 3 feet) with back foot slightly turned out. Keep back as parallel to the floor as possible with both legs straight. Some participants may not be able to reach very parallel, encourage participants not to push themselves too far. Try to keep the hips level side to side.

Modifications:

1. Place hands on a chair or table.
2. Chair: Sitting in a chair, extend one leg straight forward with the heel on the floor and the other leg bent with the foot on the floor. Exhale, bend forward at the hips until there is a feeling of a stretch in the back of the leg. Inhale sit back up. Repeat and hold for 3 breaths. Repeat on other side.

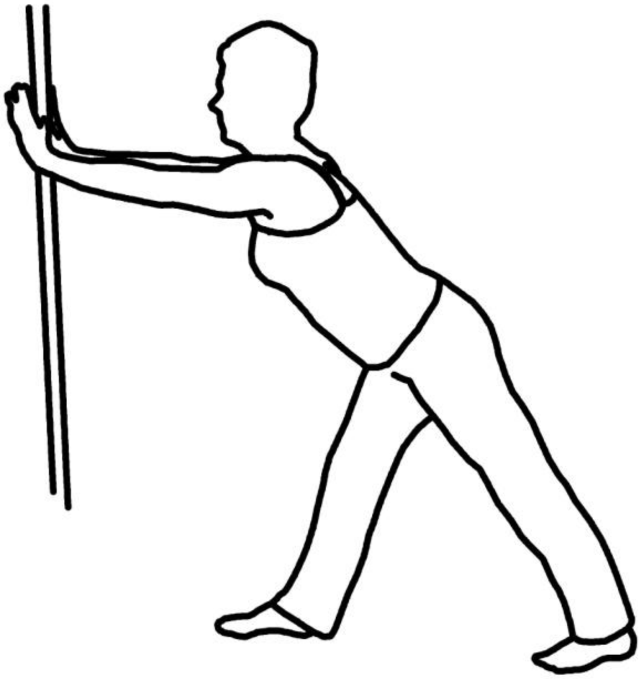

Sun Salutations

Description: A series of postures done in a sequence from Mountain pose to Downward Facing Dog pose and back to Mountain pose.

How to teach:

1. Mountain
2. Mountain with arms up – inhale
3. Hands to hips – exhale to
4. Flat back forward bend - head up, hands on blocks, or chair – inhale
5. Forward bend – head down, hands on floor, blocks, or chair – exhale
6. Bend knees, place hands on floor and step back to Downward Facing Dog pose
7. Downward Dog – inhale, hold 3 breaths
8. Walk back in toward hands to Forward bend pose
9. Forward bend – head down, hands on floor, blocks, or chair - exhale
10. Flat back forward bend - head up, hands on legs, blocks, or chair – inhale, exhale
11. Hands to hips, inhale come up to mountain
12. Mountain with arms up - inhale
13. Mountain - exhale

Supported Bridge

Description: Lying on back with knees bent and a block under the pelvis making a bridge shape with the body.

How to teach: Lying on back with knees bent, feet flat on mat and hip distance apart, walk feet in toward hips as far as possible. Keep thighs and feet parallel. Reach hands toward feet, grabbing either feet or sides of mat. Turn upper arms and shoulders down toward floor and tuck shoulders in toward each other. Inhale, press feet into the floor, lifting tailbone up. Keep lifting from backs of thighs (not from the lower back) until back is up off floor. Place block on its lowest side under tailbone. Rest the back of hips down onto the block. Maintain the feet hips distance apart and thighs parallel to each other. With the arms by the sides of the body, bend and press down on the elbows to lift the chest up, without lifting the head or shoulders. Then relax the arms on the floor slightly out to the side. The back of the neck remains long and relaxed.

Modifications: If able, lift hips up higher and place block on the next height under tailbone. To raise the hips higher, raise the heels up and walk the feet into the hips as close as possible, re-lift the hips. Walk the feet out until the feet are flat on the floor, thighs parallel to each other.

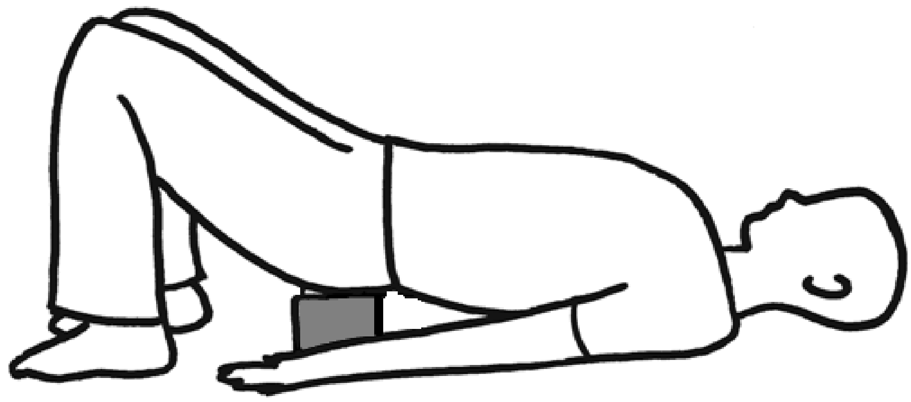

Table Top with Leg Extended Pose

Description: On hands and knees, extend one leg at a time behind you.

How to teach: Begin on hands and knees. Place hands directly under shoulders and knees directly under hips. Back is flat and level like a table. Inhale, extend right leg behind with leg straight and foot on the floor. Exhale, press back through heel. Lift abdomen up into body. Inhale, bring right knee back to return to all fours, and repeat on the other side by stretching left leg behind.

To prevent injury, caution participants to not overextend the neck. Encourage them to keep the spine of the neck in line with the spine of the upper back. Also encourage participants to look forward or slightly down.

Modifications:

1. If there is wrist discomfort, make a fist with both hands resting weight on knuckles with thumbs facing down.
2. If participants need more cushioning under knees or hands, roll sides of mat under the knees or top of the mat under hands.
3. Rest your forearms on the floor rather than your hands
4. Standing with hands on the back of a chair (bending forward) or against the wall, extend one leg at a time back with the toes down on the floor.
5. Chair: Sit on the edge of a chair, extend one leg out in front with the heel on the floor. Press through the heel.

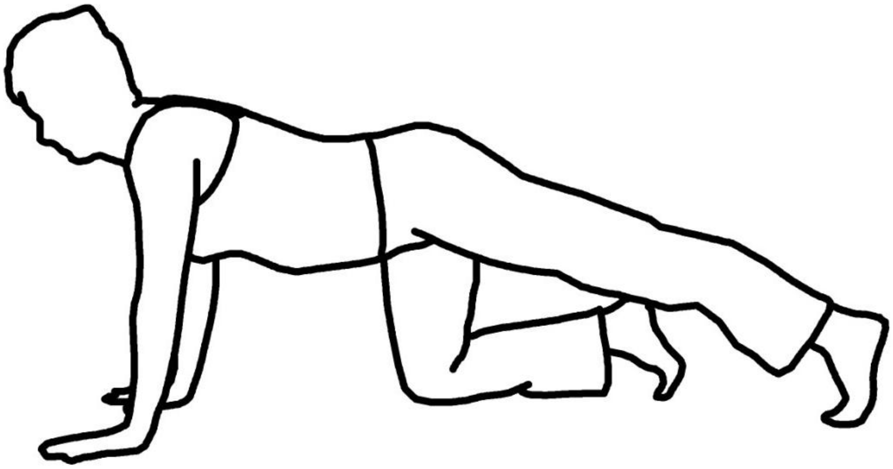

Toe Taps

Description: Abdominal exercise lying on back with knees bent and feet off the floor

How to teach: Begin with three pelvic tilts. Take the feet off the floor so that the legs are at a right angle with the knees over the hips and the feet in line with the knees (the shins parallel to the floor). The hands are either interlaced behind the head or down by the sides of the body gripping the sides of the mat. If the hands are holding the mat, turn the upper arms down toward the floor. On an exhale, press the abdomen down toward the floor, keep the torso and one leg stable as the other toe slowly goes to the floor lightly taps the floor and comes back up again. Repeat with the other foot. Repeat one toe then the other 10 times, maintaining a slow steady smooth breath. Keep the face and shoulders relaxed. Make sure not to lift the abdomen up toward the ceiling or let the pelvis rock up and down as the legs move; only one leg is moving at a time, everything else is stable.

Modifications:

1. From Pelvic Tilt Pose (p.70), lift one foot up off the floor at a time, alternating feet.
2. If the head is tilting back, place blanket under head.

Triangle Pose

Description: With straight legs and arms, the body bends sideways to make several triangle shapes.

How to teach: From Mountain Pose (pg.68) separate feet 3.5-4 feet apart with feet parallel to each other and toes pointing forward. Extend arms out to sides at shoulder height with palms facing down. Turn right leg to the right 90˚. Align right foot, knees and front thigh in a straight line. Turn left foot slightly in so toes are pointing slightly toward the right. Exhale, extend torso to the right, bending from hips. Place right hand onto right leg and left arm straight up in line with left shoulder. Keep legs, arms and back straight. Inhale, raise arms and torso back up. Turn feet back to face forward. Repeat on other side.

Modification:

1. Triangle at wall
2. Place right hand on a chair seat when bending to the right, and vice versa on left.
3. Chair: Sitting in a chair, extend right leg out to side turning the whole leg out 90 degrees with the toes pointing to the side wall. The left leg is bent with the foot on the floor. Bend to the right side at the hips, bending directly over the extended leg and place the right hand on the leg.

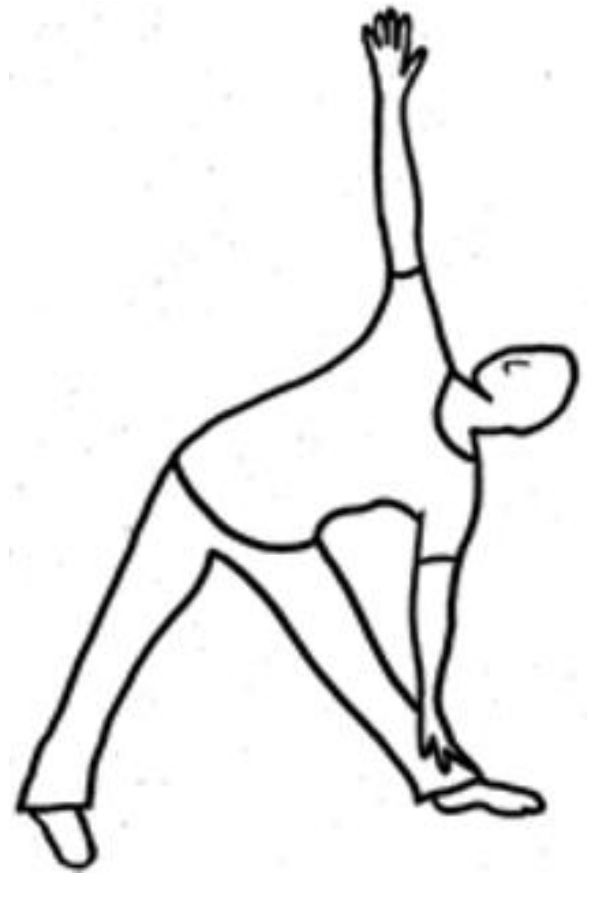

Triangle at Wall Pose

Description: Stand with feet 3-4 feet apart at right angle to wall. Bend from hips to reach arm closest to wall toward the wall.

How to teach: Stand with left side facing and left hand on wall. Turn left leg toward wall with toes close to or touching the wall. Turn right foot slightly in toward the wall so that the right foot is at a diagonal. Bend at left hip toward the wall, and move right hand up above head. Keep head in line with left foot (head, left arm and leg aligned with each other).

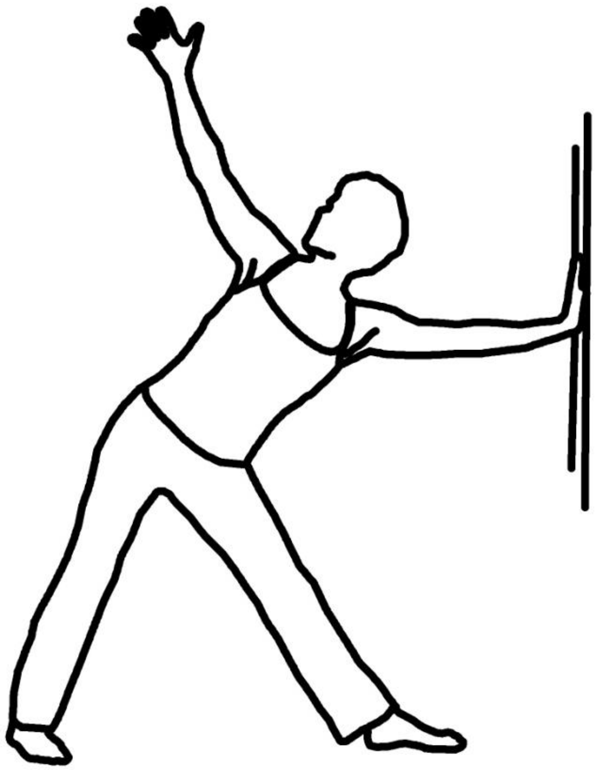

Wall Dog Pose

Description: Standing forward bend with hands on the wall instead of the floor and legs back at a diagonal

How to teach: Stand facing the wall with feet hip width apart. Bend forward from hips, placing hands on the wall and walk hands up the wall slightly higher than shoulder height. Stand far enough from the wall so that the legs angle back with the arms, legs and back all straight. Press down through the feet and pull up through the thighs. Pull thighs and hips back away from the wall while gently pressing hands into the wall. Keep head in line with the arms.

Modifications:

1. Chair: Sit on edge of a chair, extend both legs out, hips weigh apart and raise both arms straight up over head, shoulder distance apart.

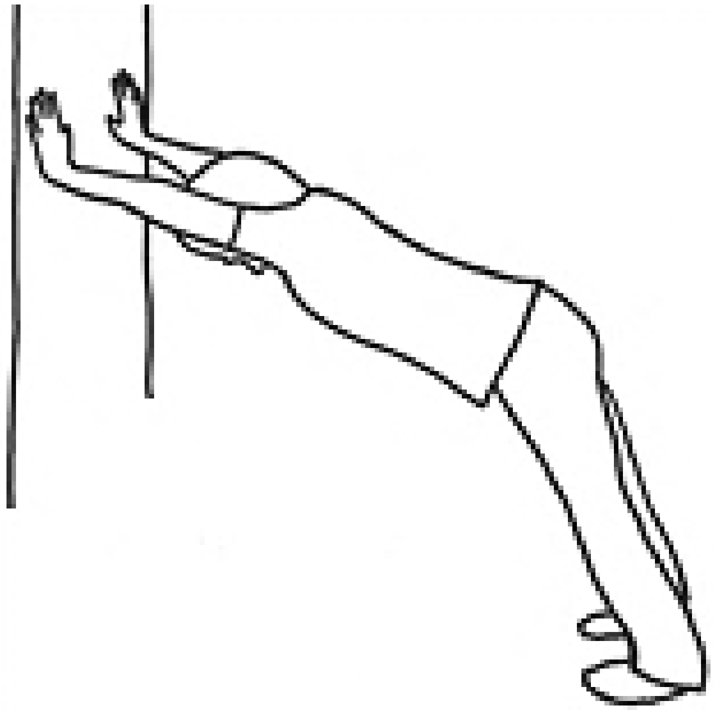

Warrior I Pose

Description: Standing with feet apart, front knee bent, arms raised up with fingers pointing to the ceiling in line with shoulders.

How to teach: From Mountain Pose (pg. 68) move the right leg 3.5-4 feet back. Keep feet parallel to each other with toes pointing forward. Extend arms out to the sides at shoulder height with palms facing down. Turn right leg to the right 90˚.Turn left foot in about 60˚ to the right, turning shoulders and torso to the right with arms extended to the sides at shoulder height to face toward the right leg. Exhale, bend knee to form a 90˚ (knee over ankle). Keeping back leg straight and firm (adjust distance between feet to put knee over ankle). Lift abdomen up toward head while moving lower back down. Inhale, lift chest and raise arms straight up over head in line with ears. Lift up through the arms, lengthening the whole spine. Be careful not to arch lower back. To come out of pose, exhale arms down, straighten right leg then back to standing position and step back to Mountain Pose.

Modifications:

1. Step back foot slightly to the side, away from center of the body to make a wider stance if unsteady.
2. Place hands on a chair in front of body
3. Warrior I at Wall.
4. Chair: Using a chair without arms, sit sideways on the chair with the right leg bent into a right angle (with the knee over the ankle, shin perpendicular to the floor) and the left leg extended behind. Facing sideways on the chair, raise the arms up over head and lift chest. If the arms up cause back pain, keep the arms on the hips.

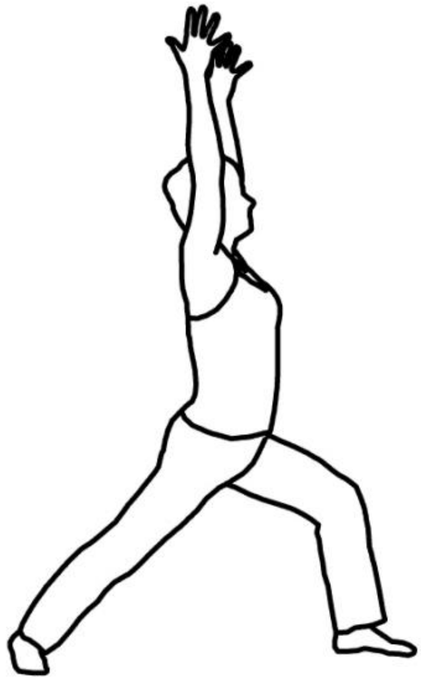

Warrior I at Wall Pose

Description: Facing wall with hands and one foot at wall, bend knee to form a right angle.

How to teach: Stand facing the wall, with hands on the wall at shoulder height. Step right foot up to the wall, step left leg back as far as possible. Turn back foot out slightly and stretch through back leg. Try to press the left heel down into the floor as much as possible. Lift abdomen up while moving the lover back down (making sure not to arch lower back). Lift chest up and relax shoulders down. On an exhale, bend the right leg into a right angle so that the knee is directly over the ankle and the thigh is as parallel to the floor as possible. Try to keep spine upright (vertical). Stay for several breaths, inhale and straighten leg to come out. Repeat on left.

Modifications:

1. Allow the back of the heel to come off floor
2. Place a block between knee and wall pushing knee into block.
3. Chair: Using a chair without arms, sit sideways on the chair with the right leg bent into a right angle (with the knee over the ankle, shin perpendicular to the floor) and the left leg extended behind. Facing sideways on the chair, raise the arms up over head and lift chest. If the arms up cause back pain, keep the arms on the hips.

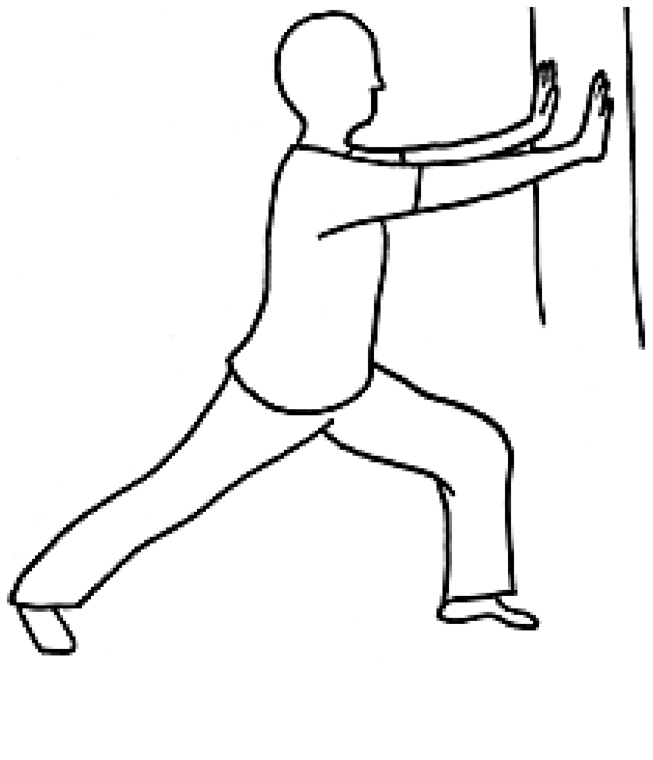

Guideline for Teaching Yoga Relaxation Exercises

Preparing for Relaxation Exercises

Preparing the Body for Relaxation Exercises

Remove glasses if you wear any. Remove clips from back of head. Stay warm – if you would like, you can put on a sweater or cover yourself with a blanket. Soften your eyes, letting them rest in their sockets. Lie flat on your back with arms resting comfortably, palms upward. Separate legs slightly, feet hip width apart. Extend the legs and slightly turn them outward. Allow your legs and feet to roll out to the side. Make any additional adjustments so you are comfortable.

Let your eyes soften or close. Let your head be heavy. Let body sink into the mat. Circle your wrists and ankles. Settle the mind. Let go of effort. Breathe naturally. Surrender your body to gravity. Begin with a sense of softening, opening.

Release your body to the mat. Release arms slightly out to the sides of body, turn palms upward. Move shoulder blades in toward your back opening and broadening chest. Release neck and gently move it from side to side. Begin to settle into the body. Consciously relax the body. Relax the muscles of your face, let your eyes be still, let your eyes close if you'd like. Let your jaw relax. Let your tongue rest inside your mouth. Let your body sink into the mat. Consciously relax your entire body, letting it become heavy. Feel how the earth supports the weight of your body in its entirety. Let go. Release all effort.

Turn attention to breath. Let the breath become steadier, longer, and more even. Continue to soften muscles and release tension. Follow the inhalations and exhalations, remaining awake. Notice how the world is when you are still inside.

Preparing for Relaxation Exercises

Guiding Participants through the Relaxation Exercises

Use the guided meditations listed below and the description for Savasana to guide participants through the relaxation exercise. Try to use a variety of relaxation exercises throughout the intervention. The included exercises are only guidelines; feel free to incorporate additional exercises you may use in your practice or to modify any of the listed exercises. With all of the relaxation exercises, encourage participants to be aware of any sensations, experiences, and mental or physical feelings that may arise but not to judge or criticize what they may feel.

Ending the Relaxation Exercise

Gently rock yourself over to your right side bringing your knees up in fetal position. Take a few breaths here as you settle into this first position you were in before you were born. Take a slow calming breath and press your left hand down to the mat as you rise up into a comfortable seated position.

Relaxation Exercises

Savasana

Description: Lying supine on mat, palms facing upwards, feet hip width apart.

How to teach: Lie flat on back with arms resting comfortably, slightly out from the sides of body, palms upward. Separate legs so that they are apart enough to allow legs and feet to roll out to each side. Move shoulder blades in toward back opening and broadening chest. Release neck and gently move it from side to side.

Modifications:

1. Assistant covers each participant with a blanket
2. If there is back discomfort, place bolster or blanket under knees
3. If there is back discomfort, place calves on a chair seat
4. Seated relaxation: Sit against wall or on chair with spine supported and blanket over legs and/or with head resting on knees.
5. Side relaxation: Lying on side with support of blanket under head, under top arm and another supporting and/or between knees.
6. Belly Relaxation: Lying on belly, with feet slightly turned in toward each other and with hands or a blanket folded under head.

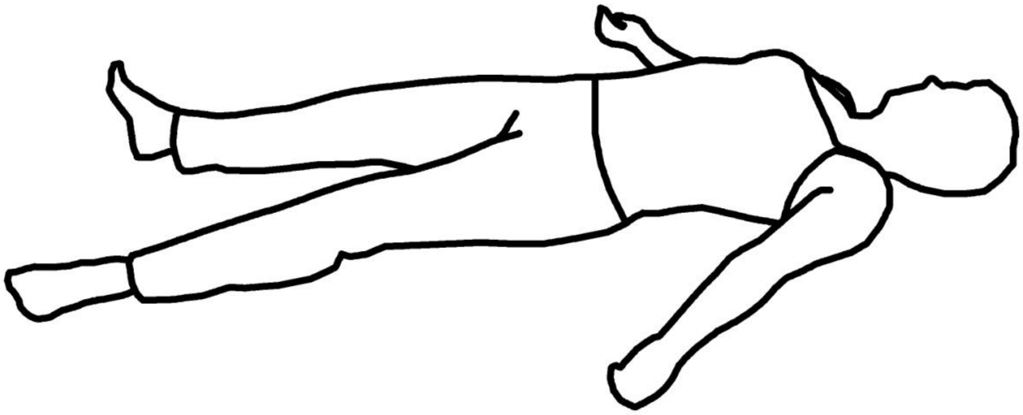

Suggestions for Relaxation Exercises

Body Scan:

- Begin by focusing on the breath. Become aware of normal breathing.
- Move the focus to the body. Become aware of any sensations, uncomfortable parts, the temperature and feel of the body.
- Guide participants to relax all visible body parts starting with the feet and moving to the crown of the head.
- Move through the toes, feet, ankles, calves, knees, upper legs, hips, torso, fingers, wrists, arms, shoulders, neck, jaw, mouth, tongue, nose, eyes, forehead, ears, and whole face.
- Notice any sensations, positive, negative or neutral. There may not be any sensations.
- To end, start by wiggling toes and then fingers, slowly moving the body. Notice any changes in the body without judgment or resistance.

Progressive Muscle Relaxation:

- Begin by tensing all the muscles of the face. Make a tight grimace, close the eyes, and clench the jaw, mouth, and lips. Hold this tightness for the count of three while inhaling.
- Now exhale and relax completely. Completely relax the face and feel the tension seep away from facial muscles.
- Inhale, then while exhaling stretch the muscles of the face by opening the mouth as wide as possible, sticking out the tongue and rolling the eyes up. Inhale and exhale normally.
- Completely tense the neck and shoulders, again inhaling and counting to three. Then exhale and relax.
- Continue down the body, repeating this procedure for the chest, abdomen, right and left arms (making a fist), hips, right and left legs.

Circle Joints:

- Bend one leg, lifting it up slightly off the floor. Circle ankle in one direction three times, and three times in the other direction. Continue with knee, then whole leg. Move progressively to the hands, arms, then shoulders.

Suggestions for Relaxation Exercises

Conscious Relaxation:

- Lie on back, close eyes. For each body part, feel the body part, sense the weight. Repeat this activity for the following body parts: feet, knees, upper legs and thighs, abdomen and chest, hips, hands, upper arms, neck, head and skull, mouth and jaw (pay special attention to if jaw muscles are clenched), eyes (sense if forcibly closing eyelids), face and cheeks.
- Mentally scan your body. If you find any place that is still tense, consciously relax that place and let it sink to the floor.

Deep Relaxation

- Lie down comfortably with whatever support needed. Let eyes soften or close. Let head be heavy and sink into floor. Let go of any effort, just relax and surrender body to gravity. Let body soften. Relax from your daily demands and challenges, letting the mind settle on the breath. Relax your arms, legs, shoulders, tongue away from teeth, jaw, relax all the muscles of the face, letting go of any facial expressions. Relax the eyes, letting them sink back to sockets, imagining they are two pebbles floating in a pond from the top to the bottom of bond. Relax between eyebrows, skin of forehead from the center of temples. Let any tension in brain drain from forehead down back to the skull. Relax mind, letting all chatter quiet. Feel the breath coming and going on its own without any effort.
- Remind participants to relax completely, focusing the mind on the breath for as long as possible, trying to be fully present with each inhale and relaxing deeper with each exhale. When participants realize mind has wandered, gently bring it back to the breath.

Guideline for Teaching Yoga Breathing Exercises by Week

Breathing Exercises

All breathing exercises are done lying down on the back. Set up 2 blankets, one folded in a long rectangular shape to go the length of the spine from the waist to the top of the head. The other folded for a "pillow" placed on top of the first blanket, going under the neck and head. Place a bolster if available under the knees. Start with relaxation, closing eyes lightly. It's very important that throughout the exercises breathing always remains comfortable, never forcing it or pushing it. If there is any struggle, go back to normal breathing to recover.

Awareness of Normal Breath

Week 1

1. Breathing through the nose with the mouth closed, notice the feel of the air as it comes into your body and then goes out. Be aware of your body breathing in, saying to yourself, "I am breathing in." Be aware of your body breathing out, saying, "I am breathing out." Repeat.
2. Don't control the breath, just be aware of your body breathing without any effort on your part.

Week 2

1. Notice the feel of the air as it comes into your body and then goes out. Where do you feel the breath in your body the most?
2. Notice the temperature of the air as it comes into the nose at the nostrils and again as the air goes out. Does the temperature of the air change from the inhale to the exhale?
3. Notice the quality of the breath. Is the breath bumpy or smooth, light or heavy, starting and stopping?
4. Notice the normal length of the inhale and exhale. Is the inhale longer or shorter than the exhale?
5. These questions don't need answers, they are to focus attention and continue normal breath awareness.

Breathing Exercises

Even Breathing

Week 3

1. Review normal breath awareness.
2. Breathing in, synchronize the chest expanding upward and outward at the same time.
3. Breathing out, notice the same on the inhale if one side of the lungs inflates more than the other. Breath in, filling both lungs evenly. Same for breathing out. To teach this, you can have participants come into Child's Pose (on the floor or using two chairs). Teacher and assistant should ask permission to place their hands on each side of the participant's ribs. Ask participant to breathe into hands evenly and smoothly. Teacher and assistant will be able to feel the breath on each side of the lungs and inform the participant if they are breathing evenly. The participants can make corrections as they are able, never forcing the breath.

3-Part Breathing

Weeks 4 and 5

1. Teach participants the location of the diaphragm muscle and how it needs to relax when breathing.
2. Place hands on lower belly and naval area. Relax belly and breathe normally with easy, comfortable breaths. Notice if there is any movement under the hands. Gently, draw the belly in on the inhale and relax the belly on the exhale.
3. Place hands on lower side ribs (floating ribs). With a relaxed belly, gently try to breathe out so that the ribs move out into hands. Practice breathing, moving lower ribs out into hands and back in like an accordion—going in and out.
4. Place hands on upper chest, under collarbones. Imagine nostrils under hands and breathe in under hands. Notice if the upper chest has any movement with easy, normal breaths, being careful not to tense neck and shoulders.
5. Take normal, slow, smooth, easy breaths, breathing in until there is a full feeling. Breathe out until there is an empty feeling and it is noticeable where the breath moves in the torso. Discuss how the torso is like a glass that fills from the bottom to the top when it is filled with water. In the same way the lungs fill from the bottom to the top with air. Exhale, empty lungs slowly from the top to the bottom, from upper chest down to belly.

Breathing Exercises

Lengthening each Exhale

Week 6

1. Review synchronizing and evening out the breath.
2. Start with normal breathing, then exhale quietly until the lungs feel empty but without pressing down on the abdomen.
3. Inhale normally through the nose.
4. Exhale slowly, deeply, steadily, until the lungs feel empty.
5. Continue with normal inhales and slow, deep, steady, and conscience exhales, lengthening each exhale comfortably.
6. Discuss how lengthening the exhale helps us relax and release tension and stress.

Lengthening each Inhale

Week 7

1. Review normal breath awareness.
2. Relax the diaphragm and stretch it sideways while breathing in and without inflating the abdomen. Slowly, deeply, and steadily inhale until you feel full.
3. Exhale slowly, easily but not deeply and not all at once. The exhale will be slightly longer than normal.
4. Discuss how lengthening the inhale helps to invigorate and energize us.

Lengthening each Inhale and Exhale

Weeks 8 and 9

1. Relax with normal breathing
2. Exhale fully
3. Breathe in until you feel full, letting your ribs lift and spread as you breathe in. Notice that there is a slight natural pause at the end of an inhale before new exhale begins.
4. Breathe out slowly, deeply, and steadily, without letting all the air out at once. Exhale gradually until you feel empty, maintaining the fullness of the lungs as long as possible. Notice that there is a slight natural pause at the end of the exhale before a new inhale begins.
5. After several cycles of slow, deep, and steady breaths, begin to lengthen the breath by counting each inhale and exhale. Inhale, slowly counting, 1, 2, 3. Pause, exhaling slowly counting 1, 2, 3, pause. Have the participants take a natural breath in between each slow breath. Next, inhale counting slowly, 1, 2, 3, 4, pause, exhale, 1, 2, 3, 4, pause. Normal breath. Have participants choose to either repeat inhaling and exhaling for the count of 4, return to a count of 3, or lengthening to a count of 5. It is very important that the breath remains comfortable, never forcing or pushing it.

Breathing Exercises

Ocean Breath (Ujjayi Breath)

Weeks 10, 11, 12

1. Inhales are made with a sibilant sound "sss" and exhales are made with an aspirate "hhh."
2. Exhaling through the nose, slightly close or contract the back of the throat to make a quiet "hhh" sound. Keep the back of the throat contracted while inhaling to quietly make the "sss" sound. To learn to make the sound, start with an exhale through the mouth. Try using one or several of these images to teach the sound:
    - Breathe out as you would to fog a mirror
    - Breathe like when you whisper
    - Breathe as you would if you were trying to see your breath on a cold day.
    - Breathe so it sounds like a quieter Darth Vader.
3. If the sound isn't coming, let it go. It will come on its own with practice.
4. Listening to the sound of the breath helps us stay focused and also gives information about its quality, rhythm and pace.

Poems
By Week

Poems by Week

Week One

Look To This Day

Look to this day:

For it is life, the very life of life.

In its brief course

Lie all the verities and realities of your existence.

The bliss of growth,

The glory of action

The splendor of achievement

Are but experiences of time.

For yesterday is but a dream

And tomorrow is only a vision;

And today well-lived, makes

Yesterday a dream of happiness

And every tomorrow a vision of hope.

Look well therefore to this day;

Such is the salutation to the every-new dawn!

-Kalidasa

Week Two

Conscious Breathing

"There are a number of breathing techniques you can use to make life vivid and more enjoyable. The first exercise is very simple. As you breathe in, you say to yourself, 'Breathing In, I know that I am breathing in.' And as you breathe out say, 'Breathing out, I know that I am breathing out.' Just that. You recognize your in-breath as an in-breath and your out-breath as an out-breath. This technique can help you keep your mind on your breath. As you practice, your breath will become peaceful and gentle, and your mind and body will also become peaceful and gentle. Breathing in and out is very important, and it is enjoyable. Our breathing is the link between our body and our mind. By concentrating on our breathing, 'In' and 'Out,' we bring body and mind back together and become whole again. Conscious breathing is an important bridge."

-Thich Nhat Hanh, *Peace is Every Step*

Poems by Week

Week Three

The Journey

One day you finally knew
What you had to do, and began,
though the voices around you
Kept shouting their bad advice-
Though the whole house
Began to tremble
And you felt the old tug
At your ankles.
"Mend my life!"
each voice cried.
But you didn't stop.
You knew what you had to do,
Though the wind pried
With its stiff fingers
At the very foundations,
Though their melancholy
Was terrible.
It was already late
Enough, and a wild night,
And the road full of fallen
Branches and stones.
But little by little,
As you left their voices behind,
The stars began to burn
Through the sheets of clouds,
And there was a new voice
Which you slowly
Recognized as your own,
That kept you company
As you strode deeper and deeper
Into the world,
Determined to do
The only thing you could do - - -
Determined to save
The only life you could save.

-Mary Oliver, *Dream Work*

Week Four

Go In and In

Go in and in.
Be the space
Between two cells,
The vast, resounding
Silence in which
Spirit dwells.
Be sugar dissolving
On the tongue of life.
Drive in and in,
As deep as you can dive.
Be infinite, ecstatic truth.
Be love conceived and born in union.
Be exactly what you seek,
The Beloved, singing Yes,
Tasting Yes, embracing Yes,
Until there is only essence;
The All of Everything
Expressing through you
As you. Go in and in
And turn away from
Nothing that you find.

-Danna Faulds, *Go In and In: Poems From the Heart of Yoga*

Week Five

THE LOST HORSE

Long ago in a village in Northern China, there lived a man who owned a magnificent horse. So beautiful was this horse that people came from miles around just to admire it. They told him he was blessed to own such a horse.

"Perhaps," he said. "But what seems like a blessing may be a curse."

One day, the horse ran off. It was gone. People came to say how sorry they were for his bad luck.

"Perhaps," he said. "But what seems like a curse may be a blessing."

A few weeks later, the horse returned. It was not alone. It was followed by twenty-one wild horses. By the law of the land, they became his property. He was rich with horses. His neighbors came to congratulate him on his good fortune.

"Truly," they said, "you have been blessed."
"Perhaps. But what seems like a blessing may be a curse."

Shortly after that his son – his only son – tried to ride one of the wild horses. He was thrown from it and broke his leg. The man's neighbors came to say how sorry they were. Surely, he had been cursed.

"Perhaps," he said. "But what seems like a curse may be a blessing."

A week later, the emperor came through that village, drafting every able-bodied young man for a war against the people of the north. It was a horrible war. Everyone who went from that village was killed. Only that man's son survived, because of his broken leg.

To this day, in that village, they say, "What seems like a blessing may be a curse. And what seems like a curse may be a blessing."

-Ancient Chinese Tale, as told by Joel ben Izzy

Week Six

Remember

Remember the sky that you were born under,
Know each of the star's stories.
Remember the moon, know who she is.
Remember the sun's birth at dawn, that is the
Strongest point of time. Remember sundown
and the giving away to night.
Remember your birth, how your mother struggled
to give you form and breath. You are evidence of
her life, and her mother's and hers.
Remember your father. He is your life, also.
Remember the earth whose skin you are;
red earth, black earth, yellow earth, white earth
brown earth, we are earth.
Remember the plants, trees, animal life who all have their
tribes, their families, their histories, too. Talk to them,
listen to them. They are alive poems.
Remember the wind. Remember her voice. She knows the
origin of this universe. Remember that you are all people and that all people
are you.
Remember that you are this universe and that this
universe is you.
Remember that all is in motion, is growing, is you.
Remember that language comes from this.
Remember the dance that language is, that life is.
Remember

~ Joy Harjo, *She Had Some Horses*

Week Seven

Do yoga with no goal but
To be in the moment. This
Breath, this stretch, this wave
Of emotion rolling in. Watch it
Crest, and break, then dissipate.

Hold the body like a
Lover in a close embrace,
Listening with intimacy,
Touching with tenderness.

Yoga is a threshold into
Mystery; each pose an
Open doorway and an
Invitation to unfold.

Sensations rise and fall,
And through it all the
Deathless center radiates
The simple truth of union.

~ Danna Faulds, *Go In and In: Poems From the Heart of Yoga*

Week Eight

Let It Go

Let go of the ways you thought life
would unfold; the holding of plans
or dreams or expectations - Let it
all go. Save your strength to swim
with the tide. The choice to fight
what is here before you now will
only result in struggle, fear, and
desperate attempts to flee from the
very energy you long for. Let go.
Let it all go and flow with grace
that washes through your days whether
you receive it gently or with all your
quills raised to defend against invaders.
Take this on faith: the mind may never
find the explanations that it seeks, but
you will move forward nonetheless.
Let go, and the waves' crest will carry
you to unknown shores, beyond your
wildest dreams or destinations. Let it
all go and find the place of rest and
peace, and certain transformation.

~ Danna Faulds, *Poems from the Heart of Yoga*

Week Nine

Our true home is in the present moment.
To live in the present moment is a miracle.
The miracle is not to walk on water.
The miracle is to walk on the green Earth in the present moment, to appreciate the peace
and beauty that are available now.
Peace is all around us—
In the world and in nature-
And within us-
In our bodies and our spirits.
Once we learn to touch this peace,
We will be healed and transformed.
It is not a matter of faith;
It is a matter of practice.

-Thich Nhat Hanh, *Touching Peace: Practicing the Art of Mindful Living*

Week Ten

Joy at Sudden Disappointment

Whatever comes, comes from a need,
A sore distress, a hurting want.
Every part of you has a secret language.
Your hands and your feet say what you've done.
And every need brings in what's needed.
Pain bears its cure like a child.
Ask a difficult question,
And the marvelous answer appears.
Be thirsty for the ultimate water,
And then be ready for what will
Come pouring from the spring.
This spreading radiance
Of a True Human Being has great importance.
[Let yourself] open out like a rose
losing itself petal by petal.
Don't grieve for what doesn't come.
Some things that don't happen
keep disasters from happening.

-Rumi Jelaluddin

Week Eleven

Acceptance

When the spent sun throws up its rays on cloud
And goes down burning into the gulf below,
No voice in nature is heard to cry aloud
At what has happened. Birds, at least must know
It is the change to darkness in the sky.
Murmuring something quiet in her breast,
One bird begins to close a faded eye;
Or overtaken too far from his nest,
Hurrying low above the grove, some waif
Swoops just in time to his remembered tree.
At most he thinks or twitters softly, 'Safe!
Now let the night be dark for all of me.
Let the night be too dark for me to see
Into the future. Let what will be, be.'

~ Robert Lee Frost

Week Twelve

With All Your Heart

Say out loud, I want to live a happy life
Listen to what you just said
Take notice of things
Surround yourself with people you love
Listen to the wind
Imagine
Let everything change all the time
Let go of the why
Welcome miracles
Thank god
Constantly breath
Tell the truth about how you feel
Make choices
Want what you want
Let the animals reach you
Have children teach you
Take good care of your body
Love passionately
Share your dreams
Spread your gifts
Check it out
Forgive the past
Dive in
Eat it up
Take chances
Be real.

-Anonymous

The 12 Principles

The following at the 12 principles used during the Back to Health Study. These themes are incorporated into each class through the yoga philosophy section and the teachings for each class.

- **Week 1 (Define Yoga):** Participants are given a context and overview of the yoga intervention. They are introduced to yoga as tool to help relieve their low back pain through a union of body, mind, breath, and soul. They are taught how to enter, be in, and exit postures safely and mindfully.

- **Week 2 (The Practice of Non-violence):** Participants are encouraged to practice non-violence toward others and themselves. The difference between harmful pain and healthy discomfort in poses is discussed.

- **Week 3 (Truth and Honesty):** Participants are encouraged to be honest with themselves in their practice of yoga as well as to recognize and have compassion for their own weaknesses and accept that they may have to do poses differently than others in the class.

- **Week 4 (Purity/Cleanliness):** Participants are taught to become aware of the cleansing elements of yoga, to feel the breath clearing the body of toxins and bringing in new oxygen. They are encouraged to incorporate healthy habits into their lives outside of yoga (e.g., healthy food and relationships).

- **Week 5 (Contentment/Gratitude):** Participants are encouraged to feel at peace in the present moment and grateful for what we have right now. In yoga practice, participants are encouraged to focus on what they can do, instead of what they cannot do.

- **Week 6 (Discipline/Will Power):** Like achieving goals in life, participants are taught that staying with our practice of yoga requires discipline, will power, and patience. With discipline/will power we can move toward doing whole poses that might be initially difficult for us.

The 12 Principles

- **<u>Week 7 (Study and Awareness):</u>** Participants are introduced to the idea of self-awareness and encouraged to be aware of when to make adjustments in their poses. Similarly, with awareness in life we can work toward changing old unhealthy habits.

- **<u>Week 8 (Acceptance/Letting Go):</u>** Participants are encouraged to accept themselves and the current moment, practicing this acceptance in each pose.

- **<u>Week 9 (Moderation/Not Greed):</u>** Participants are encouraged to be in control of their desires and to seek balance in their lives. In yoga practice, it is better to practice a little bit of yoga frequently than to practice a lot of yoga every once in a while.

- **<u>Week 10 (Balancing Effort and Ease):</u>** The concepts of effort and ease in yoga postures are discussed. Participants are taught how to balance effort and ease in their yoga practice and encouraged to seek similar balance in their daily lives.

- **<u>Week 11 (You are what you practice):</u>** Participants are encouraged to practice being the person they would like to be (e.g. compassion not judgment, friendliness instead of anger, joy for others instead of jealousy).

- **<u>Week 12 (We're all in This Together):</u>** Participates are guided to accept the ups and downs of life with equanimity As they begin to accept themselves and their challenges in their practice of yoga, they are encouraged to also carry this self-acceptance into daily life and to the acceptance of other people and their challenges. This understanding gives a sense of common ground and connection not only within ourselves but with all people".

Bibliography

Bibliography

Barks, Coleman, with John Moyne, trans. *The Essential Rumi*. Edison, New Jersey: Castle Books, 1995.

Chodron, Pema. *The Wisdom of No Escape: And the Path of Loving-Kindness*. Boston: Shambhala Publications, 1991.

Cope, Stephen. *The Wisdom of Yoga: A Seeker's Guide to Extraordinary Living*. New York: Random House, Inc., 2006.

Cope, Stephen. *Yoga and the Quest for the True Self*. Random House, 2000. New York, Bantam, 2000.

Farhi, Donna. *Teaching Yoga: Exploring the Teacher-Student Relationship*. Berkley, California: Rodmell Press, 2006.

Faulds, Danna. *Go In and In: Poems From the Heart of Yoga*. Greenville, Virginia: Peaceable Kingdom Books, 2002.

Feurstein, Georg, Ph.D. *The Philosophy of Classical Yoga*. Rochester, Vermont: Inner Traditions International, 1996.

Feuerstein, Georg, Ph.D. *The Yoga Tradition: Its History, Literature, Philosophy and Practice.* Prescott, Arizona: Hoh Press, 1998.

Frost, Robert. *The Poetry of Robert Frost*. New York: Henry Holt and Company, 1969.

Gates, Rolf and Kenison, Katrina. *Meditations from the Mat: Daily Reflections on the Path of Yoga*. New York: Random House, 2002.

Hafiz, with Daniel Ladinsky, trans. *The Gift*. New York: Penguin Compass, 1999.

Hanh, Thich Nhat. *The Thich Nhat Hanh Collection: Peace Is Every Step, Teachings on Love, The Stone Boy and Other Stories*. One Spirit, New York: Bookspan, 2004.

Bibliography

Harjo, Joy. *She Had Some Horses.* New York: Thunder's Mouth Press, 2006

Iyengar, B.K.S. *Light on Yoga*. New York: Schocken Books, 1966.

Kaminoff, Leslie. *Yoga Anatomy*. Human Kinetics: Champlain, Illinois, 2007.

Lasater Judith, Ph.D., P.T. *Living your Yoga: Finding the Spiritual in Everyday Life*. Berkeley, California: Rodmell Press, 2000.

Miller, Richard, C. Ph.D. *Breathing for Life: Articles on the Art and Science of the Breath.* Sebastopol, California: Anahata Press, 1990.

Oliver, Mary. *Dream Work*. New York: Atlantic Monthly Press, 1986.

Oliver, Mary. *New and Selected Poems*. Boston, MA: Beacon Press, 1992.

Oman, Maggie, ed. *Prayers for Healing*. San Francisco, California, Conari Press, 1997.

Parry, Danaan. *Warriors of the Heart, Earth Stewards*. Bainbridge Island, Washington: Network Publications, 1991.

Saper RB, Sherman KJ, Cullum-Dugan D, Davis RB, Phillips RS, Culpepper L. Yoga for chronic low back pain in a predominantly minority population: a pilot randomized controlled trial. Alternative Therapy Health Medicine. 2009 Nov-Dec;15(6):18-27.

Roberts, Elizabeth & Amidon, Elias*. Life Prayers: 365 Prayers, Blessings, and Life Affirmations to Celebrate the Human Journey*. San Francisco, California: Harper Collins, 1996.

Walcott, Derek. *Collected Poems 1948-1984*. New York: Farrar, Straus and Giroux, 1986.

Weber, Eva Bell. *Journey with the Master*. Camarillo, California: Devorss & Company,1984.

Appendix I

Supplementary Readings and Poems

Supplementary Readings and Poems

Love After Love

The time will come
When, with elation
You will greet yourself arriving
at your own door, in your own mirror,
and each will smile at the other's welcome,
and say, sit here. Eat.
You will love again the stranger who was yourself.
Give wine. Give bread. Give back your heart
To itself, to the stranger who has loved you
All your life, whom you ignored
For another, who knows you by heart.
Take down the love letters from the bookshelf,
The photographs, the desperate notes,
Peel your own image from the mirror.
Sit. Feast on your life.

-Derek Walcott

Breathing in, I calm my body. Breathing out, I smile. Dwelling in the present moment I know exactly where I am; I know this is a wonderful moment.
"Calming, Smiling, Present Moment, Wonderful Moment"
~Thich Nhat Hanh

Breathing in, I know that I am breathing in.
Breathing out, I know that I am breathing out.
Breathing in, I see myself as a flower.
Breathing out, I feel flesh.
Breathing in, I see myself as a mountain.
Breathing out, I feel solid.
Breathing in, I see myself as still water.
Breathing out, I reflect things as they are.
Breathing in, I see myself as space.
Breathing out, I feel free
~Thich Nhat Hanh

Supplementary Readings and Poems

Love Meditation

May I be peaceful, happy, and light in body and spirit.
May I be safe and free from injury
May I be free from anger, afflictions, fear, and anxiety.
~ Thich Nhat Hanh

May I be well.
May I be happy.
May I be healed from suffering.
May I be at peace.
May everyone be well.

May everyone be happy.
May everyone be healed from suffering
May everyone be at peace.

~Ancient Buddhist Quotation

Namaste

I honor the place in you
In which the entire universe dwells.
I honor the place in you
Which is of love, of truth,
Of light and of peace.
When you are in that
Place in you,
And I am in that place in me,
We are one.
~ Traditional Yogic Salutation

Structured Hatha Yoga Protocol

Yoga Posture (*Asana)*	Classes Incorporating Posture by Segment				Total Classes Incorporating Posture
	Segment 1 Weeks 1-3<br><br>Opening to Something Greater	Segment 2 Weeks 4-6<br><br>Listening to the Wisdom of the Body	Segment 3 Weeks 7-9<br><br>Engaging Your Power	Segment 4 Weeks 10-12<br><br>Bringing it Home	
Svasana Relaxation and Breathing Exercises	✓	✓	✓	✓	12
Yoga Postures					
Knee to Chest*	✓	✓	✓	✓	12
Knee Together Twist*	✓	✓	✓	✓	12
Pelvic Tilt*	✓	✓	✓		9
Cat and Cow Pose (and modifications)*	✓	✓	✓		9
Chair Pose (and modifications)*	✓	✓	✓		9
Shoulder Opener*	✓	✓	✓	✓	12
Crescent Moon (and modifications)*	✓	✓	✓		9
Mountain Pose (and modifications)*	✓	✓	✓	✓	9
Chair twists, standing & seated	✓	✓	✓	✓	12
Child's Pose*	✓		✓	✓	9
Cobra (and modified)*	✓	✓	✓	✓	12
Bridge Pose* (with and without support	✓	✓	✓	✓	12
Reclining Cobbler*	✓	✓	✓	✓	12
Downward Facing Dog (and at wall)*	✓	✓	✓	✓	12
Triangle Pose (with and without the wall)		✓	✓	✓	9
Locust Pose*	✓	✓			6
Sphinx*		✓	✓	✓	9
Standing forward bend at wall*		✓	✓	✓	9
Warrior Pose*			✓	✓	5
Extended Leg Pose*		✓	✓	✓	8
Sun Salutations				✓	3
Baby Dancer Pose*				✓	3
Spinal Rolls				✓	3
Svasana Integrative Relaxation	✓	✓	✓	✓	12

Acknowledgements

There are several groups we would like to recognize in the production of this manual. First there is the expert panel of yoga teachers who helped to develop an early draft of the yoga for chronic low back pain manual. These members include Diana Cullum-Dugan, Maya Breuer and Deborah Neubauer.

Secondly, we would like to recognize the co-author of a previous draft of this manual, Anna Dunwell.

The group of dedicated yoga teachers who taught the yoga for chronic low back pain protocol and provided feedback for this manual were integral to its development. These include Smoke Montgomery, Lisa Cahill, Deidre Alessio, Danielle Ciofani, Victoria Garcia Drago, Andrea Soler and Margaret Furtado.

We would also like to thank the research staff who worked on the early manuals, including Nadia Khouri, Florence Uzogara, Julia Keosaian, Christian Cerrada and Danielle Dresner.

We would like to recognize Carol Faulkner for her work on this manual. Her work has been instrumental to the development of this version.

Lastly, those that worked on the current version of the yoga for chronic low back pain teachers manual which include Martina Tam, Margo Godersky and Shayna Egan.
