# Supplementary material for: Yoga vs. physical therapy vs. education for chronic low back pain in predominantly minority populations: study protocol for a randomized controlled trial
Source: Trials. 2014 Feb 26;15:67. doi: 10.1186/1745-6215-15-67 (PMC3944007; doi:10.1186/1745-6215-15-67)
Supplement: Additional file 3 — Physical Therapy Training Manual. A training and reference manual that includes a description of the physical therapy protocol and instructions for using the treatment-based classification algorithm. [file 1745-6215-15-67-S3.pdf]

Physical Therapy Intervention Manual

A Guide for Physical Therapists

Back to Health Study

Boston Medical Center
Department of Family Medicine

Table of Contents

Introduction and Welcome 1

Low Back Pain 3

Physical Therapy for Low Back Pain 5

Fear Avoidance Beliefs in Chronic Low Back Pain 8

The Back to Health Study Design 9

Overview of Physical Therapy Study Intervention 11

Physical Therapy Treatment and Maintenance Phases 13

Physical Therapist Roles and Responsibilities 14

Scheduling and Attendance Policies and Procedures 16

Research Team Data Collection 17

Human Subjects Protection Training 18

Physical Therapy Online Training Modules 19

Online Module Training Access 21

Overview of Physical Therapy Treatment Phase 27

Overview of Physical Therapy Maintenance Phase 29

Classifying the Participant using the Treatment-Based Classification System 30

Fear-Avoidance Belief Questionnaire (FABQ) 36

Modified Oswestry Questionnaire (MOQ) 40

Initial Intake Form 41

Weekly Intake and Treatment Visits 46

Follow-Up Flow Sheet 49

Home Exercise Program 51

Assignment and Treatment: Maintenance Group and Home Practice Group 61

Appendix A: Forms and Questionnaires 62

Appendix B: Physical Therapy Examination Procedures 82

Appendix C: The Back Book Content Summary 86

Appendix D: Physical Therapy Terminology 87

Appendix E: Relevant Literature 88

Introduction and Welcome

Welcome to the Back to Health study! Chronic low back pain (cLBP) affects 5-10% of U.S. adults annually and costs over $50 billion per year in direct health care expenditures. Individuals from low-income, minority backgrounds are disproportionately impacted by cLBP due to disparities in access and treatment. Multiple cLBP studies and meta-analyses strongly support a moderate benefit for exercise therapy individually-delivered by a physical therapist, a reimbursed and well-established treatment to which physicians refer 22-38% of their low back pain patients. Several recent studies also suggest yoga as an effective treatment for cLBP. Yoga may also have other relevant benefits for cLBP patients, such as improved mood, stress reduction and lower health care costs. Education about self-care for back pain has also been tested in studies and has been found to be helpful. Education can be in various forms, such as provider counseling, written materials and support. However, no studies have directly compared yoga, physical therapy (PT) and education for the treatment of cLBP in any population—low-income, minority, or otherwise. Patients, providers, and health insurers need to know how well established treatments such as PT compare to complementary therapies such as yoga and commonly used self-care approaches such as education to ultimately reduce disparities in cLBP for minority populations. The Back to Health study was designed to address this important question. Back to Health is an NIH-funded comparative effectiveness randomized controlled trial for people from predominantly low-income minority backgrounds with cLBP. We will compare three treatment groups:

1. A standardized evidence-based clinical exercise therapy protocol individually delivered by a physical therapist
2. A standardized 12-week yoga protocol delivered in a class format
3. An education program that includes a comprehensive book on evidence-based self-care approaches for cLBP

The major outcomes of interest in the study are back pain intensity and function. Medication use, quality of life, psychological parameters, and cost effectiveness are some of the other important outcomes that will be measured.

As a member of the Physical Therapy Team, your participation in the study is extremely appreciated. Your commitment to providing the best possible physical therapy care to the study participants is critical for the success of the study. The physical therapy protocol for the study uses the Treatment Based Classification Method designed by Anthony Delitto, PhD, Chairman of the Department of Physical Therapy at the University of Pittsburgh. The protocol is a product of over 15 years of research and refinements,

and truly reflects evidence-based best practices. Adherence to the protocols described herein are essential for the study to be valid and the results generalizable. We acknowledge that physical therapists' preferences and treatment styles vary significantly and it may be difficult sometimes to follow a proscribed treatment protocol when you may think the patient would benefit from something different. For the purposes of this study, we ask you to please try to follow this protocol as closely as possible. As questions about the protocol, study, or logistics arise, please feel free to speak with anyone involved in the study including Dr. Delitto, Anisha Patel (PT Team leader), and Dr. Saper (Principal Investigator).

Again, thank you for your enthusiasm and dedication to the Back to Health study.

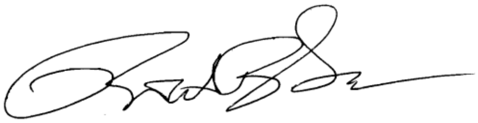

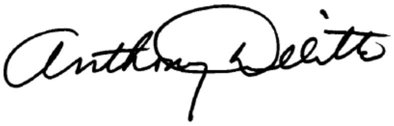

Robert B. Saper, MD MPH
Principal Investigator
Robert.Saper@bmc.org
(617) 414-6276

Anthony Delitto, PhD, PT, FAPTA
Physical Therapy Consultant
delitto@pitt.edu
(414) 383-6579

© Boston Medical Center

Low Back Pain

Low back pain (LBP) is the most common cause of pain in the United States, resulting in substantial morbidity, disability and cost to society. Approximately one-fourth of U.S. adults experience LBP at least one day over a three-month period. LBP accounts for 34 million office visits annually by family physician and primary care internists. Annual direct costs for LBP care in the U.S. are more than $50 billion and indirect costs (e.g., productivity) are estimated to be even greater. Back pain patients incur up to 75% more medical expenditures than patients without back pain. Back injury is the leading and most expensive cause of workers' compensation claims.

Health Disparities and Low Back Pain

The deleterious impact of LBP for people from low-income minority backgrounds is greater due to disparities in treatment and access. Although LBP prevalence in U.S. amongst whites, blacks, and Hispanics is similar, racial and ethnic disparities in access and treatment exist. Medical expenditures for LBP in minorities are 30% lower than for whites. For example, minorities with LBP receive less patient education, narcotic prescriptions, back surgery, specialty referrals, and intensive rehabilitation for occupational back injuries. Reasons for disparities may include lack of adequate health insurance, lower income, and less education, all factors associated with increased risk and severity of back pain. Attitudes and beliefs of providers and patients may also play a role. For example, physicians may assess a white patient's pain to be greater than a black patient's pain. However, the patient may perceive the opposite; the black patient may perceive her own pain greater than the white person perceives her pain. A history of experienced or perceived racial discrimination by a minority individual can also be associated with greater levels of back pain. Few intervention studies for LBP targeting minority populations have been conducted. *Although several studies have demonstrated racial and socioeconomic disparities in LBP treatment and outcomes, there is still a large need for LBP intervention trials that specifically target underserved populations.*

Non-Specific Chronic Low Back Pain

Chronic low back pain (cLBP) lasting more than 12 weeks affects an estimated 5-10% of U.S. adults. Physicians identify a definite anatomic source for the back pain in only a small minority of patients. Examples include a large herniated disc, spinal canal stenosis, or vertebral compression fracture. The majority of patients, however, are classified as having non-specific cLBP. Non-specific cLBP accounts for a majority of back-related health expenditures. The chronicity of pain for some LBP patients is

marked. For example, 100% of our pilot subjects reported cLBP for >1 year and one-third reported cLBP for >7 years.

However, few nonpharmacologic intervention studies for cLBP have included ongoing structured maintenance components beyond an initial 8-16 week initial intervention period. In a 2007 systematic review of 43 nonpharmacologic RCTs, only one exercise study contained a formal continuing exercise program for participants. Keeping chronic diseases such as diabetes and asthma well controlled requires a chronic disease management model with ongoing evaluation and care. Similarly, optimizing long-term outcomes for cLBP will likely also require an ongoing chronic disease management approach. *Therefore, cLBP studies need to evaluate not only long-term follow-up but also long-term models designed to maintain clinical effectiveness and support patient adherence.*

Treatment for Non-specific cLBP

Although there is a range of conventional pharmacologic, nonpharmacologic, and surgical procedures used for non-specific cLBP, most patients report only modest or moderate relief at best. In 2007, Chou and Huffman performed a systematic review and meta-analysis of nonpharmacologic treatments for back pain and authored evidence-based joint clinical practice guidelines on behalf of the American College of Physicians and American Pain Society for acute, sub-acute, and chronic low back pain. They recommended initial management for non-specific cLBP should include advice to remain physically active and education on back self-care. If necessary, medication with good evidence for benefitting cLBP, such as acetaminophen or nonsteroidal anti-inflammatory drugs, can be judiciously used. When needed, the guidelines recommend several conventional nonpharmacologic therapies, including exercise therapy and cognitive behavioral education. *Nonetheless, patient satisfaction with the effectiveness of conventional cLBP treatment is relatively low. Thus, there is a substantial need for research to identify more helpful therapies.*

Physical Therapy for Low Back Pain

Physical therapy (PT) comprises a group of health professionals that evaluate patients with musculoskeletal disorders and administer a myriad of interventions, some consistent with practice guidelines (e.g., active exercise approaches) and some inconsistent (e.g., passive modalities). Though considerable variation in care exists, there is some evidence that care administered by physical therapists that is consistent with practice guidelines is more cost-effective when compared with passive interventions. *Like many other areas of health care, a referral to PT may result in the provision of a wide variety of treatments, some supported by clinical guidelines and some not. We will address this issue in the Back to Health study by using only an evidence-based approach to represent "best practices" of PT for cLBP.*

Although exercise therapy for cLBP can be conducted in many formats and settings, it most commonly occurs through a physician referral to a physical therapist. A substantial proportion of patients with low back pain are referred to PT, especially by primary care doctors and orthopedists. Most PT visits are from physician referral because insurance companies typically reimburse the physical therapist only if prescribed by a physician. Analyses of the National Ambulatory Medical Care Survey show 22% of patients with mechanical low back pain seen by primary care physicians are referred to PT. Data from the Medical Expenditure Panel Survey determined that the annual mean expenditures on PT per respondent with spine problems increased from $115 in 1997 to $129 in 2005, and there was an estimated $4.3 billion total spent on PT for spine problems in 2005. Back problems comprise a significant portion of the conditions physical therapists commonly treat. A national survey of outpatient PT practices found that 26% of visits were for low back pain.

Data also suggest disparities in access to PT. Individuals with higher education are more likely and those with Medicaid are less likely to receive PT. *PT is the most common nonpharmacologic referral for cLBP made by physicians.*

Evidence for an Exercise-Based Physical Therapy Approach to Chronic Low Back Pain

There are several evidence-based clinical guidelines for the treatment of cLBP that provide guidance for physical therapists. The American Pain Society/American College of Physicians issued a clinical practice guideline finding good evidence that exercise therapy has a moderate effect on cLBP. Due to inconsistent or poor quality evidence, they were unable to recommend several passive therapies commonly used by PTs such as transcutaneous electrical nerve stimulation (TENS) or ultrasound. The Philadelphia

Panel Evidence-Based Clinical Practice Guidelines on Selected Rehabilitation Interventions for Low Back Pain as well as several European guidelines found compelling evidence for therapeutic stretching, strengthening, and mobility exercises. Systematic reviews and meta-analyses support these guidelines. A Cochrane review of 43 cLBP trials found strong evidence that exercise therapies are as effective as or more effective than other conservative treatments. Using Bayesian multivariable random-effects meta-regression techniques, they concluded the most effective exercise therapy strategy for improving cLBP was individually designed exercise programs delivered in a supervised format (e.g., home exercises with regular therapist follow-up) and that encouraging adherence to achieve high dosage, stretching and muscle-strengthening exercises were the best types of exercises for improving pain and function, respectively. *High dose, individually designed, PT-supervised exercise programs for cLBP consisting of stretching and strengthening exercises with home practice is broadly supported by evidence and clinical guidelines.*

Treatment-Based Classification System for cLBP

Many back pain researchers and clinicians argue that cLBP is a heterogeneous condition and therefore a “one size fits all” treatment approach will lead to unsatisfactory results. This has led to several attempts to subgroup patients, linking each subgroup to a specific treatment with the goal of identifying best management strategies. In 1995, Delitto et al. published one such classification system for the treatment of acute low back pain. This method, referred to as the Treatment-Based Classification System, was based upon empiric observations that patients with specific clusters of signs and symptoms could be classified into one of four categories: directional preference (flexion, extension, and lateral shift patterns), stabilization, manipulation, and traction. A specific set of interventions was suggested for each of the categories. Since Delitto’s original publication, several prospective RCTs suggest that patients treated by PTs according to this system had better functional outcomes than patients treated with a non-directional preference approach. Subsequent studies have focused on the validity of individual symptoms and signs for each subgroup and the effectiveness of specific treatments for each subgroup. Several studies have demonstrated excellent inter-rater reliability for specific examination signs related to the stabilization and specific exercise groups.

In 2006, Fritz et al. adapted Delitto’s original system using previous research to publish a modified treatment-based classification algorithm consisting of only three subgroups: directional preference, stabilization, and manipulation. RCTs using this modified system found greater decreases in disability than the use of a non-classification based approach. Moreover, physical therapists with and without extensive experience with the method could apply the classification system reliably without a difference in patient

© Boston Medical Center

outcomes. Only the directional preference and stabilization subgroups are described here because of their importance for cLBP treatment (manipulation is used for acute LBP episodes only).

Directional Preference: Patients in this subgroup exhibit the *centralization phenomenon*, which occurs when a clinically directed movement in a specific direction, such as lumbar flexion or extension, causes the patient's pain to rapidly decrease or move from a more peripheral location (e.g., buttocks) to a more central location (midline of the lumbar spine). Conversely, movement in the opposite direction may cause an increase in pain intensity or movement of pain to a more peripheral location. The DP-Extension pattern is more common and centralizes with extension and is treated with repeated end-range extension exercises. Conversely, patients with the DP-Flexion pattern are prescribed end-range flexion exercises.

Stabilization: LBP in this subgroup is believed to be from spinal instability as a result of weak spinal muscles (transversus abdominus, erector spinae/multifidus, quadratus lumborum, oblique abdominals). For the stabilization category, a patient must meet at least three of the following criteria:

1. Age <40 years old
2. Passive straight leg raise range of motion >91°
3. Aberrant movement pattern during trunk flexion
4. A positive prone instability test

Stabilization patients are most likely to benefit from lumbar muscle strengthening and stabilization exercises.

Fear Avoidance Beliefs in Chronic Low Back Pain

The Fear-Avoidance Model of Exaggerated Pain Perception introduced in 1983 divides pain perception into two components: the physiologic response to a painful stimulus and an emotional component related to fear of pain. Waddell developed the Fear-Avoidance Belief Questionnaire (FABQ), a reliable and valid instrument for measuring the degree that fear of pain impacts a person's avoidance of physical activity (measured by the FABQ-PA subscale score) or work (measured by the FABQ-W subscale score). By avoiding physical activity, pain and function further worsen. This vicious cycle of fear of movement-causing pain and consequent inactivity leading to greater disability is illustrated below.

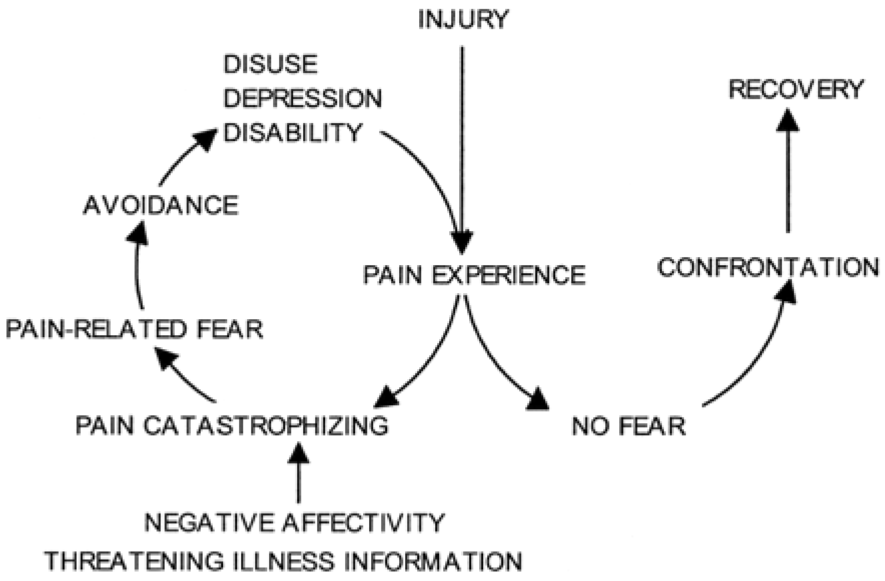

Source: Vlaeyen JWS, Linton SJ. Fear-avoidance and its consequences in chronic musculoskeletal pain: a state of the art. Pain 2000;85:317–332.

Secondary analyses of FABQ scores from physical therapy RCTs have found an elevated FABQ Work subscale score (≥29) is a strong predictor of poor prognosis, including ability to return to work. Early identification of elevated FABQ scores can trigger the PT to initiate appropriate cognitive behavioral interventions, including educational materials and a graded exercise program. RCTs have demonstrated the efficacy of these interventions for reducing fear-avoidance beliefs and improving outcomes.

Studies have shown that patients with low back pain get maximal positive effect when physical therapists combine (1) simple cognitive behavioral education for a high fear avoidance patient, (2) strengthening and stretching exercises based on the treatment-based classification approach, and (3) aerobic exercise as appropriate.

The Back to Health Study Design

The Back to Health study is a 52-week comparative effectiveness randomized controlled trial of individually-delivered physical therapy (PT), once per week yoga classes, and an educational book on self-care for cLBP in 320 individuals from predominantly minority backgrounds recruited from Boston Medical Center and affiliated community health centers. The 52-week trial starts with an initial 12-week Treatment Phase followed by a 40-week Maintenance Phase.

For the 12-week Treatment Phase, participants are randomized in a 2:2:1 ratio into (1) a standardized evidence-based exercise therapy protocol individually delivered by a physical therapist and supplemented by home practice; (2); a standardized once per week hatha yoga class supplemented by home practice; and (3) education delivered through a self-care book. The study co-primary endpoints are mean pain intensity over the previous week measured on an 11-point (0-10) numerical rating scale and back-specific function measured using the 23-point modified Roland Morris Disability Questionnaire.

For the 40-week Maintenance Phase, PT participants will be re-randomized in a 1:1 ratio to either a structured ongoing maintenance PT program or a home practice group. The structured ongoing maintenance PT group will have five “booster” sessions at 18, 26, 32, 40, and 46 weeks. The home practice group will be encouraged to continue their home practice exercises at home and will not receive any additional sessions. Similarly, yoga participants will be re-randomized in a 1:1 ratio to either a structured ongoing maintenance yoga program (once per week classes) or a home practice group. Education participants will be encouraged to continue to review and follow the recommendations of their educational materials.

The study flow diagram on the next page illustrates the overall study design.

Treatment Phase

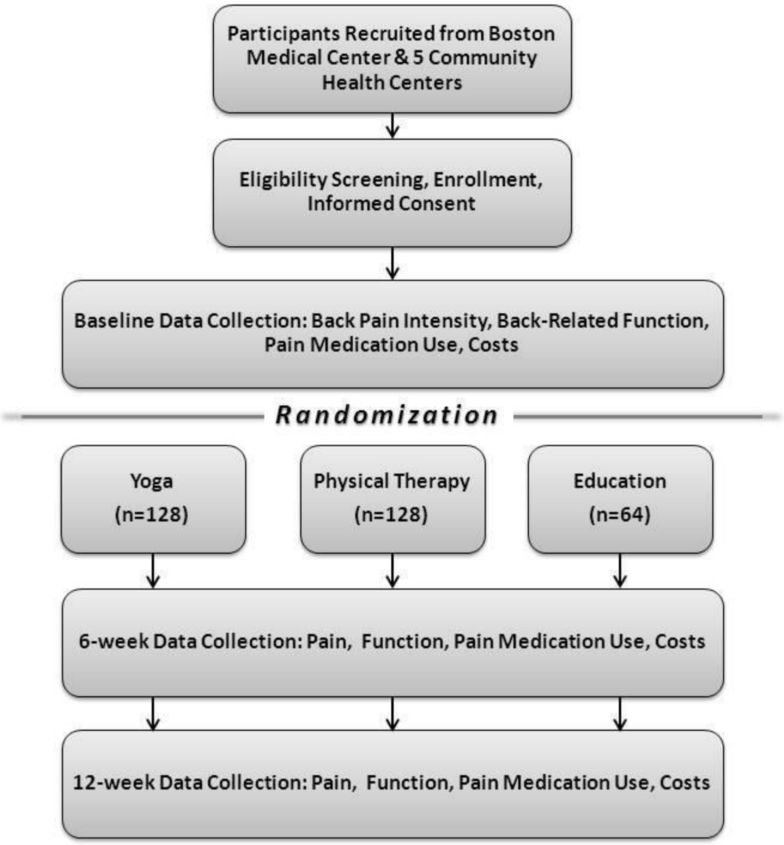

Maintenance Phase

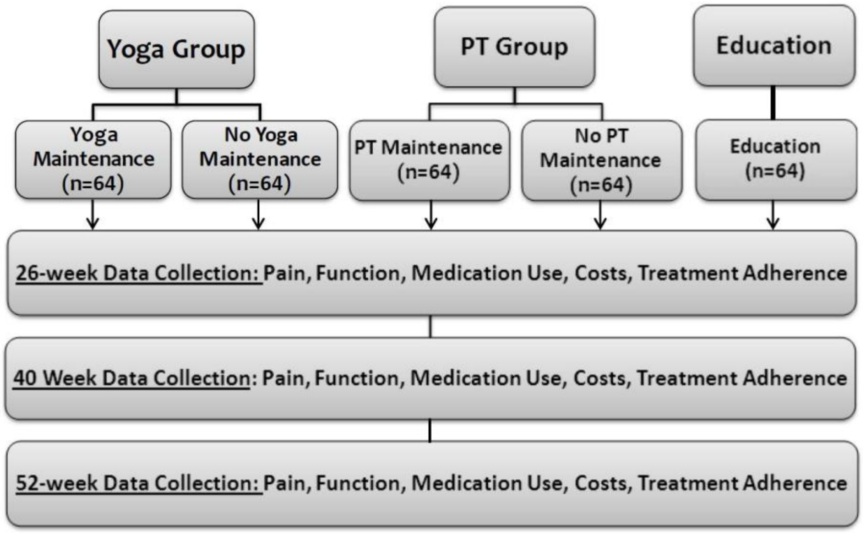

© Boston Medical Center

Overview of Physical Therapy Study Intervention

Participants randomized to the PT treatment intervention will receive 15 total sessions (1 initial intake/classification visit + 13 treatment visits + 1 discharge visit) during the 12-week Treatment Phase of the study.

- PT Session schedule:
  - Week 1: Intake Visit
  - Weeks 2-4: Two treatment visits per week
  - Weeks 5-11: One treatment visit per week
  - Week 12: Discharge visit
- The participant is classified according to the Treatment Based Classification System at the initial intake visit. Their classification is continually reassessed throughout the Treatment Phase (at least once per week). Since the patient population has cLBP, most participants are expected to fall into the stabilization category. For those who fall into the directional preference category, the physical therapist should attempt to help participants progress to the stabilization classification category over the course of the Treatment Phase.
- All in-person PT sessions utilize exercises designed for the stabilization classification group, unless the participant develops true radicular pain.
- Participants are assessed for fear avoidance beliefs using the Fear Avoidance Beliefs Questionnaire (FABQ). Participants exhibiting elevated risk of fear avoidance beliefs, as indicated by a FABQ-Work subscale score equal to or greater than 29, receive three additional interventions:
  1. *The Back Book*, a brief educational pamphlet that presents principles which counter fear avoidance beliefs
  2. Coaching from the physical therapist using principles that counter fear avoidance beliefs (see page 36)
  3. A graded aerobic exercise program which progresses incrementally until target heart rates are achieved for specified durations
- A home exercise program (HEP) is assigned according to weekly classification by the physical therapist.
  - General recommendations of home exercises for participants in the stabilization group are 30 minutes on days when not attending PT session.
  - Participants in the directional preference groups (extension or flexion) will be encouraged to perform home exercises as frequently as needed in order to address and control their symptoms, at least twice per day, and as best as the participant is able to complete them.
- A home exercise program (HEP) is reviewed at each visit to address compliance and progression. Participants are requested to fill out a daily HEP log to record

the completion of PT exercises completed on their own and submit the log to the physical therapist on a weekly basis.

- Upon discharge at the 12-week PT visit, all participants undergo a physical examination. In addition, a home exercise program is reviewed with each participant and a written description of these exercises is provided to the participant.
- Within days of the final visit of the 12-week Treatment Phase and corresponding 12-week data collection, PT participants are notified as to whether they have been randomly assigned to a PT Maintenance Group or Home Practice Group.
    - The PT Maintenance Group will meet with the physical therapist for five additional sessions over the remaining 40 weeks of the study. These sessions occur on or around 18, 26, 32, 40, and 46 weeks.
    - The Home Practice Group is encouraged to continue doing their home practice program, as prescribed by the PT at the 12-week visit, and will receive no additional sessions.

Physical Therapy Treatment and Maintenance Phases

12-week Treatment Phase

- One-on-one session with a physical therapist
- 15 sessions: Intake + Weeks 2-4 twice per week + Weeks 5-12 once per week
- Participants will complete Fear Avoidance Beliefs Questinonnaire (FABQ) and Modified Oswestery Questionnaire

  Physical therapist calculates score

  BMC research staff provide score in advance
- Participants receive Home Exercise Program (HEP) assigned by physical therapist
- Participants are encouraged to practice at home. Physical therapy supplies will be provided at no cost.

40-week Maintenance Phase

- Participants assigned to Maintenance Group or Home Practice Group
- Maintenance Group

  5 "booster sessions" scheduled at 18, 26, 32, 40 and 46 weeks
- Home Pratice Group

  Discontinue in-person PT sessions

  Participants are encouraged to complete exercises at home over 40-week Maintenance Phase

© Boston Medical Center

Physical Therapist Roles and Responsibilities

<u>Study-Specific Training</u>

All physical therapists on the PT Study Team have substantial professional experience in treating patients, specifically those with low back pain. Training specifically for participating on the PT Study Team therefore is mostly focused on learning the specific treatment protocols used. These may be more or less familiar to each therapist depending upon their own educational background and professional experience. Study-specific trainings each physical therapist must complete to participate in the study include:

1. Completing a set of web-based educational modules that have been developed by the University of Pittsburgh to train therapists in the Treatment Based Classification System. These modules include basic information about the system, links to additional resources and research, and quizzes to demonstrate that you have acquired the presented knowledge. More information on how to access the web modules and their content can be found on page 19.
2. Reading this training manual thoroughly.
3. Attending study-specific trainings in the protocol led by Anisha Patel, Dr. Delitto, or others.
4. Reading and becoming familiar with the themes presented in *The Back Book*, a small pamphlet that underscores the principles behind overcoming fear avoidance behaviors. This is the pamphlet that will be given to PT participants who have elevated scores on the FABQ-W subscale.
5. Training in Human Subjects Protection—Federal laws governing the conduct of human subject research mandate that all study personnel having contact with study participants undergo training in human subjects protection. Information on how to access either of these trainings can be found on page 18.

Please contact one of the following Back to Health Study research staff for further assistance:

Julia Keosaian, MPH, Senior Research Assistant
Julia.Keosian@bmc.org
617.414.6228

Chelsey Lemaster, Research Assistant
Chelsey.Lemaster@bmc.org
617.414.6201

As you progress through these training materials, please note any questions you may have. These can be discussed with the PT Team Leader Anisha Patel, our PT Consultant Dr. Delitto, the Principal Investigator Dr. Saper, or others.

Data Collection

The vast majority of "data" for this study will be collected by the study staff and not the physical therapists. Data collection by the research staff usually occurs *before* the onset of the PT session scheduled for that day and is conducted in such a way to minimize any interference to the physical therapists. However, we do ask participants to fill out a daily Home Exercise Program (HEP) log to record the completion of PT exercises completed on their own and submit the log to the physical therapist on a weekly basis. You should review these at each visit to address compliance and progression of participant. Physical therapists should encourage participants to complete logs truthfully.

Scheduling and Attendance Policies and Procedures

Scheduling

- Research staff (PT Coordinator) schedules participant appointments.
    - Research staff will encourage participants to arrive 15-20 minutes before their appointment time in order to complete study-related paperwork before each session, including the Fear Avoidance Beliefs Questionnaire (FABQ) and the Modified Oswestry Questionnaire (MOQ).
- All efforts are made for participants to work with the same physical therapist at each appointment whenever possible.
- In the case of a cancelled or missed appointment, the primary physical therapist notifies the research team's PT Coordinator and the research team contacts the participant to reschedule the missed PT session.
    - However, in the instance where a participant is late to an appointment (5-10 minutes), a reminder call is administered.
    - If the participant arrives late to his or her appointment, the physical therapist will do his or her best to accommodate the participant and complete a PT session as time allows, while prioritizing participants who adhered to their scheduled appointment time.
    - In instances where the participant does not attend an appointment, the Back to Health PT Coordinator contacts the participant to reschedule.
- Initial intake PT sessions are separated by 60 minutes between scheduled participants.
- All subsequent PT sessions are separated by 30 minutes between scheduled participants.
- Sessions are approximately 45-60 minutes and are divided into 30 minutes working directly with an individual physical therapist followed by up to 30 minutes of aerobic exercise.

Attendance

- Participants complete a sign-in sheet at their treatment site.
- Research staff collect the sign-in sheets and reschedule any missed appointments for a designated rescheduling time.
    - PT sessions must be separated by at least one day between sessions.

Research Team Data Collection

Data Collection

At the beginning of the following sessions, physical therapists may receive paper copies of the Fear Avoidance Belief Questionnaire (FABQ) and/or Modified Oswestry Questionnaire (MOQ) from the participants:

- Week 6
- Week 12
- Week 18
- Week 32
- Week 46

The research team will collect data in survey format at the following time points:

- Baseline (Week 0)
- Week 6
- Week 12
- Week 18 (Maintenance Group)
- Week 26 (Maintenance Group & Home Practice Group)
- Week 30 (Maintenance Group)
- Week 40 (Maintenance Group & Home Practice Group)
- Week 46 (Maintenance Group)
- Week 52 (Maintenance Group & Home Practice Group)

A member of the research team will retrieve data from the PT offices as needed.

© Boston Medical Center

Human Subjects Protection Training

Human Subjects Protection training can be achieved in one of two ways:

1. Completing an online training sponsored by the National Institutes of Health. This takes approximately 2 hours.

2. Participate in an in-person training offered at Boston University Medical Campus. These are offered periodically and can be from 2-4 hours in length.

Information about NIH Research Certification and how to obtain certification can be found under the "Research Certification" section on the following website: http://www.bumc.bu.edu/ocr/certification/

National Institutes of Health

Online training through the National Institutes of Health (NIH) can be obtained through the following website: http://phrp.nihtraining.com

Boston University Medical Campus Clinical Research

Boston University Medical Campus hosts in-person trainings in the Evans Bio-Medical Research Building (650 Albany Street, 7th floor, room 714). An RSVP is required to attend a seminar and can be given either by calling the Office of Clinical Research or emailing irbtemp@bu.edu.

The contact information for the Office of Clinical Research and the on-site training schedule can be found on the following website: http://www.bumc.bu.edu/ocr/certification/training/

Physical Therapy Online Training Modules

Course layout: Eight modules are presented sequentially such that the introductory modules are followed by the more advanced modules. Each module begins with a pre-test. The course is then presented in form of statement summaries as well as pre-recorded online lectures. Upon completion of the module, the therapist completes a post-test to evaluate knowledge gained. The pre- and post-test questions are the same for each module and are in objective multiple choice format. The pre- and post-test evaluations serve to identify gaps in knowledge prior to reviewing the modules and to provide information to focus on while reviewing the module. In order to have successfully completed each module, a passing score of 80% is required on the post-test. If this score is not achieved, the physical therapist will be required to retake the module before proceeding to the next module.

Learning Objectives: The learning objectives for the course emphasize that upon completion, the therapists will be able to identify patients eligible for management with manipulation, stabilization, bio-behavioral intervention (AKA cognitive behavioral education), traction or directional preference exercises and tailor treatment protocols for patients classified to each of these treatments.

Continuing Education Credits: The Commonwealth of Massachusetts does not require continuing education credit for license renewal. In other states, the standards may differ.

<u>Online Module Course Contents</u>

Introductory Module 1: "Clinical Decision Making Paradigm" is an overview of the clinical decision making paradigm for management of LBP.

Introductory Module 2: "Key Elements in History Taking" is a review of the key elements in the history of the patient's episode of LBP that are helpful in developing a preliminary hypothesis regarding presence of serious underlying pathology as a source of the LBP and establishing patients' eligibility for PT treatment. A hypothetical case is presented to review this material.

Introductory Module 3: "Neurological Assessment" is an overview of assessments conducted to rule out neurological involvement in patients with LBP.

Introductory Module 4: "Low Back Pain Classification" is an overview of the treatment-based classification approach to LBP. Information regarding criteria for

staging patients with LBP based on severity of symptoms and further classification of Stage I patients into sub-groups is presented.

Advanced Module 5: "Mobilization" presents criteria (i.e., Clinical Prediction Rule) for identification of patients eligible for spinal manipulation. Evidence from the literature that supports the use of manipulation for the identified subgroup is presented. Generally, in the Treatment Based Classification system, manipulation is used for acute low back pain episodes. Our study's focus is on chronic low back pain. Participants need to have had their pain for 12 weeks or more to qualify for the study, and the majority of participants have had low back pain for a year or more. Thus, mobilization would typically not be used as part of the treatment administered in this study.

Advanced Module 6: "**The Stabilization Classification"** presents criteria for identification of patients eligible for spinal stabilization exercises. Evidence to support use of spinal stabilization for the identified subgroup is presented. A hypothetical case is presented to review this material and a protocol for use of the technique is suggested.

Advanced Module 7: "**The Directional Preference Classification"** presents criteria for identification of patients eligible for directional preference exercises. Evidence to support use of directional preference exercises for the identified subgroup is presented. A hypothetical case is presented to review this material and a protocol for use of the technique is suggested.

Advanced Module 8: "**The Bio-behavioral Assessment"** presents criteria for identification of patients that exhibit high fear avoidance beliefs and would likely benefit from bio-behavioral exercises (AKA cognitive behavioral education). Fear-avoidance beliefs are measured by the Fear-Avoidance Beliefs Questionnaire (FABQ). The explanation and meaning of scores from the FABQ are discussed. Evidence to support use of bio-behavioral exercises for the identified subgroup is presented.

Online Module Training Access

The University of Pittsburgh hosts online training modules which describe the fundamentals of the treatment based classification system for low back pain. Prior to providing care to study participants, physical therapists should complete the online training modules and the competence assessment at the conclusion of each section. There are eight modules on LBP to complete and at the end of each module you will receive an online certificate.

This is a guide to help you create an account on the University of Pittsburgh Health Sciences eLearning website.

1. Go to cme.hs.pitt.edu/ISER/servlet/IteachControllerServlet?actiontotake=displaymainpage. On the right hand side of the screen, click on the first bullet point that says "**Create Account.**"

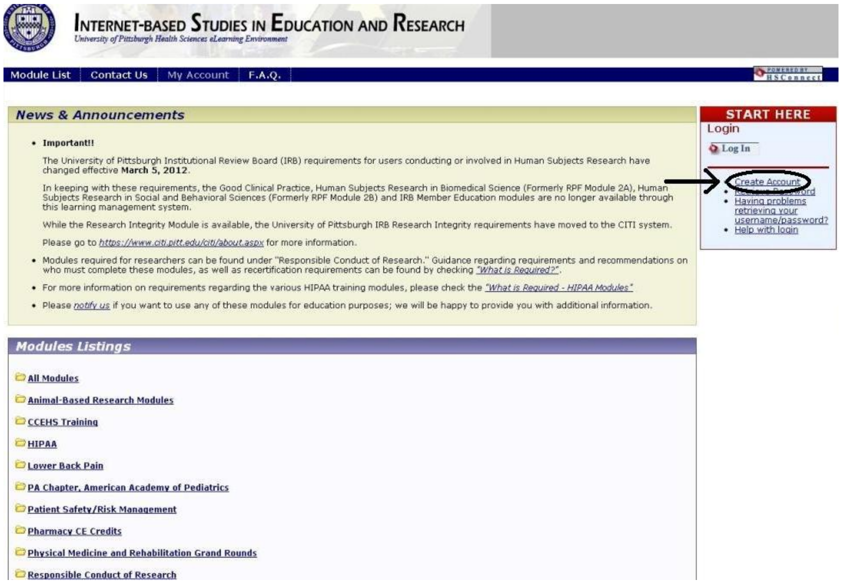

2. To create an account, enter your email address in the first box. Your email address will be your username.

   In the next box, choose a password, and confirm that password by typing it again in the last box. Click **Next**.

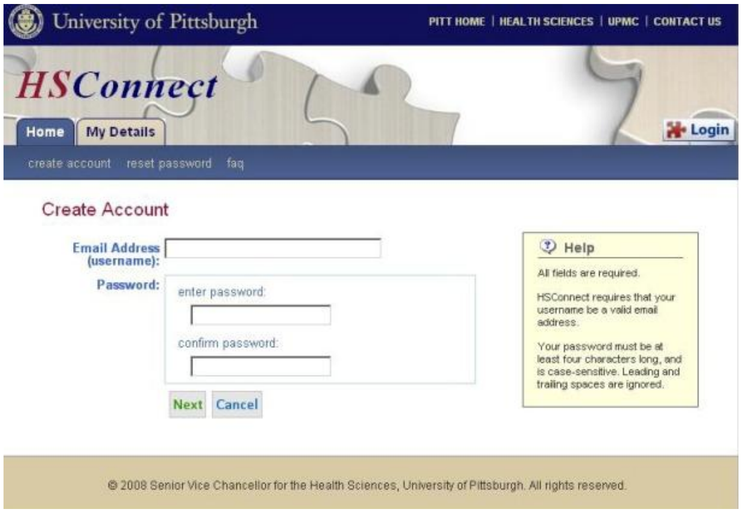

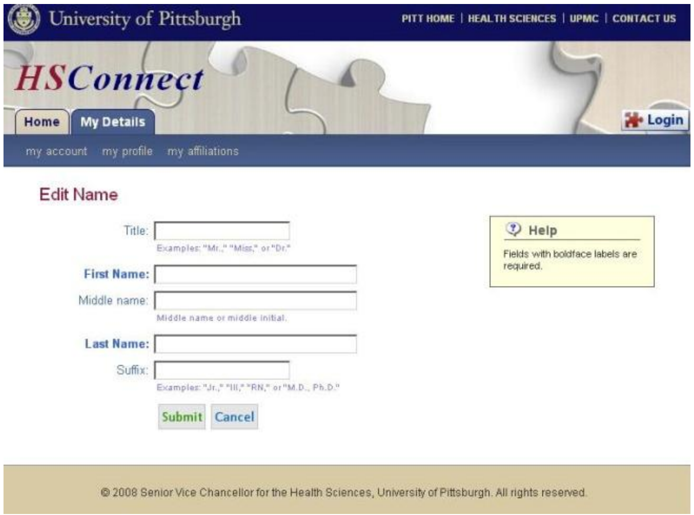

3. Enter your information in the boxes on the "Edit Name" page. Click **Submit**.

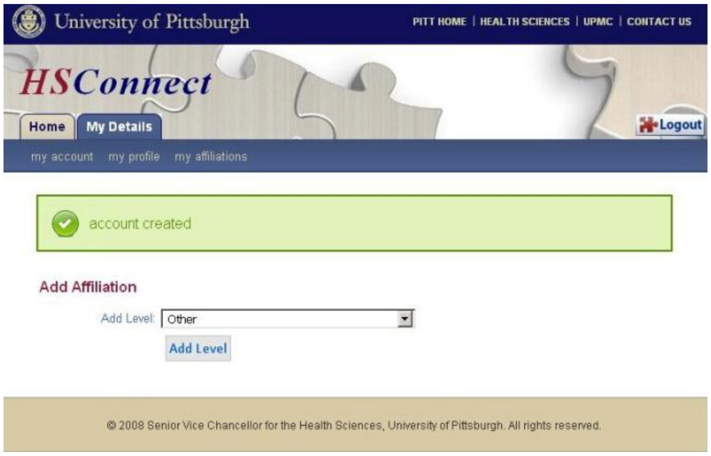

4. You should now see a green box that says "account created." From the drop-down menu on the Affiliation page, choose "Other." Then, click **Add Level**.

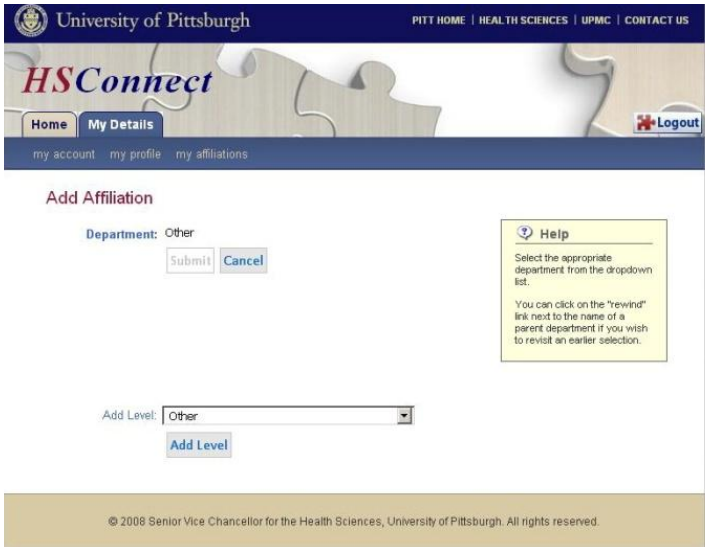

5. A second Affiliation page will come up on the screen. You should see a drop-down menu at the bottom of the page that says "Add Level." Choose **Other** and click **Add Level**.

6. On the next page, the Department should read “Other” twice. Click **Submit**.

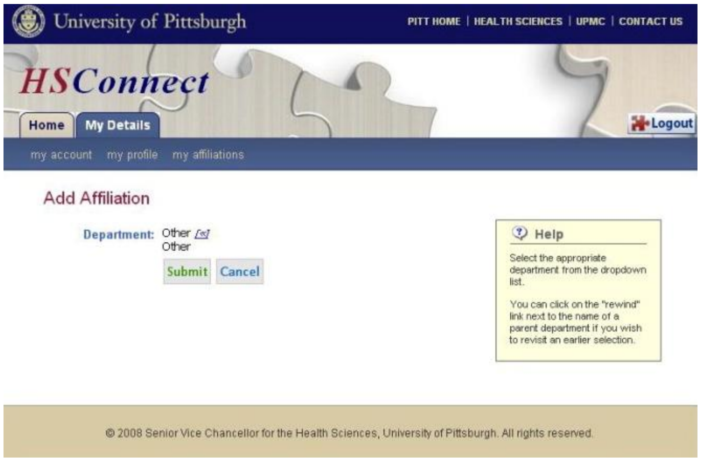

7. On the next page, there should be a green box that reads “Department saved successfully.” Click on the **Home** tab at the top left-hand side of the screen.

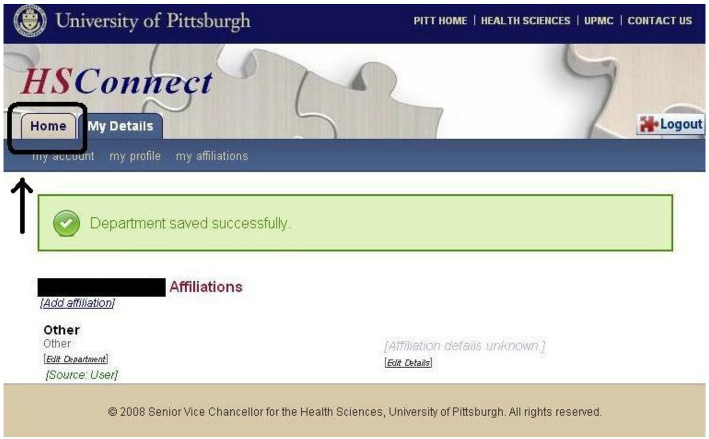

8. Under “Tools” section, select **Internet-based Studies in Education and Research**.

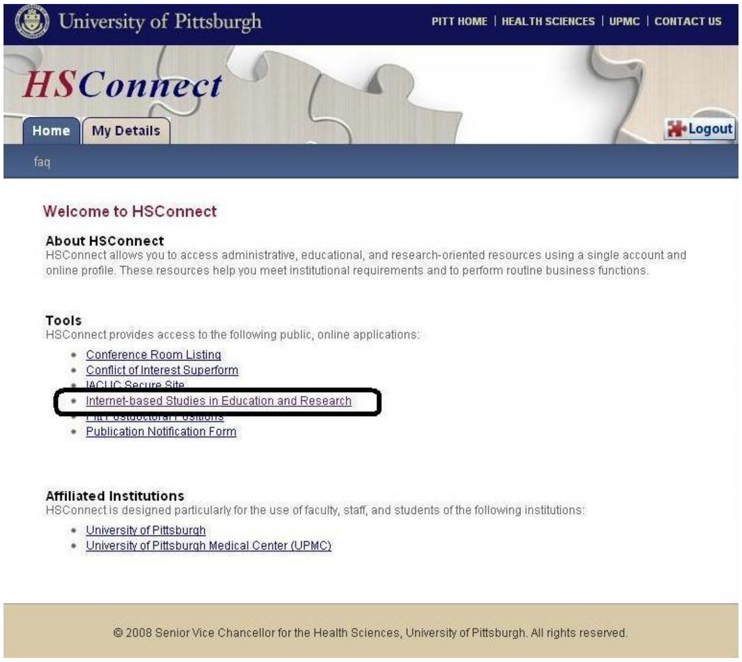

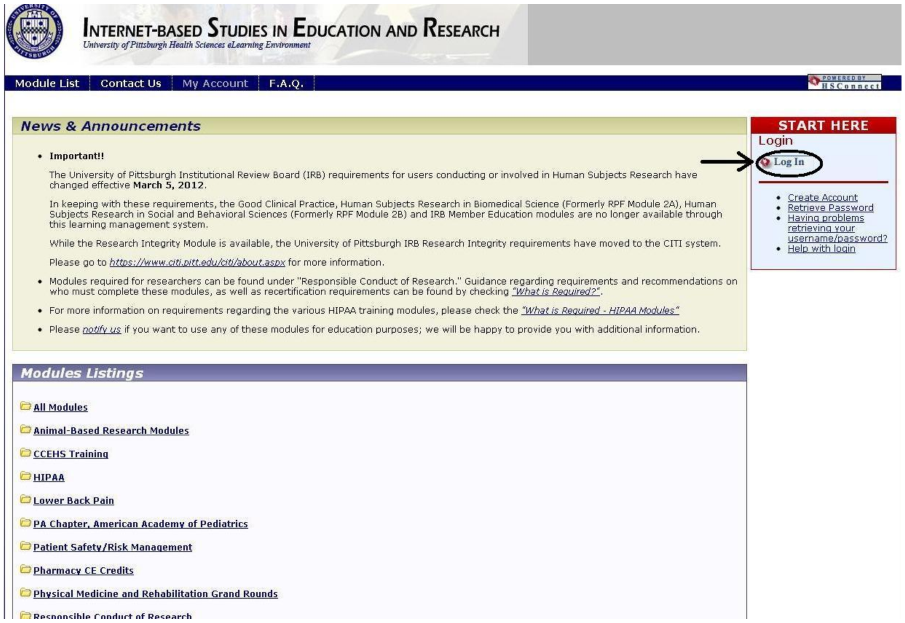

9. On the right hand side of the screen, click **Login.**

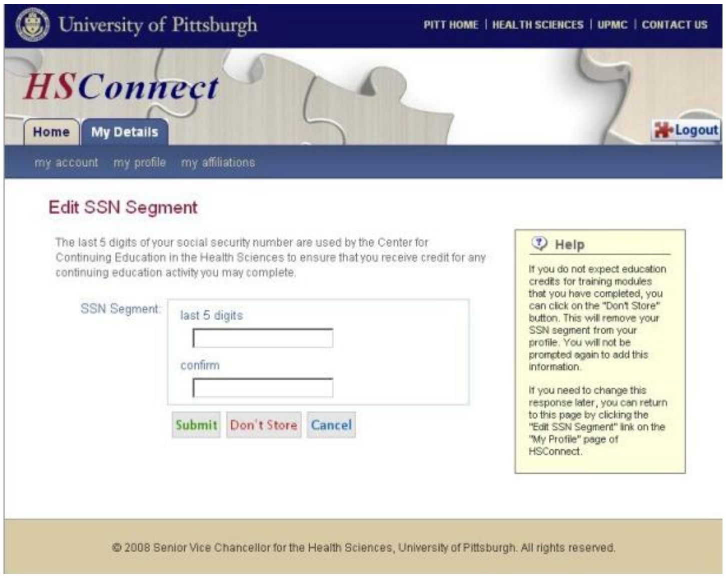

10. Now you should see a page called "Edit SSN Segment". In the first box, type in the last 5 numbers of your social security number. In the second box, type in the last 5 numbers of your social security number again to confirm.

    If you choose to keep this number stored on your account, click **Submit**. If you don't want it stored on your account, click **Don't Store.**

11. Now you should see a green box that says “SSN segment stored by user’s request.” Return to the list of modules by clicking **Home**, and then clicking **Internet-based Studies in Education and Research.** (This is the same as repeating steps 7 and 8).

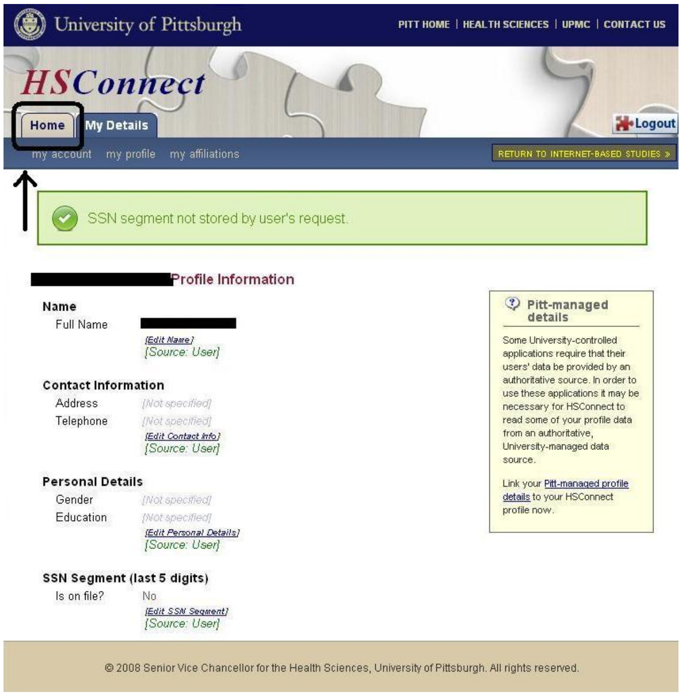

12. On the Home page, scroll down to where it says “Modules Listings.” Click on the folder that says **Lower Back Pain**. A list of 8 modules will appear. To begin the first module, click on **Low Back Pain: Course 1.**

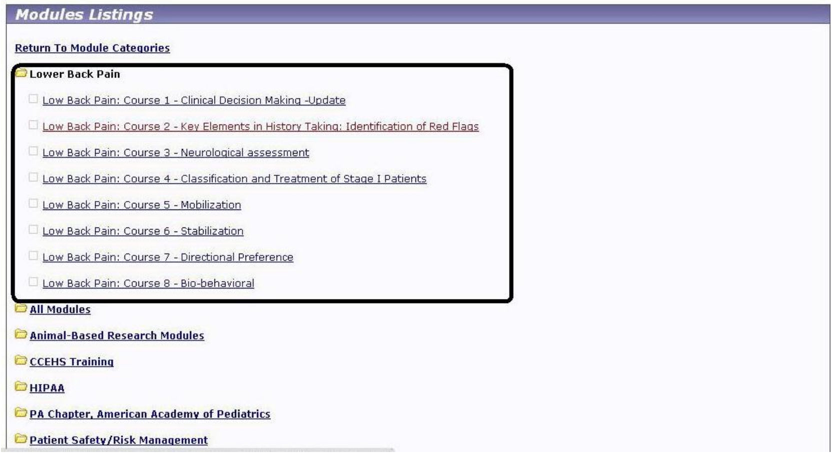

13. You are now ready to begin your first course! When you are finished reading through each section, click **Next** at the bottom of the screen. Use the “Step by Step” menu on the left side of the screen to go back to other sections if needed.

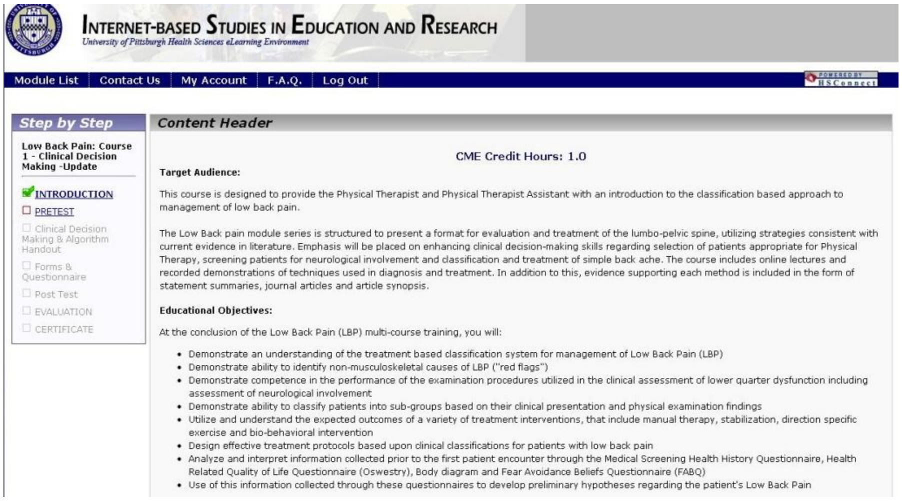

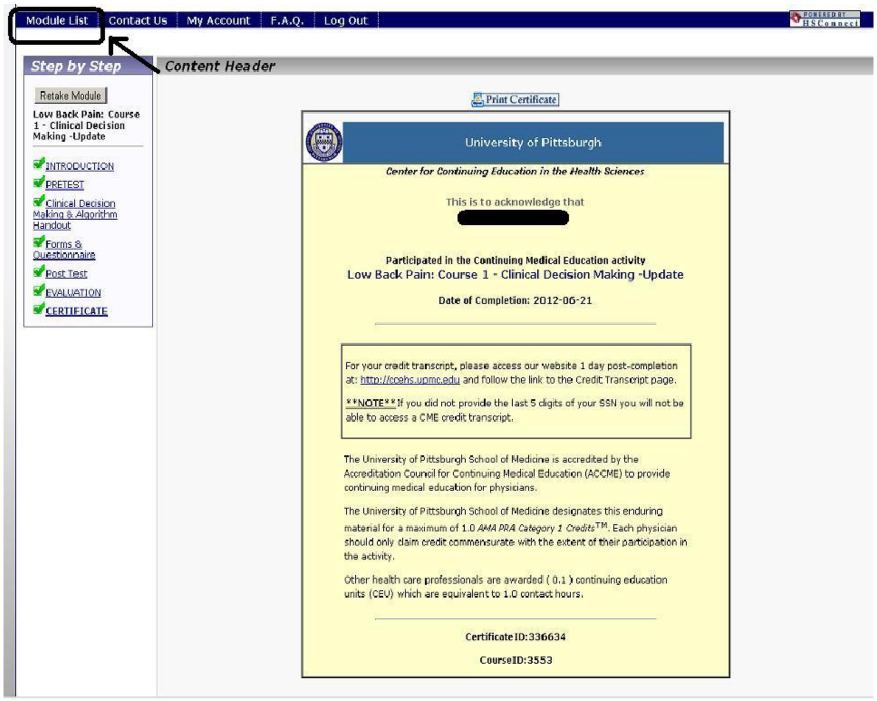

14. When you finish completing each section of the module, you will receive a certificate. You print out the certificate by clicking **Print Certificate** at the top of the screen, or you can print it out later. To continue on to the next module, click **Module List** at the

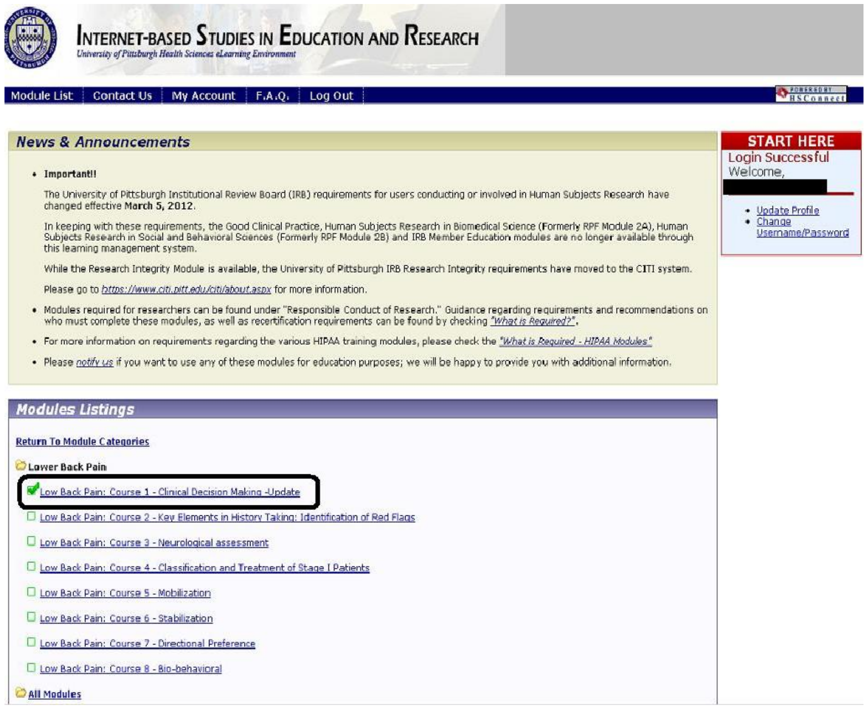

15. A green check mark next to the module indicates that you have completed that module. Continue the training by completing all eight of the modules listed under “Lower Back Pain.”

Overview of Physical Therapy Treatment Phase

Week 1

- 1-hour Intake
- Before Session
  - Review session schedule
    - If participant is late for appointment, provide reminder call
  - Review FABQ and Oswestry Scores
- During Session
  - Classification
  - Intake Form
  - Discuss adverse events and general concerns of participant
  - Implement behavioral intervention as indicated by FABQ score
- After
  - Weekly notes
  - Weekly meeting with PT research staff

Weeks 2-4

- 2 weekly sessions (1-hour)
- Before Session
  - Review session schedule
    - If participant is late for appointment, provide reminder call
- During Session
  - Reclassification
  - Intake Form
  - Discuss adverse events and general concerns of participant
- After
  - Weekly notes
  - Weekly meeting with PT research staff

Week 5

- 1 weekly session (1-hour)
- Before Session
  - Review session schedule
    - If participant is late for appointment, provide reminder call
- During Session
  - Reclassification
  - Intake Form
  - Discuss adverse events and general concerns of participant
- After
  - Weekly notes
  - Weekly meeting with PT research staff

© Boston Medical Center

Week 6

- 1 weekly session (1-hour)
- Data collection (research staff)
- Before Session
  - Review session schedule
    - If participant is late for appointment, provide reminder call
  - Review FABQ and Oswestry Scores
- During Session
  - Reclassification
  - Intake Form
  - Discuss adverse events and general concerns of participant
  - Implement Behavioral Intervention as Indicated by FABQ score
- After
  - Weekly notes
  - Weekly meeting with PT research staff

Weeks 7-11

- 1 weekly sessions (1-hour)
- Before Session
  - Review session schedule
    - If participant is late for appointment, provide reminder call
- During Session
  - Reclassification
  - Intake Form
  - Discuss adverse events and general concerns of participant
- After
  - Weekly notes
  - Weekly meeting with PT research staff

Week 12

- 1 weekly session (1-hour)
- Data collection (research team)
  - End of 12-week Treatment Phase
- Before Session
  - Review session schedule
    - If participant is late for appointment, provide reminder call
  - Review FABQ and Oswestry Scores
- During Session
  - Reclassification
  - Intake Form
  - Discuss adverse events and general concerns of participant
  - Implement Behavioral Intervention as Indicated by FABQ score
- After
  - Weekly notes
  - Weekly meeting with PT research staff

Overview of Physical Therapy Treatment Maintenance Phase

Months 4, 6, 8, 10, & 12

- Maintenance booster session (1-hour)
- Before Session
  - Review session schedule
    - If participant is late for appointment, provide reminder call
  - Review FABQ and Oswestry Scores
- During Session
  - Reclassification
  - Intake Form
  - Discuss adverse events and general concerns of participant
  - Implement behavioral intervention as indicated by FABQ score
- After
  - Weekly notes
  - Weekly meeting with PT research staff

Week 52

- No session
- Final data collection by research team

Classifying the Participants using the Treatment-Based Classification System

Initial intake sessions are 60 minutes long and are not scheduled concurrently with other appointments. Generally, initial intake is structured as follows:

Introduction to Participant

- Explain your role as a physical therapist and discuss treatment and goals
- Answer any questions participant has

Initial Intake

- Initial Intake Form
  - Demographic Information
  - Medical History

Physical Examination

- Deep Tendon Reflexes
- Motor Examinations
- Sensory Examination
- SLR
- Prone Instability Test
- Mobility Test
- Directional Preference Evaluation
- Abberant Movements
- Prone Instability Test

Treatment Classification

- Classify participant by group
  - Stabilization or directional preference (flexion or extension)
- Classify participant into Stage I or Stage II to determine whether the participant will receive general conditioning and/or aerobic exercise

Fear-Avoidance Belief Status

- Score provided by research staff
- Implement intervention if necessary (score ≥ 29)

PT-Guided Treatment Session

- Stabilization exercises or Directional Preference exercises
- Aerobic exercise

Treatment Classification

The first step in the classification scheme includes confirmation that is it safe for the participant to receive PT treatment. Although the research study staff will make all efforts during the screening and enrollment process to assure the participant has non-specific low back pain (LBP) only, it is important for the physical therapist to verify the participant's status and the absence of worrisome *red flags.* Examples of non-musculoskeletal causes of LBP include cancer, fracture, and infection. These participants typically present with signs and symptoms that, when present, should be recognized as *red flags*.

It is particularly important to identify a cluster of signs and symptoms that collectively point towards a non-musculoskeletal cause of LBP, as one isolated abnormal clinical variable may not indicate a serious pathology. If *red flags* are identified during the participant intake, the PT supervisor and the Principal Investigator (PI), Dr. Saper, should be notified immediately. *Red flags* of particular concern for participants with LBP are listed in the following chart.

**Red Flags for Potentially Serious Conditions Causing Low Back Pain**	
**Syndrome**	**Red Flags**
Spinal Fractures	• Major trauma, such as a motor vehicle accident (MVA), fall from a height or a direct blow to the spine<br>• Compression Fracture<br>→ Minor trauma or strenuous lifting in older or potentially osteoporotic individuals<br>→ Prolonged corticosteroid use<br>• Stress Fracture<br>→ Persistent back pain in younger individuals involved in repetitive hyperextension activities
Cauda Equina Syndrome	• Saddle numbness<br>• Recent onset of fecal incontinence or urinary retention<br>• Serious or progressive neurologic deficit in the lower extremities<br>• Weakness in limbs and/or gait abnormality
Neoplastic Conditions	• Age older than 50 years<br>• History of cancer<br>• Unexplained weight loss<br>• Worsening pain, especially at night
Ankylosing Spondylitis	• Age of less than 35 years at onset<br>• Insidious onset<br>• Improvement of pain with exercise<br>• No improvement of pain with rest<br>• Pain at night (with improvement upon arising or walking)
Spinal Infection	• Recent fever and chills<br>• Recent bacterial infection, IV drug use, immune suppression (from steroids, transplant, or HIV)

In order to determine whether the PT treatment is safe to administer to the participant, a neurological screening is performed during the intake portion of the initial session. The neurological screening includes a brief sensory examination, deep tendon reflexes, and a motor examination. Physical therapists should alert research staff of participants who show symptoms consistent with significant neurological deficits.

The second step of classification of participants' LBP is to classify them as Stage I, II or III by the severity of their symptoms and disability score on the Oswestry Disability Index.

Stage I

By definition, because participants in this study have chronic LBP they should be entering with LBP that is classified as Stage II. However, participants may experience a flare-up of back pain and therefore may fall into the Stage I category. Generally, low back pain that is classified as Stage I is acute. Participants with Stage I LBP report inability or difficulty in performing basic functions such as sitting, standing and walking.

Generally, the participant will be unable to perform any of the following:

- Sit for more than 30 minutes
- Stand for more than 15 minutes

Oswestry scores of participants classified as Stage I will range from 40-60% or greater at baseline. The primary goals of PT treatment for Stage I participants are pain modulation and functional restoration (Oswestry <40%).

Interventions for Stage I Participants

- Directional Preference (Flexion or Extension)
- Bio-behavioral Graded Exercises
- Cognitive Behavioral Education

Stage II

Generally, low back pain that is classified as Stage II is subacute. The lower back muscles can be conditioned to be reactivated and the participant will be able to resume normal activities. Participants with Stage II low back pain report the inability to perform activities of daily life (ADLs) such as vacuuming, cleaning, lifting and mowing the lawn.

Oswestry scores of participants classified as Stage II will range from 20-40% at baseline assessment. The primary goals of PT treatment for Stage II participants are pain modulation, elimination of physical impairments and re-activation into full participation.

Interventions for Stage II Participants

- Improving Flexibility
- Increasing Strength and Endurance
- Improving Posture and Body Mechanics
- Stabilization Exercises
- Aerobic Exercises
- Stretching

<u>Stage III</u>

Participants with Stage III LBP are able to perform instrumental activities of daily life (IADLs) and are able to withstand moderate degrees of stress placed on the low back.

Oswestry scores of participants classified as Stage III are usually 20% or less at baseline assessment. Stage III generally indicates that the participant is de-conditioned due to relative inactivity during a LBP episode.

Interventions for Stage III Participants

- Regain Function Lost Due to Inactivity
- Develop Strength and Endurance in Regard to Occupational Tasks (Work Hardening)
- Return to Physical Activity (e.g., Sports)

© Boston Medical Center

Directional Preference (Stage I)

Participants in this subgroup exhibit the *centralization phenomenon*, which occurs when a clinically directed movement in a specific direction, such as lumbar flexion or extension, causes the participant's pain to rapidly decrease or move from a more peripheral location (e.g., buttocks) to a more central location (midline of the lumbar spine). Conversely, movement in the opposite direction may cause an increase in pain intensity or movement of pain to a more peripheral location.

For example, the more common directional preference extension pattern centralizes with extension and those participants are treated with repeated end-range extension exercises. Participants with the directional preference flexion pattern, for example, are prescribed end-range flexion exercises.

Participants who demonstrate extension DP may complain of pain in the back and worsening of pain when bending forward. They may report alleviation of pain when sleeping face down on the bed with a pillow under their head and trunk.

Participants who demonstrate a flexion DP are generally older. Older adults usually have some degree of spinal degeneration and disc narrowing which is thought to contribute to narrowing of the spinal canal, leading to symptoms of flexion DP. These participants may also report an inability to stand for too long, discomfort or increase in pain during walking, and immediate relief of pain upon sitting down.

Participants classified in the directional preference group demonstrate centralization of their pain (i.e., a rapid decrease or migration from a more peripheral location to a more central location) with at least two movements during examination (i.e., extension or flexion), or centralization with one movement and peripheralization (i.e., increased pain or migration of pain to a more peripheral location) with the opposite movement. The physical therapist prescribes an exercise program that is matched to the participant's subgroup classification.

Participants classified into the DP exercise-extension subgroup are prescribed extension-oriented exercises such as extension in prone (AKA press-ups) and extension in standing (AKA backward bending).

Participants classified into the DP flexion subgroup are prescribed flexion-oriented exercises such as supine knees to chest and seated flexion. Examples of exercises used for the specific exercise extension and flexion subgroups are in the home practice program section (page 51).

Stabilization (Stage II)

LBP in this subgroup is believed to be from spinal instability as a result of weak spinal muscles (transversus abdominus, erector spinae/multifidus, quadratus lumborum, oblique abdominals). Stabilization participants are most likely to benefit from lumbar muscle strengthening and stabilization exercises.

Participants who demonstrate no centralization and meet at least three or the following four criteria are classified into the stabilization group:

1. Age under 40 years old
2. Average straight leg raise range of motion >91°
3. Positive aberrant movements
4. Positive prone stability test

Stabilization participants are given lumbar spinal stabilization exercises using the protocol described by Hicks et al. These exercises target the four major muscle groups described above (transversus abdominus, erector spinae/multifidus, quadratus lumborum, oblique abdominals). Descriptions and photos of the stabilization exercises can be found in the Home Exercise Practice section (page 56).

If a participant does not meet the criteria above for specific exercise or stabilization, the participant is classified to the best fit according to the factors enumerated in the above algorithm. All PT participants will perform their prescribed exercises during PT sessions and will be asked to practice them at home, to the best of their ability, on the days they do not attend therapy.

© Boston Medical Center

Fear-Avoidance Belief Questionnaire (FABQ)

Although all study participants will complete the FABQ at enrollment of the study, it will only influence treatment for individuals assigned to the PT group (see Appendix A for instrument). Most recent data suggest that participants with scores greater or equal to 29 on the Work Subscale (FABQ-W) should be classified as having high fear avoidance beliefs.

Bio-behavioral interventions aim to enable participants to engage in regular activities to strengthen their back muscles instead of avoiding physical activity due to fear of pain or injury. Some of the key concepts supported by the bio-behavioral model are described in the box below. Examples of simple messages that can be conveyed to the participant by the physical therapist are also given.

The Fear-Avoidance Model of Low Back Pain		
**Concept**	**Example Message**	***The Back Book*** **Reference**
No sign of serious disease or suggestion of permanent damage for participants with non-specific low back pain.	"Degenerative disc disease is like gray hair. It is a normal part of aging."	Page 3
The spine is strong and spine pain does not necessarily mean your back has any serious damage.	"Your back is strong. Back pain does not mean you are hurting your back or causing any permanent damage."	Page 3
A number of treatments can help to control the pain, but lasting relief depends on your effort.	"Lasting relief of your back pain depends on your effort."	Pages 4-5
Concentrate on activity to restore normal function and fitness.	"Our goal together is to help you function better and have better 'back health.'"	Page 7
Encourage positive attitudes and coping.		Pages 14-18

Adapted from: George SZ, Bialosky JE, Fritz JM. Physical therapist management of patient with acute low back pain and elevated fear-avoidance beliefs. Phys Ther 2004;84:538-49.

© Boston Medical Center

The research staff administers the FABQ at the following intervals of the study:

- Baseline (Week 0)
- Week 6
- Week 12
- Week 26
- Week 40
- Week 52

FABQ-W scores are calculated by the physical therapist at the beginning of the in-person session OR the Back to Health research staff and reported to the physical therapists before the participant's next PT session.

Participants with High FABQ-W Scores (≥ 29)

Although many participants will exhibit at least some degree of reluctance to engage in activities that they associate with triggering or worsening back pain, some participants are at greater risk of impeding their treatment as a result of their fear-avoidance beliefs.

- FABQ-W status (primary component of interest) is recorded as *positive risk* as indicated by scores greater than or equal to 29.

Because high FABQ scores are predictors for impeded treatment effectiveness, participants who exhibit high scores receive coaching from the physical therapist using principles that counter fear avoidance beliefs (see page 36), including:

1. *The Back Book* addresses fears surrounding activity exacerbating back pain and to dispel myths about recovering from back pain. Specific cognitive principles are emphasized to help restructure the participant's perceptions about the relationship between physical movement and their back pain. The Back Book aims to accomplish the following:

   - Reduce fears regarding activity
   - Expand the understanding that some pain and discomfort is expected during recovery and that harm is not necessarily being caused by pain experienced by the participant
   - Encourage the participant to take an active role in their recovery
   - Regain participant confidence in returning to work and physical activity

   The therapist should regularly encourage participants with high FABQ-W subscale scores to review *The Back Book.*

2. *Coaching by the Physical Therapist*

Physical therapists will reinforce the concepts encompassed in *The Back Book* by offering encouragement and coaching to provide reassurance about the back, its ability to withstand exercise in order to promote recovery, and encouragement towards activation.

3. *Graded Exercise Program during the Physical Therapist-Guided Sessions*

Regardless of FABQ-W subscale score, all participants will be prescribed an aerobic exercise program during PT sessions. Exercise can take place on a treadmill, stationary bicycle, other aerobic apparatus, or by walking outside, depending upon participant preference and available equipment at health center sites. Each health center will, at minimum, have a stationary bicycle available. All participants will be informed of the goals for aerobic exercise in terms of frequency and intensity, where ultimate goals are:

- Frequency
    - All days participants are not attending physical therapist-guided sessions
- Intensity
    - 60% target heart rate for 30 minutes

However, for participants with high FABQ-W scores, the aerobic exercise program will be graded to achieve targeted heart rate levels by gradually increasing the frequency, duration, and intensity of exercise to promote confidence and success.

The physical therapist will set targets for the first week of aerobic exercise at 10% above resting heart rate. The physical therapist will monitor the heart rate and duration of the sustained heart rate and record the information on the follow-up flow chart. If the target heart rate and duration time requirements are met during one session, the physical therapist provides positive reinforcement and increases the heart rate quota by 10% for the aerobic component of the following session. If the target heart rate and duration requirement are not met, the physical therapist reemphasizes the importance of meeting the quota (accompanied by cognitive behavioral education) and the participant continues to try to meet the previously defined goal. See below for a detailed algorithm for the graded exercise program.

© Boston Medical Center

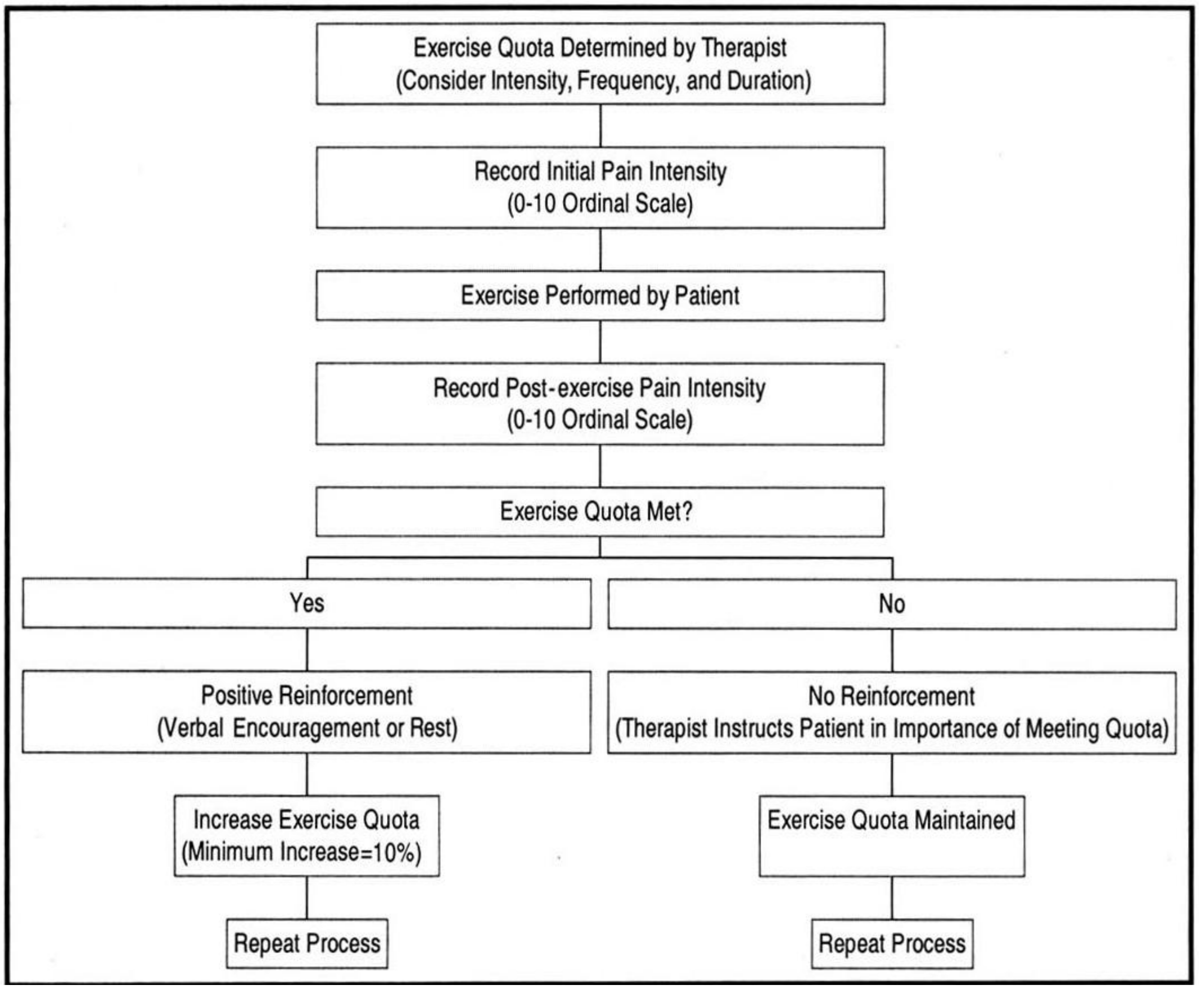

Adapted from: George SZ, Bialosky JE, Fritz JM. Physical therapist management of a patient with acute low back pain and elevated fear-avoidance beliefs. Phys Ther 2004;84:538-49.

Notably, participants who exhibit high FABQ-W subscale scores typically do not exercise at the intensity level necessary to achieve the targeted heart rate or do not maintain the duration of the exercise for 30 minutes. Intensity level and duration of aerobic exercise should be progressively incremented from the heart rates achieved by the participants during their last PT session until the targeted levels are achieved.

Participants with Low FABQ-W Scores

In lieu of the above, participants with low levels of fear avoidance behaviors (i.e., FABQ-W scores <29) will receive reinforcement by the PT to stay active, maintain positive coping strategies, and avoid passive modalities. Participants with low FABQ-W subscale scores will also receive a prescription for an exercise program but it will not be graded. This approach for participants with low FABQ scores has been used successfully in trials incorporating the FABQ classification.

Modified Oswestry Questionnaire

The Modified Oswestry Questionnaire (MOQ) assesses the level of disability of participants and will be used by the physical therapists as a measure of participants' progression at various stages of the PT treatment. The MOQ is administered at the following intervals of the study:

- Baseline (Week 0)
- Weeks1-12
- Month 4 (Maintenance Group)
- Month 6 (Maintenance Group)
- Month 8 (Maintenance Group)
- Month 10 (Maintenance Group)
- Month 12 (Maintenance Group)

The Modified Oswestry Questionnaire produces a score out of 50 points (each question comprising 5 points) which can be doubled to convert to a percentage score. However, if the participants do not provide responses to all of the questions, then the percentage score may be calculated as follows:

$$\frac{\text{Summation of response points}}{\text{Total possible points}} = \frac{\text{Total response score}}{[\text{number of questions answered}] \times 5 \text{ points}}$$

Example:

Participant provides responses to 8 of the 10 questions and the summation of the participant's responses is 16, then the score would be calculated as follows:

$$\frac{16}{8 \text{ questions answered} \times 5 \text{ points per question}} = \frac{16}{40} = 0.40 = 40\%$$

Percentage scores are interpreted as follows:

- 0% to 20% - minimal disability
- 20% to 40% - moderate disability
- 40% to 60% - severe disability
- 60% to 80% - crippled
- 80% to 100% - bed bound (or exaggerating symptoms)

Further literature references about the Modified Oswestry can be found in Appendix E.

Initial Intake Form

An initial intake form has been developed to assist the therapist in gathering all the necessary historical and physical examination information to guide the classification process. See Appendix A for the initial intake form. Primary elements of the intake are described below:

Medical History

- Location of pain
    - Low back pain (LBP)
    - Low back and buttock/thigh symptoms
        - Pain does not extend past knee from low back or buttock/thigh
    - LBP and leg symptoms distal to knee
        - Leg pain is worse than back pain
- Sought medical care for the same episode of pain that occurred in the past
- Location of other symptoms
    - Head/neck
    - Thoracic Spine
    - Upper Extremity/Extremities
    - Hip(s)
    - Knee(s)
    - Foot/Feet
    - Patient denies other symptoms
- Previous episode(s) of LBP in the last year
    - Instances when medical treatment was sought
- Frequency of pain increasing
- Subjective pain rating: participants will be asked to rate his or her pain experience on an 11-point scale (a score of 0 representing not having any pain and a score of 10 representing the worst pain imaginable) during the following time periods:
    - Worst pain in the past 24 hours
    - Best pain level in the past 24 hours
    - Average pain level in the past 24 hours

Physical Examination
At a minimum, the physical exam should include the following (results of each physical component should be recorded; photographs can be found in Appendix B):

<u>Deep Tendon Reflex (DTR) Testing</u>
With the participant seated with his/her legs unsupported, test knee jerk and ankle jerk reflexes using a reflex hammer. Record the results, using the following grading system:

- 0 = absent
- 1 = diminished
- 2 = normal
- 3 = hyper-reflexive

<u>Motor Examination</u>

- Motor examination—the following tests indicate strength and each is rated on a 5-level scale:
    - Ankle Dorsiflexion: anterior tibialis to test the L4 nerve root
    - Great Toe Extension: Extensor halluces longus (EHL) to test the L5 root
    - Ankle Eversion: Peroneals to test the L5 and S1 roots
    - Ankle Plantar Flexion: Gastroc/soleus to test the S1 and S2 roots

**Levels of Muscle Performance during Testing**	
**Level of performance**	**Definition**
5	Completes full range of motion against gravity and holds test position against strong pressure.
4	Completes full range of motion against gravity and holds test position against moderate to strong pressure.
3	Completes full range of motion against gravity and holds test position with slight or no added pressure. There may a gradual release from test position.
2	Completes partial range of motion against gravity and moves through complete range of motion in horizontal plane.
1	Completes partial range of motion in horizontal plane. No visible movement of the part, but examiner may observe or palpate contractile activity in the muscle.
0	No contraction detected in the muscle.

Rothstein JM, Roy SH, Wolf SL. The Rehabilitation Specialist's Handbook. 2nd ed. Philadelphia: F.A. Davis Co. 1998.

Sensory examination

Sensation to light touch is assessed throughout the following distributions (where sensation is classified as *absent, diminished, or normal)*:

- Medial lower leg/foot to test L4 nerve root
- Lateral leg/foot to test the L5 nerve root
- Lateral side of foot to test the S1 nerve root

Aberrant movements (standing)

Record as 'positive' (i.e., "yes") if any of the following are observed during sagittal plane motion (forward flexion and return to upright):

- Instability catch—any trunk movement outside of the plane of specified motion (e.g., side bending during trunk flexion).
- Painful arc—symptoms felt during the movement at a particular point in the motion (or through a particular portion of the range) that are not present before or after this point. A painful arc may occur in flexion or return from flexion.
- Thigh climbing—using the hands on thighs (or some other external support) to push up on when returning from flexion to upright.
- Reversal of lumbopelvic rhythm—the trunk being extended first, followed by extension of the hips and pelvis to bring the body back to upright standing from a forward flexed position.

Directional preference testing (standing)

Single movements performed in standing are not always adequate to elicit centralization. When this occurs, performing trunk flexion or extension in one direction or holding sustained positions at end range may be helpful for producing centralization. If centralization can be produced with repeated or sustained movements, the participant will be treated using those movements (i.e., flexion or extension exercises).

- See the specific exercises for extension DP group listed in the Home Exercise Program section (page 51).

Lumbar flexion range of motion (ROM) (standing)

Using a bubble inclinometer (calibrated to zero degrees prior to testing) placed at the thoraco-lumbar junction and keeping their knees fully extended, participants are asked to flex forward in an attempt to reach their hands to the floor. The measurement is recorded in degrees.

- Make note of the behavior of the symptoms before, during, and following the movement in question. In order to effectively identify centralization or peripheralization, the participant's baseline status prior to movement testing must be determined. Therefore, the participant must be questioned regarding the location and nature of symptoms just prior to movement. The participant then performs the test movement and explains any change in symptoms that may occur as a result.
- It is important to remember that temporary fluctuations in pain intensity with movement do not constitute centralization or peripheralization.

<u>Average straight leg raise (SLR) (supine)</u>

Holding a bubble inclinometer (calibrated to zero degrees prior to testing) placed along the lateral malleolus, the PT will passively lift the participant's leg with one arm while using the other arm to maintain the same knee in extension. The leg is raised slowly to the maximum tolerated SLR, watching for hip flexor tightness on the other side and ensuring the resting leg does not lift off the surface. The opposite leg is tested in the same manner.

Maximum SLR is recorded for the right and left legs in degrees and the measurements from each of the leg sides are averaged.

<u>Spinal segment mobility (spring) testing (prone)</u>

Testing is performed by placing the hypothenar eminence of the hand over the spinous process of the segment to be tested. Keeping the elbow and wrist extended, the examiner applies a firm anteriorly-directed pressure on the spinous process to isolate the painful segment(s). Painful segments are retested with the participant's legs lifted. The following are the three possible findings for each level tested:

1. Hypomobility—passive mobility is judged to be hypomobile in at least 1 segmental level
2. Normal—passive mobility is judged to be normal throughout the lumbar spine (L1-L5)
3. Hypermobility—passive mobility is judged to be hypermobile in at least 1 segmental level

Both 'hypo' and 'hyper' may be indicated if at least one segment that is hypomobile and at least one segment that is hypermobile are found. If all segments exhibit normal mobility, then record the participant's mobility as 'normal.'

Prone instability test (prone)

The participant lies prone with the body on the examining table, legs over the edge and feet resting on the floor. While the participant rests in this position, the examiner applies posterior to anterior pressure to a lumbar spinal segment. This is repeated along all lumbar spinal segments and the participant reports any provocation of pain. Then the participant lifts the legs off the floor (the participant may hold the table to maintain position) and compression is applied again to the lumbar segments indicated as painful by the participant during the previous test.

- If pain is present in the initial resting position but subsides substantially in the second position (legs off floor), the test is *positive*. If pain is present in the resting position but does not subside substantially in the second position, the test is *negative*.

© Boston Medical Center

Weekly Intake and Treatment Visits

Summary of Intake and Treatment Visits	
15-20 minutes	Participant Checks In/Completes Surveys (periodic visits only)
5-10 minutes	Intake/Reclassification
20 minutes	Complete Therapist-Guided Stabilization Exercises
15-30 minutes	Aerobic Exercise

During the first 5-10 minutes of the session, participants should be reclassified. Physical therapists re-evaluate participants once per week in order to ensure they are categorized within the correct treatment group for the home exercise program.

The weekly intake and physical exam consists of the following:

- Subjective pain rating
- Aberrant movements
- Directional preference testing
- Lumbar flexion range of motion
- Average SLR
- Spinal segment mobility testing
- Prone instability test

The physical therapist provides additional guidance or review of PT exercises if the participant displays noticeable regression.

Regardless of the classification of the participant during the intake, the next 20 minutes should be used to complete stabilization exercises guided by the physical therapist. In rare cases, if radicular pain is present, the participant would perform directional preference exercises in accordance with his/her classification at intake.

Stabilization Exercise Regimen

Participants should begin with the first exercise in each muscle category, working towards the progression criteria (adding time and repetitions according to the chart) before moving on to the next exercise in the following muscle category. If a participant is unable to complete all the recommended repetitions, then the participant should end exercises for that muscle group and begin exercises for the next muscle group. Participants continue to perform exercises to his or her individual ability until all

repetitions of the stabilization exercises are achieved. Starting positions should be adjusted for those participants in the DP group in order to keep radicular symptoms under control. Participants should stop the exercises if their radicular symptoms worsen or they are unable to continue.

Stabilization Exercises		
**Primary Muscle Group**	**Exercises**	**Criteria for Progression**
**Transversus abdominus**	Abdominal Bracing	30 repetitions w/ 8s hold
	Bracing with heel slides	20 repetitions per leg w/ 4s hold
	Bracing with leg lifts	20 repetitions per leg w/ 4s hold
	Bracing with bridging	30 repetitions w/ 8s hold, progress to 1 leg
	Bracing in standing	30 repetitions w/ 8s hold
	Bracing with standing row exercise	20 repetitions w/ 6s hold
	Bracing with walking	
**Erector spinae/ multifidus**	Quadraped arm lifts w/ bracing	30 repetitions w/ 8s hold on each side
	Quadraped leg lifts w/ bracing	30 repetitions w/ 8s hold on each side
	Quadraped alt arm & leg lifts w/ bracing	30 repetitions w/ 8s hold on each side
**Quadratus lumborum**	Side support w/ knees flexed	30 repetitions w/ 8s hold on each side
	Side support w/ knees extended	30 repetitions w/ 8s hold on each side
**Oblique abdominals**	Side support w/ knees flexed	30 repetitions w/ 8s hold on each side
	Side support w/ knees extended	30 repetitions w/ 8s hold on each side

Participants begin with the first exercise in each muscle category, working towards the progression criteria (adding time and repetitions according to the chart) before moving on to the next exercise in the following muscle category. If a participant is unable to complete all the recommended repetitions, then the participant should end exercises for that muscle group and begin exercises for the next muscle group. If the participant is able to complete the exercises within a muscle group category, then the participant should progress to the next exercise within that muscle group exercise category. Participants will continue to perform exercises to his or her individual ability until all repetitions of exercises within all muscle groups have been achieved.

Next, an aerobic component will performed during the PT treatment session. Although the order of the stabilization exercises and the aerobic exercises is not imperative, the sessions are standardized (first stabilization exercises, then aerobic exercises) for the design of the study. Furthermore, performing stabilization with the participant at the beginning of the session and having the participant complete the aerobic component of

the session last will allow the physical therapist to begin stabilization exercises with the next participant.

During the last 15-30 minutes of the session, participants engage in aerobic exercise. For aerobic exercise, participants choose between walking/jogging on the treadmill and riding a stationary bicycle (keeping in mind directional preference if present). The aim of the aerobic exercise is for participants to maintain their heart rate at 65-75% of the maximum estimated heart rate as determined by their sex and age. Heart rate is measured by the PT using a battery-operated heart rate monitor. Intensity may be adjusted to achieve the target heart rate, where maximum HR is estimated by the following equations:

<u>Men</u>
Maximum heart rate = 220 beats per minute – (age)

<u>Women</u>
Maximum heart rate = 200 beats per minute – (age)

Participants with high FABQ-W Subscale Scores (≥29)

Participants who demonstrate high Fear-Avoidance Beliefs (FABQ-W score ≥29) will receive additional encouragement and coaching during the stabilization exercises to remind the participant that not all back pain is detrimental to recovery and that physical activity is beneficial for low back pain. These participants will be encouraged to complete the most rigorous exercise level in which they are comfortable and are able to engage, as deemed appropriate by the physical therapist.

Participants with high FABQ scores from the most recent survey will be gently encouraged with positive reinforcement to engage in aerobic exercise by the physical therapist (see cognitive behavioral education chart on page 36).

Follow-Up Flow Sheet

The follow-up flow sheet is a tool to record participant treatment and is divided into the following study time periods by page:

- Initial Intake (week 1) and Twice Per Week Sessions (weeks 2-4)
- Once Per Week Sessions (weeks 5-8 & 9-12)
- Maintenance Sessions (months 4, 6, 8, 10, &12)

A sample can be found in Appendix A.

Other components included on this sheet are the following:

Patient Estimated Maximum Heart Rate: Reference estimated maximum heart rate based on sex and age, using the following equations:

<u>Men</u>
Maximum heart rate = 220 beats per minute – (age)

<u>Women</u>
Maximum heart rate = 200 beats per minute – (age)

Date: date of the PT session

Patient Late for Appointment: check if the participant is late to the appointment

Classification: Classification for the participant each week during intake (stabilization, extension directional preference, or flexion directional preference)

Modified Oswestry Disability score: Score from the most recent Modified Oswestry Questionnaire taken by the participant. The participant will either provide the completed questionnaire and the score may be calculated or it will be provided by the Back to Health research study staff before the session.

FABQ-W subscale score: Work subscale score from the Fear-Avoidance Belief Questionnaire. The participant will either provide the completed questionnaire and the score may be calculated or the score will be provided by the Back to Health research study staff before the session.

Stabilization Exercises: The number of repetitions for each exercise and the duration of holding can be recorded. Criteria for progression to the next exercise are also listed.

Stationary Bike: Aerobic exercise in the form of stationary bicycle time (in minutes)

Patient's Age: Age of the patient, used to calculate the target heart rate

Target Heart Rate: Based on participant's FABQ-W subscale score, age and sex. For more details, please refer to procedures for participants with high FABQ-W in the "Intake and Treatment Visits" section (page 46).

Percent Target Heart Rate: Based on participant's FABQ-W subscale score, age and sex. For more details, please refer to procedures for participants with high FABQ-W in the "Intake and Treatment Visits" section (page 46).

Time at Target Heart Rate: Time (in minutes) the participants spend at the target heart rate

© Boston Medical Center

Home Exercise Program

Participants are classified weekly during intake. Based on their individual classification, participants are given home exercise programs (HEP) to complete on non-treatment days and record their progression using the HEP log which participants submit on a weekly basis. The physical therapist reviews the HEP with the participants at each visit.

Directional Preference Classification Groups

When working with participants classified into the directional preference subgroups (i.e., flexion or extension), the goal is to centralize the participant's leg symptoms, and ultimately be able to re-classify the participant into the stabilization group.

For the participant with an extension DP, the following exercises should be given as a home exercise program. Participants are asked to work towards the suggested hold times and repetitions, keeping in mind that they should avoid any exercises that worsen symptoms (i.e., cause peripheralization).

Specific Exercises for Extension Directional Preference Group*

1. Prone lying. Lie on your stomach with arms along your sides and head turned to one side. Maintain this position for 5 to 10 minutes.

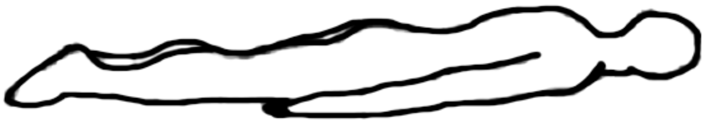

2. Prone lying on elbows. Lie on your stomach with your weight on your elbows and forearms and with your hips touching the table, floor or mat. Relax your lower back. Remain in this position 5 to 10 minutes. If this causes pain, repeat exercise 1, then try again.

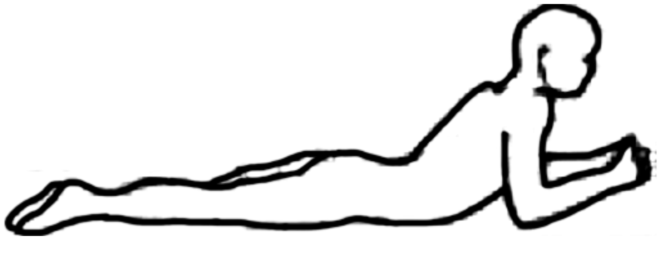

*Adapted from: www.backtrainer.com/Williams-Flexion-Versus-McKensie-Extension-Exercises-For-Low-Back-Pain.html

© Boston Medical Center

3. Prone press-ups. Lie on your stomach with palms near your shoulders, as if to do a standard push-up. Slowly push your shoulders up, keeping your hips on the surface and letting your back and stomach sag. Slowly lower your shoulders. Repeat 10 times.

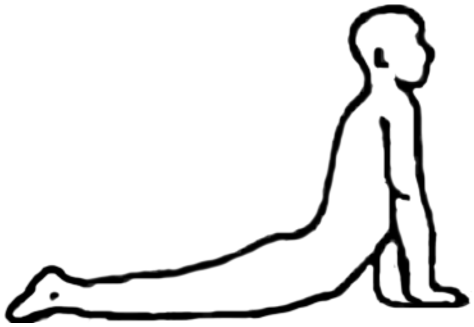

4. Progressive extension with pillows. Lie on your stomach and place a pillow under your chest. After several minutes, add a second pillow. If this does not hurt, add a third pillow after a few more minutes. Stay in this position up to 10 minutes. Remove pillows one at a time over several minutes.

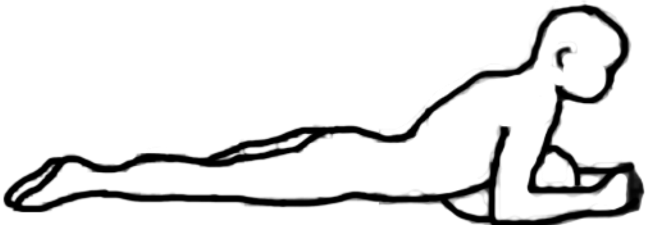

5. Standing extension. While standing, place your hands in the small of your back and lean backward. Hold for 20 seconds and return to neutral standing. Repeat 10 times. Use this exercise after normal activities during the day that place your back in a flexed position: lifting, forward bending, sitting, etc.

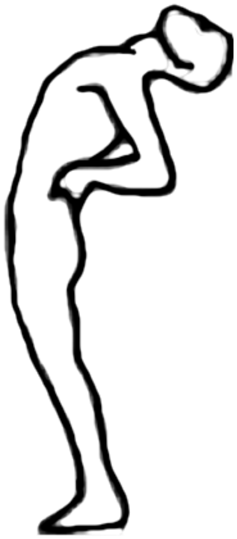

Specific Exercises for Flexion Directional Preference Group†

1. Pelvic tilt. Lie on your back with knees bent, feet flat on floor. Flatten the small of your back against the floor, without pushing down with the legs. Hold for 5 to 10 seconds. Repeat 10 times.

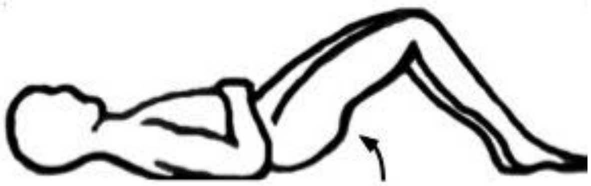

2. Single knee to chest. Lie on your back with knees bent and feet flat on the floor. Slowly pull your right knee toward your shoulder and hold 5 to 10 seconds. Lower the knee and repeat with the other knee. Repeat 10 times on each side.

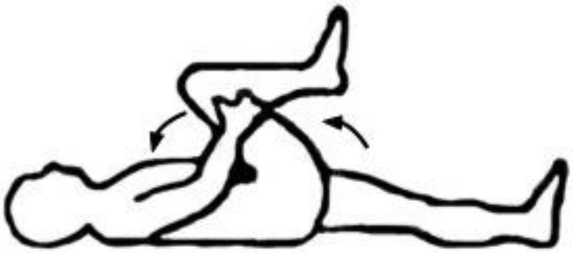

3. Double knee to chest. Begin as in the previous exercise. After pulling right knee to chest, pull left knee to chest and hold both knees for 5 to 10 seconds. Slowly lower one leg at a time. Repeat 10 times.

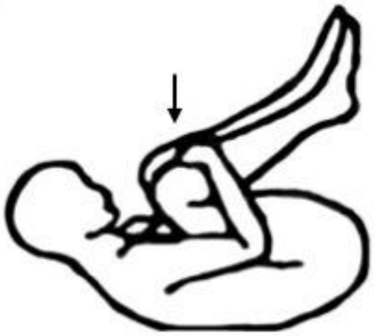

†Adapted from: www.backtrainer.com/Williams-Flexion-Versus-McKensie-Extension-Exercises-For-Low-Back-Pain.html

4. Partial sit-up. Do the pelvic tilt (exercise 1) and, while holding this position, slowly curl your head and shoulders off the floor. Hold briefly. Return slowly to the starting position. Repeat 10 times.

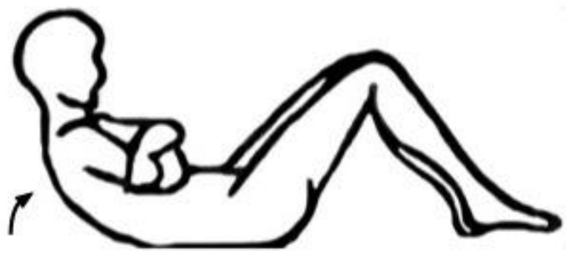

5. Hamstring stretch. Start in long sitting, legs outstretched with toes directed toward the ceiling and knees fully extended. Slowly lower the trunk forward over the legs, keeping knees extended, arms outstretched over the legs, and eyes focus ahead. Hold for 5 to 10 seconds. Return to starting position. Repeat 10 times.

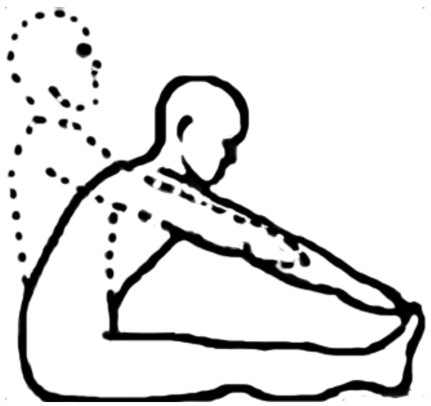

6. Hip flexor stretch. Place left foot in front of the right with the left knee flexed and the right knee held rigidly straight. Flex forward through the trunk until the left knee contacts the axillary fold (armpit region). Hold for 5 to 10 seconds. Repeat with right leg forward and left leg back. Repeat 10 times on each side.

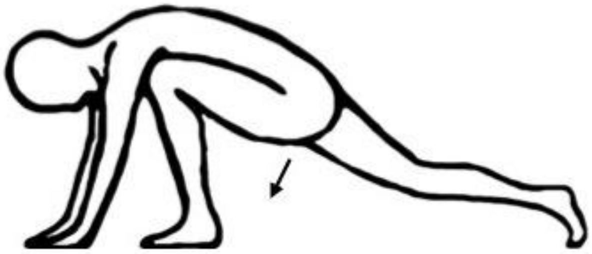

7. Squat. Stand with both feet parallel, about shoulders' width apart. Attempting to maintain the trunk as perpendicular as possible to the floor, keeping eyes focused ahead and feet flat on the floor, the participant slowly lowers his body by flexing his knees.

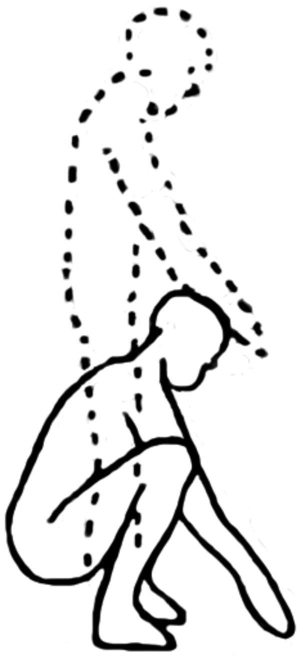

Stabilization Classification Group

For participants classified into the stabilization classification group, exercises should be taught and assigned according to the Home Exercise Program handout. Participants should begin with the first exercise in each muscle category, working towards the progression criteria (adding time and repetitions according to the chart) before moving on to the next exercise in the following muscle category.

Participants with high FABQ-W subscale scores will receive additional coaching (see page 36 for cognitive behavioral education chart) and monitoring by the therapist.

If the participant is able to complete the exercises within a muscle group category, then the participant should progress to the next exercise within that muscle group exercise category. If a participant is unable to complete all the recommended repetitions, then the participant should end exercises for that muscle group and begin exercises for the next muscle group. Participants continue to perform exercises to his or her individual ability until all repetitions of exercises within all muscle groups of the stabilization exercises are able to be achieved. Participants should stop the exercises if their radicular symptoms worsen or they are unable to continue.

Abdominal Bracing Exercises (Non-Weight Bearing)

1. Abdominal bracing (supine)

Lie on your back with your knees bent. Tighten your stomach muscles **without** pressing your back flat to the floor. Hold for 8 seconds, counting aloud to avoid holding your breath. Relax and repeat.

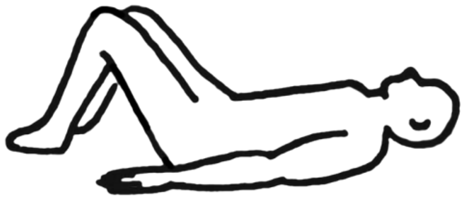

Repetitions: ______________/30

2. Abdominal bracing (supine) with heel slide

Lie on your back with knees bent as above. While tightening your stomach muscles (abdominal brace), slide the heel of one foot away from you until your knee is straight (3 second count).

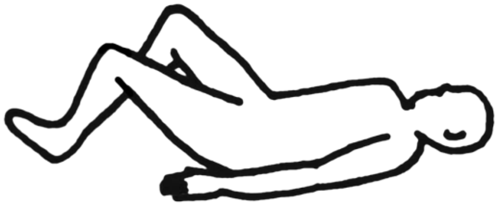

Then, slide your heel back towards you until your knee is in its original bent position (3 second count). Relax and repeat on opposite leg.

Repetitions: ______________/20

© Boston Medical Center

3. Abdominal bracing (supine) with leg lifts

Lie on your back with knees bent. While tightening your stomach muscles (abdominal brace), lift one foot about 6 inches off the floor for a 3 second count. Then, return it to the floor at a 3 second count. Relax and repeat with opposite leg.

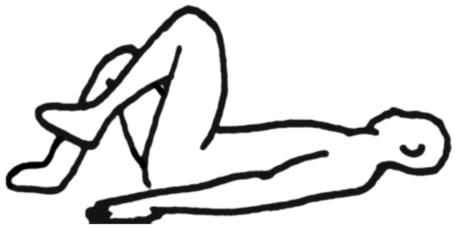

Repetitions: _____________/20

4. Abdominal bracing (supine) with bridging

Lie on your back with knees bent. While tightening your stomach muscles (abdominal brace), tighten your buttocks and slowly lift them off the floor. Do not allow your back to arch. Hold for 8 seconds. Relax and repeat.

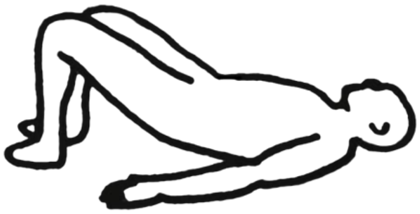

Repetitions: _____________/30

5. Bracing with single leg bridging

Lie on your back with knees bent. While tightening your stomach muscles (abdominal brace), tighten your buttocks and slowly straighten one knee so that only one foot is on the floor. Then, slowly lift your buttocks off the floor. Hold for 8 seconds. Relax and repeat.

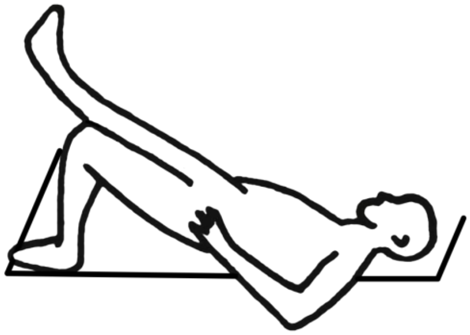

Repetitions: _____________/30

Abdominal Bracing Exercises (Weight Bearing)

1. Abdominal bracing in standing

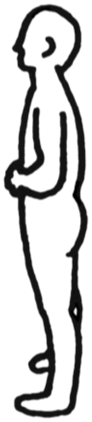

While standing, tighten your stomach muscles without changing the curve in the small of your back. Hold for 8 seconds. Relax and repeat.

Repetitions: _____________/30

2. Isometric Torsion

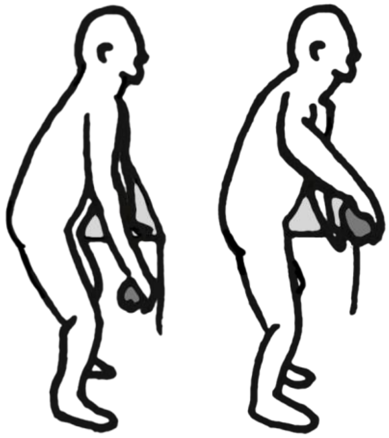

Stand in a slightly forward-bent position with one hand used as support on a table. Perform the **abdominal brace** and lift a light weight (2-3 lb. dumbbell, can of soup, etc.) to the final position seen in the pictures to the right.

Repetitions: _____________/20

3. Abdominal bracing with walking

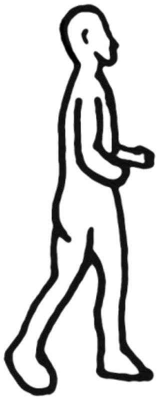

While walking, perform the abdominal brace. Hold 8 seconds. Relax for 10 seconds and repeat. Continue this cycle as you walk. Progress up to 10 minutes of walking.

© Boston Medical Center

Side Support Exercises

1. Side support with knees flexed

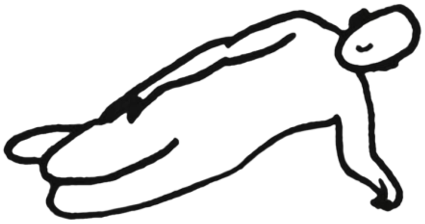

Lie on your side with knees bent and upper body supported on the lower elbow. Then, lift your body from the table with all weight borne on the lower knee and elbow. Hold for 8 seconds. Relax and repeat.

Repetitions: ______________/30

2. Side support with knees flexed and bracing

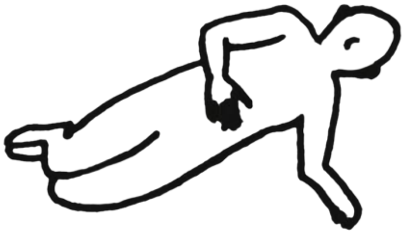

Perform the abdominal brace (tighten your stomach muscles) then do the side support as above. Hold for 8 seconds. Relax and repeat.

Repetitions: ______________/30

3. Side support with knees extended

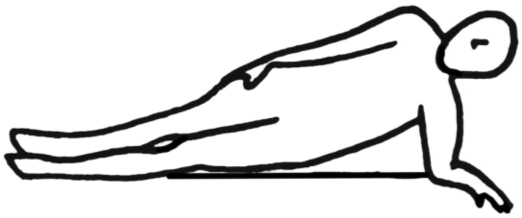

Lie on your side with knees straight and upper body supported on the lower elbow. Then, lift your body from the table with all weight borne on the lower foot and elbow. Hold for 8 seconds. Relax and repeat.

Repetitions: ______________/30

4. Side support with knees extended and bracing

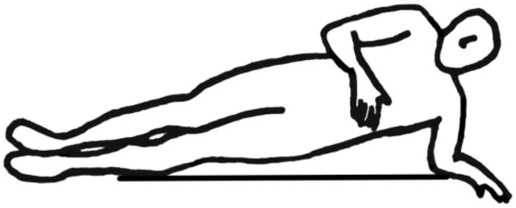

Perform the abdominal brace (tighten your stomach muscles) then do the side support as above with knees straight. Hold for 8 seconds. Relax and repeat.

Repetitions: ______________/30

5. Advanced Side Bridge

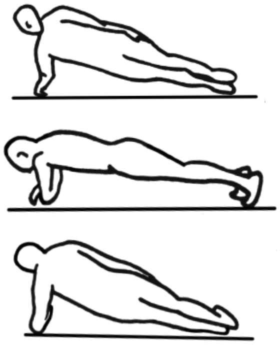

Perform the side support as described above. Roll from one elbow to the other while abdominally bracing to attain a side support position on the opposite elbow. Then, lower yourself from side support position on the opposite elbow down to the floor.

Repetitions: ______________/30

Quadruped Exercises

Do not begin exercise in this group until able to complete Abdominal Bracing in supine (10 repetitions x 8 second holds)

1. Quadruped Arm Lifts with bracing

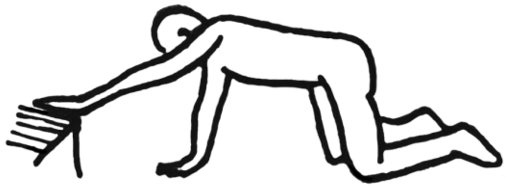

Start on your hands and knees. Tighten your stomach muscles. Then lift your right arm from the table. Hold for 8 seconds. Return to start position and repeat with left arm.

Repetitions: _____________/30

2. Quadruped Leg Lifts with bracing

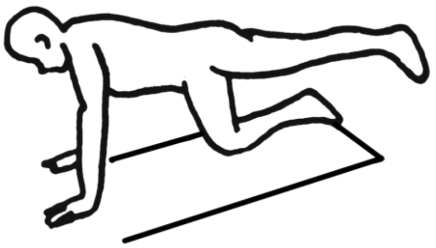

Start on your hands and knees. Tighten your stomach muscles. Then extend your right leg so that your knee is lifted from the table. Keep your hips level with the table as you do this. Hold for 8 seconds. Return to start position and repeat with left leg.

Repetitions: _____________/30

3. Quadruped Opposite Arm and Leg Lift with bracing

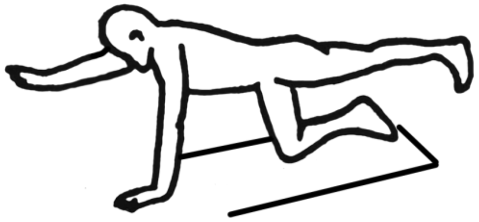

Start on your hands and knees. Tighten your stomach muscles. Then extend your right leg so that your knee is lifted from the table and lift your left arm from the table at the same time. Keep your hips level with the table as you do this. Hold for 8 seconds. Return to start position and repeat with left leg.

Repetitions: _____________/30

© Boston Medical Center

Assignment and Treatment: Maintenance Group and Home Practice Group

Within days of the final PT visit and 12-week data collection, PT participants learn whether they have been randomly assigned to the PT Maintenance Group or Home Practice Group. The PT Maintenance Group will meet with the physical therapist for five additional sessions over the remaining 40 weeks of the study. These sessions occur on or around weeks 18, 26, 32, 40, and 46 of the study. The participants assigned to the Home Practice group complete their home practice program as prescribed by the PT at the 12-week visit, and receive no additional sessions.

Participants in both groups will complete and submit home practice logs.

Maintenance Group

Participants assigned to the PT Maintenance Group will continue to attend five additional PT sessions over the following 40 weeks of the study at the following intervals: 18, 26, 32, 40 and 46 weeks.

Maintenance sessions are scheduled by the research staff. However, if a participant is late to an appointment, NEPT places a reminder call to the participant. All sessions missed that day are reported to the research staff who will reschedule the missed session.

Each session during the Maintenance Phase will be conducted similar to previous sessions in the Treatment Phase, as follows:

**Maintenance Phase Treatment Visits**	
5-10 minutes	Intake/Reclassification
20 minutes	Complete Stabilization Exercises
15-30 minutes	Aerobic Exercise

Participants with high FABQ scores from the most recent survey will be gently encouraged with positive reinforcement to engage in aerobic exercise by the physical therapist (page 36).

Home Practice Group

Participants assigned to the Home Maintenance Group will perform recommended exercises at home, in accordance with the home practice program assigned by the physical therapist at the 12-week visit before the random assignment to either the Maintenance Group or Home Practice Group.

© Boston Medical Center

APPENDIX A: Forms and Questionnaires

Back to Health Patient Questionnaire	63
Physical Therapy First Intake Form	64
Physical Therapy Weekly Visit Intake Form	66
Follow-up Flow Chart Weeks 1-4	67
Follow-up Flow Chart Weeks 5-8	68
Follow-up Flow Chart Weeks 9-12	69
Follow-up Flow Chart Weeks Maintenance	70
Weekly Home Practice Log: Extension Exercises	71
Weekly Home Practice Log: Flexion Exercises	72
Weekly Home Practice Logs: Stabilization Exercises	73
12-Week Discharge Summary	77
FABQ Questionnaire	78
Modified Oswestry LBP Disability Questionnaire	81

Back to Health Patient Health Questionnaire

First Name: ___________________ Last Initial: ______ Age: ______ Date:_____/_____/_______

Handedness (Circle one): Right Left **Women**: Are you currently pregnant? (Circle one) Y / N

Within the past year, have you had any of the following symptoms (check all that apply):

☐ Chest Pain	☐ Loss of balance	☐ Bladder/bowel problems
☐ Heart palpitations	☐ Difficulty walking	☐ Unexplained weight loss/gain
☐ Persistent cough	☐ Joint pain or swelling	☐ Poor circulation
☐ Hoarseness	☐ Pain at night	☐ Fever/chills/sweats
☐ Shortness of breath	☐ Difficulty sleeping	☐ Headaches
☐ Dizziness or fainting	☐ Loss of appetite	☐ Hearing problems
☐ Coordination problems	☐ Nausea/vomiting	☐ Visions problems
☐ Weakness in the arms or legs	☐ Difficulty swallowing	☐ Other: _________________

Do you have allergies to latex or latex sensitivity? __________________________________

List any other allergies you have: ____

____

Medical History (check conditions that you currently have or have had in the past):*

☐ Arthritis	☐ Low blood sugar/hypoglycemia	☐ Tuberculosis
☐ Broken bones/fractures	☐ Head injury	☐ Hepatitis
☐ Osteoporosis	☐ Multiple sclerosis	☐ HIV Infection
☐ Circulation/vascular problems	☐ Muscular dystrophy	☐ Other infectious disease
☐ **Heart attack**	☐ Parkinson's disease	☐ Ulcers/stomach problems
☐ **High blood pressure**	☐ Seizure/epilepsy	☐ Skin diseases
☐ Lung problems	☐ Developmental/growth problems	☐ Depression
☐ Stroke	☐ Thyroid problems	☐ Kidney problems
☐ **Diabetes**	☐ Cancer (type: __________)	☐ Other: _________________

* Please notify your physical therapist of any of the **bolded conditions** you currently have or have had.

List all past surgeries (month/year): ____

____

Are you on beta-blocker medication (e.g. Metoprolol, Toprol, Atenolol)? ____________________

List all medications you are currently taking: ______________________________________

____

____

____

Physical Therapist Initials ______________ Date of Review _________________

B2H STAFF USE ONLY: Printed Name ________________ Signature _________________ Date ________

Back to Health
A Study Comparing Yoga, Physical Therapy & Education for Chronic Low Back Pain

PHYSICAL THERAPY FIRST VISIT INTAKE FORM

PT Initials: ________

DEMOGRAPHIC INFORMATION

Date:__/__/_____ Patient Name: ____________________ Gender: □ Male □ Female

MEDICAL HISTORY

1. Location of pain (*check one*):

   □ LBP

   □ LBP and buttock/thigh symptoms

   (pain does not extend past knee from low back or buttock/thigh)

   □ LBP and leg symptoms distal to knee (leg pain is worse than back pain)

2. Sought medical care for the same episode of pain that occurred in the past?

   □ Yes □ No

3. Location of other symptoms (*check all that apply*):

   □ Head/neck
   □ Thoracic Spine
   □ Upper Extremity/Extremities
   □ Hip(s)
   □ Knee(s)
   □ Foot/Feet
   □ Patient denies other symptoms

4. Previous episode(s) of LBP in the last year (i.e instances when medical treatment was sought):

   □ 0 □ 1-2 □ 3-5 □ > 5

5. Frequency of pain increasing?

   □ Yes □ No

6. Worst pain level in past 24 hours: ______/10

7. Best pain level in past 24 hours: ______/10

8. On average, pain level in the past 24 hours: ______/10

PHYSICAL EXAMINATION

DEEP TENDON REFLEXES

	Left	Right
Knee Jerk	____	____
Ankle Jerk	____	____

0=Absent 2=Normal
1=Diminished 3=Hyper Relative

MOTOR EXAMINATIONS

	Left	Right
Ant. Tibialis (L4)	__/5	__/5
EHL (L5)	__/5	__/5
Peroneals (L5-S1)	__/5	__/5
Gastroc/Soleus (S1-S2)	__/5	__/5

0-10 Heel Raises

SENSORY EXAMINATION

	Left			Right		
	Absent	Diminished	Normal	Absent	Diminished	Normal
L4 (Medial Lower Leg/Foot)	□	□	□	□	□	□
L5 (Lateral Leg/Foot)	□	□	□	□	□	□
S1 (Lateral Side of Foot)	□	□	□	□	□	□

Back to Health — A Study Comparing Yoga, Physical Therapy & Education for Chronic Low Back Pain

Patient Name: ______________________________ **PT Initials:** ___________

PHYSICAL EXAM (continued)

1. Aberrant Movements
   - □ Yes
   - □ No
2. Directional Preference
   - □ Extension
   - □ Flexion
   - □ No Directional Preference
3. Lumbar Flexion ROM: __________
4. Average SLR
   - □ ≥91
   - □ <91

   RIGHT: _____ _____ LEFT: _____ _____
5. Spinal Segment Mobility Testing
   - □ Hypo
   - □ Normal
   - □ Hyper
6. Prone Instability Test
   - □ Positive
   - □ Negative

TREATMENT CLASSIFICATION

7. Stage I (*check one*):
   - □ Stabilization
   - □ Flexion Directional Pref
   - □ Extension Directional Pref
8. Stage II (*check all that apply*):
   - □ Aerobic
   - □ General Conditioning (Stabilization Exercises)
9. Oswestry Score: __________
10. FABQW Score: __________
11. FABQW Status (*check one*):
    - □ Negative Risk (< 29)
    - □ Positive Risk (≥ 29)

    (for Positive Risk, also select "Patient Education/Instruction" and "Behavioral Therapy" in the Intervention Section)

NOTE: You must check (1) One Stage I category and (if applicable) one or more Stage II categories and (2) One FABQ status

INTERVENTIONS

12. Interventions (check all that apply):
    - □ Patient Education/Instruction
    - □ Behavioral Therapy

Check one of three:
- □ Extension Protocol
- □ Flexion Protocol
- □ Stabilization Protocol

Check all that apply:
- □ Functional Training
- □ General Conditioning (Stabilization Exercises)

Reviewed Medical History: □ Unremarkable □ Other: ______________________________

NOTES

____
____
____
____
____
____
____
____
____
____

Back to Health — A Study Comparing Yoga, Physical Therapy & Education for Chronic Low Back Pain

PHYSICAL THERAPY WEEKLY VISIT INTAKE FORM

PT Initials: ________

DEMOGRAPHIC INFORMATION

Date:___/___/_____ Patient Name: ______________________ Gender: □ Male □ Female

PHYSICAL EXAMINATION

1. Aberrant Movements
   - □ Yes
   - □ No

2. Directional Preference
   - □ Extension
   - □ Flexion
   - □ No Directional Preference

3. Lumbar Flexion ROM: __________

4. Average SLR
   - □ ≥91
   - □ <91

   RIGHT: _____ _____ LEFT: _____ _____

5. Spinal Segment Mobility Testing
   - □ Hypo
   - □ Normal
   - □ Hyper

6. Prone Instability Test
   - □ Positive
   - □ Negative

7. Worst pain level in past 24 hours: _____/10

8. Best pain level in past 24 hours: _____/10

9. On average, pain level in the past 24 hours: _____/10

TREATMENT CLASSIFICATION

12. Stage I (*check one*):
    - □ Stabilization
    - □ Flexion Directional Pref
    - □ Extension Directional Pref

13. Stage II (*check all that apply*):
    - □ Aerobic
    - □ General Conditioning (Stabilization Exercises)

NOTE: You must check (1) One Stage I category and (if applicable) one or more Stage II categories and (2) One FABQ status

14. Oswestry Score: __________

15. FABQW Score: __________

16. FABQW Status (*check one*):
    - □ Negative Risk (< 29)
    - □ Positive Risk (≥ 29)

    (for Positive Risk, also select "Patient Education/Instruction" and "Behavioral Therapy" in the Intervention Section)

INTERVENTIONS

16. Interventions (check all that apply):
    - □ Patient Education/Instruction
    - □ Behavioral Therapy

Check one of three:
- □ Extension Protocol
- □ Flexion Protocol
- □ Stabilization Protocol

Check all that apply:
- □ Functional Training
- □ General Conditioning (Stabilization Exercises)

NOTES

____

____

____

____

____

____

Back to Health — A Study Comparing Yoga, Physical Therapy & Education for Chronic Low Back Pain

Follow-Up Flow Chart

Participant Age: _______

Estimated Maximum Heart Rate: _______

Participant First Name (Cohort ID): ________________________

**Visit**			Intake	Visit 2	Visit 3	Visit 4	Visit 5
**Date of Service**							
**Patient Late for Appointment (mins)**							
**Classification***							
***STAB = Stabilization, DP - EX = Extension Directional Preference, DP - FL= Flexion Directional Preference**							
Modfied Oswestry score (%)							
FABQ-W	Baseline:	Book Given: ☐	Coached: ☐	Coached: ☐	Coached: ☐	Coached: ☐	Coached: ☐
**Stabilization Exercise**		Criteria for progression (reps x time)					
Abdominal bracing (supine)		30x8"					
Bracing w/ heel slides (supine)		20x6"					
Bracing w/ leg lifts (supine)		20x6"					
Bracing w/ bridging (supine)		30x8"					
Bracing w/ single leg bridging (supine)		30x8"	R	R	R	R	R
		30x8"	L	L	L	L	L
Bracing (standing)		30x8"					
Bracing w/ standing row (isometric torsion)		20x6"					
Quadruped arm lifts w/ bracing		30x8"					
Quadruped leg lifts w/ bracing		30x8"					
Quadruped opposite arm/leg lifts w/ bracing		30x8"					
Side support w/ knees flexed and abdominal bracing		30x8"	R	R	R	R	R
		30x8"	L	L	L	L	L
Side support w/ knees extended and abdominal bracing		30x8"	R	R	R	R	R
		30x8"	L	L	L	L	L
**Directional Preference Exercise**	Flexion	PT SK DK PSU HS XS SQ					
	Extension	PP PSP PPU PLE SLE					
Aerobic Exercise (total time)							
Resting Heart Rate		High FABQ ONLY!					
Target Heart Rate (10% above resting or above last aerobic HR)							
Target Heart Rate (65-75% of Max)							
Time at Target Heart Rate							
**Treatment Notes**							
**Physical Therapist's Initials**							

Key: Pelvic Tilt: **PT** Single Knee to Chest: **SK** Double Knee to Chest: **DK** Partial Sit-up: **PSU** Hamstring Stretch: **HS** Hip-Flexor Stretch: **XS** Squat: **SQ** Prone Position: **PP** Prone Supported Position: **PSP** Prone Press-ups: **PPU** Progressive Lumbar Extension: **PLE** Standing Lumbar Extension: **SLE**

Back to Health Use Only: Staff Initials ____________________ Date ____________________

Version 2.0

Back to Health — A Study Comparing Yoga, Physical Therapy & Education for Chronic Low Back Pain

Follow-Up Flow Chart

Participant Age: ________

Estimated Maximum Heart Rate: ________

Participant First Name (Cohort ID): ____________________________

**Visit**		Visit 6	Visit 7	Visit 8	Visit 9	Visit 10
**Date of Service**						
**Patient Late for Appointment (mins)**						
**Classification***						
***STAB = Stabilization, DP - EX = Extension Directional Preference, DP - FL= Flexion Directional Preference**						
Modfied Oswestry score (%)						
FABQ-W — Baseline:	Book Given: ☐	Coached: ☐	Coached: ☐	Coached: ☐	Coached: ☐	Coached: ☐
**Stabilization Exercise**	Criteria for progression (reps x time)					
Abdominal bracing (supine)	30x8"					
Bracing w/ heel slides (supine)	20x6"					
Bracing w/ leg lifts (supine)	20x6"					
Bracing w/ bridging (supine)	30x8"					
Bracing w/ single leg bridging (supine)	30x8"	R	R	R	R	R
	30x8"	L	L	L	L	L
Bracing (standing)	30x8"					
Bracing w/ standing row (isometric torsion)	20x6"					
Quadruped arm lifts w/ bracing	30x8"					
Quadruped leg lifts w/ bracing	30x8"					
Quadruped opposite arm/leg lifts w/ bracing	30x8"					
Side support w/ knees flexed and abdominal bracing	30x8"	R	R	R	R	R
	30x8"	L	L	L	L	L
Side support w/ knees extended and abdominal bracing	30x8"	R	R	R	R	R
	30x8"	L	L	L	L	L
**Directional Preference Exercise** — Flexion	PT SK DK PSU HS XS SQ					
**Directional Preference Exercise** — Extension	PP PSP PPU PLE SLE					
Aerobic Exercise (total time)						
Resting Heart Rate	High FABQ ONLY!					
Target Heart Rate (10% above resting or above last aerobic HR)	High FABQ ONLY!					
Target Heart Rate (65-75% of Max)						
Time at Target Heart Rate						
**Treatment Notes**						
**Physical Therapist's Initials**						

Key: Pelvic Tilt: **PT** Single Knee to Chest: **SK** Double Knee to Chest: **DK** Partial Sit-up: **PSU** Hamstring Stretch: **HS** Hip-Flexor Stretch: **XS** Squat: **SQ** Prone Position: **PP** Prone Supported Position: **PSP** Prone Press-ups: **PPU** Progressive Lumbar Extension: **PLE** Standing Lumbar Extension: **SLE**

Back to Health Use Only: Staff Initials ____________________ Date ____________________

Version 2.0

Back to Health — A Study Comparing Yoga, Physical Therapy & Education for Chronic Low Back Pain

Follow-Up Flow Chart

Participant Age: ________

Estimated Maximum Heart Rate: ________

Participant First Name (Cohort ID): ______________________

**Visit**		Visit 11	Visit 12	Visit 13	Visit 14	Visit 15
**Date of Service**						
**Patient Late for Appointment (mins)**						
**Classification***						
***STAB = Stabilization, DP - EX = Extension Directional Preference, DP - FL= Flexion Directional Preference**						
Modfied Oswestry score (%)						
FABQ-W Baseline:	Book Given: ☐	Coached: ☐	Coached: ☐	Coached: ☐	Coached: ☐	Coached: ☐
**Stabilization Exercise**	Criteria for progression (reps x time)					
Abdominal bracing (supine)	30x8"					
Bracing w/ heel slides (supine)	20x6"					
Bracing w/ leg lifts (supine)	20x6"					
Bracing w/ bridging (supine)	30x8"					
Bracing w/ single leg bridging (supine)	30x8"	R	R	R	R	R
	30x8"	L	L	L	L	L
Bracing (standing)	30x8"					
Bracing w/ standing row (isometric torsion)	20x6"					
Quadruped arm lifts w/ bracing	30x8"					
Quadruped leg lifts w/ bracing	30x8"					
Quadruped opposite arm/leg lifts w/ bracing	30x8"					
Side support w/ knees flexed and abdominal bracing	30x8"	R	R	R	R	R
	30x8"	L	L	L	L	L
Side support w/ knees extended and abdominal bracing	30x8"	R	R	R	R	R
	30x8"	L	L	L	L	L
**Directional Preference Exercise** — Flexion	PT SK DK PSU HS XS SQ					
**Directional Preference Exercise** — Extension	PP PSP PPU PLE SLE					
Aerobic Exercise (total time)						
Resting Heart Rate	High FABQ ONLY!					
Target Heart Rate (10% above resting or above last aerobic HR)						
Target Heart Rate (65-75% of Max)						
Time at Target Heart Rate						
**Treatment Notes**						
**Physical Therapist's Initials**						

Key: Pelvic Tilt: **PT** Single Knee to Chest: **SK** Double Knee to Chest: **DK** Partial Sit-up: **PSU** Hamstring Stretch: **HS** Hip-Flexor Stretch: **XS** Squat: **SQ** Prone Position: **PP** Prone Supported Position: **PSP** Prone Press-ups: **PPU** Progressive Lumbar Extension: **PLE** Standing Lumbar Extension: **SLE**

Back to Health Use Only: Staff Initials ____________________ Date ____________________

Version 2.0

Back to Health
A Study Comparing Yoga, Physical Therapy & Education for Chronic Low Back Pain

Follow-Up Flow Chart

Participant Age: _______

Estimated Maximum Heart Rate: _______

Participant First Name (Cohort ID): ________________________

**Visit**		Maintenance 1	Maintenance 2	Maintenance 3	Maintenance 4	Maintenance 5
**Date of Service**						
**Patient Late for Appointment (mins)**						
**Classification***						
***STAB = Stabilization, DP - EX = Extension Directional Preference, DP - FL= Flexion Directional Preference**						
Modfied Oswestry score (%)						
FABQ-W — Baseline:	Book Given: ☐	Coached: ☐	Coached: ☐	Coached: ☐	Coached: ☐	Coached: ☐
**Stabilization Exercise**	Criteria for progression (reps x time)					
Abdominal bracing (supine)	30x8"					
Bracing w/ heel slides (supine)	20x6"					
Bracing w/ leg lifts (supine)	20x6"					
Bracing w/ bridging (supine)	30x8"					
Bracing w/ single leg bridging (supine)	30x8"	R	R	R	R	R
	30x8"	L	L	L	L	L
Bracing (standing)	30x8"					
Bracing w/ standing row (isometric torsion)	20x6"					
Quadruped arm lifts w/ bracing	30x8"					
Quadruped leg lifts w/ bracing	30x8"					
Quadruped opposite arm/leg lifts w/ bracing	30x8"					
Side support w/ knees flexed and abdominal bracing	30x8"	R	R	R	R	R
	30x8"	L	L	L	L	L
Side support w/ knees extended and abdominal bracing	30x8"	R	R	R	R	R
	30x8"	L	L	L	L	L
**Directional Preference Exercise** — Flexion	PT SK DK PSU HS XS SQ					
**Directional Preference Exercise** — Extension	PP PSP PPU PLE SLE					
Aerobic Exercise (total time)						
Resting Heart Rate	High FABQ ONLY!					
Target Heart Rate (10% above resting or above last aerobic HR)						
Target Heart Rate (65-75% of Max)						
Time at Target Heart Rate						
**Treatment Notes**						
**Physical Therapist's Initials**						

Key: Pelvic Tilt: **PT** Single Knee to Chest: **SK** Double Knee to Chest: **DK** Partial Sit-up: **PSU** Hamstring Stretch: **HS** Hip-Flexor Stretch: **XS** Squat: **SQ** Prone Position: **PP** Prone Supported Position: **PSP** Prone Press-ups: **PPU** Progressive Lumbar Extension: **PLE** Standing Lumbar Extension: **SLE**

Back to Health Use Only: Staff Initials ____________________ Date ____________________ *Version 2.0*

Weekly Home Practice Log: Extension Exercises

First Name and Last Initial ONLY: ______________________________

Please let us know when and for how long you did physical therapy exercises at home since your last appointment.

Date:	Prone Lying 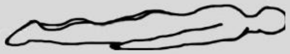	Prone Lying on Elbows 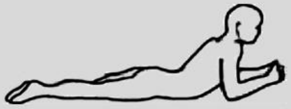	Prone Press-ups 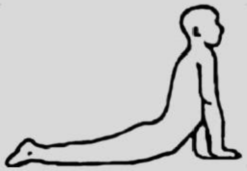	Progressive Extension with Pillows 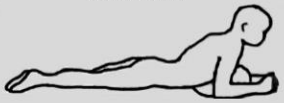	Standing Extension 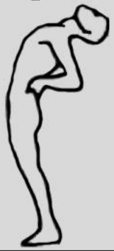
________	☐Yes ☐No<br># reps______	☐Yes ☐No<br># reps______	☐Yes ☐No<br># reps______	☐Yes ☐No<br># reps______	☐Yes ☐No<br># reps______
________	☐Yes ☐No<br># reps______	☐Yes ☐No<br># reps______	☐Yes ☐No<br># reps______	☐Yes ☐No<br># reps______	☐Yes ☐No<br># reps______
________	☐Yes ☐No<br># reps______	☐Yes ☐No<br># reps______	☐Yes ☐No<br># reps______	☐Yes ☐No<br># reps______	☐Yes ☐No<br># reps______
________	☐Yes ☐No<br># reps______	☐Yes ☐No<br># reps______	☐Yes ☐No<br># reps______	☐Yes ☐No<br># reps______	☐Yes ☐No<br># reps______
________	☐Yes ☐No<br># reps______	☐Yes ☐No<br># reps______	☐Yes ☐No<br># reps_____	☐Yes ☐No<br># reps______	☐Yes ☐No<br># reps______
________	☐Yes ☐No<br># reps______	☐Yes ☐No<br># reps______	☐Yes ☐No<br># reps_____	☐Yes ☐No<br># reps______	☐Yes ☐No<br># reps______
________	☐Yes ☐No<br># reps______	☐Yes ☐No<br># reps______	☐Yes ☐No<br># reps_____	☐Yes ☐No<br># reps______	☐Yes ☐No<br># reps______

Comments/Notes:

© Boston Medical Center

Weekly Home Practice Log: Flexion Exercises

First Name and Last Initial ONLY: ______________________

Please let us know when and for how long you did physical therapy exercises at home since your last appointment.

**Date:**	**Pelvic tilt**<br>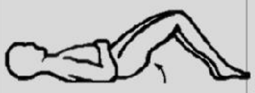	**Single Knee to chest**<br>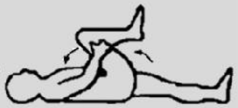	**Double knee to chest**<br>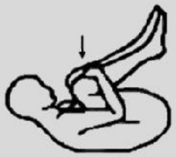	**Partial sit-up**<br>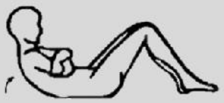	**Hamstring stretch**<br>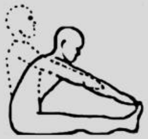	**Hip Flexor stretch**<br>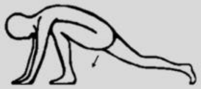	**Squat**<br>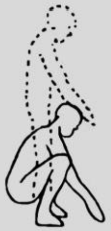
________	☐Yes ☐No<br># reps________	☐Yes ☐No<br># reps________	☐Yes ☐No<br># reps________	☐Yes ☐No<br># reps________	☐Yes ☐No<br># reps________	☐Yes ☐No<br># reps________	☐Yes ☐No<br># reps________
________	☐Yes ☐No<br># reps________	☐Yes ☐No<br># reps________	☐Yes ☐No<br># reps________	☐Yes ☐No<br># reps________	☐Yes ☐No<br># reps________	☐Yes ☐No<br># reps________	☐Yes ☐No<br># reps________
________	☐Yes ☐No<br># reps________	☐Yes ☐No<br># reps________	☐Yes ☐No<br># reps________	☐Yes ☐No<br># reps________	☐Yes ☐No<br># reps________	☐Yes ☐No<br># reps________	☐Yes ☐No<br># reps________
________	☐Yes ☐No<br># reps________	☐Yes ☐No<br># reps________	☐Yes ☐No<br># reps________	☐Yes ☐No<br># reps________	☐Yes ☐No<br># reps________	☐Yes ☐No<br># reps________	☐Yes ☐No<br># reps________
________	☐Yes ☐No<br># reps________	☐Yes ☐No<br># reps________	☐Yes ☐No<br># reps_______	☐Yes ☐No<br># reps________	☐Yes ☐No<br># reps________	☐Yes ☐No<br># reps________	☐Yes ☐No<br># reps________
________	☐Yes ☐No<br># reps________	☐Yes ☐No<br># reps________	☐Yes ☐No<br># reps_______	☐Yes ☐No<br># reps________	☐Yes ☐No<br># reps________	☐Yes ☐No<br># reps________	☐Yes ☐No<br># reps________
________	☐Yes ☐No<br># reps________	☐Yes ☐No<br># reps________	☐Yes ☐No<br># reps_______	☐Yes ☐No<br># reps________	☐Yes ☐No<br># reps________	☐Yes ☐No<br># reps________	☐Yes ☐No<br># reps________

Comments/Notes:

© Boston Medical Center
Program for Integrative Medicine & Health Care Disparities
Back to Health Trial

Weekly Home Practice Log: Stabilization Exercises

First Name and Last Initial ONLY: ______________________________

Please let us know when and for how long you did physical therapy exercises at home since your last appointment.

**Date:**	**Abdominal Bracing** 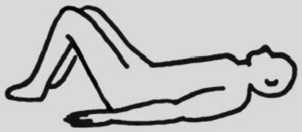 (8 second hold)	**Abdominal Bracing w/ Heel Slide** 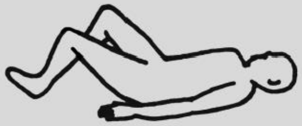 (6 second hold)	**Abdominal Bracing w/ Leg Lifts** 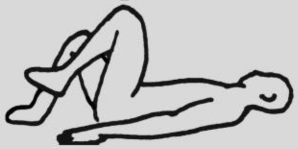 (6 second hold)	**Abdominal Bracing w/ Bridging** 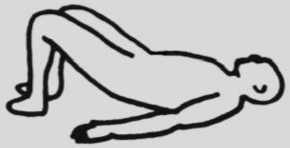 (8 second hold)	**Abdominal Bracing w/ Single Leg Bridging*** 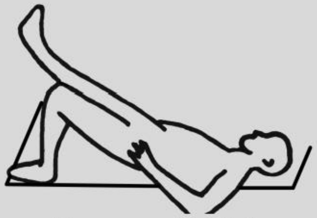 (8 second hold)
_______	☐Yes ☐No<br># reps_______/30	☐Yes ☐No<br># reps_______/20	☐Yes ☐No<br># reps_______/20	☐Yes ☐No<br># reps_______/30	☐Yes ☐No<br># reps_______/30
_______	☐Yes ☐No<br># reps_______/30	☐Yes ☐No<br># reps_______/20	☐Yes ☐No<br># reps_______/20	☐Yes ☐No<br># reps_______/30	☐Yes ☐No<br># reps_______/30
_______	☐Yes ☐No<br># reps_______/30	☐Yes ☐No<br># reps_______/20	☐Yes ☐No<br># reps_______/20	☐Yes ☐No<br># reps_______/30	☐Yes ☐No<br># reps_______/30
_______	☐Yes ☐No<br># reps_______/30	☐Yes ☐No<br># reps_______/20	☐Yes ☐No<br># reps_______/20	☐Yes ☐No<br># reps_______/30	☐Yes ☐No<br># reps_______/30
_______	☐Yes ☐No<br># reps_______/30	☐Yes ☐No<br># reps_______/20	☐Yes ☐No<br># reps_______/20	☐Yes ☐No<br># reps_______/30	☐Yes ☐No<br># reps_______/30
_______	☐Yes ☐No<br># reps_______/30	☐Yes ☐No<br># reps_______/20	☐Yes ☐No<br># reps_______/20	☐Yes ☐No<br># reps_______/30	☐Yes ☐No<br># reps_______/30
_______	☐Yes ☐No<br># reps_______/30	☐Yes ☐No<br># reps_______/20	☐Yes ☐No<br># reps_______/20	☐Yes ☐No<br># reps_______/30	☐Yes ☐No<br># reps_______/30

Comments/Notes:

© Boston Medical Center

Program for Integrative Medicine & Health Care Disparities

Back to Health Trial

Back to Health
A Study Comparing Yoga, Physical Therapy & Education for Chronic Low Back Pain

Weekly Home Practice Log: Stabilization Exercises

First Name and Last Initial ONLY: ______________________

Please let us know when and for how long you did physical therapy exercises at home since your last appointment.

Date:	Abdominal Bracing while Standing 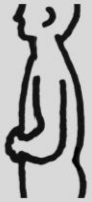 (8 second hold)	Bracing w/ Standing Row 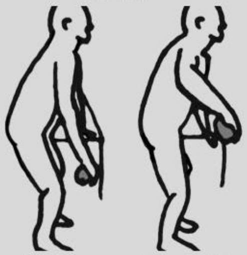 (6 second hold)	Quadruped Arm Lifts w/ Abdominal Bracing 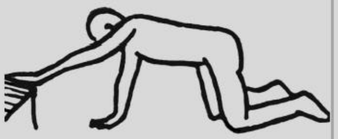 (8 second hold)	Quadruped Leg Lifts w/ Abdominal Bracing 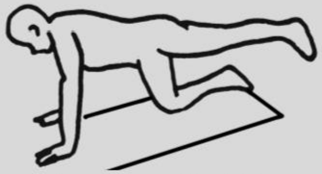 (8 second hold)
________	☐Yes ☐No<br># reps_______/30	☐Yes ☐No<br># reps_______/20	☐Yes ☐No<br># reps_______/30	☐Yes ☐No<br># reps_______/30
________	☐Yes ☐No<br># reps_______/30	☐Yes ☐No<br># reps_______/20	☐Yes ☐No<br># reps_______/30	☐Yes ☐No<br># reps_______/30
________	☐Yes ☐No<br># reps_______/30	☐Yes ☐No<br># reps_______/20	☐Yes ☐No<br># reps_______/30	☐Yes ☐No<br># reps_______/30
________	☐Yes ☐No<br># reps_______/30	☐Yes ☐No<br># reps_______/20	☐Yes ☐No<br># reps_______/30	☐Yes ☐No<br># reps_______/30
________	☐Yes ☐No<br># reps_______/30	☐Yes ☐No<br># reps_______/20	☐Yes ☐No<br># reps_______/30	☐Yes ☐No<br># reps_______/30
________	☐Yes ☐No<br># reps_______/30	☐Yes ☐No<br># reps_______/20	☐Yes ☐No<br># reps_______/30	☐Yes ☐No<br># reps_______/30
________	☐Yes ☐No<br># reps_______/30	☐Yes ☐No<br># reps_______/20	☐Yes ☐No<br># reps_______/30	☐Yes ☐No<br># reps_______/30

Comments/Notes:

© Boston Medical Center
Program for Integrative Medicine & Health Care Disparities
Back to Health Trial

Weekly Home Practice Log: Stabilization Exercises

Back to Health — A Study Comparing Yoga, Physical Therapy & Education for Chronic Low Back Pain

First Name and Last Initial ONLY: ______________________________

Please let us know when and for how long you did physical therapy exercises at home since your last appointment.

Date:	**Quadruped Opposite Arm and Leg Lift w/ Abdominal Bracing** 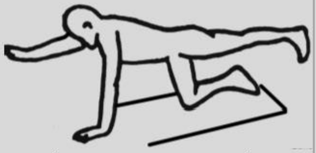 (8 second hold)	**Side Support w/ Knees Flexed and Abdominal Bracing** 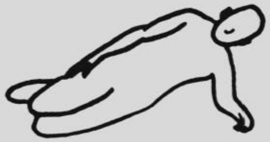 (8 second hold)	**Side Support w/ Knees Extended and Abdominal Bracing** 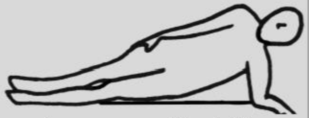 (8 second hold)	**Advanced Side Bridge** 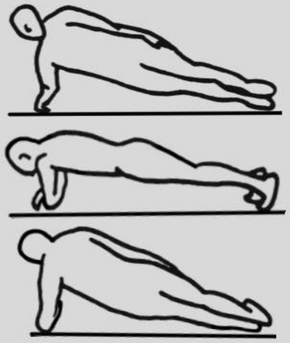
________	☐Yes ☐No # reps_______/30	☐Yes ☐No # reps_______/30	☐Yes ☐No # reps_______/30	☐Yes ☐No # reps_______/30
________	☐Yes ☐No # reps_______/30	☐Yes ☐No # reps_______/30	☐Yes ☐No # reps_______/30	☐Yes ☐No # reps_______/30
________	☐Yes ☐No # reps_______/30	☐Yes ☐No # reps_______/30	☐Yes ☐No # reps_______/30	☐Yes ☐No # reps_______/30
________	☐Yes ☐No # reps_______/30	☐Yes ☐No # reps_______/30	☐Yes ☐No # reps_______/30	☐Yes ☐No # reps_______/30
________	☐Yes ☐No # reps_______/30	☐Yes ☐No # reps_______/30	☐Yes ☐No # reps_______/30	☐Yes ☐No # reps_______/30
________	☐Yes ☐No # reps_______/30	☐Yes ☐No # reps_______/30	☐Yes ☐No # reps_______/30	☐Yes ☐No # reps_______/30
________	☐Yes ☐No # reps_______/30	☐Yes ☐No # reps_______/30	☐Yes ☐No # reps_______/30	☐Yes ☐No # reps_______/30

Comments/Notes:

STAFF USE ONLY

COLLECTION	DATA ENTRY
Name:______________ Date:__________	Name:______________ Date:_________
Signature: _________________	Signature: _________________

Over the Next 9 Months

1. **Continue performing the recommended exercises.**
   Continue to practice the physical therapy exercises at home and fill out your **Weekly Home Practice Log** each week which will be collected by the research team. Don't worry if you don't have time every day to complete the exercises, just do what works best for you and your schedule.

2. **Take care of yourself.**
   Continue normal activities. Please notify your physical therapist during an in-person session or call the research study team if you experience *any* injury during the course of the study. The Back to Health research team may be reached at **617-414-6211** to report an injury or if you have any questions or concerns.

3. **Attend survey dates.**
   You will be contacted to schedule a time and place to take your surveys. If you would like to reschedule, please contact our research team.

4. **Keep in Touch.**
   Please let us know if your address or phone number changes. Please contact the research team if your contact information needs to be updated (for example, if your mailing address or telephone number changes).

We hope that you have a wonderful experience as a participant in the Back to Health Study and thank you for your participation!

The Back to Health Research Team
617-414-6211

Fear Avoidance Belief Questionnaire (FABQ)

The follow statements are things that other patients have told us about their back pain. For each statement, please rate how you much you agree or disagree where "0" is "completely disagree" and "6" is "completely agree."

1. My pain was caused by physical activity.

0	1	2	3	4	5	6
**Completely Disagree**			**Unsure**			**Completely Agree**

2. Physical activity makes my pain worse.

0	1	2	3	4	5	6
**Completely Disagree**			**Unsure**			**Completely Agree**

3. Physical activity might harm my back.

0	1	2	3	4	5	6
**Completely Disagree**			**Unsure**			**Completely Agree**

4. I should not do physical activities which might make my pain worse.

0	1	2	3	4	5	6
**Completely Disagree**			**Unsure**			**Completely Agree**

5. I cannot do physical activities which might make my pain worse.

0	1	2	3	4	5	6
**Completely Disagree**			**Unsure**			**Completely Agree**

The following statements are about how your normal work affects or would affect your back pain. Again, for each statement, please rate how you much you agree or disagree where "0" is "completely disagree" and "6" is "completely agree."

6. My pain was caused by my work or an accident at work.

0	1	2	3	4	5	6
**Completely Disagree**			**Unsure**			**Completely Agree**

7. My work aggravated my pain.

0 1 2 3 4 5 6
Completely Disagree — Unsure — Completely Agree

8. I have a claim for compensation for my pain.

0 1 2 3 4 5 6
Completely Disagree — Unsure — Completely Agree

9. My work is too heavy for me.

0 1 2 3 4 5 6
Completely Disagree — Unsure — Completely Agree

10. My work makes or would make my pain worse.

0 1 2 3 4 5 6
Completely Disagree — Unsure — Completely Agree

11. My work might harm my back.

0 1 2 3 4 5 6
Completely Disagree — Unsure — Completely Agree

12. I should not do my normal work with my present pain.

0 1 2 3 4 5 6
Completely Disagree — Unsure — Completely Agree

13. I cannot do my normal work with my present pain.

0 1 2 3 4 5 6
Completely Disagree — Unsure — Completely Agree

14. I cannot do my normal work until my pain is treated.

0 1 2 3 4 5 6
Completely Disagree — Unsure — Completely Agree

15. I do not think that I will be back to my normal work within 3 months.

0	1	2	3	4	5	6
**Completely Disagree**			**Unsure**			**Completely Agree**

16. I do not think that I will ever be able to go back to that work.

0	1	2	3	4	5	6
**Completely Disagree**			**Unsure**			**Completely Agree**

Scoring:

FABQPA—sum questions 2, 3, 4, and 5

FABQW—sum questions 6, 7, 9, 10, 11, 12, and 15

Modified Oswestry Questionnaire

1. In regards to your **pain intensity**, which of the following statements is **most true** for you in your current condition:

   0☐ I can tolerate the pain I have without having to use pain medication.
   1☐ The pain is bad, but I can manage without having to take pain medication.
   2☐ Pain medication provides me with complete relief from pain.
   3☐ Pain medication provides me with moderate relief from pain.
   4☐ Pain medication provides me with little relief from pain.
   5☐ Pain medication has no effect on my pain.

2. In regards to your **personal care** (e.g., washing and dressing), which of the following statements is **most true** for you in your current condition:

   0☐ I can take care of myself normally without causing increased pain.
   1☐ I can take care of myself normally, but it increases my pain.
   2☐ It is painful to take care of myself, and I am slow and careful.
   3☐ I need help, but I am able to manage most of my personal care.
   4☐ I need help every day in most aspects of my care.
   5☐ I do not get dressed, I wash with difficulty, and I stay in bed.

3. In regards to **lifting** (e.g. heaving objects), which of the following statements is most true for you in your current condition:

   0☐ I can lift heavy weights without increased pain.
   1☐ I can lift heavy weights, but it causes increased pain.
   2☐ Pain prevents me from lifting heavy weights off the floor, but I can manage if the weights are conveniently positioned (e.g., on a table).
   3☐ Pain prevents me from lifting heavy weights, but I can manage light to medium weights if they are conveniently positioned.
   4☐ I can lift only very light weights.
   5☐ I cannot lift or carry anything at all.

4. In regards to **walking**, which of the following statements is **most true** for you in your current condition:
   0☐ Pain does not prevent me from walking any distance.
   1☐ Pain prevents me from walking more than 1 mile. (1 mile = 1.6 km).
   2☐ Pain prevents me from walking more than 1/2 mile.
   3☐ Pain prevents me from walking more than 1/4 mile.
   4☐ I can walk only with crutches or a cane.
   5☐ I am in bed most of the time and have to crawl to the toilet.

5. In regards to **sitting**, which of the following statements is **most true** for you in your current condition:

   0☐ I can sit in any chair as long as I like.
   1☐ I can only sit in my favorite chair as long as I like.
   2☐ Pain prevents me from sitting for more than 1 hour.
   3☐ Pain prevents me from sitting for more than 1/2 hour.
   4☐ Pain prevents me from sitting for more than 10 minutes.
   5☐ Pain prevents me from sitting at all.

6. In regards to **<u>standing</u>**, which of the following statements is **<u>most true</u>** for you in your current condition:

0☐ I can stand as long as I want without increased pain.
1☐ I can stand as long as I want, but it increases my pain.
2☐ Pain prevents me from standing for more than 1 hour.
3☐ Pain prevents me from standing for more than 1/2 hour.
4☐ Pain prevents me from standing for more than 10 minutes.
5☐ Pain prevents me from standing at all.

7. In regards to **<u>sleeping</u>**, which of the following statements is **<u>most true</u>** for you in your current condition:

0☐ Pain does not prevent me from sleeping well.
1☐ I can sleep well only by using pain medication.
2☐ Even when I take medication, I sleep less than 6 hours.
3☐ Even when I take medication, I sleep less than 4 hours.
4☐ Even when I take medication, I sleep less than 2 hours.
5☐ Pain prevents me from sleeping at all.

8. In regards to **<u>your social life</u>**, which of the following statements is **<u>most true</u>** for you in your current condition:

0☐ My social life is normal and does not increase my pain.
1☐ My social life is normal, but it increases my level of pain.
2☐ Pain prevents me from participating in more energetic activities (e.g., sports, dancing).
3☐ Pain prevents me form going out very often.
4☐ Pain has restricted my social life to my home.
5☐ I have hardly any social life because of my pain.

9. In regards to **<u>traveling</u>**, which of the following statements is **<u>most true</u>** for you in your current condition:

0☐ I can travel anywhere without increased pain.
1☐ I can travel anywhere, but it increases my pain.
2☐ My pain restricts my travel over 2 hours.
3☐ My pain restricts my travel over 1 hour.
4☐ My pain restricts my travel to short necessary journeys under 1/2 hour.
5☐ My pain prevents all travel except for visits to the physician / therapist or hospital.

10. In regards to **<u>your employment or homemaking</u>**, which of the following statements is **<u>most true</u>** for you in your current condition:

0☐ My normal homemaking / job activities do not cause pain.
1☐ My normal homemaking / job activities increase my pain, but I can still perform all that is required of me.
2☐ I can perform most of my homemaking / job duties, but pain prevents me from performing more physically stressful activities (e.g., lifting, vacuuming).
3☐ Pain prevents me from doing anything but light duties.
4☐ Pain prevents me from doing even light duties.
5☐ Pain prevents me from performing any job or homemaking chores.

APPENDIX B: Physical Therapy Examination Procedures

Deep Tendon Reflex (DTR) Testing

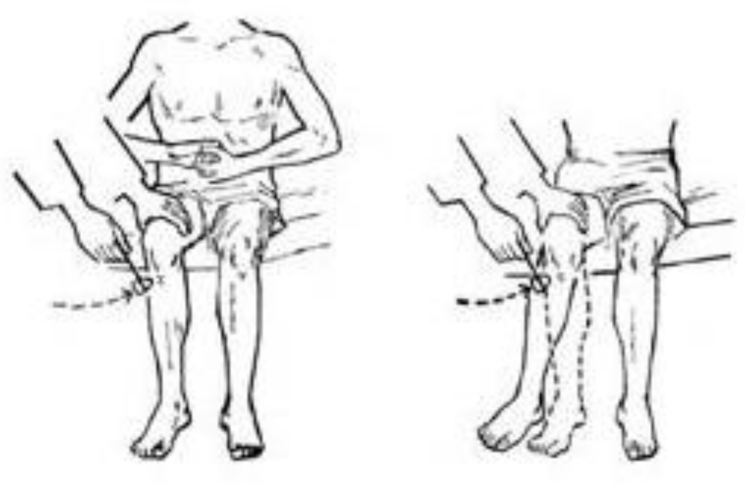

Motor Examination

Ankle Dorsiflexion: anterior tibialis to test the L4 nerve root

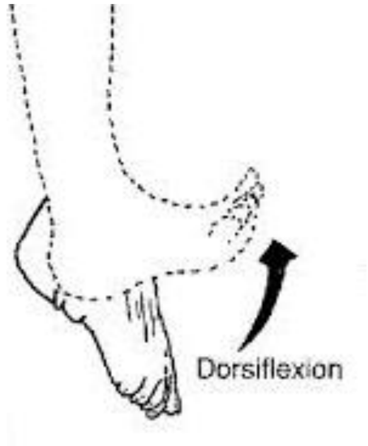

Great Toe Extension: Extensor hallucis longus (EHL) to test the L5 nerve root

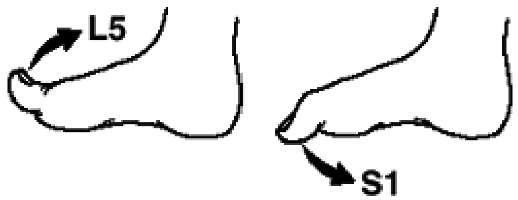

© Boston Medical Center

Ankle Eversion: Peroneals to test the L5 and S1 roots

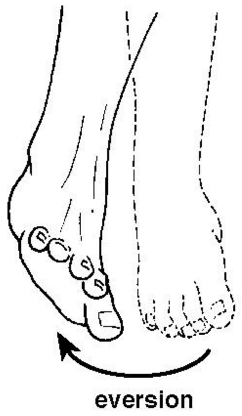

Ankle Plantar Flexion: Gastroc/soleus to test the S1 and S2 roots

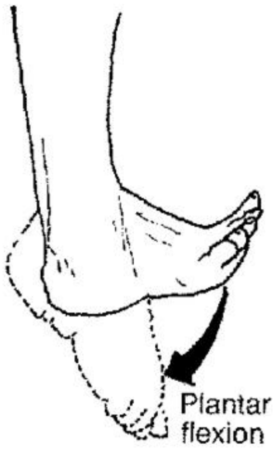

<u>Sensory examination</u>

Medial lower leg/foot to test L4 nerve root

Lateral leg/foot to test the L5 nerve root

Lateral side of foot to test the S1 nerve root

Aberrant movements (standing)

Record as 'positive' (i.e. "Yes") if any of the following are observed during sagittal plane motion (forward flexion and return to upright):

- Instability catch - any trunk movement outside of the plane of specified motion (e.g., side bending during trunk flexion).
- Painful arc - symptoms felt during the movement at a particular point in the motion (or through a particular portion of the range) that are not present before or after this point. A painful arc may occur in flexion or return from flexion.
- Thigh climbing - using the hands on thighs (or some other external support) to push up on when returning from flexion to upright
- Reversal of lumbopelvic rhythm - the trunk being extended first, followed by extension of the hips and pelvis to bring the body back to upright standing from a forward flexed position

Lumbar flexion range of motion (ROM) (standing)

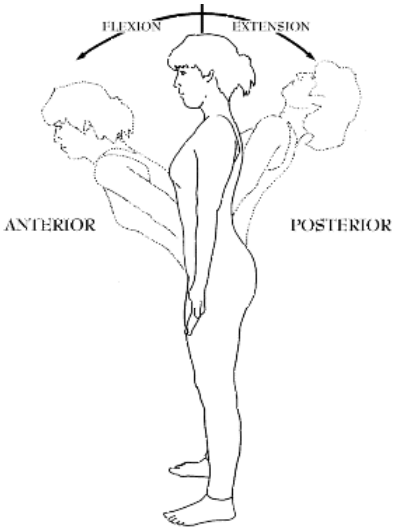

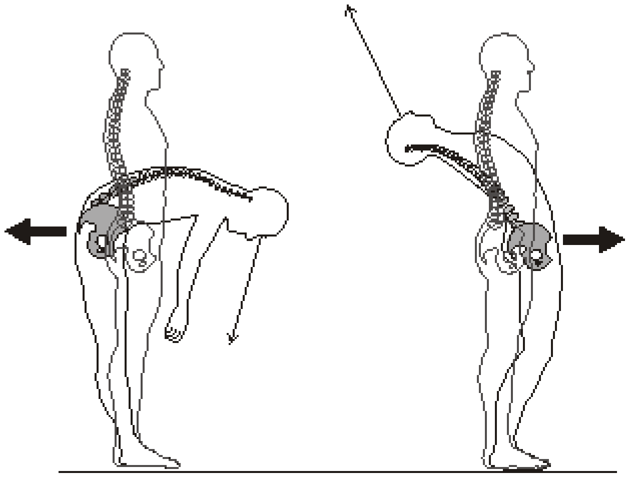

Average straight leg raise (SLR) (supine)

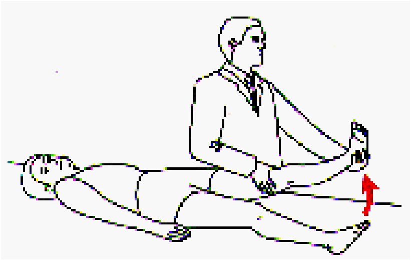

Spinal segment mobility (spring) testing (prone)

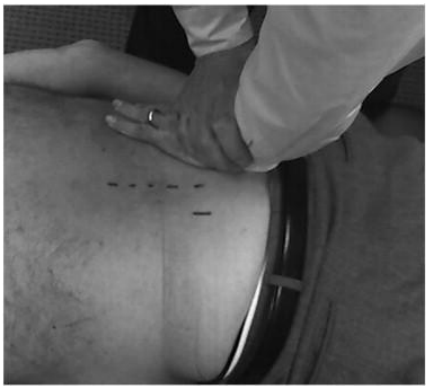

Prone instability test (prone)

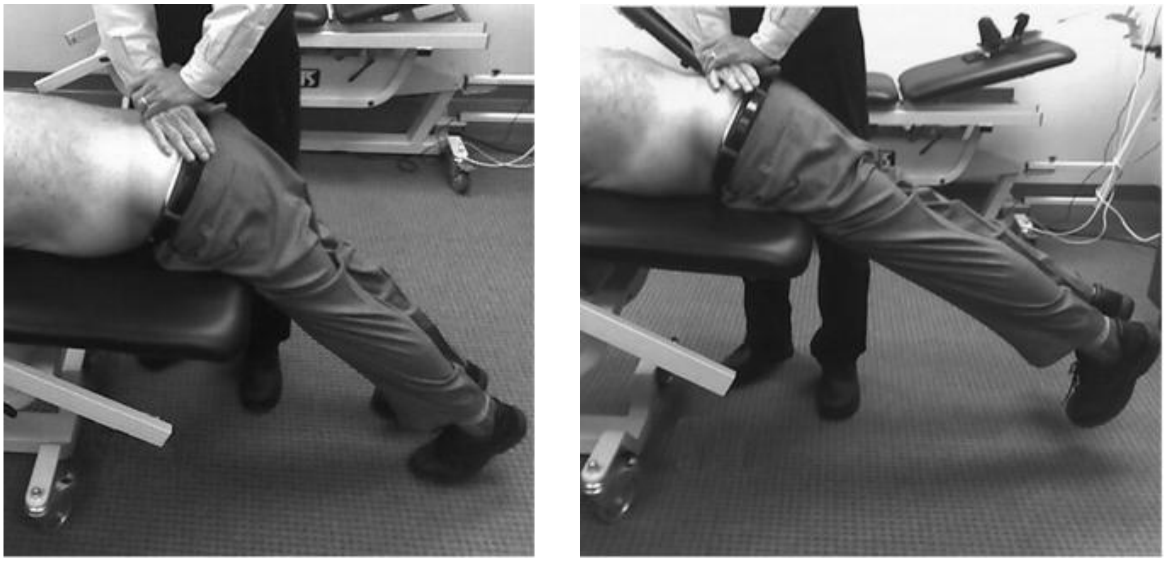

© Boston Medical Center

APPENDIX C: *The Back Book** Content Summary

Back Facts Page 2

Causes of Back Pain Page 3

Rest or Stay Active? Page 4

Activity is Good Page 5

Dealing with an Attack of Back Pain Page 7

The Risk of Chronic Pain Page 12

How to Stay Active Page 14

Getting on with Your Life Page 16

It's Your Back Page 18

* Roland M, Waddell G, Klaber Moffet K, Burton K, Main C. *The Back Book*. Norwich, UK: The Stationary Office; 1996.

Appendix D: Physical Therapy Terminology

Abduction: lateral movement away from the midline of the body

Adduction: medial movement toward the midline of the body

Anatomical position: body upright, feet together, arms hanging at sides, palms facing forward, thumbs facing away from body, fingers extended

Anterior (ventral): toward front

Circumduction: circular movement (combining flexion, extension, adduction, and abduction) with no shaft rotation

Deep: further from the surface

Distal: farther away from reference point or away from vertex of head

Eversion: moving sole of foot away from medial plane

Extension: straightening the joint resulting in an increase of angle

Flexion: bending the joint resulting in a decrease of angle

Hyperextension: extending the joint beyond anatomical position

Inferior: away from vertex of head

Inversion: moving sole of foot toward medial plane

Lateral: away from sagittal midline of body

Long sitting: sitting position, with legs in front (e.g., on a floor or other surface)

Medial: toward sagittal midline of body

Posterior (dorsal): toward back

Pronation: internal rotation resulting in appendage facing downward

Prone: laying on stomach, face down

Protrusion: moving anteriorly (e.g., chin out)

Proximal: closer to reference point or toward vertex of head

Retrusion: moving posteriorly (e.g., chin in)

Rotation: rotary movement around the longitudinal axis of the bone

Superficial: closer to the surface

Superior: toward vertex of head

Supination: external rotation resulting in appendage facing upward

Supine: laying on back, face up

© Boston Medical Center

Appendix E: Relevant Literature

Amick BC, III, Lerner D, Rogers WH, Rooney T, Katz JN. A review of health-related work outcome measures and their uses, and recommended measures. Spine 2000;25:3152-3160.

Andersson GB. Epidemiological features of chronic low-back pain. Lancet 1999;354:581-585.

Binkley J, Finch E, Hall J, Black T, Gowland C. Diagnostic classification of patients with low back pain: report on a survey of physical therapy experts. Phys Ther 1993;73:138-150.

Bombardier C. Outcome assessments in the evaluation of treatment of spinal disorders: summary and general recommendations. Spine 2000;25:3100-3103.

Borkan JM, Koes B, Reis S, Cherkin DC. A report from the Second International Forum for Primary Care Research on Low Back Pain. Reexamining priorities. Spine 1998;23:1992-1996.

Brennan GP, Fritz JM, Hunter SJ, Thackeray A, Delitto A, Erhard RE. Identifying subgroups of patients with acute/subacute "nonspecific" low back pain: results of a randomized clinical trial. Spine 2006;31:623-631.

Browder DA, Childs JD, Cleland JA, Fritz JM. Effectiveness of an extension-oriented treatment approach in a subgroup of subjects with low back pain: a randomized clinical trial. Phys Ther 2007;87:1608-1618.

Carey TS, Garrett JM. The relation of race to outcomes and the use of health care services for acute low back pain. Spine 2003;28:390-394.

Cherkin DC, Deyo RA, Battie M, Street J, Barlow W. A comparison of physical therapy, chiropractic manipulation, and provision of an educational booklet for the treatment of patients with low back pain. N Engl J Med 1998;339:1021-1029.

Childs JD, Fritz JM, Flynn TW et al. A clinical prediction rule to identify patients with low back pain most likely to benefit from spinal manipulation: a validation study. Ann Intern Med 2004;141:920-928.

Chou R, Huffman LH. Nonpharmacologic therapies for acute and chronic low back pain: a review of the evidence for an American Pain Society/American College of Physicians clinical practice guideline. Ann Intern Med 2007;147:492-504.

Chou R, Qaseem A, Snow V et al. Diagnosis and treatment of low back pain: a joint clinical practice guideline from the American College of Physicians and the American Pain Society. Ann Intern Med 2007;147:478-491.

Dagenais S, Caro J, Haldeman S. A systematic review of low back pain cost of illness studies in the United States and internationally. Spine J 2008;8:8-20.

Delitto A, Erhard RE, Bowling RW. A treatment-based classification approach to low back syndrome: identifying and staging patients for conservative treatment. Phys Ther 1995;75:470-485.

Delitto A, Shulman AD, Rose SJ. On developing expert-based decision-support systems in physical therapy: the NIOSH low back atlas. Phys Ther 1989;69:554-558.

Deyo RA, Mirza SK, Martin BI. Back pain prevalence and visit rates: estimates from U.S. national surveys, 2002. Spine 2006;31:2724-2727.

Deyo RA, Weinstein JN. Low back pain. N Engl J Med 2001;344:363-370.

Fishbain DA, Cutler RB, Cole B et al. Are patients with chronic low back pain or chronic neck pain fatigued? Pain Med 2004;5:187-195.

Freburger JK, Carey TS, Holmes GM. Physician referrals to physical therapists for the treatment of spine disorders. Spine J 2005;5:530-541.

Freburger JK, Holmes GM, Carey TS. Physician referrals to physical therapy for the treatment of musculoskeletal conditions. Arch Phys Med Rehabil 2003;84:1839-1849.

Fritz JM, Brennan GP, Clifford SN, Hunter SJ, Thackeray A. An examination of the reliability of a classification algorithm for subgrouping patients with low back pain. Spine 2006;31:77-82.

Fritz JM, Cleland JA, Brennan GP. Does adherence to the guideline recommendation for active treatments improve the quality of care for patients with acute low back pain delivered by physical therapists? Med Care 2007;45:973-980.

Fritz JM, Cleland JA, Childs JD. Subgrouping patients with low back pain: evolution of a classification approach to physical therapy. J Orthop Sports Phys Ther 2007;37:290-302.

Fritz JM, Delitto A, Erhard RE. Comparison of classification-based physical therapy with therapy based on clinical practice guidelines for patients with acute low back pain: a randomized clinical trial. Spine 2003;28:1363-1371.

Fritz JM, Delitto A, Vignovic M, Busse RG. Interrater reliability of judgments of the centralization phenomenon and status change during movement testing in patients with low back pain. Arch Phys Med Rehabil 2000;81:57-61.

Fritz JM, George S. The use of a classification approach to identify subgroups of patients with acute low back pain. Interrater reliability and short-term treatment outcomes. Spine 2000;25:106-114.

© Boston Medical Center

Fritz JM, Whitman JM, Childs JD. Lumbar spine segmental mobility assessment: an examination of validity for determining intervention strategies in patients with low back pain. Arch Phys Med Rehabil 2005;86:1745-1752.

George SZ, Bialosky JE, Fritz JM. Physical therapist management of a patient with acute low back pain and elevated fear-avoidance beliefs. Phys Ther 2004;84:538-549.

George SZ, Delitto A. Clinical examination variables discriminate among treatment-based classification groups: a study of construct validity in patients with acute low back pain. Phys Ther 2005;85:306-314.

George SZ, Fritz JM, Bialosky JE, Donald DA. The effect of a fear-avoidance-based physical therapy intervention for patients with acute low back pain: results of a randomized clinical trial. Spine 2003;28:2551-2560.

George SZ, Fritz JM, Childs JD. Investigation of elevated fear-avoidance beliefs for patients with low back pain: a secondary analysis involving patients enrolled in physical therapy clinical trials. J Orthop Sports Phys Ther 2008;38:50-58.

Hall H, McIntosh G. Low back pain (chronic). Clin Evid 2008.

Hayden JA, van Tulder MW, Malmivaara AV, Koes BW. Meta-analysis: exercise therapy for nonspecific low back pain. Ann Intern Med 2005;142:765-775.

Hayden JA, van Tulder MW, Tomlinson G. Systematic review: strategies for using exercise therapy to improve outcomes in chronic low back pain. Ann Intern Med 2005;142:776-785.

Haythornthwaite JA, Wegener S, Benrud-Larson L et al. Factors associated with willingness to try different pain treatments for pain after a spinal cord injury. Clin J Pain 2003;19:31-38.

Heiss DG, Fitch DS, Fritz JM, Sanchez WJ, Roberts KE, Buford JA. The interrater reliability among physical therapists newly trained in a classification system for acute low back pain. J Orthop Sports Phys Ther 2004;34:430-439.

Hicks GE, Fritz JM, Delitto A, McGill SM. Preliminary development of a clinical prediction rule for determining which patients with low back pain will respond to a stabilization exercise program. Arch Phys Med Rehabil 2005;86:1753-1762.

Hicks GE, Fritz JM, Delitto A, Mishock J. Interrater reliability of clinical examination measures for identification of lumbar segmental instability. Arch Phys Med Rehabil 2003;84:1858-1864.

Hides JA, Jull GA, Richardson CA. Long-term effects of specific stabilizing exercises for first-episode low back pain. Spine 2001;26:E243-E248.

Jette AM, Davis KD. A comparison of hospital-based and private outpatient physical therapy practices. Phys Ther 1991;71:366-375.

Kellett KM, Kellett DA, Nordholm LA. Effects of an exercise program on sick leave due to back pain. Phys Ther 1991;71:283-291.

Koes BW, van Tulder MW, Ostelo R, Kim BA, Waddell G. Clinical guidelines for the management of low back pain in primary care: an international comparison. Spine 2001;26:2504-2513.

Lethem J, Slade PD, Troup JD, Bentley G. Outline of a Fear-Avoidance model of exaggerated pain perception—I. Behav Res Ther 1983;21:401-408.

Licciardone JC. The epidemiology and medical management of low back pain during ambulatory medical care visits in the United States. Osteopath Med Prim Care 2008;2:11.

Long A, Donelson R, Fung T. Does it matter which exercise? A randomized control trial of exercise for low back pain. Spine 2004;29:2593-2602.

Luo X, Pietrobon R, Sun SX, Liu GG, Hey L. Estimates and patterns of direct health care expenditures among individuals with back pain in the United States. Spine 2004;29:79-86.

Protas EJ, Mayer TG, Dersh J, Keeley J, Gatchel RJ, McGeary D. Relevance of aerobic capacity measurements in the treatment of chronic work-related spinal disorders. Spine 2004;29:2158-2166.

Razmjou H, Kramer JF, Yamada R. Intertester reliability of the McKenzie evaluation in assessing patients with mechanical low-back pain. J Orthop Sports Phys Ther 2000;30:368-383.

Riddle DL. Classification and low back pain: a review of the literature and critical analysis of selected systems. Phys Ther 1998;78:708-737.

Ritter PL, Gonzalez VM, Laurent DD, Lorig KR. Measurement of pain using the visual numeric scale. J heumatol 2006;33:574-580.

Roland M, Waddell G, Klaber-Moffett K, et al. The Back Book. Norwich, UK: The Stationary Office, 1996.

Shaughnessy M, Caulfield B. A pilot study to investigate the effect of lumbar stabilisation exercise training on functional ability and quality of life in patients with chronic low back pain. Int J Rehabil Res 2004;27:297-301.

Slade PD, Troup JD, Lethem J, Bentley G. The Fear-Avoidance model of exaggerated pain perception—II. Behav Res Ther 1983;21:409-416.

© Boston Medical Center

Spinhoven P, Ter KM, Kole-Snijders AM, Hutten MM, Den Ouden DJ, Vlaeyen JW. Catastrophizing and internal pain control as mediators of outcome in the multidisciplinary treatment of chronic low back pain. Eur J Pain 2004;8:211-219.

Taylor NF, Dodd KJ, Damiano DL. Progressive resistance exercise in physical therapy: a summary of systematic reviews. Phys Ther 2005;85:1208-1223.

Van Dillen LR, Sahrmann SA, Norton BJ et al. Reliability of physical examination items used for classification of patients with low back pain. Phys Ther 1998;78:979-988.

Vlaeyen JW, Linton SJ. Fear-avoidance and its consequences in chronic musculoskeletal pain: a state of the art. Pain 2000;85:317-332.

Von KM, Jensen MP, Karoly P. Assessing global pain severity by self-report in clinical and health services research. Spine 2000;25:3140-3151.

Waddell G, Newton M, Henderson I, Somerville D, Main CJ. A Fear-Avoidance Beliefs Questionnaire (FABQ) and the role of fear-avoidance beliefs in chronic low back pain and disability. Pain 1993;52:157-168.

Werneke MW, Hart DL, George SZ, Stratford PW, Matheson JW, Reyes A. Clinical outcomes for patients classified by fear-avoidance beliefs and centralization phenomenon. Arch Phys Med Rehabil 2009;90:768-777.

Whitman JM, Fritz JM, Childs JD. The influence of experience and specialty certifications on clinical outcomes for patients with low back pain treated within a standardized physical therapy management program. J Orthop Sports Phys Ther 2004;34:662-672.

© Boston Medical Center
