# Supplementary material for: Yoga vs. physical therapy vs. education for chronic low back pain in predominantly minority populations: study protocol for a randomized controlled trial
Source: Trials. 2014 Feb 26;15:67. doi: 10.1186/1745-6215-15-67 (PMC3944007; doi:10.1186/1745-6215-15-67)
Supplement: Additional file 4 — Education Newsletters. One- to two-page newsletters mailed to education participants at 3, 6, 9, and 12 weeks written at a 6th grade level that highlight main points from the assigned chapters of The Back Pain Helpbook[60]. [file 1745-6215-15-67-S4.pdf]

Education Newsletter Volume 1

How to Safely Resume Your Normal Activities

**What should I do if I'm having severe pain?**	Use gentle stretching. This will help you reduce the tension in your back.
**What if I am having a severe flare-up and it is too painful to move?**	You may find that using medication, and either heat or ice, makes it easier to stretch and get moving again.
**What should I be doing as my severe pain gets better?**	Continue with your normal activities within 1 to 3 days as your worst pain gets better. Try to keep up with as many household chores, family outings, and other routine activities as possible.
**Because of my back pain, should I be doing fewer activities?**	No. You should gradually increase your activities over a few days or up to a week.
**What if an activity increases my pain throughout the day?**	If an activity causes increased pain for the rest of the day, do less of the activity or find a substitute.
**When I have a flare-up, I like to stay in bed or frozen in one position.**	Do not do this! Movement, even if uncomfortable, can help you get through a flare-up quickly. You can start by walking within your home. Try walking for a few minutes every hour.
**So I should push through the pain?**	Not necessarily. Pace yourself and use common sense. Give yourself permission to take it easy for a few days until the worse pain goes down. Pushing through severe pain might trigger a second back spasm.
**After a flare-up, should I do as little as possible?**	No. Doing too little may lead to a longer period of pain. Pacing yourself means finding out how much of an activity you can tolerate, and then performing that much on a consistent basis.

© Boston Medical Center

Education Newsletter Volume 2

Effective Self Care

4 Steps to Making a Plan of Action

1. **Exactly what you are going to do:** For example, is your plan to start a walking program, do stretching exercises, or perhaps begin to do social activities that you have been putting off?
2. **How much you will do:** For example, will you walk for 15 or 50 minutes?
3. **When will you do it:** Will you walk before breakfast, during your lunch break, or at night after the evening news?
4. **How often you will do the activity:** It is best to say that you will do something five times a week rather than every day? That way, if you miss the activity one day you will not feel guilty or feel like you have failed.

The Three R's for Managing Flare-Ups

Relieve severe pain and inflammation during a flare-up.

Resume your normal activities as the flare-up goes down. Do not wait until you are free of pain; begin to stretch, exercise, and take up your normal activities.

Restore your self-confidence and emotional well-being. It is important to identify and address the worry, distress, and negative thoughts that come with a severe episode of back pain.

How to Talk to Your Doctor about Back Pain

- Be specific when you talk to your doctor about what is worrying you.
- Describe what the pain is preventing you from doing.
- Tell your doctor whether you want a prescription pain reliever.
- Have realistic expectations.
- Ask questions.
- If you are interested, ask for information about other pain-reducing techniques.
- Tell your doctor about any pain control methods you may already be using.

Tips for Taking Over-the-Counter Medicine

(more tips listed on page 66)

- Choose medicine with a single ingredient, for example, some pain relievers have caffeine in them, which can disrupt your sleep.
- Read the label carefully.
- Ask the pharmacist for help.
- Avoid mixing medicines.
- Do not exceed the recommended dosage.
- Never use a drug from an unlabeled container.

© Boston Medical Center
Program for Integrative Medicine & Health Care Disparities
Back to Health Trial

Managing Flare-Ups and Emergencies

**Do not panic** ⬇	Avoid negative thoughts and don't get upset.
**Find out whether the problem is a serious risk to your health** ⬇	Read the "Red Flags of Serious Illness" on page 17. If you have any of these symptoms, contact your doctor and the *Back to Health* study staff as soon as possible.
**Control your pain** ⬇	If you are in severe pain, take medicine right away.
**Ice the painful area** ⬇	Whether you are having back or leg pain, use ice on the lower back. Do not use heat because it might increase swelling.
**Take it easy** ⬇	It is ok to lie down and rest for a while but do not stay in bed any longer than necessary. Try to get up and move around after 30 to 60 minutes.
**Use relaxation, distraction, and reassuring self-talk** ⬇	Try meditation or focused breathing. Go to chapter 9 to learn these methods.
**Slowly start to move** ⬇	The sooner you start to move, the better. If you are in too much pain to move at first, try again after taking pain medicine and using ice.
**Stretch** ⬇	Stretching will help you get through a flare-up faster, so begin gentle stretching as soon as possible. Expect some discomfort, but avoid stretching that causes severe pain.
**Keep up your strength and endurance** ⬇	Within a couple of days you should be able to return to some light strengthening and endurance exercises.
**Continue aerobic exercise** ⬇	Once the worst of your pain has decreased, you can start with walking on level ground at a pace and distance that is comfortable to you.
**Ways to control pain**	After you have recovered, it is helpful to keep using over-the-counter medications, heat, ice, and other methods. You are also better off going to work and doing your normal activities.

Back to Health — A Study Comparing Yoga, Physical Therapy & Education for Chronic Low Back Pain

Physical Methods for Back Pain

- Exercise: The best for managing back pain.
- Applying Cold and Heat: For a sudden flare-up, applying ice is the most helpful. If ice does not work for you, try using a heating pad.
- Massage: A gentle, stroking massage is the safest.
- Spinal Manipulation and Mobilization: Performed by chiropractors and osteopaths.
- Traction, "Deep Heat," and Other Techniques: read about other pain control techniques on pages 80 – 81.

Mind-Body Techniques

- Breathe Deeply to Relieve Stress: Relaxing to reduce stress can lower muscle tension and back pain.
- Progressive Muscle Relaxation: Helps you find muscle tension before it builds to pain. Learn how to do this at the bottom of page 87, continuing on pages 88 – 89.
- "Letting Go" Relaxation: Similar to progressive muscle relaxation, but it does not require you to tighten your muscles. Learn how to do this on pages 89 – 90.
- Meditation and Prayer: Involves concentrating on your breathing and repetition of the special word or phrase.
- Guided Imagery: Similar to daydreaming. Close your eyes, and ask a family member or a friend read the script on pages 91 – 92.

Using Deep Breathing as a Relaxation Tool

- Make time for deep breathing: It only takes a few minutes each day.
- Get as comfortable as possible: Sit in a chair with back support or lie comfortably on the floor or bed.
- Monitor your breathing: Place one hand on your stomach and one on your chest. Practice breathing until the hand on your stomach moves more than the one on your chest.
- Let go of tension: Inhale through your nose and exhale through slightly puckered lips. Notice that you feel more relaxed as you exhale.

Tips for Coping with Depression

- Visit or call friends, go on outings, join a group, or volunteer.
- Make plans and carry them out.
- Remain as active and involved in normal activities as possible.
- Exercise regularly.
- Go to work, accomplish things at home, or spend time on a hobby.
- Reward yourself when you accomplish something.
- Have fun.
- Avoid alcohol.
- If you are taking medicine, talk to your doctor about any possible side effects.
- Seek professional help.

© Boston Medical Center
Program for Integrative Medicine & Health Care Disparities
Back to Health Trial

Education Newsletter Volume 3
Physical Activity and Exercise

Four Basics to Good Mechanics

1. When lifting or carrying something, keep the weight close to your body.
1. Keeps your ears, shoulders, and hips in line.
2. Keep your back straight and do no slouch.
3. Try not to twist your back.

Take a look at the pictures and descriptions of how to perform everyday activities on pages 126 – 137.

Activities that include:
- Waking up, using the sink, and getting dressed – pages 126 – 129.
- Sitting and standing up – pages 129 – 132.
- Lifting, pulling, pushing and reaching – pages 132 – 135.
- Cooking, cleaning, outdoor activities (like shoveling snow) – pages 136 – 137.

Exercises for Building Strength and Endurance

Take a look at the pictures and descriptions of simple floor exercises for your back pain on pages 154 – 159.

Six Steps for Finding Time for Exercise

1. Write down your daily routine.
2. Find activities that can be combined with some type of exercise.
3. Find times when you are not busy.
4. Find activities you might be willing to give up for exercise.
5. Think about how you relax after a busy day.
6. Set aside a special time to exercise during the day.

Benefits of Stretching

- Stretching can reduce pain: This is probably the main reason people with back pain stretch regularly.
- Stretching prevents muscle strains and pulls: If you have good flexibility, you can more safely move through a wider range of motion.
- Flexibility helps you to keep a neutral position during your daily activities: Leg and hip flexibility will give your spine the most support against gravity and will give you the best protection from injury, tiredness, and pain.

Tips for Increasing Strength and Endurance

- Do the exercises at least two times a week.
- Use strength-training exercises before you do aerobic exercises.
- Warm up your muscles.
- Start by doing as many repetitions as you can, and add more as you get stronger.
- Breathe naturally and smoothly.
- Build endurance as well as strength.
- Use your trunk muscles to support your back and stay within your comfort zone when exercising.
- Stop exercising if you become significantly tired.
- Slowly increase the exercise time and intensity.
- Challenge yourself to do more.

© Boston Medical Center

Education Newsletter Volume 4

Everyday Insights for Better Living

How to Re-Establish a Normal Sleeping Pattern

- Set a regular waking time and stick with it.
- Set a time for going to bed.
- Try not to vary your schedule from day to day.
- Avoid daytime naps.
- Re-evaluate how much sleep your body needs.
- Sleep better by spending less time in bed.

How to Prevent Future Sleep Problems

- Avoid caffeine.
- Avoid alcohol.
- Exercise regularly.
- Take time to slow down as bedtime approaches.
- Do not lie awake at night.
- Find another time for worrying and planning.
- Do not overuse sleeping pills.

How to Avoid Problems in Your Relationships

- Focus on others: instead of your back.
- Pay attention: to the ways your back problem causes distress for others.
- Notice any sacrifices: others have made for you and acknowledge them.
- Ask about the thoughts and feelings: of your family and friends.
- Remain as active and self-reliant as possible.
- Interact with others: just as you would if your back was not a problem.
- Avoid taking out your frustrations: on others.
- Tell others directly: if you occasionally need their help, or if you do not need their help, with a task that they offer to do for you.
- Do not withdraw from life: Maintain your normal social interactions.

Tips for Workers with Back Pain

- Use good body mechanics.
- Pace yourself.
- Know your limitations.
- Design your workstation to minimize the use of poor posture and body mechanics.
- Discuss any potential risk of injury with your boss.
- When flare-ups occur, try to keep working, even if it is at a modified job.
- If you're not well enough to go to work, maintain contact with your boss and coworkers.
- When you are looking for a new job, it is usually best to present yourself as healthy and capable, as long as you are sure you can perform the job.

The American Chronic Pain Association's Ten Steps for Dealing with Pain

1. Accept the Pain
2. Get Involved
3. Learn To Set Priorities
4. Set Realistic Goals
5. Know Your Basic Rights
6. Recognize Your Emotions
7. Learn To Relax
8. Exercise
9. See Total Picture
10. Reach Out

© Boston Medical Center
