# Supplementary material for: Yoga vs. physical therapy vs. education for chronic low back pain in predominantly minority populations: study protocol for a randomized controlled trial
Source: Trials. 2014 Feb 26;15:67. doi: 10.1186/1745-6215-15-67 (PMC3944007; doi:10.1186/1745-6215-15-67)
Supplement: Additional file 5 — Study Policies and Procedures Manual. Comprehensive manual for research staff for operationalizing different study activities, including recruitment, enrollment, treatment interventions, data collection, qualitative research, and adverse events. [file 1745-6215-15-67-S5.pdf]

Back to Health

A Study Comparing Yoga, Physical Therapy & Education for Chronic Low Back Pain

Policies and Procedures Manual

Version 3.0 October 21, 2013

Table of Contents

Introduction

Policies and Procedures Overview	1
Study Overview Flow Sheet	2
Yoga and Physical Therapy Sites	5

Recruitment, Consent, and Screening

Recruitment Flow Sheet	7
Comprehensive Recruitment Screening Procedures	8
Procedure for Posting Brochures and Flyers	19
Back to Health Recruitment Flyer	22
Back to Health Recruitment Brochure	23
Eligibility Screening Questionnaire	25
Challenging Situations Procedure	32
Information Session Meeting Procedure	33
Information Session Reminder Phone Call Script	36
Information Session Presentation and Script	37
Study Fact Sheet	55
Informed Consent Checklist	57
Monitoring Recruitment Progress	61

Yoga

Yoga Treatment Arm Flow Sheet	66
Yoga Randomization Script	67
Yoga Class General Reminder Script	68
Yoga Survey Reminder Call	69
Yoga Maintenance Randomization Script	70
Yoga Adverse Event Call Script	71
Yoga Home Practice Call Script	72
Weekly Home Practice Log	73
Weekly Attendance Reporting	74
Yoga Class Observation Forms	75

Physical Therapy

PT Treatment Arm Flow Sheet	85
PT Randomization Phone Call Script	86
PT Survey Reminder Call	87

Table of Contents

PT Maintenance Randomization Script	88
PT Adverse Event Call Script	89
PT Home Practice Call Script	90
Weekly Home Practice Log: Extension Exercises	91
Weekly Home Practice Log: Flexion Exercises	92
Weekly Home Practice Log: Stabilization Exercises	93
Weekly Attendance Reporting	96

Education

Education Treatment Arm Flow Sheet	98
Education Randomization Phone Call Script	99
Education Survey Reminder Script	100
Education Check-In Phone Call Script	101
Education Adverse Event Call Script	102
Health Education Assignment	103

Data Collection and Management

Data Collection Timeline	105
Staff Blinding and Responsibilities	104
General Data Collection Guidelines	107
Randomization Procedure	108
Survey Administration Procedures	110
Data Entry Procedure	114
Data Management Procedures	115
Verification of Eligibility Checklist	117
Survey Distribution Schedule	119
Procedure for Filing Paper Surveys	120

Qualitative

Qualitative Interview Request Script	124
Interview Guide for Participants	125

Adverse Events

Adverse Event Form	129
Adverse Event Procedures	131

Procedures and Policies Overview

The purpose of this manual is to serve as a guide through the various phases of this randomized controlled trial. The standardized script will help to ensure that research members who carry out this research study are utilizing the most effective and appropriate approaches in administering and facilitating the various aspects of the study.

Specifically, this manual provides scripts for recruitment and eligibility screenings, data collection and management, yoga, physical therapy, and education treatment arm materials, and qualitative data collection.

Study Overview Flow Sheet

Forms Needed

Stage	Forms Needed
**Recruitment**	• Posting of Brochures and Flyers<br>• Recruitment Activities in Excel Procedure
**Telephone In-Take**	• Procedure for Answering Phones<br>• Procedure for Voicemail and Email
**Telephone Eligibility Screening**	• Eligibility Screening Procedure<br>• Verbal Consent<br>• Eligibility Screening Questionnaire
**In-Person Info Session**	• Info Session Reminder Call<br>• Info Session Procedure<br>• Info Session Power Point Presentation<br>• Information Sheet
**In-Person Consent**	• Informed Consent Checklist<br>• Written Informed Consent<br>• Welcome Letter
**Eligibility Checklist**	• Eligibility Checklist Form
**Baseline Survey**	• Baseline Questionnaire<br>• Survey Administration Script<br>• Survey Administration DOs and DON'Ts
**Randomization**	• **Appropriate** Randomization Phone Call Script
**3 Week Education Check-In**	• Education Check-In Phone Call Script
**6 Week Data Collection**	• 6-week Questionnaire<br>• Survey Administration Script<br>• Survey Administration DOs and DON'Ts

Study Overview Flow Sheet

9 Week Education Check-In

- Education Check-In Phone Call Script

12 Week Data Collection

- 12-week Questionnaire
- Survey Administration Script
- Survey Administration DOs and DON'Ts

Maintenance Randomization

- **Appropriate** Maintenance Randomization Phone Call Script

17 Week Non-Structured Maintenance & Education Check-In

- **Appropriate** Home Practice Call Script
- Education Check-In Phone Call Script

22 Week Non-Structured Maintenance & Education Check-In

- **Appropriate** Home Practice Call Script
- Education Check-In Phone Call Script

26 Week Data Collection

- 26-week Questionnaire
- Survey Administration Script
- Survey Administration DOs and DON'Ts

27 Week Non-Structured Maintenance & Education Check-In

- **Appropriate** Home Practice Call Script
- Education Check-In Phone Call Script

32 Week Non-Structured Maintenance & Education Check-In

- **Appropriate** Home Practice Call Script
- Education Check-In Phone Call Script

Study Overview Flow Sheet

37 Week Non-Structured Maintenance & Education Check-In

- **Appropriate** Home Practice Call Script
- Education Check-In Phone Call Script

↓

40 Week Data Collection

- 40-week Questionnaire
- Survey Administration Script
- Survey Administration DOs and DON'Ts

↓

42 Week Non-Structured Maintenance & Education Check-In

- **Appropriate** Home Practice Call Script
- Education Check-In Phone Call Script

↓

47 Week Non-Structured Maintenance & Education Check-In

- **Appropriate** Home Practice Call Script
- Education Check-In Phone Call Script

↓

52 Week Data Collection

- 52-week Questionnaire
- Survey Administration Script
- Survey Administration DOs and DON'Ts

Back to Health
A Study Comparing Yoga, Physical Therapy & Education for Chronic Low Back Pain

Yoga and Physical Therapy Sites

Boston Medical Center (PT & Yoga)
1 Boston Medical Center Pl.
Boston, MA 02118

South Boston Community Health Center (Yoga)
409 West Broadway
Boston, MA 02127

Upham's Corner Health Center (Yoga)
415 Columbia Rd.
Dorchester, MA 02125

Dorchester House Multi-Service Center (PT & Yoga)
1353 Dorchester Ave.
Dorchester, MA 02122

Codman Square Health Center (Healthworks) (Yoga)
450 Washington Ave.
Dorchester, MA 02124

Greater Roslindale Medical Center (Yoga)
4199 Washington St.
Dorchester, MA 02130

The Lowney Building (PT)
1234 Hyde Park Ave.
Hyde Park, MA 02136

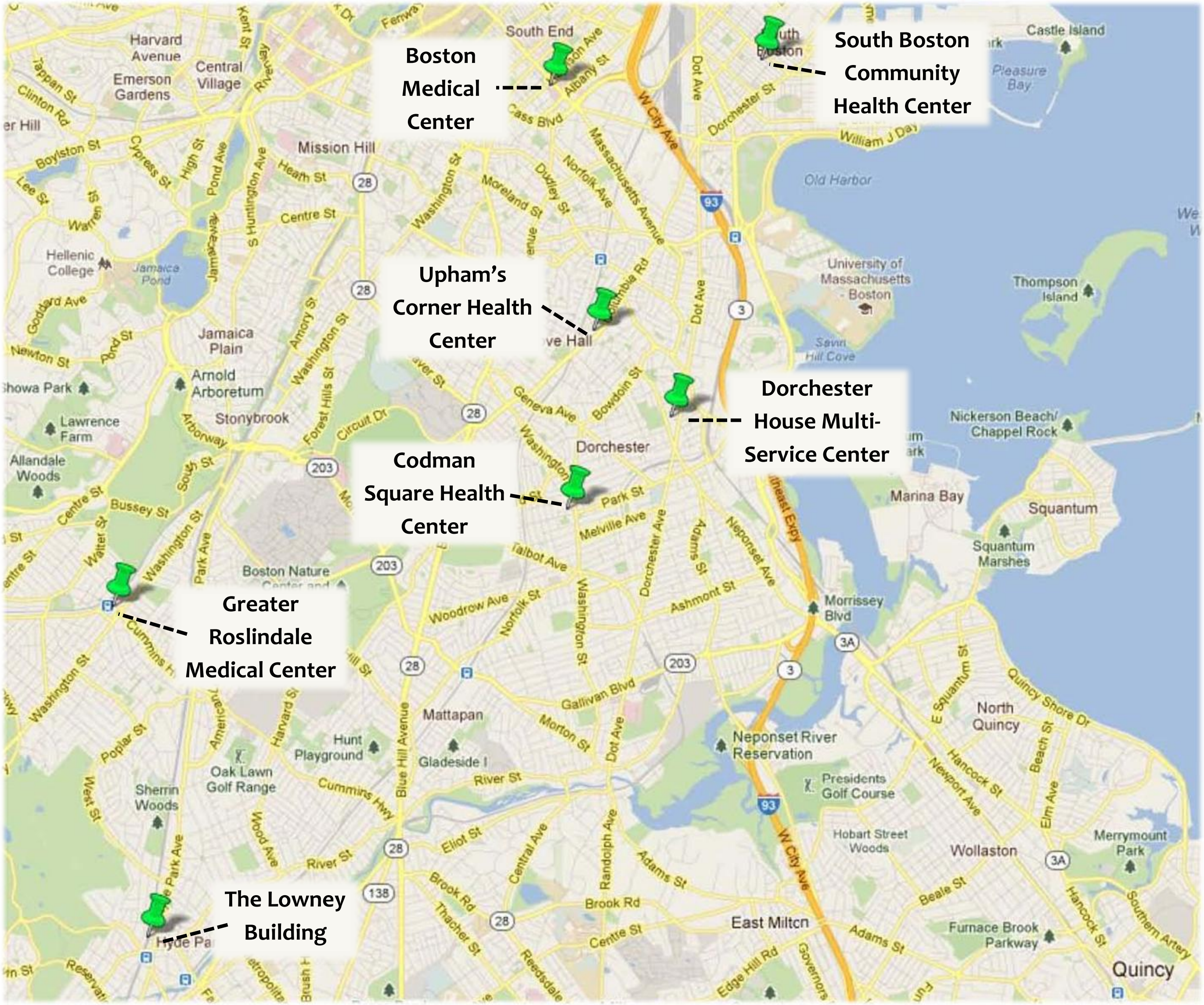

© Boston Medical Center
Program for Integrative Medicine & Health Care Disparities
Back to Health Trial

Back to Health

Recruitment, Consent, and Screening Procedures

Recruitment Flow Sheet

Forms Needed

Recruitment
- Posting of Brochures and Flyers
- Recruitment Activities in Excel Procedure

↓

Telephone In-Take
- Procedure for Answering Phones
- Procedure for Voicemail and Email

↓

Telephone Eligibility Screening
- Eligibility Screening Procedure
- Eligibility Screening Questionnaire
- Verbal Consent

↓

In-Person Info Session
- Info Session Reminder Call
- Info Session Procedure
- Info Session Power Point Presentation
- Information Sheet

↓

In-Person Consent
- Informed Consent Checklist
- Written Informed Consent
- Welcome Letter

↓

Baseline Survey
- Eligibility Verification Checklist
- Baseline Survey

↓

START OF STUDY

(See Data Collection Timeline)

CHECKING VOICEMAIL AND E-MAIL

General:

- The study team voicemail and email is checked at the beginning of *each* shift, as well as during the shift as seen fit.
- This information is then entered into the spreadsheet "B2H Telephone Screening Inquiry Log", giving the date, time, participant's name, and message.
  - Click on the "**Inquiry**" tab
  - Fill out the date, name, message type, contact information, and call details given through the e-mail or voicemail
  - Highlight the entire row by clicking the row letter
  - Fill background by clicking on the downward pointing arrow and selecting red
  - Save file
- This document is locked and saved.
- The voicemail is then deleted and the e-mail response is BCC'd to the entire recruitment team and marked as read.

Leaving a Voicemail

- Script:
  - *Hello, this is* [your name] *calling from Back to Health. Please call me back at [phone number] when you have a chance. Thanks!*
  - This does not include the participant's name so that their information is kept as confidential as possible.
- Fill out time and date of the call back.

DAILY SPREADSHEET PROCEDURE

At the beginning of shift:

1. Black out an entire row and type the date.
2. Check to see if anyone wants to be called back that day by reading notes of cases in progress (blue). Make a note of it and remind people coming in later in the day. Mark anyone who has been called three times with no response green.
3. Check to see if anyone has an Information Session the next day. If so, make sure to call them that day (see the Procedure for Info Session Reminder Call).
4. Assign yourself to people who you will be calling that day, making sure to coordinate with the other people working with you.
5. Check to see if you need to follow-up with anyone.
    a. Find tasks assigned to you on the main StudyTrax home page.
    b. Click on 'Task Search' on the left-hand panel and use the 'Filter' to locate tasks assigned to you.
    c. Set task to 'Complete' if a final decision has been made.
    d. Copy and Paste the **complete** follow-up log, including PI's notes, into the participant's 'Subject Overview' page.
        i. Enter Date and Signature.
    e. If applicable, click on 'Follow-up complete' in the Telephone Survey to pass the participant.

At the end of shift:

1. Send an email to the recruitment team with general notes about things that came up during the day (do not include names or personal information).

STEP 1: ANSWERING THE PHONE

Be ready by having the materials nearby or opened while by a phone:

- Study Trax Page, logged in
- B2H Telephone Screening Log
- Calendar (to schedule info session, if applicable)
- Blank Verbal Consent Form
- Pen and Sticky notes

© Boston Medical Center
Program for Integrative Medicine & Health Care Disparities
Back to Health Trial

Comprehensive Recruitment Screening Procedures

Script:

Hello, thanks for calling Back to Health, this is (name).

(Wait for response.)

Response:

- **Interested in participating in study:**

  Back to Health is a study that compares yoga, physical therapy, and education for lower back pain. We are currently looking for people with chronic lower back pain to be a part of the study, which will last for one year. Eligible participants will be randomly divided into one of three treatment groups doing either yoga, physical therapy, or education.

  Does this sound like something you may be interested in?

  If YES (INTERESTED):

  In order to find out if you can be in the study, we will first need to complete a questionnaire over the phone that lasts about 20-30 minutes. Is this a good time to do the questionnaire?

  If YES: *Great, I am just pulling it up,* one *moment please.*

  [SKIP to Telephone Screening Prescreening Questions (below)]

  If NO: *When would be a good time for you to complete the questionnaire?*

  [ACTION: Mark down time and date in "Best Time to Call Back" in **Spreadsheet** and highlight in yellow]

  [ACTION: Continue to Telephone Screening Prescreening Questions (below)]

  If NO (NOT INTERESTED):

  Okay, thank you for your call and let us know if you change your mind! Before you go, I just wanted to ask a couple of questions for our records if that is okay. [Pause for response]

  If YES: [SKIP to Telephone Screening Prescreening Questions (below)]

  If NO (try to avoid this): *That's okay, thank you for your time, have a nice day!* [HANG UP]

If speaks SPANISH and there is too much of a language barrier:

Lo siento, pero para matricular en este estudio, usted necesitaría hablar inglés bueno. Yo hablo un poquito español. ¡Gracias, que tenga un buen día!

"loh see-ehn-toh, pare-oh pah-rah mah-treek-oo-lar en ey-stay ey-stoo-dee-oh, oo-stehd ness-ess-ee-tahr-E-ah ab-blar eeng-glays bway-noh. Yo ab-bloh oon poh-kee-toh ess-pah- Ñ-ole. Grah-see-ahs, kay tayng-gah oon bwen dee-ah!"

STEP 2: TELEPHONE PRESCREENING QUESTIONS:

1. *Can I have your first and last name please?*
   [ACTION: Mark Message Type]
2. *What is the best number to reach you at?*
3. *Do you have an e-mail address that we can reach you at?*
   [ACTION: Enter Call Details]
   [ACTION: Enter your name under 'Responsibility']
   [ACTION: Enter any notes]
4. *How old are you?*
   a. If 18-64→eligible
   b. If not 18-64→ineligible (wait until step 8 to end call)
5. *What is your zip code?*
   a. **If OUTSIDE OF BOSTON:** *If you are placed in the physical therapy or yoga group in our study, you will have to attend sessions each week at [health centers]. This may require you to travel. Does that sound like something you would be able to do?*
      If it is a problem: *Would you like us to put your name down for the next cohort, which will be held at [health center]?*
      - **YES, I would like to be added to the waitlist:** *When would be a good time to call you back?*
        i. [ACTION: Mark in "Best Time to Call Back" in **Spreadsheet** and highlight in yellow]
        ii. *Great, we will speak with you later on. Thank you for your call and have a nice day!*

iii. [HANG UP]

iv. [ACTION: Mark in **Waitlist for Screening by Zip Code**]

- **NO, I would not like to be added to the waitlist:** *Okay, sorry about that, I hope you have a nice day, and feel free to give us a call if you change your mind. Our phone number is [phone number]. Thanks, goodbye.* [HANG UP]

If it is NOT a problem: *Great, I'm glad you will be able to travel there.*

6. *How did you hear about the study?*

If INELIGIBLE:

- Due to age: *I am sorry, but you are not eligible for the study. Thank you for your interest and have a nice day!* [HANG UP]

  If "WHY?": *You must be between the ages of 18 and 64 to participate.*

STEP 3: PASS PRESCREENING

If ELIGIBLE:

UNAVAILABLE TO DO SCREENING NOW: *Thank you, we will speak with you on* [day] *at* [time] *have a nice day!* [HANG UP]

STEP 4: VERBAL CONSENT

AVAILABLE TO DO SCREENING NOW: *Okay, we are going to start the questionnaire, one moment please. I am just pulling it up.*

ACTIONS:

1. Find your next SID # ["SID" tab in **Spreadsheet** and highlight in red]
2. Click and enter "New Subject" On Study Trax.
3. SKIP to Step 3: INTITIAL ELIGIBILITY SCREENING PROCEDURE

Answering Questions

Answer question to the best of your ability. Below are some potential questions.

- **What is CLBP?**

  Chronic low back pain is non-specific low back pain lasting for twelve weeks or longer.

- **How do I know if I am eligible?**

To be eligible for this study, you must be between 18-64 years old; have current non-specific low back pain persisting ≥12 weeks which will be determined through a series of questions; understand English well enough to follow treatment instructions and answer survey questions; be willing to list comprehensive contact information for at least one friend, family member, or work colleague who will always know how to contact the participant.

- **Where will I take yoga or physical therapy classes if I am eligible?**

  Yoga classes will be held at [locations]; you will be assigned to one of these locations based on your personal preference. Physical therapy classes will be held at [locations].

- **What is the time commitment?**

  You can expect to go in to one of the community health centers once to twice per week. We have both weekday and weekend times available.

If you do not know the answer to the question:

1. *I am sorry, but I am not sure. I will check with my supervisor. Do you mind if I put you on hold?*
   [ACTION: Put on hold and call recruitment coordinator]
   [ACTION: Answer question]

2. **If question still cannot be answered:** *I am sorry, but I will need to check with the Principal Investigator and get back to you as soon as possible. What phone number is best to reach you at?*
   [ACTION: mark phone number in "Best Time to Call Back" in **Spreadsheet**]
   [SKIP to Telephone Screening Prescreening Questions (above)]

3. *Thank you, please give us a call if you have any other questions. Have a nice day!*
   [HANG UP]

Remember not to leave any identifiable information out
(notes should be shredded as soon as entered onto computer).

STEP 4: VERBAL CONSENT (CONTINUED) AND ELIGIBILITY SCREENING

PART ONE: ON THE PHONE

1. Find your next SID # ["SID " tab in **Spreadsheet** and highlight in red]
2. Go to StudyTrax Home and click enter "New Subject"

3. Select the Cohort number for the new participant.
4. Start the Questionnaire, beginning with RCT Contact Info.
   - Follow prompts and scripts on screen.
   - If they answer "don't know," ask them politely to take some time to think about it. If they truly don't know, then input DK.
   - "DK/REF" responses are "-88" when numerical values are needed.
   - "NA" responses are "-99" when numerical values are needed.
   - If participant answers "yes" to weight loss question, ask "*How much weight have you lost over how much time?*" Use common sense to determine if it is cause for concern. If so, have PI follow-up.
   - Make sure that blanks are marked as "NA" before moving on to next section.
   - Save throughout screening. It may take a few moments to save and refresh, so you can say, "*One moment as I load the page.*"
   - When first section is complete and saved, it will appear as green in the side bar.

STEP 5: ELIGIBILITY SCREENING**

Next, move on to the screening questionnaire, RCT Telephone Eligibility Screening, by clicking on it in the side bar.

If ELIGIBLE:

1. [ACTION: Go to the Team Calendar and look for the upcoming info session times]
2. *The next step will be to come in for an information session, where we will go over a consent form and answer any questions you may have. This will take about an hour. Is there a certain time of day or day of the week that works best for you?*
3. [ACTION: Compare their preferences to the available slots in the Calendar. If there are no info sessions that they can come to, put them on a waiting list.]

STEP 6: SCHEDULE IN-PERSON*

If ABLE TO SCHEDULE TIME/DATE:

Great! Thank you for your patience, we will see you on [date] *at* [time]*. We will be meeting at* [name of health center]*, located at:*

Do you have any questions at this time?

© Boston Medical Center

If YES: [Answer to best of your ability]

If DON'T KNOW ANSWER*: I am sorry, but I am not sure. I will check with my supervisor. Do you mind if I put you on hold?*

a. [ACTION: Put on hold and call recruitment coordinator]
b. [ACTION: Answer question]
c. **If question still cannot be answered:** *I am sorry, but I will need to check with the Principal Investigator and get back to you as soon as possible. What phone number is best to reach you at?*
   i. [ACTION: Mark "Best Time to Call Back" and make a note of question and highlight participant in the **Telephone Screening Inquiry Spreadsheet**]

If NO: *Thank you, please give us a call if you have any questions. Our number is [phone number]. Have a nice day!* [HANG UP]

[SKIP to Part Two: After Hanging Up]

If UNABLE TO SCHEDULE TIME/DATE:

I'm sorry that none of those times work for you. Are you generally busy at that time each week? (Pause)

If YES: *Unfortunately, these are the times that we will be holding the yoga classes on a regular basis during the study. If these times do not work for you, it may be difficult to be in the study at all. If you think that this could be a problem, we unfortunately cannot move onto the next step in the process.* (Pause) *Thank you for your time in completing the screening, give us a call if your availability changes. Thanks! Goodbye.* [HANG UP]

If NO: *The reason I am asking is because these are the times that we will be holding the yoga classes on a regular basis during the study, so we want to make sure that you are available during at least one of these times. Would you be able to come in during any of these times next week?*

If YES: [SKIP to "If Able to Schedule Time/Date"]

If NO: *That's okay. We can instead meet you at [health center] at another time. What times and days would be better for you?* (Pause)
[ACTION: Mark SID number and "one-on-one info session" in appropriate time slot on Calendar]

Perfect! I will mark you down for (day of week, date) *at* (time), *just one moment, please.*

We will meet at [meeting location details]. *Feel free to give us a call if at you have any questions. Our number is* [phone number]. *Thanks for your patience, we will see you then!* [HANG UP]

[ACTION: Send an e-mail to the team-member(s) who will be in at that time.]

[SKIP to Part Two: After Hanging Up, especially 3b]

If INELIGIBLE:

1. Follow procedures as prompted on screen.
2. If participant asks why he/she is ineligible: *I'm sorry, I am not trained to interpret the results. Thank you for your time, I hope you have a nice day.* (Pause)
3. If participant becomes upset, refer to "Challenging People" procedure.

PART TWO: AFTER HANGING UP

STEP 7: AFTER PHONE CALL

1. Verbal Consent Form:
   a. Fill out last page, making sure to sign, print your name, and write the date.
   b. Write down the SID number on top right hand corner.
   c. Find the corresponding SID folder in the Recruitment file cabinet, check off "Signed Verbal Consent" and date, and return folder to locked file cabinet. (This must be locked at the end of the day.)
      - There are 3 forms that will be included in the folders:
         1. Signed Verbal Consent Form
         2. Informed Consent Progress Report
         3. Eligibility Verification Form
      - When each of these forms is completed, the label on the front of the folder will be checked off and dated and the forms will be stored inside.
      - These forms will be filed immediately once completed.
      - The filing cabinet will be locked at all times, unless it is currently in use.
2. In Study Trax:
   a. If ineligible write down ALL ineligibility reasons (underneath screening status)

b. Fill in all missing answers with "NA"
c. Edit Subject→ check "Needs to be Followed up" if needs PI follow-up.
d. Check to make sure all questions were answered and that each section is marked as green.

3. * Calendar:

a. If scheduled time for info session, record on the Calendar by adding the participant's SID number to the description (under "Edit Event") in the appropriate Info Session time slot. (Info Session>Edit Event>Description). Update the title of the event by adding the number of people signed up for the info session in parentheses.
b. Create an event if meeting one-on-one at BMC (if meeting with the participant at another time was not possible). Title it "One-on-One Info Session" and write his/her SID number in the description. Send an e-mail to the other team member(s) who will be in that day so that he/she can meet with the participant (remember that they will meet at the ER front desk on that day).
c. Make sure the correct date is marked.

4. **If follow-up from PI needed:

a. Locate 'Tasks' on the left-hand panel of the Subject Overview page.
b. Click the green 'Plus' button
   i. Set Priority, Status, and Due Date (~1 week) appropriately
   ii. Set 'Name' to *Eligibility Follow-up*
   iii. Assign to PI
   iv. Include a succinct description on what needs to be followed up on
   v. **Date and initial your description**

NOTE: Names and phone numbers should NOT be included in Tasks

c. Save and Return
d. Return to the 'Subject Overview' page
   i. Click on 'Edit Subject' and enter Date and Signature
e. Once PI has completed the follow-up, he will assign the task back to you.
f. Record this information in the Recruitment Document, under the Follow Up tab.

Remember not to leave any identifiable information out

(notes should be shredded as soon as entered onto computer

and file cabinets should be locked at the end of the day).

FILING FOLDERS

- There are 3 forms that will be included in the folders:
    1. Signed Verbal Consent Form
    2. Informed Consent Progress Report
    3. Eligibility Verification Form
- When each of these forms is completed, the label on the front of the folder will be checked off and dated and the forms will be stored inside.
- These forms will immediately be filed once completed.
- The filing cabinet will be locked at all times, unless it is currently in use.

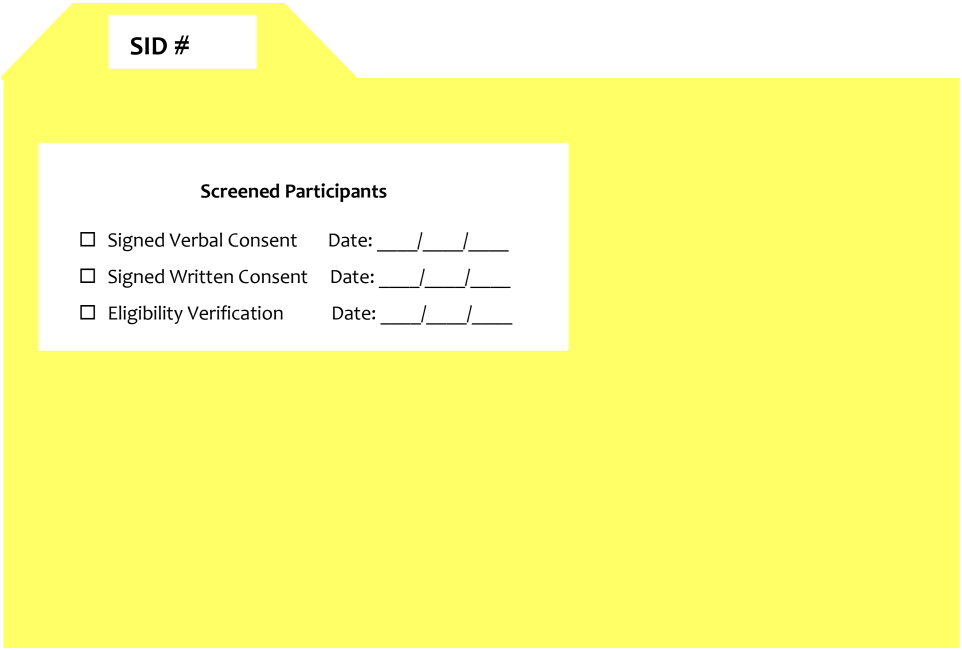

© Boston Medical Center

Procedure for Posting Brochures and Flyers

Materials

- Push pins
- Tape
- Recruitment Box
    - Weekly count sheet (1)
    - Pen (2)
    - Full Flyers (40)
    - Mini-flyers (240)
    - Pull tab flyers (40)
    - Brochures (240)
- Business Cards
- Team T-shirt

Posting Flyers in the Community

- Types of Flyers:
    - Full Flyer
    - Mini Flyer
    - Pull-Tab Flyer
    - Brochure
- Flyers are placed on either the windows, bulletin boards, or desks of the following places in the neighborhoods of the health centers where the yoga classes are taking place in order to recruit participants:
    - Community Health Centers
    - General Community Centers
    - Churches
    - Barbershops
    - Hair Salons
    - Nail Salons
    - Grocery Stores
    - Small Restaurants and Fast Food Places
    - Laundromats
    - Pharmacies

Procedure for Posting Brochures and Flyers

Entering Establishments

Wear the team T-shirt and gear with appropriate business casual clothing and minimal jewelry or flashy accessories. Approach an employee in a friendly manner, being patient if they are busy. Take note of any places within the establishment where a flyer or brochure could be placed or posted.

Script for Flyer Placement

Hello, I am part of the Recruitment Team from a study that we are doing at Boston Medical Center to compare the yoga, physical therapy, and education for low back pain. We are currently looking for participants in the community and are wondering if it would be possible to post flyers ________________ (on the bulletin board, in the store window, on the table, etc.).

If yes:

Thank you, where would you prefer? (wait for response) *Would you mind if we come back each week to replenish the flyer?* (wait for response) *Great, here is our business card* [ACTION: hand business card]*. We will see you* **[day],** *have a nice day!*

If no:

That's okay, we understand. Thank you, have a nice day!

Maintenance of Flyers

- Each week box contents will be counted and entered into B2H Recruitment Count Sheet. This will then be restocked.
- Each week the flyers and brochure supply will be checked at each location and replaced or replenished when necessary.
- At each location:
  - Find if a flyer is posted
  - If the full flyer is posted or the pull-tab flyer has most of its tabs → do not post another or replace

- If pull-tab flyers are posted but all tabs are pulled or most have been pulled→ replace the full flyer with a pull-tab flyer
- Locations with brochures and mini-flyers, replenish the stock or holders
- On the count sheet, record the number of flyers and brochures left at each location

- Find and locate potential new locations to post flyers and brochures in the areas surrounding the community health centers
- Record new location on the count sheet by providing the name of the establishment and address

After Recruitment Activities

- Open the Excel file "B2H Recruitment Activities"
- Select the "Materials" worksheet
- Fill in the cells on "Materials" worksheet with the values on the corresponding cells on the count sheet
- Add new locations to the worksheet and file by following the "B2H Recruitment Activities Excel Sheet Procedure"
- Save the "B2H Recruitment Activities" file
- Print to replace or return the current count sheet for the following weeks recruitment activities trip

DO YOU SUFFER FROM CHRONIC LOW BACK PAIN?

Researchers at Boston Medical Center are conducting a research study to compare three strategies to manage chronic low back pain

You may be able to receive yoga, physical therapy, or education at no cost

Adults of all backgrounds from who have had low back pain for at least 12 weeks are encouraged to call

For more information, please call (617)414-6211 or email backtohealthstudy@bmc.org

Compensation will be provided

BUMC/BMC Institutional Review Board
IRB NUMBER: H-31455
IRB APPROVAL DATE: 02/13/2013
IRB EXPIRATION DATE: 02/12/2014

Version 2.0 Approved from 2/13/2013 to 2/12/2014

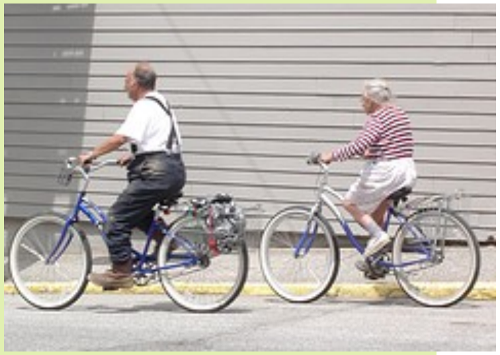

Yoga, Physical Therapy, and Education May Help Reduce Low Back Pain!

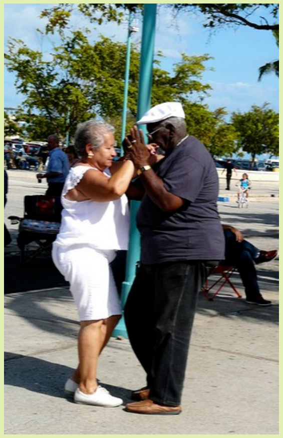

For more information, please call (617) 414 - 6211 or email backtohealthstudy@bmc.org

Yoga, Physical Therapy, and Education May Help Relieve Your Low Back Pain

For more information, please call (617) 414—6211 or

email backtohealthstudy@bmc.org

Adults of all backgrounds who have had low back pain for at least 12 weeks are encouraged to call.

BOSTON MEDICAL CENTER

EXCEPTIONAL CARE. WITHOUT EXCEPTION.

Back to Health

A Study Comparing Yoga, Physical Therapy & Education for Chronic Low Back Pain

Back to Health

A Study Comparing Yoga, Physical Therapy & Education for Chronic Low Back Pain

For More Information Call (617) 414 – 6211

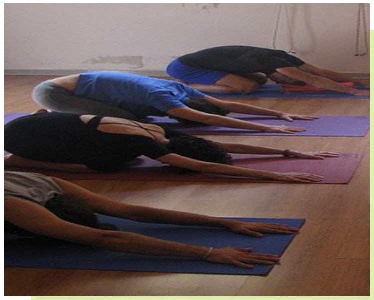

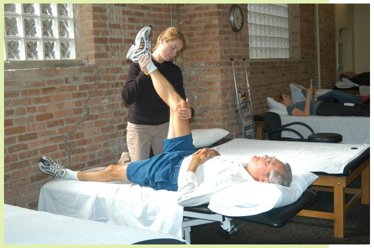

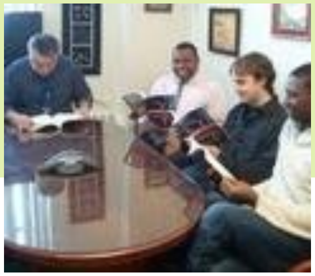

Healing your back ....

Do you suffer from low back pain?

Low back pain is a common cause of suffering for many patients. Medications often do not provide adequate relief. Yoga, physical therapy, and education have shown promise as a treatment for chronic low back pain.

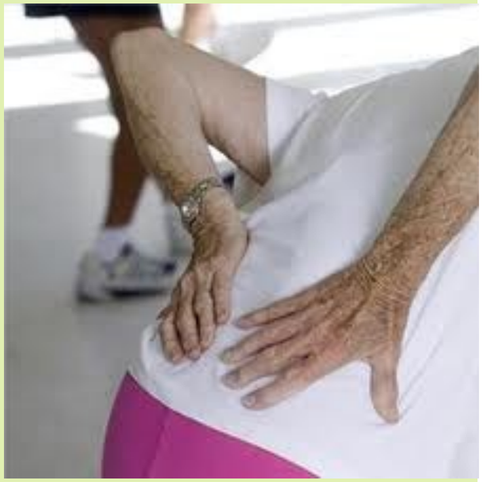

If you suffer from low back pain...

You may be able to receive treatment at no cost in a research study comparing yoga, physical therapy, and education for low back pain management.

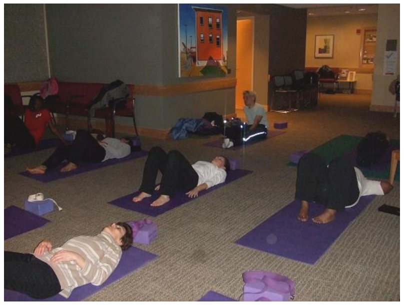

Compensation will be provided for participating.

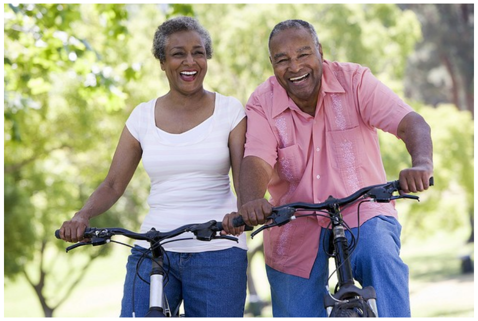

What is yoga?

Yoga is a safe, gentle, and soothing exercise that involves stretching, breathing, and relaxation Many appreciate the benefits of yoga and find that it also reduces stress and lifts mood.

What is physical therapy?

Physical therapy is a safe treatment that involves stretching and exercises that focus on building muscle strength. Many experience increased mobility and improved flexibility due to physical therapy.

What is education?

Education is a way for people to learn different strategies to improve their back pain. Many feel that educational materials help them better manage their pain.

BUMC/BMC Institutional Review Board
IRB NUMBER: H-31455
IRB APPROVAL DATE: 02/13/2013
IRB EXPIRATION DATE: 02/12/2014

Eligibility Screening Questionnaire

Checklist:

1. Are you between the ages 18 and 64?
   ☐ Yes ☐ No

2. What is your zip code? ___________________

3. How did you hear about the study?
   ☐ Doctor
   Please specify where: ___________________
   ☐ Community Health Center Staff
   Please specify where: ___________________
   ☐ Flyer or Brochure
   Please specify where: ___________________
   ☐ Letter in the mail
   ☐ Friend or Family Member
   Please specify where: ___________________
   ☐ Newspaper
   Please specify where: ___________________
   ☐ Radio
   Please specify where: ___________________
   ☐ Other
   Please specify where: ___________________

Contact Information

Name: ____

DOB: ________ /_________ /_________

Street: ____ Apt#: _____________

City: ___________________________________

State: ___________________________ Zip: ______________

Telephone

W. ________________________ H: ________________________ C: ________________________

Email: ____

© Boston Medical Center

Do you receive any of your health care at Boston Medical Center or an affiliated community health center (e.g. Dorchester House Multi-service Center, Codman Square Health Center, Upham's Corner Community Health Center, Greater Roslindale Medical & Dental Center, South Boston Community Health Center)?

☐ Yes ☐ No ☐ Don't know/Refuse

Which site?

☐ Dorchester House Multi-service Center
☐ Codman Square Health Center
☐ Upham's Corner Community Health Center
☐ Mattapan Community Health Center
☐ Greater Roslindale Medical & Dental Center
☐ Boston Medical Center
☐ Other—Please specify where: ______________________

OK. Now I am going to need to get your verbal consent before we continue with the eligibility screening survey. I will tell you information about this study, your participation in this survey, and your rights. Please feel free to ask me questions at any time.

—Verbal Consent—

Is it OK with you now if I go ahead and ask you now a number of questions to determine if you are eligible for the study?

☐ Yes ☐ No

Now we will begin the survey. We would like to better understand how well you are able to do your usual activities and how you rate your own health. This is not a test and there are no right or wrong answers. Choose the response that best represents the way you feel. Please answer each question as best as you can. I am happy to repeat a question at anytime. Please remember that your answers are confidential. OK, let's begin. I will now ask you a number of questions.

1. In order to be eligible for this research study, you must be able to understand spoken and written English. Do you think this will be a problem for you?
   ☐ Yes ☐ No

2. What is your age?
   ☐ Under 18 ☐ 18 to 64 ☐ 65 or greater

3. In the past 3 months, have you had a period of two weeks or longer during which you had **no** low back pain?
   ☐ Yes ☐ No ☐ Don't know/Refuse

4. In the past week how often have you had low back pain?
   ☐ Not at all
   ☐ Less than half the time
   ☐ Half the time
   ☐ More than half the time
   ☐ All the time
   ☐ Don't know/Refuse

5. Pick a number between 0 and 10 that best describes your low back pain in the last 7 days, where 0 is no pain and 10 is the worst possible pain. _______

6. In the next 12 months, are you planning to move out of the area or have a significant change in your schedule which would make it impossible to participate in the study?
   ☐ Yes ☐ No ☐ Don't know/Refuse

7. Have you ever been diagnosed by a doctor or other health professional with cervical radiculopathy, herniated disk, slipped disc in your neck, or a pinched nerve in the neck?
   ☐ Yes ☐ No ☐ Don't know/Refuse

Sciatica

8. In the past month, have you had numbness or tingling in the leg, foot, or groin?
   ☐ Yes ☐ No ☐ Don't know/Refuse

9. In the past month, have you had pain that travels down your leg below the knee?
   ☐ Yes ☐ No ☐ Don't know/Refuse

Spinal Stenosis

10. What is your age?
☐ Under 60 ☐ 60-70 years old ☐ Over 70 years old ☐ Don't know/Refuse

11. How long have you had your symptoms?
☐ Less than 6 months
☐ 6 months or more
☐ Don't know/Refuse

12. Does your symptom improve when you bend forward?
☐ Yes
☐ No
☐ Don't know/Refuse

13. Does your symptom improve when you bend backward?
☐ Yes
☐ No
☐ Don't know/Refuse

14. Does your symptom occur while you stand up?
☐ Yes
☐ No
☐ Don't know/Refuse

15. If your symptom occurs while walking, does it improve by resting?
☐ Yes
☐ No
☐ Don't know/Refuse

16. Do you have urinary incontinence? (trouble controlling your bladder)
☐ Yes
☐ No
☐ Don't know/Refuse

Sciatica

17. Does the pain in your leg sometimes get worse with coughing or sneezing?
☐ Yes ☐ No ☐ Don't know/Refuse

18. Which is worse, your back pain or your leg pain?
☐ Leg pain is worse
☐ Leg pain and back pain are the same
☐ Back pain is worse
☐ Don't know/refuse

19. Have you practiced yoga in the past 6 months?
☐ Yes ☐ No ☐ Don't know/Refuse ☐ Participation not significant

20. Have you visited a physical therapist in the past 6 months? (for your back)
☐ Yes ☐ No ☐ Don't know/Refuse

21. Have you read The Back Pain Helpbook or the Back Book in the past 6 months?
☐ Yes ☐ No ☐ Don't know/Refuse

22. Did you participate in the Yoga Dosing Study (YLBP2) or the Physical Therapy Pilot?
☐ Yes ☐ No ☐ Don't know/Refuse

23. Have you ever had back surgery?
☐ Yes ☐ No ☐ Don't know/Refuse

24. Have you ever fractured one or more of the vertebrae, or bones, in your spine?
☐ Yes ☐ No ☐ Don't know/Refuse

25. Have you recently lost a lot of weight for unknown reasons, that is, not as a result of a deliberate diet?
☐ Yes ☐ No ☐ Don't know/Refuse

26. Have you ever been diagnosed by a doctor or other health professional with severe or progressive **scoliosis**, **spinal stenosis**, s**pondylolisthesis**, or **ankylosing spondylitis**?
☐ Yes ☐ No ☐ Don't know/Refuse

27. Have you ever been diagnosed by a doctor or other health professional with **rheumatoid arthritis**?
☐ Yes ☐ No ☐ Don't know/Refuse

27A. **IF YES to 27**, Do you see or have you been referred to a rheumatologist or arthritis specialist?
☐ Yes ☐ No ☐ Don't know/Refuse

27B. **IF YES to 27**, Have you ever been prescribed any of the following medications?
☐ Methotrexate
☐ Prednisone
☐ Sulfasalazine (Brand Name →Azulfidine)
☐ Leflunomide (Brand Name→Arava)
☐ Etanercept (Brand Name→Enbrel)
☐ Adalimumab (Brand Name→Humira)

☐ Hydroxychloroquine (Brand Name→Plaquenil)

28. Have you EVER been diagnosed by a doctor or other health professional with **hepatitis**?
☐ Yes ☐ No ☐ Don't know/Refuse

28A**. IF YES to 28**, are you currently receiving treatment for this condition or planning to receive treatment in the next 12 months?
☐ Yes ☐ No ☐ Don't know/Refuse

29. In the next 12 months, are you planning to see any of the following health care providers for your back problem?
☐ MEDICAL SPECIALIST, FOR EXAMPLE, A RHEUMATOLOGIST OR NEUROLOGIST
☐ SURGEON, FOR EXAMPLE, AN ORTHOPEDIC SURGEON OR NEUROSURGEON
☐ PHYSICAL THERAPIST
☐ CHIROPRACTOR
☐ OSTEOPATH
☐ MASSAGE THERAPIST OR OTHER BODYWORKER
☐ ACUPUNCTURIST
☐ OTHER, SPECIFY ______________________________
☐ NONE
☐ Don't know/Refuse
☐ More than 1 specialist

29A. **IF YES to 29**, Do you have an appointment already scheduled?
☐ Yes ☐ No ☐ Don't know/Refuse

29B. **If YES to 29A**, are you willing to postpone it until the end of the study?
☐ Yes ☐ No ☐ Don't know/Refuse

30. Do you use a wheelchair?
☐ Yes ☐ No ☐ Don't know/Refuse

31. Are you currently involved in a lawsuit or are you applying for compensation of any kind for your back problem? By compensation, I mean for example, personal injury insurance, worker's compensation, social security disability insurance, or private disability insurance?
☐ Yes ☐ No ☐ Don't know/Refuse

32. Do you have severe vision or hearing problems?
☐ Yes ☐ No ☐ Don't know/Refuse

33. Do you have personal beliefs that prohibit practicing yoga, physical therapy, or education methods in this study?
    ☐ Yes ☐ No ☐ Don't know/Refuse

Thank you for answering our questions. You may be eligible for the research study. We would like to arrange a time to meet to make sure you are definitely eligible, receive a detailed explanation of the research study, have an opportunity to have any questions you may have answered, and possibly enrolled.

Would you like to arrange a time? ☐ Yes ☐ No

Intake appointment time
Date: ____________________ **Time:** ________________________

I'd like to gather some more information about how best to contact you:

Do you have access to internet at home? ☐ Yes ☐ No

Would you like to receive study information by email? ☐ Yes ☐ No

Would you like to receive study information by text message? ☐ Yes ☐ No

Can we contact you about similar research opportunities in the future? ☐ Yes ☐ No

Contact Name 1: ___________________________

Relationship to patient:___________________________

Address:____

Home tel: (_____)_____-__________ Work tel: (_____) ______-___________

Cell tel: (_____)_____-___________ Email:_____________________________

Contact Name 2: ___________________________

Relationship to patient:___________________________

Address:____

Home tel: (_____)_____-__________ Work tel: (_____) ______-___________

Cell tel: (_____)_____-___________ Email:_____________________________

Examples of Challenging Situations

1. Someone who is challenging:
   - Defensive
   - Accusatory
   - Suspicious
   - Rude
   - Memory issue
   - Upset from a mistake made by us (whether or not it was our fault)
   - Condescending
   - Odd from the beginning
   - Feeling uncomfortable
   - Sounds Intoxicated
   - LOTS of questions (not due to language barrier)

2. If the individual MAY meet any of these criteria:

   SAY: "It sounds like you have a lot of questions; I think that my supervisor can better help you…"

   "I am transferring you to her now." Put them on hold, call recruitment coordinator and explain the situation and she will take it from there.

What Recruitment Coordinator will do:
• Offer to send more info in a packet (to create some space and give them time)<br>• Speak more extensively to assess concerns and eligibility<br>• Refer participant to PI

3. How to Record this:
   a. **Study Trax**: Write "CP" in Study Trax under Edit Subject, along with a short 2-word explanation (using words listed above). Make sure to explain the situation to the person who does their info session.
   b. **Recruitment Doc**: Write "CP" in "Info Session Tab" under Notes

4. Report to Team at upcoming team meeting

Information Session
Meeting Procedure

The information session is a chance for the participant to meet with a member of the research team and learn more about the study. At this time, the participant may voice any questions or concerns and choose whether or not they will enroll in the study.

Materials

- Power Point Presentation
- Information Packets
- Snacks and Refreshments
- Laptop
- Information Session Lockbox and Gift card(s)

Before Information Session

- Find an empty room to hold the session.
- Set up and prepare for presentation.
- Have information packet, snack, refreshment, and gift card ready.
- Check the "notes section" in the spreadsheet to see if another staff member left an important reminder or note.

Information Session: Introduction

- Open the information session by introducing yourself and the study.
- The presenter may follow the script below, which is a suggested introduction to open the session.

  "Hello, and welcome to the Back to Health information session. My name is [name], and I will be conducting the information session today. Today's information session will contain a presentation detailing the study, a question and answer portion, and an explanation of the informed consent forms."

Power Point Presentation

- The session leader may lead into the Power Point session with the following script.

  "To follow along with the Power Point presentation, you can find the presentation slides in your information packets that you received when entering the room. If there are any questions during the presentation, please do not hesitate to ask."

- Proceed with the presentation by reading the slides and elaborating on the information. Answer any questions the audience may have during the presentation.

Question & Answer Session

The information meeting leaders answer questions about the study, general study design, and concerns the eligible participants may have. Some potential questions are:

- *What are the chances of being in the* [TREATMENT GROUP]?
  - You have a 40% chance of being in the Yoga group, a 40% chance of being in the physical Therapy group, and a 20% chance of being in the education group.
- *Can I choose or specify which treatment group I want to be in?*
  - No one on the study team can influence which treatment group a study participant will be placed. A computer will randomly assign each participant to a treatment group. Random meaning "by chance" like rolling a dice.
- *How difficult/intense are the yoga classes?*
  - All yoga classes are beginner/introduction level yoga classes.
- *Can I participate in the other forms of treatment outside of the study while continuing with my assigned treatment?*
  - When a person voluntarily enrolls in the study, we wish for individuals to stay with their assigned treatment group and do not participate in the other types of treatments outside of the study. For example, if a person is assigned to education and stays with the assigned treatment, but outside of the study attends a weekly yoga classes in the neighborhood.
- *Can I drop-out of the study once I enroll?*
  - Yes you may drop out of the study at any time during your enrollment. Your participation is voluntary, so you may step away from the study at any time.
- *How will I receive the stipend?*
  - After completing the survey at 6 weeks, 12 weeks, 26 weeks, 40 weeks, and 52 weeks. The amounts that will be given at each survey are not finalized, but the total amount received will be $400.
- *How will the study staff keep the personal/identifiable information confidential?*
  - All information on computers will be password protected. Information on paper will be kept in locked filing cabinets in secure rooms.
- *How will this study affect my insurance?*
  - Participating in the study will not affect your insurance.

- *Can I switch out of my assigned treatment group to another one that I prefer?*
    - Once enrolled in the study, you cannot switch or ask to switch into a different treatment group. If your participation is dependent on your assigned treatment group, then I would suggest you do not participate in the study.

Introduction to Consent Session

- If those in attendance do not wish to participate in the study, inform them that they may leave the information session at this time. The following script may be used.

  "In the next portion of the information session, we will discuss and review the informed consent. If you do not wish to participate in the study, thank you for your time and you may leave at this time."

- The remaining attendees read the written informed consent form.
- Answer any questions they may have about the study, and clarify any points of uncertainty.

One-on-one Consent Session

- Review the written consent form with the individual.
- Review the general information of the study, the treatment groups, risk & discomforts, benefits, confidentiality, and rights as a participant by following the "Informed Consent Checklist" form.
- Help clarify any unclear points and points of misunderstanding.
- If the person agrees to enroll in the study → have participant SIGN and DATE both consent forms.
- Upon receiving the SIGNED and DATED consent form → the team member must SIGN and DATE the consent forms as well.
- Welcome the participant into the study. You may use the following script.

  "Congratulations! You are now fully enrolled in Back to Health Study. We have for you a letter that officially welcomes you to our study [hand participant envelope] and a small token of our appreciation [hand participant mug]. Do you have any questions for me?"

  "Thanks again and we're excited that you're going to be a part of our study. Have a wonderful day!"

© Boston Medical Center

Information Session
Reminder Phone Call Script

Reminder Phone Call for Informed Consent Information Session

- Each participant will be called within 24 hours before their information session with the staff.
- The staff will follow the specified script and leave a message with someone or leave a voicemail if the participant is not available at the moment.
- This will be entered onto the **Telephone Screening Inquiry** spreadsheet**.**

Reminder for Information Session

Hello, this is (your name) with Back to Health. Is (participant's name) available?

If YES: *Hello (participant's name). This is a reminder from the Back to Health study at Boston Medical Center that you have a meeting scheduled on* **[day of the week and date]** *at* **[time]** *at* **[health center].**

If NO: *Can I leave a message for (participant)? This is a reminder from the Back to Health study at Boston Medical Center that [he/she] has a meeting scheduled on* **[day of the week and date]** *at* **[time]** *at* **[health center].**

Information Session Presentation

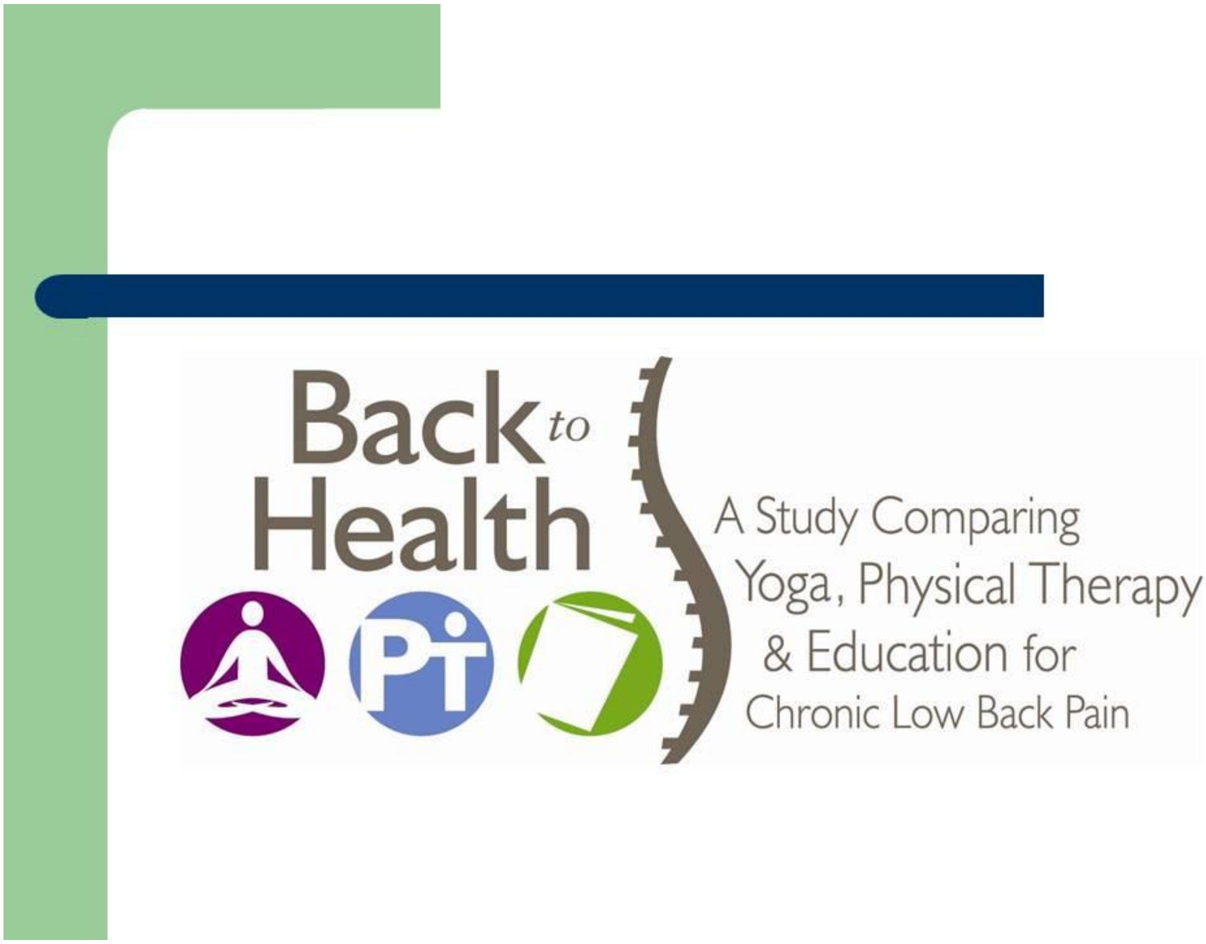

Thank you so much for joining us today, my name is [name]! We are going to tell you more about the Back to Health study and what you can expect if you participate in our study. If you have questions at any time, please feel free to stop me.

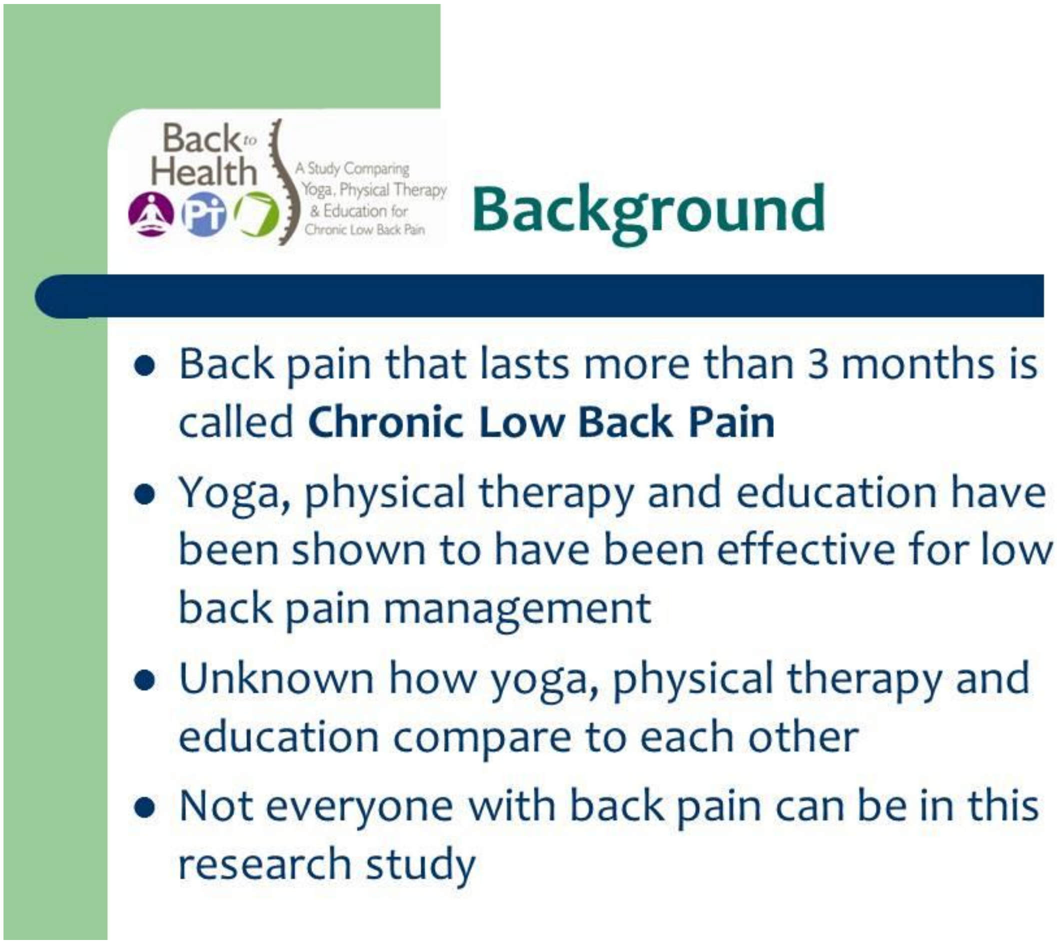

First, I would like to give you a bit of background information about our study. Our study compares 3 different treatments for chronic low back pain. The 3 treatments that we are comparing are yoga, physical therapy, and education. Each of these treatments are effective for low back pain management, but we are not sure which is best. We spoke to each of you over the phone and asked you many questions about your pain to make sure that you have the kind of back pain we are looking for and to make sure that you are able to safely participate.

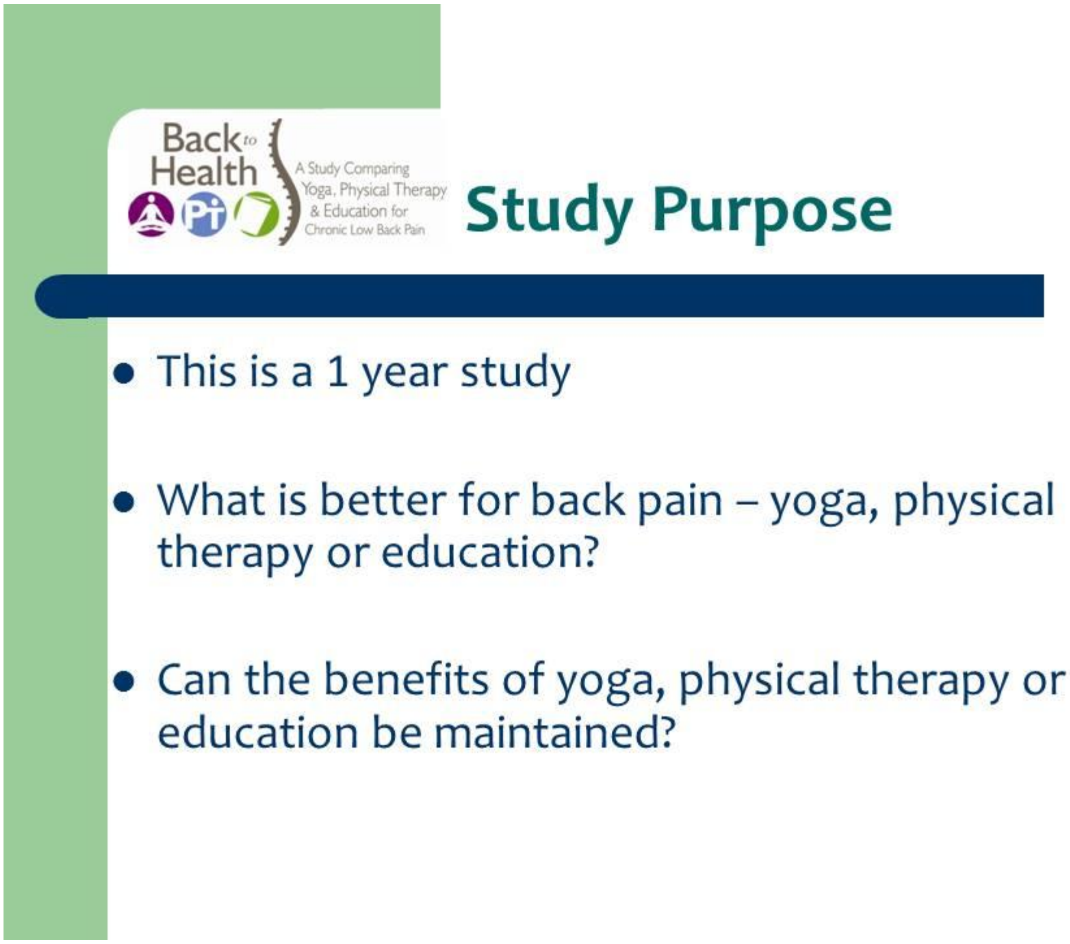

We are doing a year-long study to find out if yoga, physical therapy, or education is the best for treating this type of back pain. After 12 weeks of the treatment, we also want to see if the benefits of yoga, physical therapy or education can last over time.

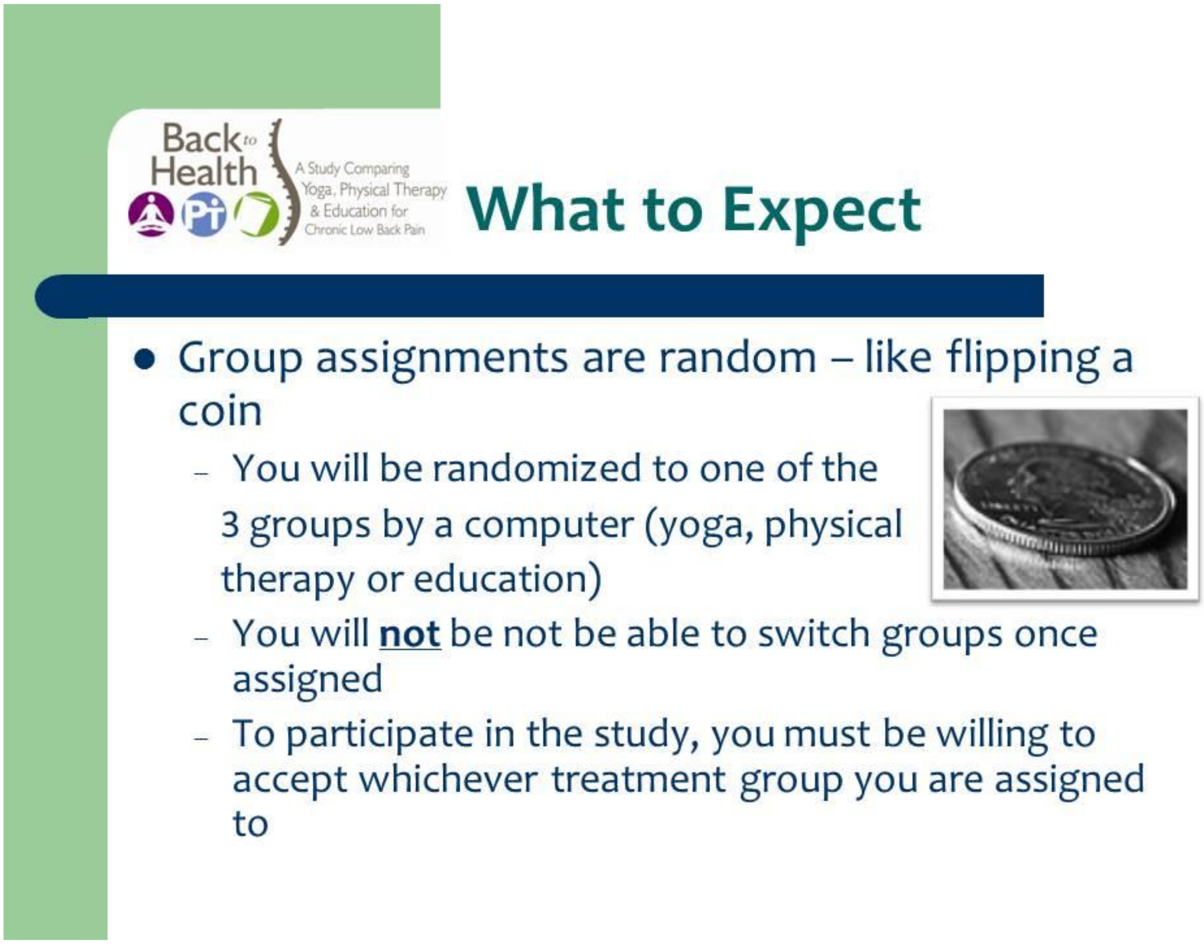

So, what can you expect by participating in this study? Each person will be randomly assigned to one of the 3 groups by a computer. This makes the process completely random, like flipping a coin. So this means that you will not be able to pick which group you are in and you will not be able to switch groups once you are assigned. This is really important because if you agree to be in the study you will need to be willing to accept whichever treatment group you are assigned to—either yoga, physical therapy, or education. Can I answer any questions so far?

© Boston Medical Center

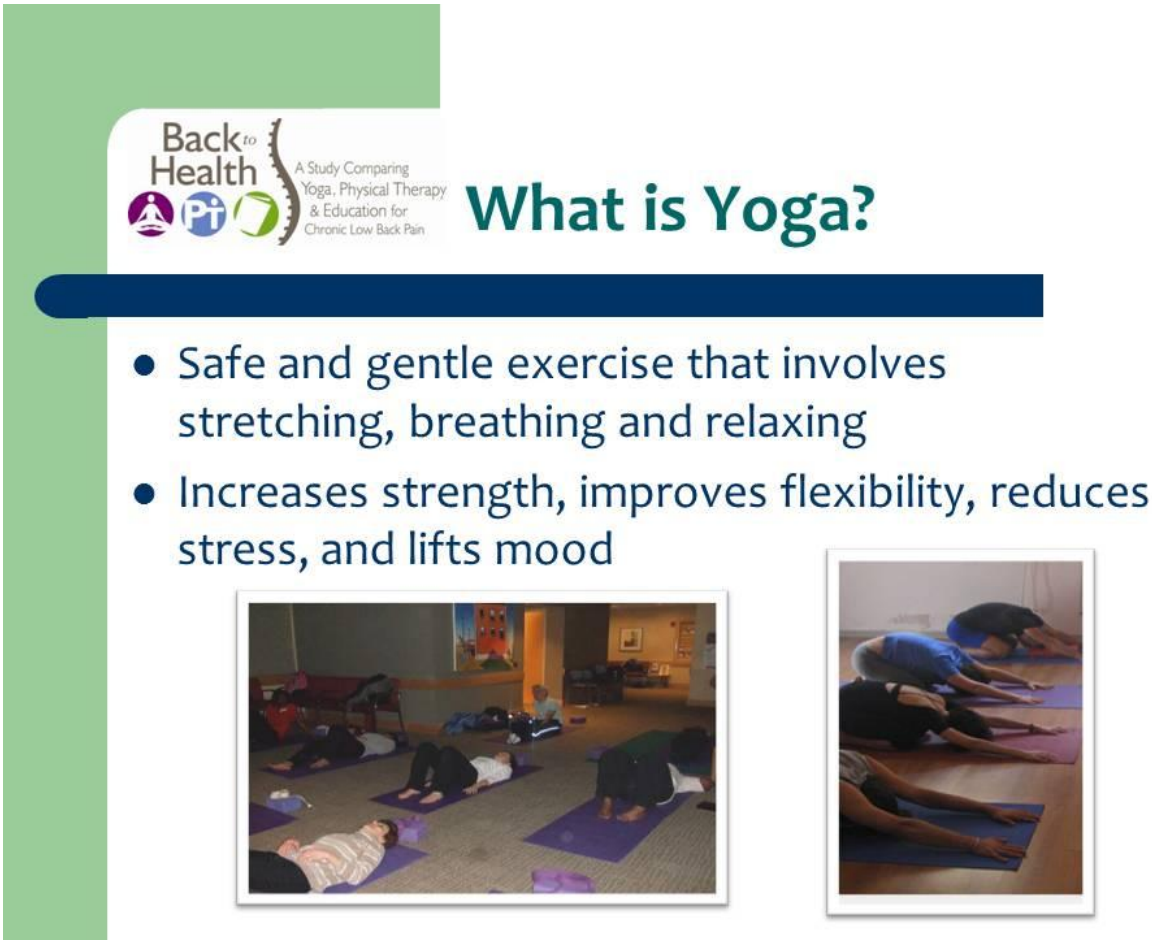

Now we are going to talk about each treatment group.

One of the treatments that you may be assigned to is yoga. Yoga is a safe and gentle exercise that involves stretching, breathing, and relaxing. It increases your strength, improves your flexibility, reduces your stress, and lifts your mood. The yoga we use in this study was specially designed for people like you with chronic low back pain.

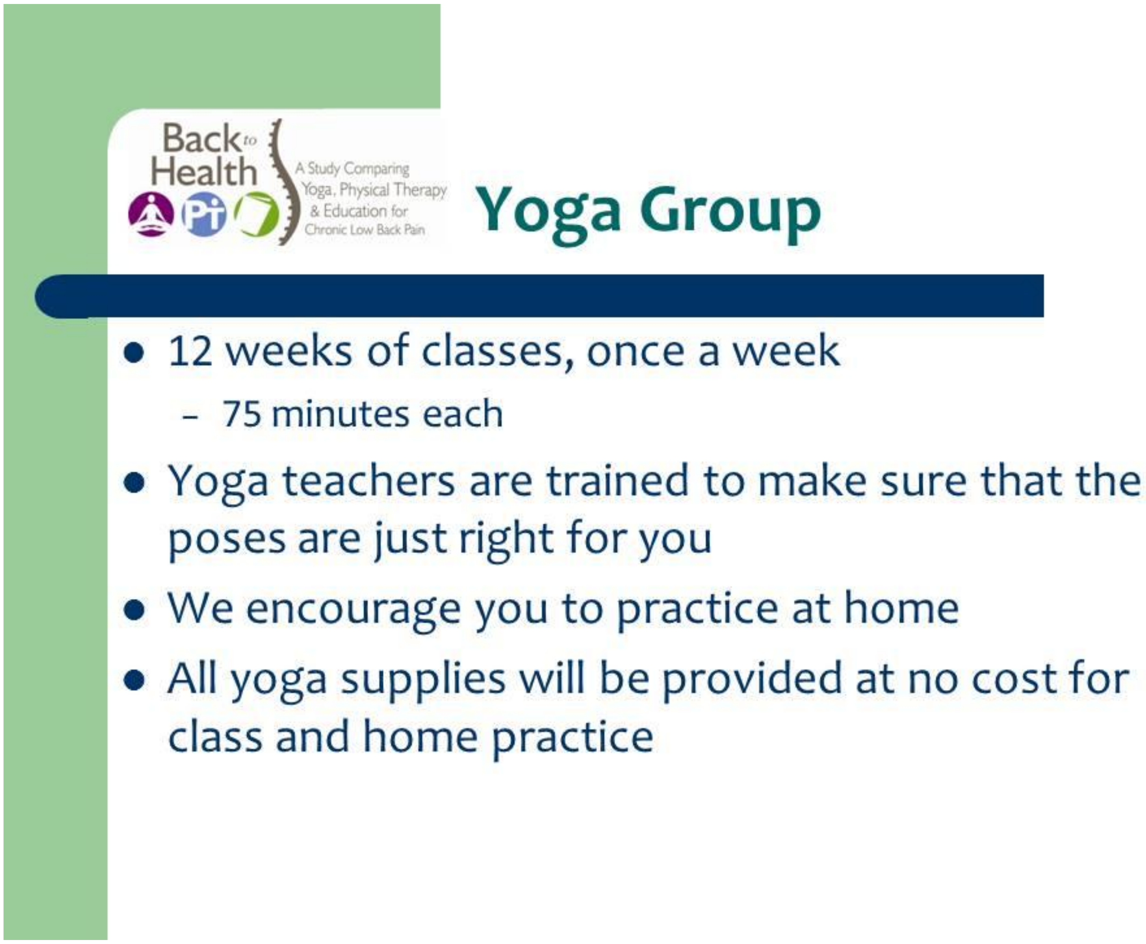

If you are placed in this group, you will have 12 weeks of classes, with one 75-minute class per week. The yoga teachers are trained specifically to make sure that the poses are just right for you, and the other yoga students will also have chronic low back pain. In addition to this weekly class, we encourage you to practice some poses and stretches that you learn on your own, at home. All of the yoga supplies will be given to you at no cost for class and home practice.

© Boston Medical Center

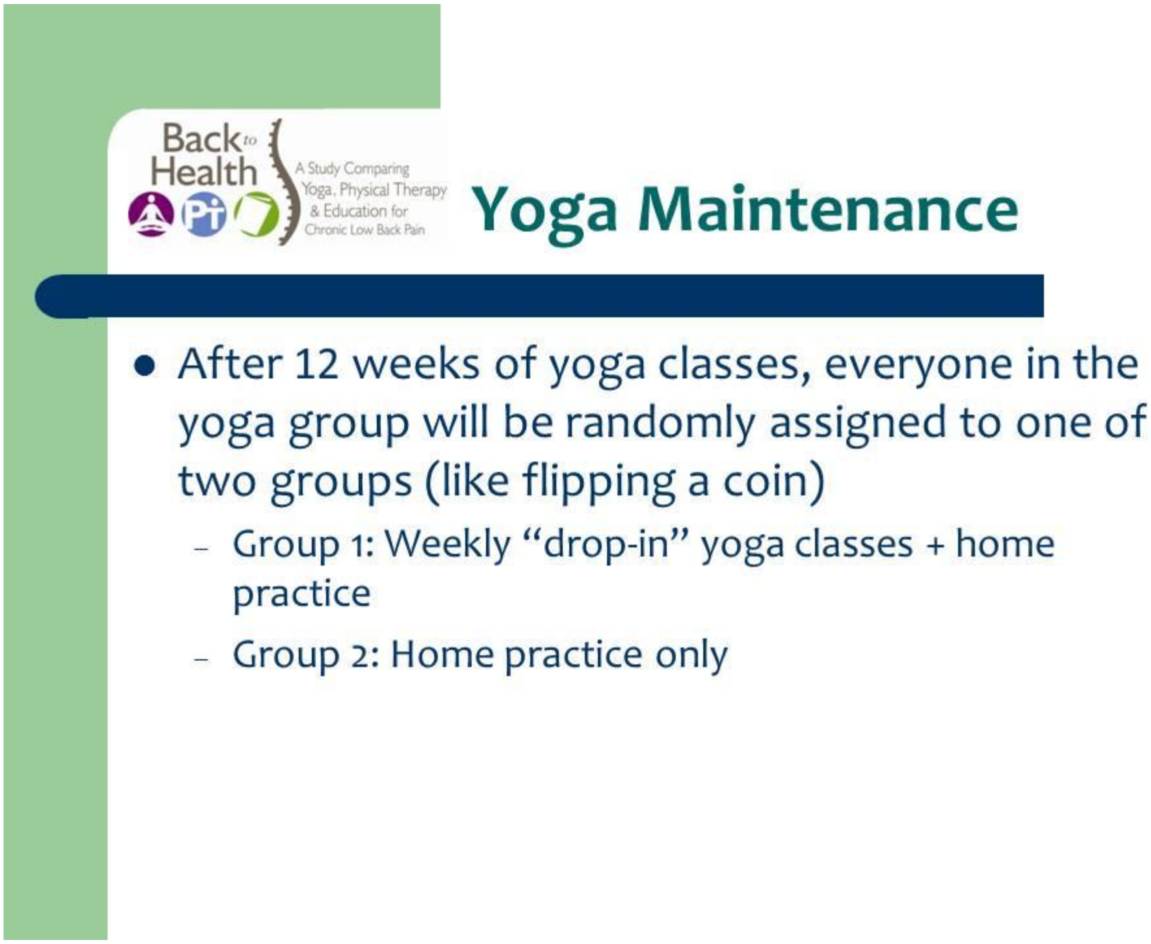

Then, after 12 weeks of yoga classes, people in the yoga group will be randomly split up into one of two groups. One group will only do home practice, meaning they will practice yoga poses at home, rather than in class. The second group will continue to attend their weekly yoga classes and they will continue to do home practice throughout the week.

Information Session Presentation

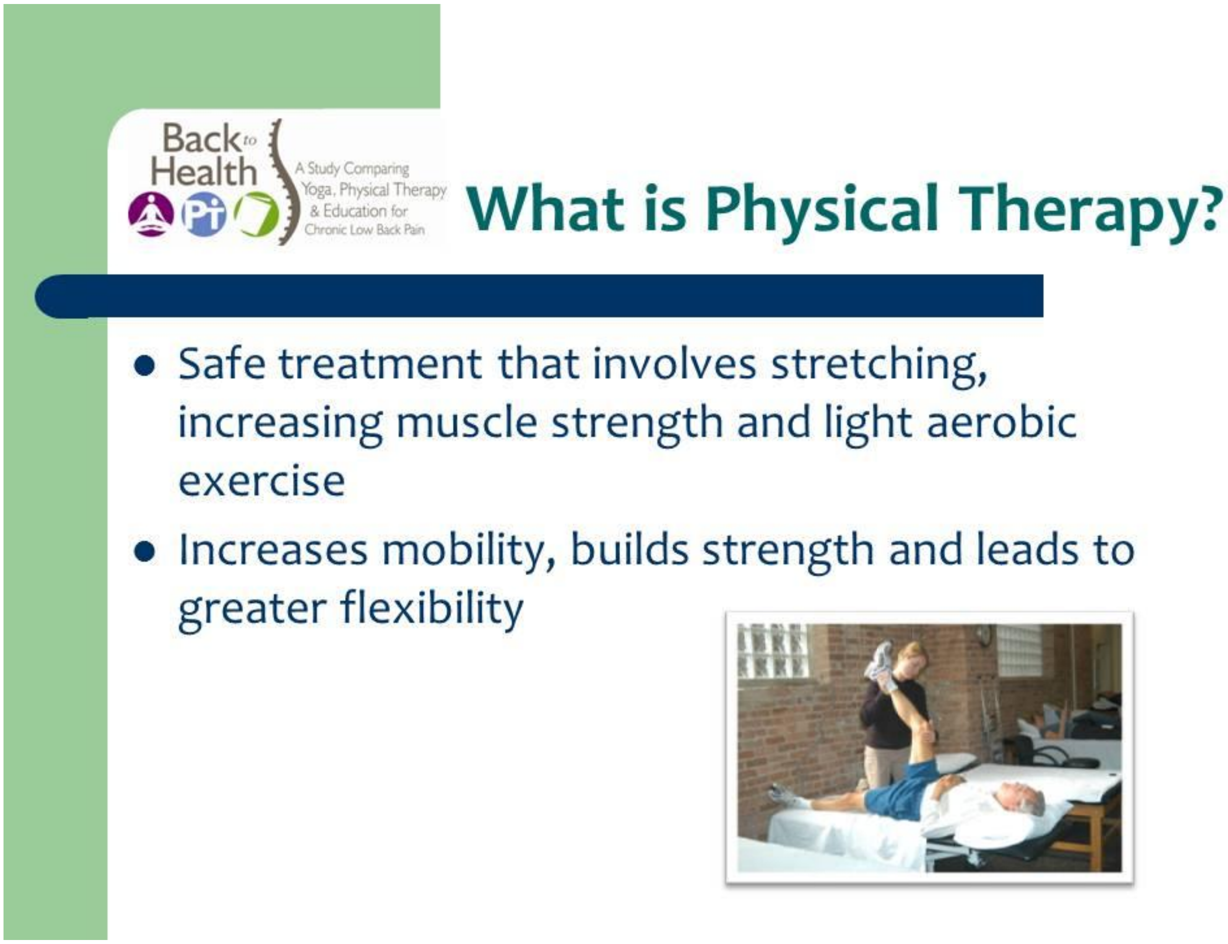

The second treatment group is physical therapy. Physical therapy is a safe treatment that involves stretching, increasing muscle strength, and light aerobic exercise. It helps with low back pain by increasing mobility and flexibility and building strength. The physical therapy in this study was created by a physical therapy expert for people with chronic low back pain.

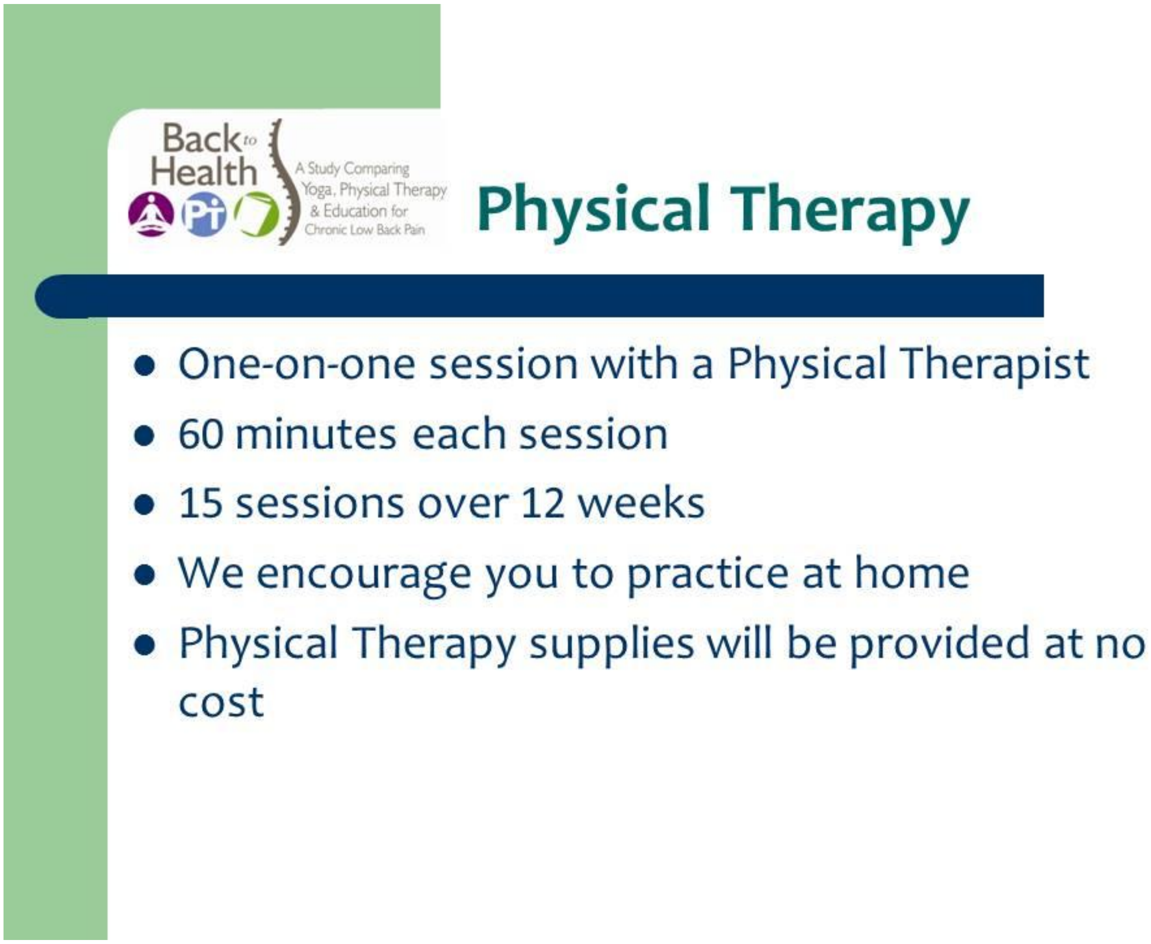

If you are placed in this group, you will have one-on-one sessions with a physical therapist. There are 15 60-minute sessions over the course of 12 weeks. This means that there will be one session per week, with the exception of weeks 2, 3, and 4, where there will be two sessions per week. The physical therapist will teach you some exercises that we would encourage you to practice at home. Additionally, physical therapy supplies will be provided at no cost.

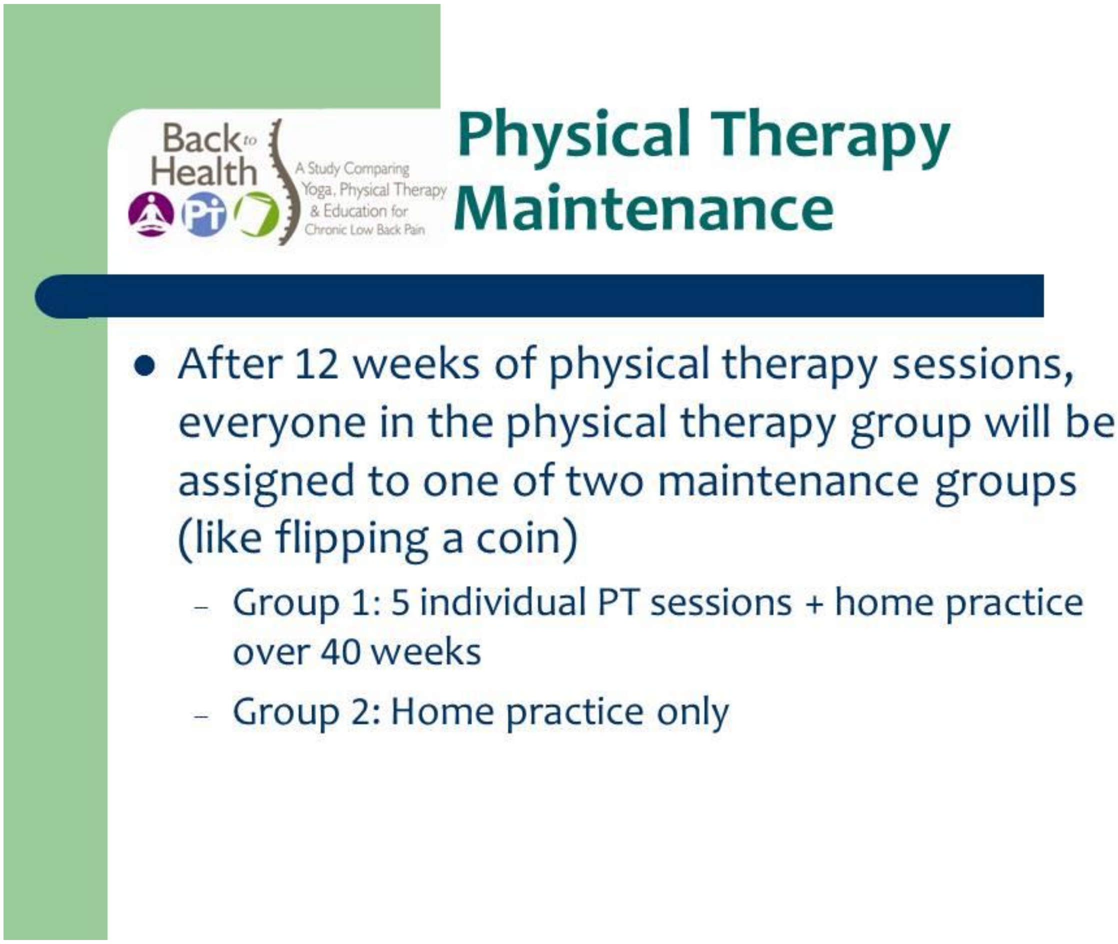

After the initial 12 weeks of physical therapy sessions, the group will be split up in a way similar to the yoga group. They will be split up into two groups, one that will continue doing home practice. The other group will have an additional 5 physical therapy sessions, as well as home practice over the final 40 months.

© Boston Medical Center
Program for Integrative Medicine & Health Care Disparities
Back to Health Trial

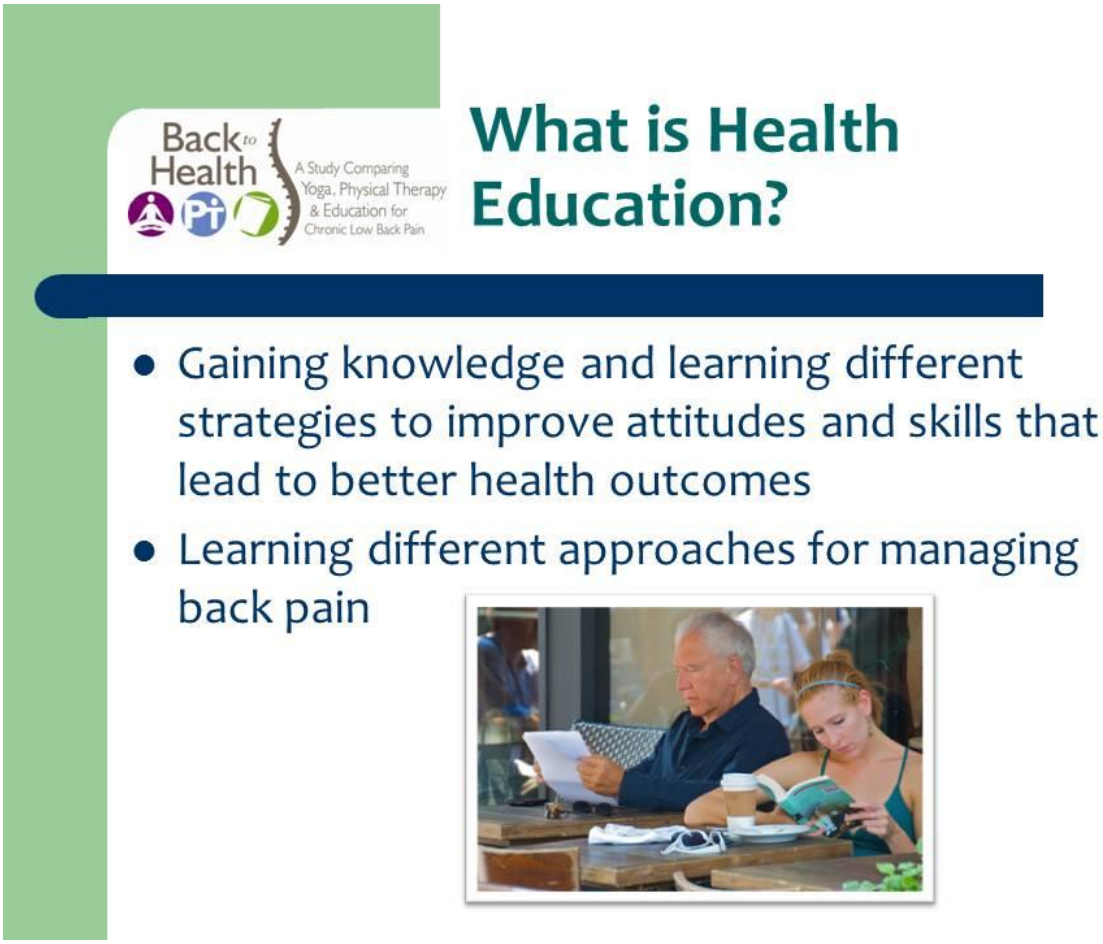

The health education group will gain knowledge and learn different strategies to improve attitudes and skills that lead to better health. Reading this book will teach you different approaches for managing back pain.

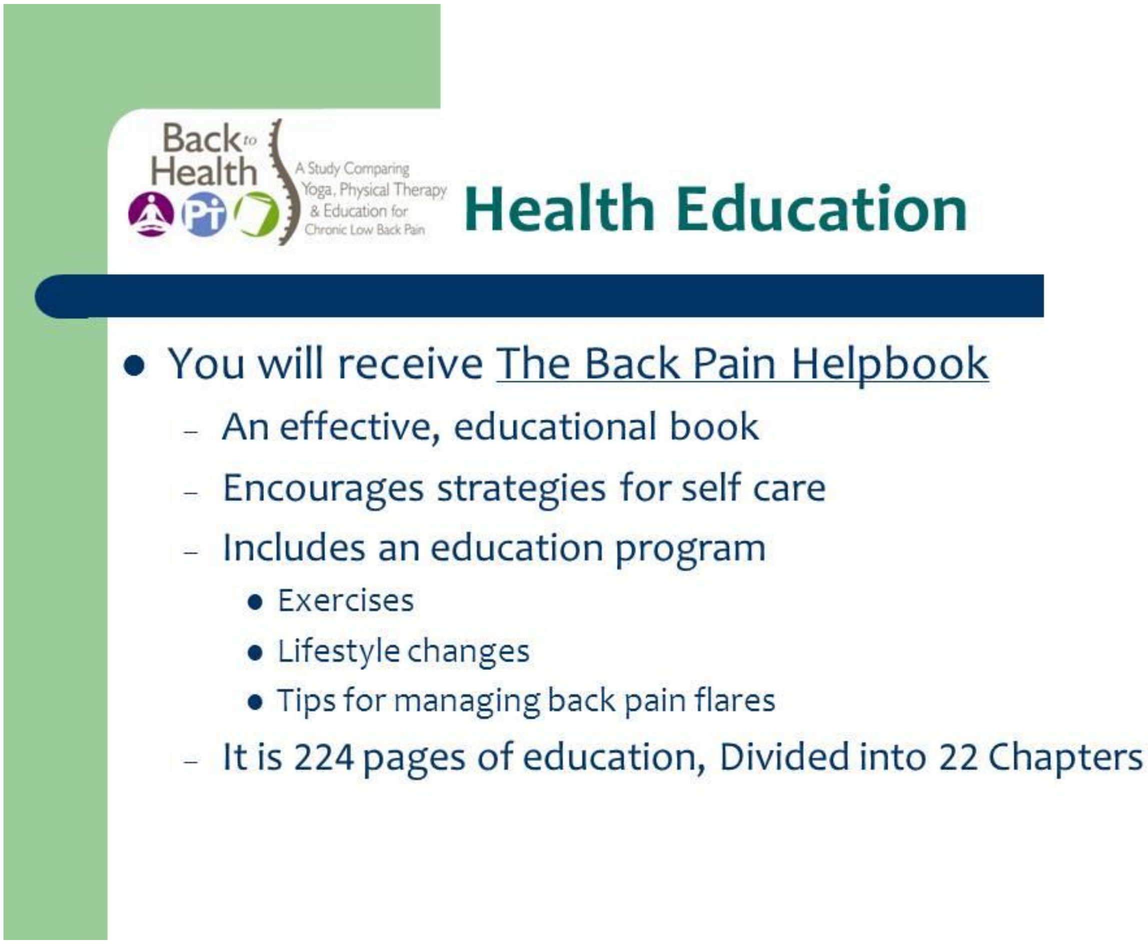

If you are placed into this group, you will receive <u>The Back Pain Helpbook</u>. This is an effective, educational book that encourages strategies for self-care for your back pain. This book includes an education program, which provides different exercises, lifestyle changes, and tips for managing back pain flares. It is 224 pages, divided into 22 chapters.

Do you have any questions about anything that we've gone over so far?

© Boston Medical Center

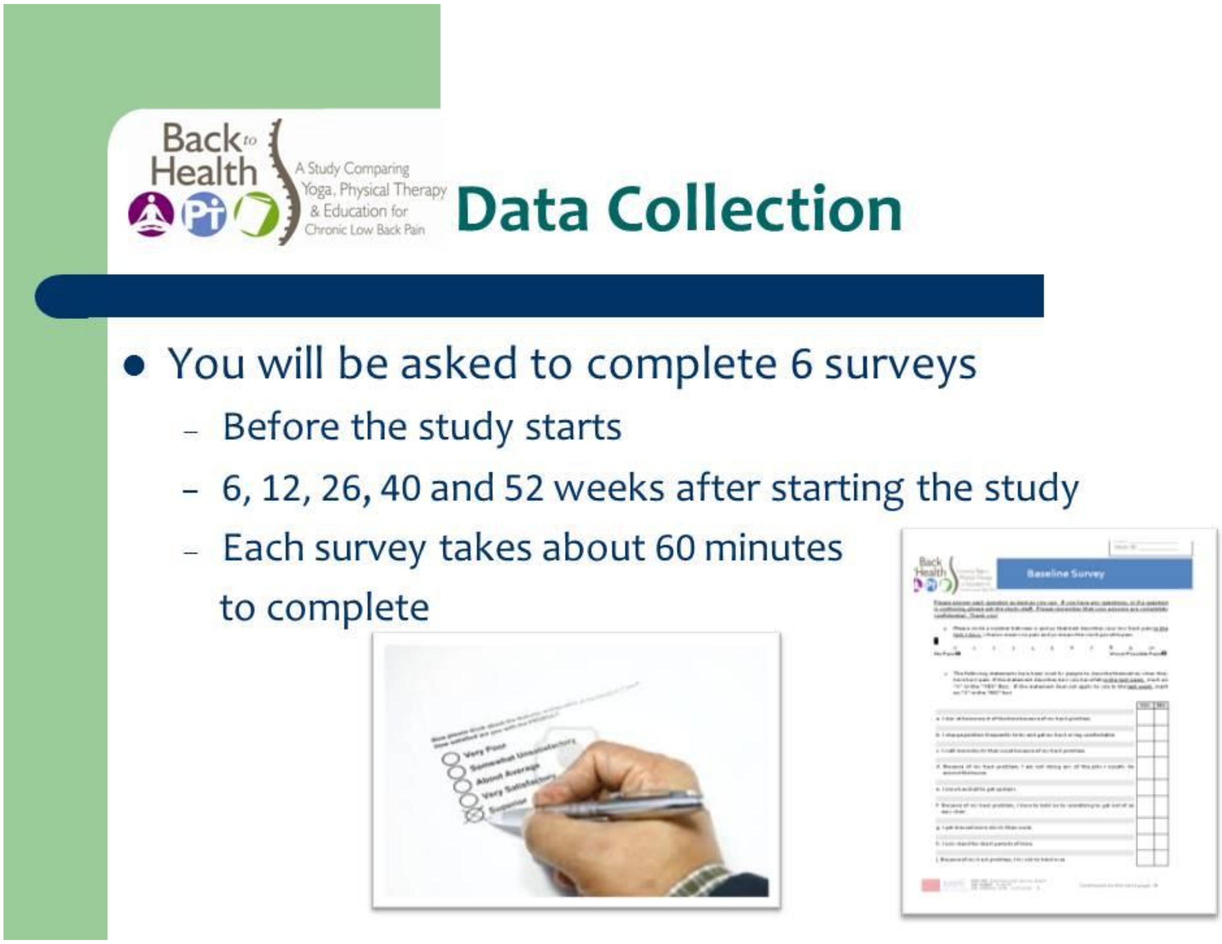

Along the way, you will be asked to complete surveys so that we are able to see how you are doing. There will be 6 surveys. One will be before the study starts, and the others will be during weeks 6, 12, 26, 40, and 52. Each survey takes about 60 minutes to complete.

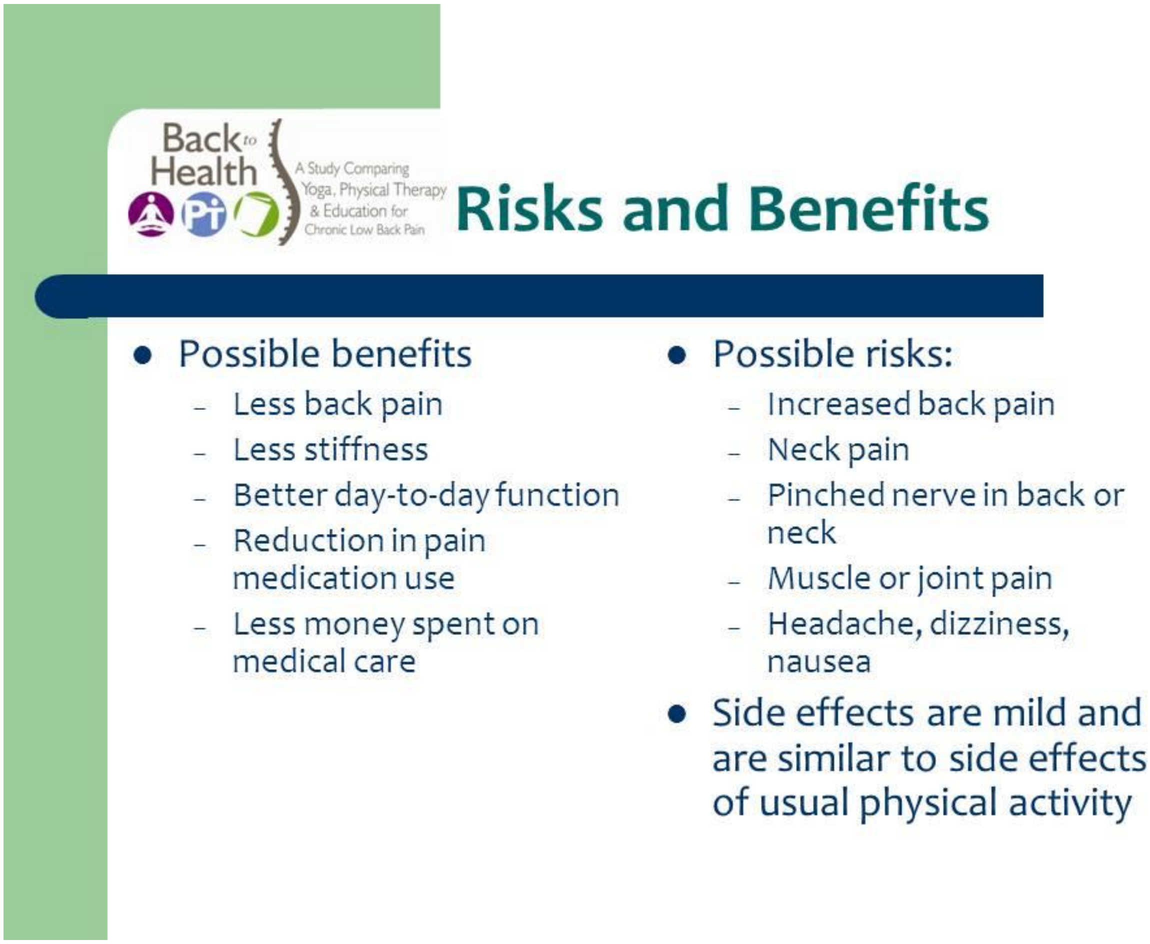

There are both possible risks and benefits for the treatment groups, as listed on the slide. Some possible benefits are less back pain, less stiffness, better day-to-day function, reduction in pain medication use, and less money spent on medical care. Some possible risks are increased back pain, neck pain, a pinched nerve in the back or neck, muscle or joint pain, a headache, dizziness, or nausea. Side effects are mild and are similar to side effects of usual physical activity.

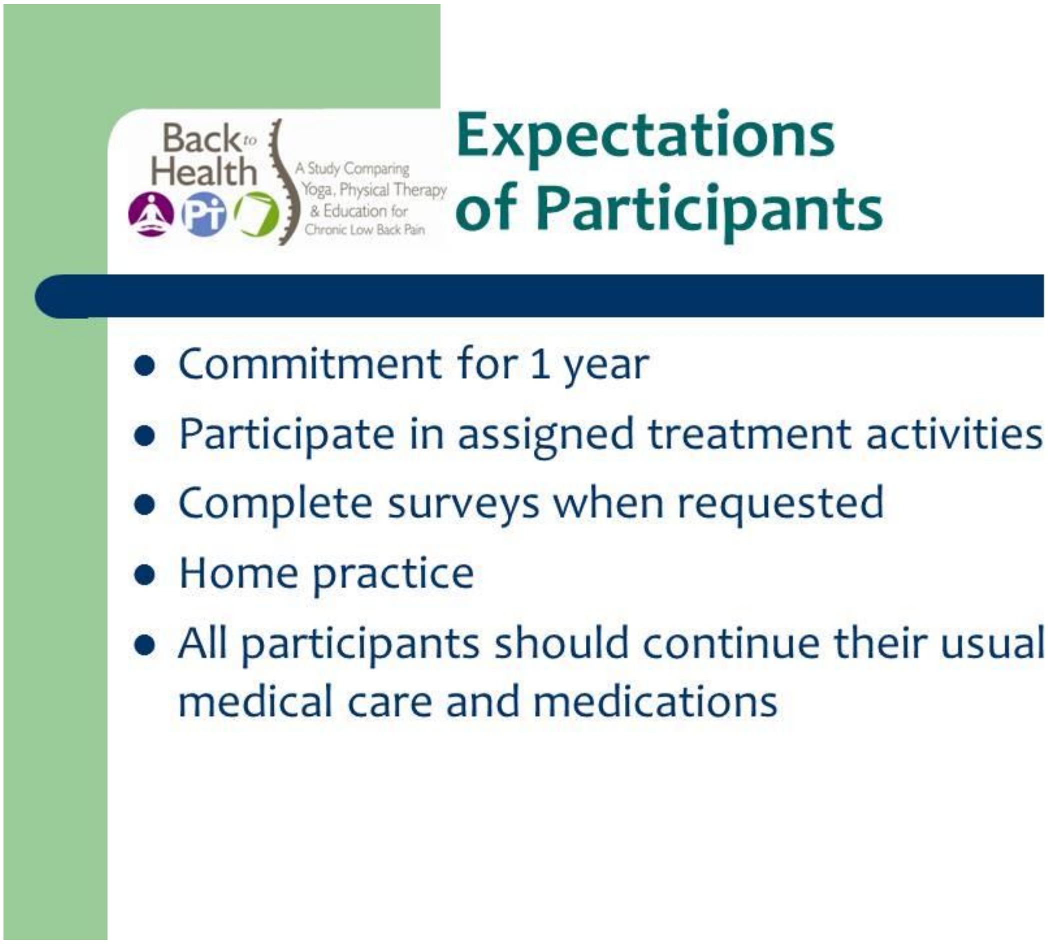

As a participant, we expect that you are willing to commit one year to the study. We also expect you to participate in assigned treatment activities, to complete surveys when requested, and to participate in home practice. All participants should continue their usual medical care and medications.

© Boston Medical Center

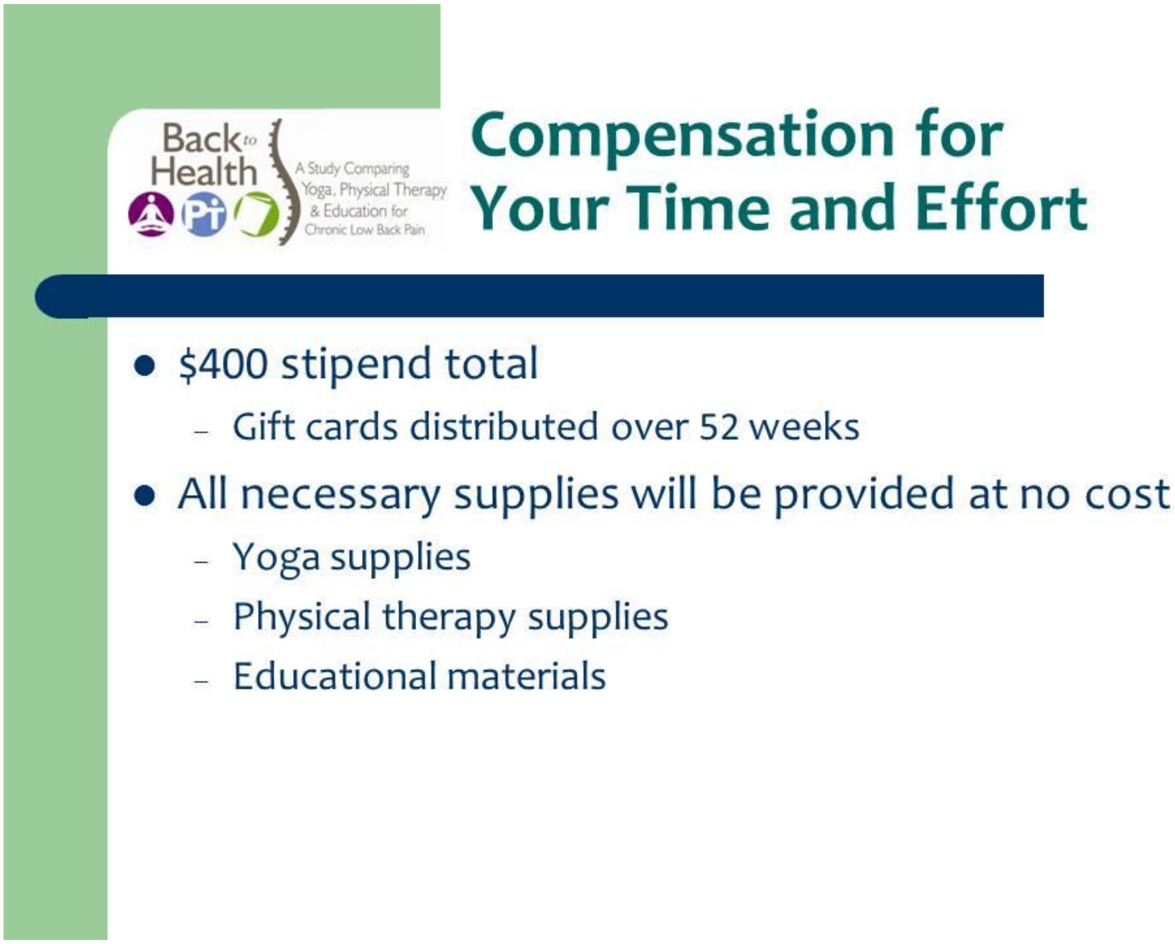

As a compensation for your time and effort, you will receive a total of $400 in the form of Target gift cards over the course of 52 weeks. These gift cards will be given to you after you finish each of your surveys. Additionally, all necessary supplies will be provided at no cost, which include yoga supplies, physical therapy supplies, and educational materials.

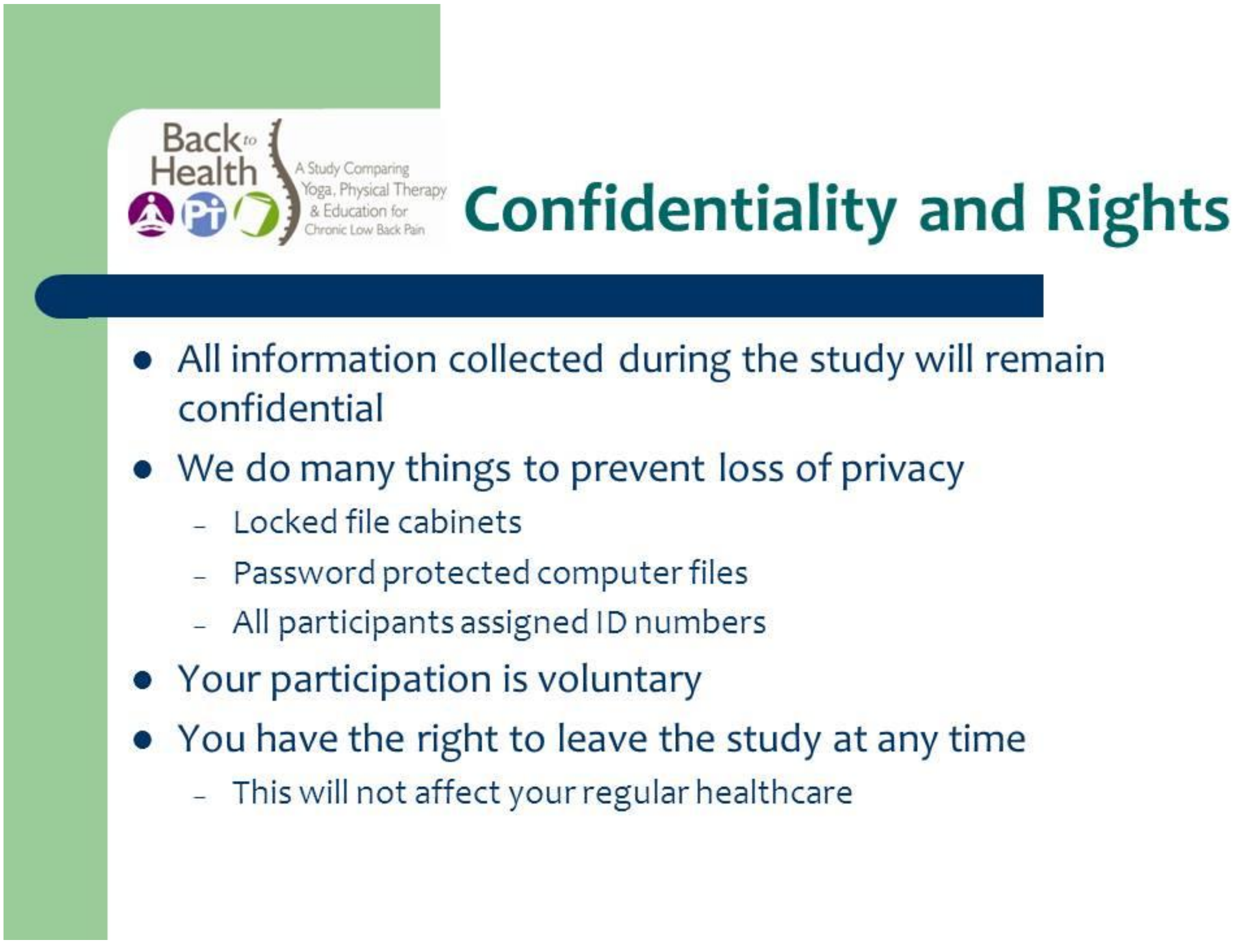

And lastly, all information collected during the study will remain confidential. We do many things to prevent a loss of privacy, such as locking our file cabinets, password-protecting our computer files, and assigning ID numbers to each participant. Your participation is voluntary and you have the right to leave the study at any time. Participating in, or leaving the study will not affect your regular healthcare.

© Boston Medical Center

Contact Information

For General Information and Questions:
(617) 414-6211
backtohealthstudy@bmc.org

Principal Investigator:
Robert Saper, MD MPH
(617) 414-6276
Robert.Saper@bmc.org

Research Coordinators:
Chelsey Lemaster
(617) 414-6201
Chelsey.Lemaster@bmc.org

Dan Do
(617) 414-4464
Daniel.Do@bmc.org

Here is some contact information in case you have any questions. What questions can I answer at this point?

Back to Health Study Fact Sheet

What is the *Back to Health* Study?

- *Back to Health* is a year-long study that compares yoga, physical therapy (PT), and health education for managing chronic lower back pain.
- Participants are **randomly assigned to one of the three treatment groups** by a computer.

How the Yoga Treatment Works:

- Yoga is a safe, gentle exercise that involves stretching, breathing, and relaxing which increases your strength, improves flexibility, reduces stress, and lifts your mood.
- If placed in yoga, you will attend **12 group classes** (75-minute each) once a week for 3 months.
- **After 3 months**, everyone in the yoga group is randomly placed into one of two groups:
    - **Group 1**: Continues to attend yoga classes and home practice for the next 9 months
    - **Group 2**: Practices yoga at home for the next 9 months

How the Physical Therapy Treatment Works:

- PT is a treatment that involves stretching, increasing muscle strength, and light aerobic exercise that helps your back pain by increasing mobility, flexibility, and building strength.
- If placed in PT, you will attend **15 one-on-one sessions** with a physical therapist over 3 months (2 sessions per week will take place on weeks 2, 3, and 4)
- **After 3 months**, everyone in the PT group will be randomly placed into one of two groups:
    - **Group 1**: Receives 5 additional PT sessions and practices at home practice for the next 9 months
    - **Group 2**: Practices PT at home practice for the next 9 months

How the Health Education Treatment Works:

- Health education will teach you strategies and skills to improve you back pain.
- You will receive The Back Pain Helpbook that includes tips for self-care, exercises, and lifestyle changes that will help you to be successful in managing your back pain.
- If placed in health education, you will practice the treatment at home for the year and receive **check-in calls every 3 weeks** from study staff.

Data Collection:

- You will complete **six surveys** over the course of the year.
- Surveys take place at the start of the study (baseline), 6 weeks, 3 months, 6 months, 9 months, and 1 year.

Compensation for Your Time and Effort:

- You can receive up to **$400 in the form of Target gift cards** after each survey you complete.
- All supplies for your particular treatment group will be **provided at no cost and are yours to keep!**

Back to Health Study Fact Sheet

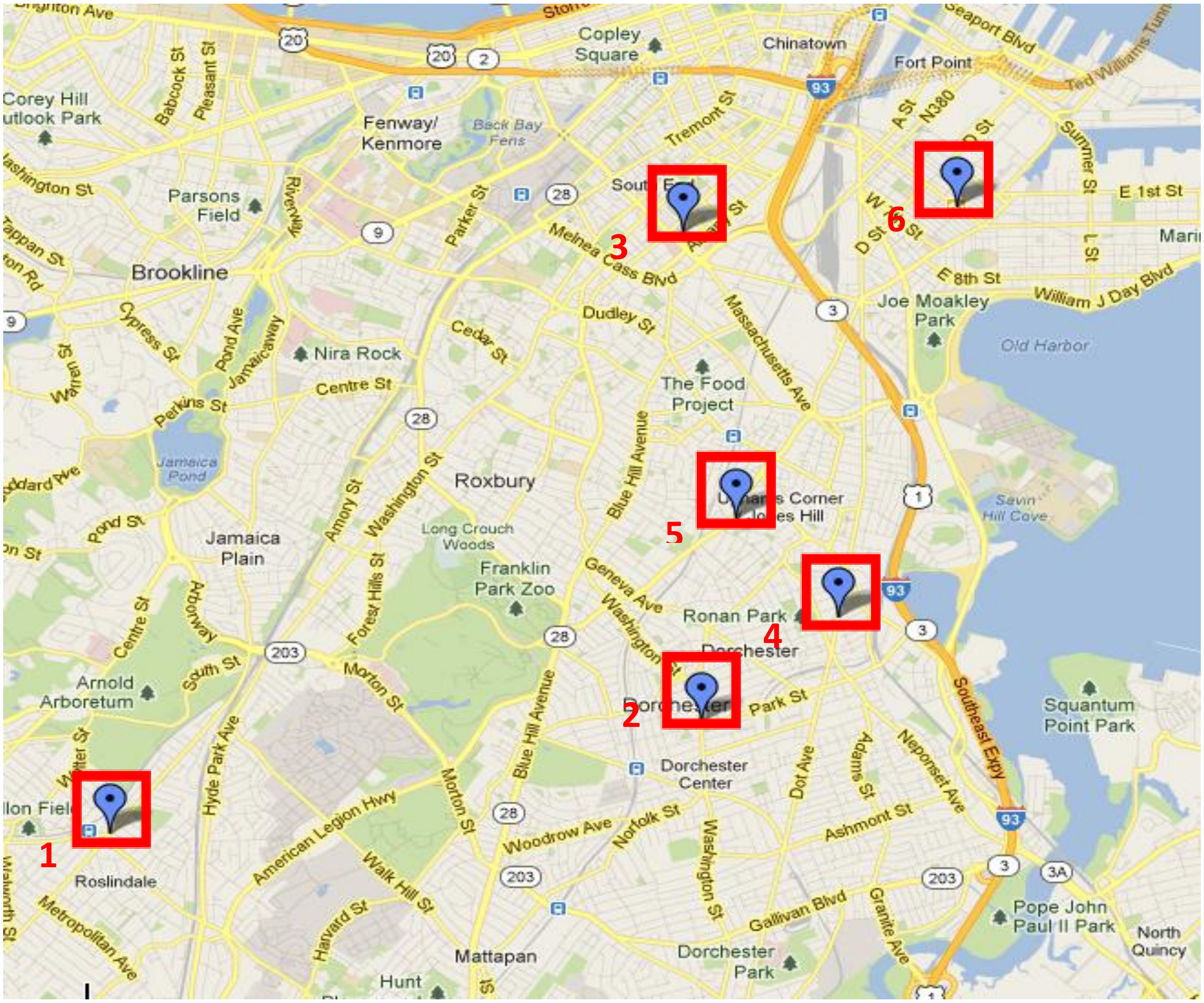

Site Locations:

1. Greater Roslindale Medical & Dental Center	4199 Washington Street<br>Roslindale, MA 02131
2. Codman Square Tech Center	450 Washington St.<br>Dorchester, MA
3. Boston Medical Center	1 Boston Medical Center Place<br>Boston, MA 02118
4. Dorchester House Multi-Service Center	1353 Dorchester Avenue<br>Dorchester, MA 02122
5. Upham's Corner Health Center	415 Columbia Rd.<br>Dorchester, MA
6. South Boston Community Health Center	409 W Broadway<br>Boston, MA 02127

© Boston Medical Center

Informed Consent Checklist and Teach Back

Materials Needed

- *2 copies of written informed consent*
- *Welcome letter*
- *Study Contact Information Sheet*
- *Study pen and mug*
- *Informed Consent Progress Note*

General Info

- ☐ Not everyone with back pain can be in this research study.
- ☐ All or part of the research will take place at the following locations: Boston Medical Center, South Boston Community Health Center, Upham's Corner Health Center, Codman Square Tech Center, Greater Roslindale Medical and Dental Center, and Dorchester House Multi-Service Center.
- ☐ This is a year-long study.
- ☐ If you agree to enter the study, you will answer questions about your back pain, pain medicines, quality of life, as well as personal information like your age, sex, work, education, health expense, mental health, and income. We will ask you these questions before the start of the study, and after 6, 12, 26, 40, 52 weeks of the study.
- ☐ There is no cost for you for being in the study. To compensate you for your time and energy that you put into this study, you will receive a stipend of up to $400 over the course of the one year study in the form of gift cards each time you complete a survey. You will also receive various treatment supplies that you can keep.
- ☐ Taking part in this study is voluntary. You have the right to refuse to take part in this study. If you decide to be in the study and then change your mind, you can withdraw from the research. Your participation is completely up to you. Your decision will not affect your being able to get health care at this institution or payment for your health care. It will not affect your enrollment in any health plan or benefits you can get.

Three Treatment Groups

- ☐ You will be assigned to one of three treatment groups: yoga, physical therapy, or health education.

Informed Consent Checklist and Teach Back

- ☐ The group that you are assigned to will be decided randomly by a computer. Random means that the group you are assigned to is pure chance, like rolling a die.

- ☐ As a participant, you will receive either one-on-one treatment with a physical therapist, yoga classes in a group, or receive a back pain education book to read at home.
  - Those who are in the physical therapy group will receive treatments that involve stretching and strengthening exercises, and aerobic exercise.
  - Those in the yoga group will participate in classes designed to be safe for people who have never done yoga before and have chronic low back pain.
  - Those in the health education group will receive a book written specifically for people with chronic low back pain.

- ☐ After 12 weeks of the study, for those who are assigned to the physical therapy group, we will tell you if you will be in the physical therapy maintenance group or not. For those who are in the yoga group, we will tell you if you will be in the yoga maintenance group or not. There is a 50-50 random chance that people in the yoga and physical therapy groups will be in the maintenance groups or not. The maintenance phase will last for 40 weeks.

- ☐ All participants assigned to the health education group will continue with education during the 40 week maintenance phase.

Risks and Discomforts

- ☐ There is always a chance that physical activity may make your back feel worse or that an injury can happen. For example, neck pain, muscle spasm, and joint pain are possible. If you do have any side effects from the treatment, or an injury from the treatment, please tell the research staff right away.

- ☐ For women: Women who are pregnant or planning to get pregnant cannot participate in the study. Women who have sex with men should use birth control during the course of the study.

Benefits

- ☐ Benefits of this study may include less back pain, less stiffness, and an increase in your ability to do everyday activities. Some people many not experience any of these benefits.

Informed Consent Checklist and Teach Back

- ☐ All three treatment options may also help you work better at your job, need less pain medicine, and spend less money on your medical care.

- ☐ All participants will receive materials that are related to their treatment group at no cost (yoga supplies, physical therapy materials, educational booklets).

Confidentiality

- ☐ You will be given a study identification (ID) number. Only your study ID number, and not your name, will be on the surveys you fill out. Only the Principal Investigator, who is in charge of the study, and the research staff, will know what your study ID number is.

- ☐ All of the information that we collect is stored in locked file cabinets and password protected computers.

- ☐ Sometimes we may need more information from your doctor or medical record to see if you can be in the study. If that is the case, only the Principal Investigator, will contact your doctor. Other than this, we will protect your privacy by not telling anyone that you are in this research study.

Rights

- ☐ You have the right to withdraw at any time.

- ☐ Withdrawing from the study will not affect your receiving healthcare at BMC or your community health center.

© Boston Medical Center

Informed Consent Checklist and Teach Back

Teach Back: I have a few questions to make sure I've explained everything to you about our study:

- ☐ Can you please tell me how long does the study lasts?
- ☐ How do we decide who is placed in each treatment group?
- ☐ What should you do if you have any side effects from either of the treatments?
- ☐ What are some risks and benefits you can expect by participating in this study?
- ☐ Who has access to your information?
- ☐ When are you allowed to withdraw from the study?
- ☐ What are your questions for me?

ACTION: Have Participant SIGN and DATE Written Consent Form

- ☐ **SIGNATURE**
- ☐ **DATE**

Congratulations! You are now fully enrolled in Back to Health Study. We have for you a letter that officially welcomes you to our study (hand participant envelope) and a small token of our appreciation (hand participant mug).

Do you have any questions for me?

Thanks again and we're excited that you're going to be a part of our study. Have a wonderful day!

Continuous Monitoring of Recruitment Progress

We employ a PDSA (Plan–Do–Study–Act) quality improvement process on a weekly basis to continuously monitor and improve our recruitment progress. Past week, cohort-to-date, and study-to-date statistics are presented and discussed at the Study Team weekly meeting using a standardized format (see example of PowerPoint recruitment summaries on the following pages). Based on these data, the team discusses progress and can make weekly decisions regarding whether to make any changes in recruitment strategy.

Recruitment data collection begins with the initial participant inquiry. At this stage contact information is collected and a brief pre-screening questionnaire is administered including age and the method via which the participant learned about the study (e.g., mailing, posted flyer, physician recommendation). Assuming the participant is in the eligible by age, the full eligibility screening survey is then administered and data are entered directly into StudyTrax. Numbers of participants who are found to be eligible, ineligible and reasons for ineligibility are recorded. Eligible participants then are invited to an in-person informational session where they receive in depth information about the study, have opportunities to ask questions, and enroll if desired through signing informed consent.

Recruitment and eligibility data are compiled and presented weekly. Selected metrics that are highlighted in weekly meeting summaries include:

- Study recruitment activities completed (e.g., number of mailed letters, flyers posted, community events attended)
- Numbers of inquiries by interested participants
- Frequencies of different ways which participants learned about the study
- Eligibility pass/fail rates
- Frequencies of specific ineligibility reasons (e.g., sciatica, spinal canal stenosis, recent PT)
- Numbers of total participants enrolled
- Number of in-person informational sessions scheduled
- Follow-ups needed for participants with uncertain eligibility
- Follow-ups needed for individuals who have inquired about the study but have not yet completed eligibility screening

Continuous Monitoring of Recruitment Progress

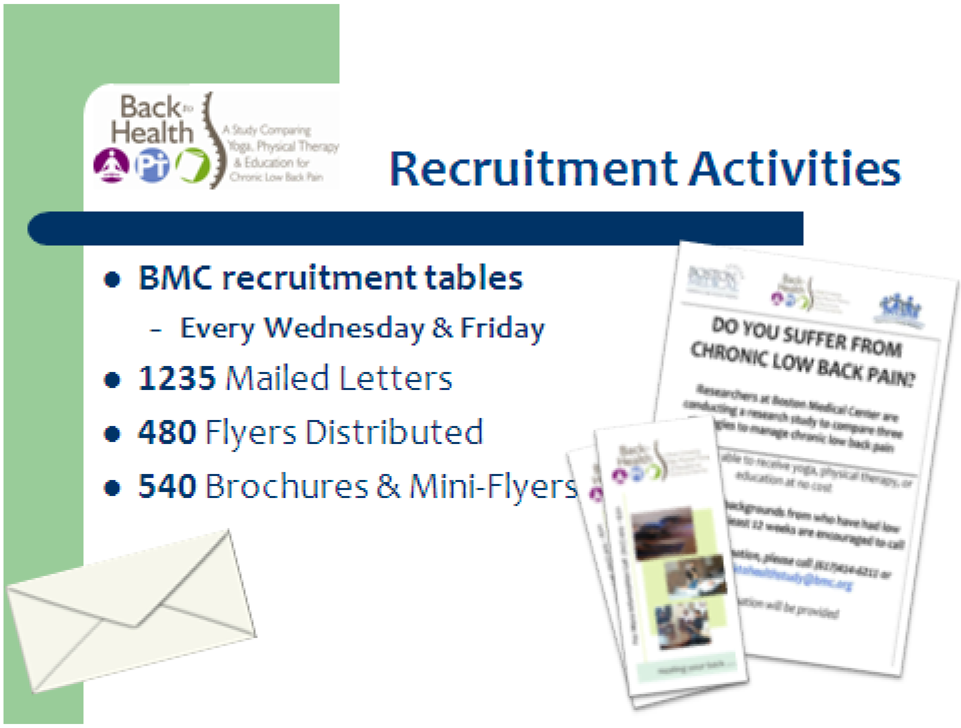

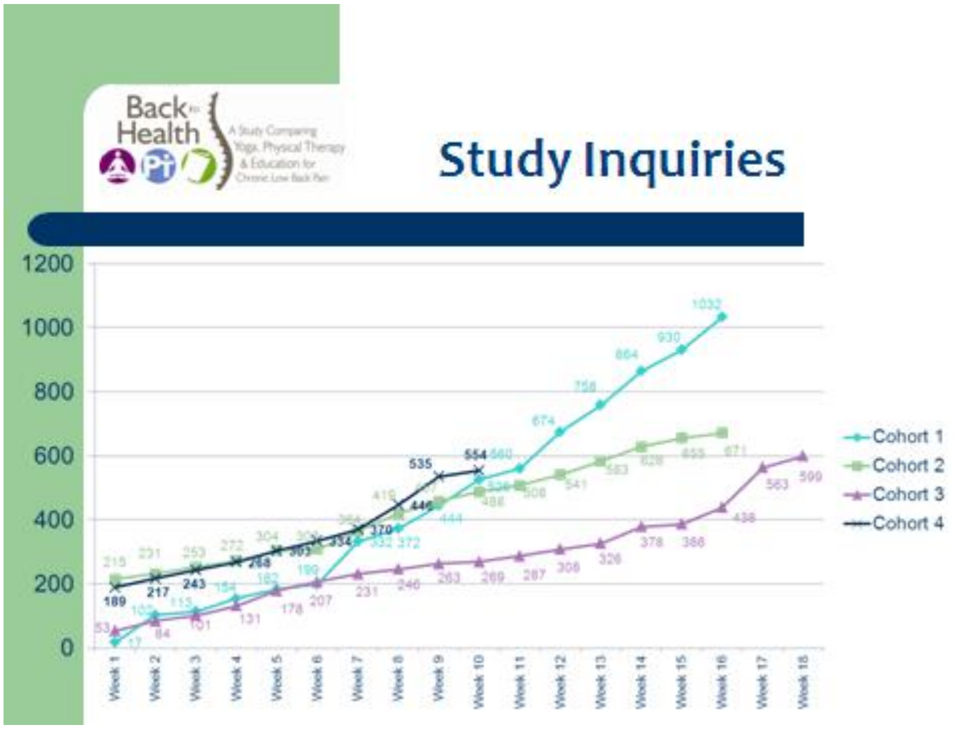

Continuous Monitoring of Recruitment Progress

Recruitment Effort Statistics Cohort 1 & 2

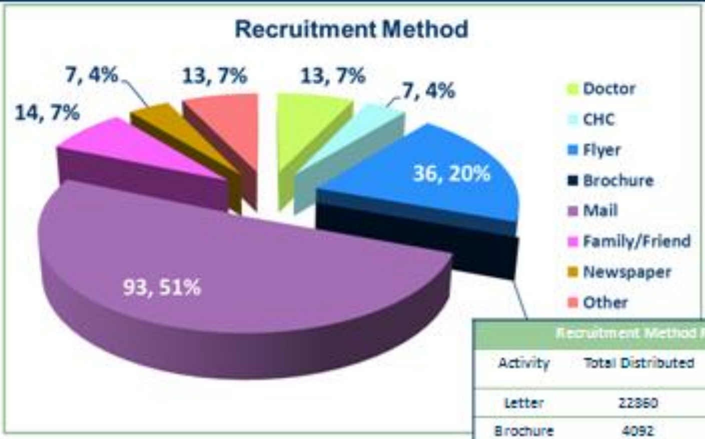

Recruitment Method Return Rate

Activity	Total Distributed	Enrolled	% Return
Letter	22860	93	.4%
Brochure	4092	0	0%
Flyer	965	36	3.7%

Cohort 3 Recruitment Plan

- **Focus on top inquiry yields:**
  - *Flyers*
  - *Letters*
  - *Doctors/Family/Friends*
- **Increase community involvement**
  - Visiting churches
  - Tables at community health centers
  - Presentations at CHCs
- **Mailing to South Boston, South End, and BMC**
- **Participant referral incentive**

Continuous Monitoring of Recruitment Progress

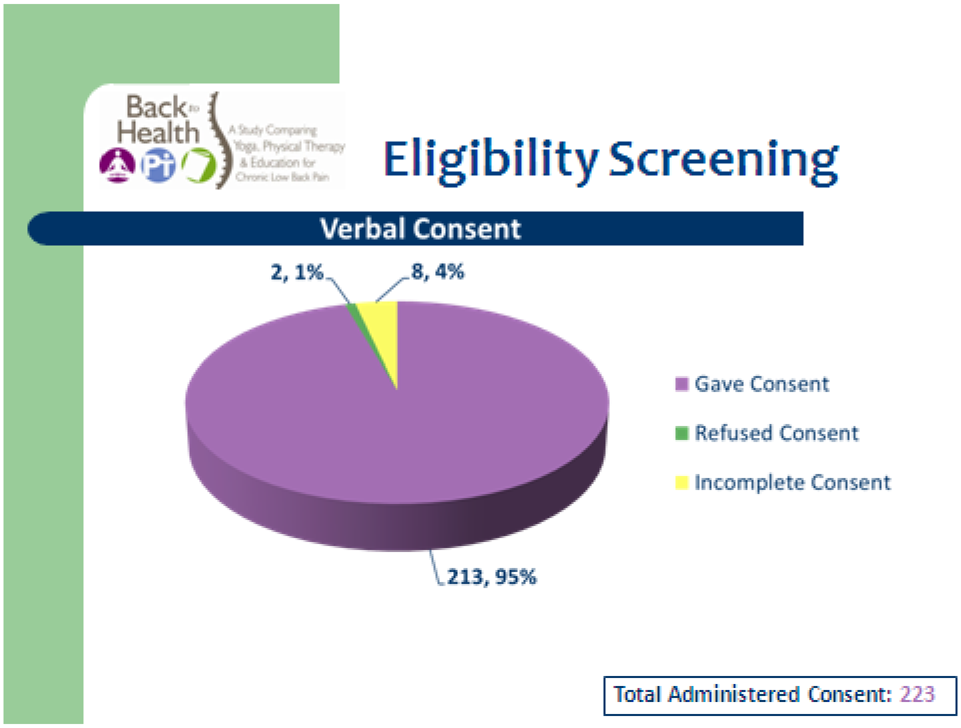

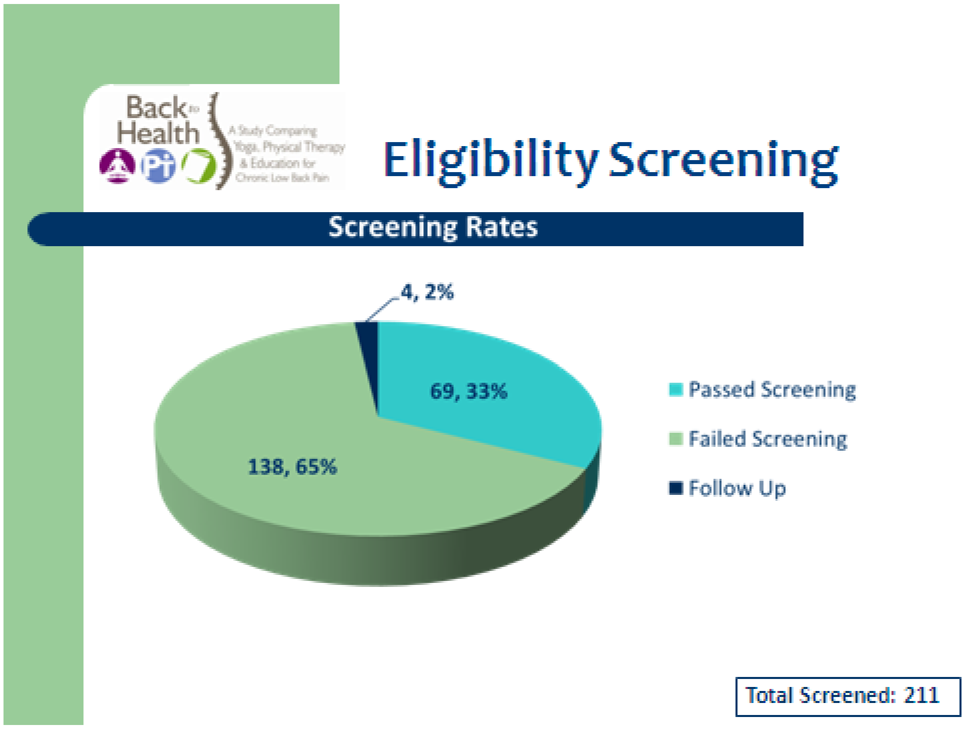

© Boston Medical Center

Yoga Policies and Procedures

Yoga Treatment Arm Flow Sheet

Step	Forms Needed
**Randomization**	• Yoga Randomization Phone Call Script
↓	
**Pick Up Materials**	• Welcome Packet<br>• Take home materials<br>• Yoga Class General Reminder Script
↓	
**6 Week Data Collection**	• 6-Week Questionnaire<br>• Survey Administration Script<br>• Survey Administration DOs and DON'Ts
↓	
**12 Week Data Collection**	• 12-Week Questionnaire<br>• Survey Administration Script<br>• Survey Administration DOs and DON'Ts
↓	
**Maintenance Randomization**	• Yoga Maintenance Randomization Phone Call Script
↓	
**26 Week Data Collection**	• 26-Week Questionnaire<br>• Survey Administration Script<br>• Survey Administration DOs and DON'Ts
↓	
**40 Week Data Collection**	• 40-Week Questionnaire<br>• Survey Administration Script<br>• Survey Administration DOs and DON'Ts
↓	
**52 Week Data Collection**	• 52-Week Questionnaire<br>• Survey Administration Script<br>• Survey Administration DOs and DON'Ts

END OF STUDY

Yoga Randomization Script

Overview:

Once participants have completed their Baseline Survey, they will receive their treatment assignment by an unblinded staff member at least 72 hours before the start of their study cohort.

Suggested Script:

Hello, this is __________ with the Back to Health study. Is ____________ available?

If yes:

> *May I speak with to ______? Hello _______. I'm* **[give name]** *from Back to Health study team. I'm calling you let you know that you have been randomly selected to receive the yoga classes as your treatment. Your yoga class will take place on* **[day(s) of the week]** *starting with [start date]. Your yoga class will be held at* **[location]**. *We will be sending you a letter in the mail with this same information. Please come to class wearing loose fitting clothing so that you can easily move about. We will be asking you to take your shoes off and also your socks if you are comfortable doing so. If you'd like, you are welcome to bring a towel with you in case you perspire, although keep in mind the class will not involve very strenuous exercise.*
>
> **Give details on where yoga class is being held (address, room, floor, etc.)**

If no:

> *When is the best time for me to call ____________ back?*

(It is best if we call them, rather than the participant calling us back so that there isn't any confusion about the message being delivered)

Yoga Class General Reminder Script

Overview:

Participants will receive a reminder phone for yoga classes for week one only. After that, participants can opt in to be on the reminder call list. They will receive a phone 24 hours before their scheduled class.

Suggested Script:

Hello, this is ______________ with Back to Health study. Is _______________ available?

If yes:

May I speak with __________? Hello __________. This is a reminder from Back to Health study at Boston Medical Center that you have a yoga class on **[day of week and date]** *at* **[time]** *at* **[location].**

If no:

When is the best time for me to call back? Thank you.

© Boston Medical Center

Yoga Survey Reminder Call

Overview:

Participants will be called 72 hours before the start of a data collection week to schedule survey administration. Participants will we asked to attend group survey administration sessions; however they may do the survey over the phone if their schedule does not allow them to complete the survey in person.

Suggested Script:

Hello, this is __________ with Back to Health study. Is ____________ available?

If no:

Do you know what time I could reach him/her? Thank you.

If yes:

May I speak with to ______? Hello _______. This is a reminder from Back to Health study at Boston Medical Center that you have a yoga class on **[day of week and date]** *at* **[time and location]**.

This week, we will be collecting data for the study. This will take place at preset times. You can do your survey at times before your yoga class or 24 hours after your class. If you cannot come in person, you can do your survey over the phone. Which option would be most convenient for you?

If in-person:

Okay, we will meet you on **[day of week and date]** *at* **[time and location].**

If over the phone:

Okay, when would you like us to call you to complete the survey? We are available Monday through Saturday from 9:00 am to 7:00 pm.

Do you have any questions? Thank you.

© Boston Medical Center

Yoga Maintenance Randomization Script

Overview:

Maintenance randomization phone calls will be conducted by unblinded study staff only. This call will be made at least one week prior to the start of the maintenance phase.

Suggested Script:

Maintenance Group:

Hello, this is __________ with the Back to Health study. Is ____________ available?

If yes:

> *May I speak with ______? Hello _______. I'm* **[give name]** *from Back to Health study team. I'm calling you let you know that you have been randomly selected to be in the maintenance group. This means that we ask you to attend once a week yoga classes over the course of the next 9 months. Your yoga classes will take place the week of* **[week]** *starting with* **[start date].** *Your yoga class will be held at* **[location]**. *We will be sending you a letter in the mail with this same information.*

If no:

> *When is the best time for me to call ____________ back?*

No Maintenance Group:

Hello, this is __________ with Back to Health study. Is ____________ available?

If yes:

> *May I speak with ______? Hello _______. I'm* **[give name]** *from Back to Health study team. I'm calling you let you know that you have been randomly selected to be in the home practice group. This means that we ask you to continue practicing yoga and following along with your guide book each day. We will be sending you a list of community yoga classes that will be held near where you live. We'll call you to check in and see how everything is going. Please feel free to call us if you have any questions or if you experience any adverse events.*

If no:

> *When is the best time for me to call ____________ back?*
>
> (It is best if we call them, rather than the participant calling us back so that there isn't any confusion about the message being delivered.)

Yoga Adverse Event Call Script

Responsibility

Adverse events will be collected by unblinded study staff. Study staff will alert the PI and the Research Fellow (Adverse Events) of any adverse events within 24 hours.

Suggested Script

Hello, this is __________ with Back to Health study. Is ____________ available?

If yes:

May I speak to _______? Hello _______. I'm **[name]** *from Back to Health study, how are you? I'm calling to check-in and see how you're doing with your yoga classes. Have you had any problems or injuries you think you experienced from your participation in yoga classes?*

If yes:

When did this problem or injury begin?

How soon after your yoga class did this problem or injury begin or did this happen during your yoga class?

If participant experienced adverse event, complete an Adverse Event Form.

If you need anything, please don't hesitate to call us. Have a nice day!

If no:

Do you know what time I could reach him/her? Thank you.

© Boston Medical Center

Yoga Home Practice Call Script

Responsibility

Unblinded study staff will call yoga participants that have been randomized to the home practice group in the maintenance phase.

Suggested Script

Hello, this is __________ with Back to Health study. Is ____________ available?

If yes:

> *May I speak with _______? Hello _______. I'm* **[give name]** *from the Back to Health study, how are you? I'm calling to check-in and see how you're doing with your yoga home practice. How many hours have you practiced yoga in the last three weeks?*
>
> *Have you attended any outside yoga classes?*
>
> > If yes: *Where and how often did you go to these classes?*
>
> *If you need anything, please don't hesitate to call us. Have a nice day!*

If no:

> *Do you know what time I could reach him/her? Thank you.*

First Name, Last Initial: ________________

Back to Health — A Study Comparing Yoga, Physical Therapy & Education for Chronic Low Back Pain

Weekly Home Practice Log

Please record when and for how long you did yoga at home, starting from last week's class.

	Date:__/__/___	Date:__/__/___	Date:__/__/___	Date:__/__/___	Date:__/__/___	Date:__/__/___	Date:__/__/___
Have you practiced yoga at home today?	Yes ☐<br>No ☐	Yes ☐<br>No ☐	Yes ☐<br>No ☐	Yes ☐<br>No ☐	Yes ☐<br>No ☐	Yes ☐<br>No ☐	Yes ☐<br>No ☐
Number of Minutes:	________min.	________min.	________min.	________min.	________min.	________min.	________min.
Comments:							

© Boston Medical Center

STAFF USE ONLY

COLLECTION
Name:____________ Date:_________
Signature: ________________

DATA ENTRY
Name:____________ Date:_________
Signature: ________________

Yoga Weekly Attendance Reporting

Each week attendance is recorded based on sign-in sheets filled out during class. This excel document allows the research coordinator and study team to track weekly attendance, total number of classes attended, and attendance rate (total classes attended/total classes offered). Weekly review of this tracking sheet at team meetings of unblinded study staff allow targeted efforts to help specific participants attain greater attendance rates. Attendance for the maintenance period is tracked in a similar manner.

Participant	Week 1	Week 2	Week 3	Week 4	Week 5	Week 6	Week 7	Week 8	Week 9	Week 10	Week 11	Week 12	Total Classes Attended	Total Attendance Rate
1														
2														
3														
4														
5														
6														
7														
8														
9														
10														
11														
12														
13														
14														
15														
16														
17														
18														
19														
20														
21														
22														
23														
24														
25														

Yoga Observation Form

Observer Name:____________________________ **Date:**____________

Lesson Number/Location	Teacher Being Observed	Assistant Being Observed

Criteria	Notes
Level of preparation and understanding of lesson content	
Clarity of instruction (e.g., respectful and aware of participant needs, building postures from ground up, modifications as needed)	
Taught all postures or sections for specified lesson; adhered to protocol	
Pacing, flow of class, vocal quality	
Rapport and connection with participants (e.g., welcoming, listening, encouraging, available after class)	

Additional Notes:

____
____
____
____
____

Yoga Treatment Fidelity Checklist

Segment 1: “Opening to Something Greater”

Observer Name:_______________________ Date:_______________
Teacher being observed:_______________ Lesson Number:________
Assistant being observed:_______________ Location:_____________

Check in with Participants	3 minutes

Yes No

- ☐ ☐ Asks participants about how their backs are feeling
- ☐ ☐ Discusses any difficulties with home practice; allows opportunity for discussion
- ☐ ☐ Conforms to specified time guidelines

Centering, Yoga Philosophy, and Lesson Introduction	3 minutes

Yes No

- ☐ ☐ Introduces lesson and objective for the day (see above)
- ☐ ☐ Conforms to specified time guidelines

Relaxation	3 minutes

Yes No

- ☐ ☐ Leads participants in relaxation exercise
- ☐ ☐ Conforms to specified time guidelines

Breathing Exercise	4 minutes

Yes No

- ☐ ☐ Leads participants in a breathing exercise that helps them adjust breath
- ☐ ☐ Provides instruction and support to participants throughout the exercise
- ☐ ☐ Does not exceed specified time guidelines

Warm Ups	20 minutes

Check which poses are used for the warm-up (should choose at least three):

Yes No

- ☐ ☐ Knees to Chest
- ☐ ☐ Knees Together Twist
- ☐ ☐ Pelvic Tilt Pose
- ☐ ☐ Cat/Cow Pose
- ☐ ☐ Child’s Pose
- ☐ ☐ Mountain Pose
- ☐ ☐ Modified Chair Pose
- ☐ ☐ Shoulder Opener Pose
- ☐ ☐ Crescent Moon Pose
- ☐ ☐ Chair Twist Pose: Seated

- ☐ ☐ Provides modifications and adjustments for participants as applicable
- ☐ ☐ Does not exceed specified time guidelines

Yoga Treatment Fidelity Checklist

Segment 1: "Opening to Something Greater"

Yoga Postures	25 minutes

Check the box of each pose that is taught:

Yes No

☐ ☐ Wall Dog Pose
☐ ☐ Triangle at Wall Pose
☐ ☐ Chair twists (standing or seated)
☐ ☐ Modified Locust Pose (Both legs, if appropriate)
☐ ☐ Cobra Pose
☐ ☐ Bridge Pose

☐ ☐ Provides modifications and adjustments for participants as applicable
☐ ☐ Does not exceed specified time guidelines

Cool-Downs	10 minutes

Check the box of each pose that is taught:

Yes No

☐ ☐ Supported Bridge Pose
☐ ☐ Knees to Chest
☐ ☐ Extended Leg Stretch
☐ ☐ Knees Together Twist
☐ ☐ Reclining Cobbler Pose

☐ ☐ Provides modifications and adjustments for participants as applicable
☐ ☐ Does not exceed specified time guidelines

Integrative Relaxation	5 minutes

Yes No

☐ ☐ Leads participants in a coordinated breathing and whole body relaxation exercise
☐ ☐ Conforms to specified time guidelines

Closing	2 minutes

Yes No

☐ ☐ Remind participants to practice each day and come to each class with the Weekly Home Practice Log and to fill out their Cost Diaries at home.

© Boston Medical Center

Yoga Treatment Fidelity Checklist

Segment 2: "Listening to the Body"

Observer Name:______________________________ Date:________________
Teacher being observed:_____________________ Lesson Number:__________
Assistant being observed:____________________ Location:_______________

Check in with Participants	3 minutes

Yes No
- ☐ ☐ Asks participants about how their backs are feeling
- ☐ ☐ Discusses any difficulties with home practice; allows opportunity for discussion
- ☐ ☐ Conforms to specified time guidelines

Centering, Yoga Philosophy, and Lesson Introduction	3 minutes

Yes No
- ☐ ☐ Introduces lesson and objective for the day (see above)
- ☐ ☐ Conforms to specified time guidelines

Relaxation	3 minutes

Yes No
- ☐ ☐ Leads participants in relaxation exercise
- ☐ ☐ Conforms to specified time guidelines

Breathing Exercise	4 minutes

Yes No
- ☐ ☐ Leads participants in a breathing exercise that helps them adjust breath
- ☐ ☐ Provides instruction and support to participants throughout the exercise
- ☐ ☐ Does not exceed specified time guidelines

Warm Ups	20 minutes

Check which poses are used for the warm-up (should choose at least three):

Yes No
- ☐ ☐ Knees to Chest
- ☐ ☐ Knees Together Twist
- ☐ ☐ Pelvic Tilt Pose
- ☐ ☐ Cat/Cow Pose
- ☐ ☐ Child's Pose
- ☐ ☐ Mountain Pose
- ☐ ☐ Modified Chair Pose
- ☐ ☐ Shoulder Opener Pose
- ☐ ☐ Crescent Moon Pose
- ☐ ☐ Chair Twist Pose: Seated

- ☐ ☐ Provides modifications and adjustments for participants as applicable
- ☐ ☐ Does not exceed specified time guidelines

Yoga Treatment Fidelity Checklist

Segment 2: "Listening to the Body"

Yoga Postures	25 minutes

Check the box of each pose that is taught:

Yes No
- ☐ ☐ Wall Dog Pose
- ☐ ☐ Triangle at Wall Pose
- ☐ ☐ Standing Forward Bend at Wall
- ☐ ☐ Modified Locust Pose (Both legs, if appropriate)
- ☐ ☐ Sphinx Pose
- ☐ ☐ Cobra Pose
- ☐ ☐ Bridge Pose

- ☐ ☐ Provides modifications and adjustments for participants as applicable
- ☐ ☐ Does not exceed specified time guidelines

Cool-Downs	10 minutes

Check the box of each pose that is taught:

Yes No
- ☐ ☐ Supported Bridge Pose
- ☐ ☐ Knees to Chest
- ☐ ☐ Extended Leg Stretch
- ☐ ☐ Knees Together Twist
- ☐ ☐ Reclining Cobbler Pose

- ☐ ☐ Provides modifications and adjustments for participants as applicable
- ☐ ☐ Does not exceed specified time guidelines

Integrative Relaxation	5 minutes

Yes No
- ☐ ☐ Leads participants in a coordinated breathing and whole body relaxation exercise
- ☐ ☐ Conforms to specified time guidelines

Closing	2 minutes

Yes No
- ☐ ☐ Remind participants to practice each day and come to each class with the Weekly Home Practice Log and to fill out their Cost Diaries at home.

Yoga Treatment Fidelity Checklist

Segment 3: "Engaging Your Power"

Observer Name:_______________________________ Date:________________

Teacher being observed:_____________________ Lesson Number:__________

Assistant being observed:_____________________ Location:_______________

Check in with Participants	3 minutes

Yes No

- ☐ ☐ Asks participants about how their backs are feeling
- ☐ ☐ Discusses any difficulties with home practice; allows opportunity for discussion
- ☐ ☐ Conforms to specified time guidelines

Centering, Yoga Philosophy, and Lesson Introduction	3 minutes

Yes No

- ☐ ☐ Introduces lesson and objective for the day (see above)
- ☐ ☐ Conforms to specified time guidelines

Relaxation	3 minutes

Yes No

- ☐ ☐ Leads participants in relaxation exercise
- ☐ ☐ Conforms to specified time guidelines

Breathing Exercise	4 minutes

Yes No

- ☐ ☐ Leads participants in a breathing exercise that helps them adjust breath
- ☐ ☐ Provides instruction and support to participants throughout the exercise
- ☐ ☐ Does not exceed specified time guidelines

Warm Ups	20 minutes

Check which poses are used for the warm-up (should choose at least three):

Yes No

- ☐ ☐ Knees to Chest
- ☐ ☐ Knees Together Twist
- ☐ ☐ Pelvic Tilt Pose
- ☐ ☐ Toe taps -pelvic tilt with feet off floor
- ☐ ☐ Table Top standing with leg extended, hands on chair seat – lift leg up and down slowly
- ☐ ☐ Cat/Cow Pose
- ☐ ☐ Mountain Pose
- ☐ ☐ Modified Chair Pose
- ☐ ☐ Shoulder Opener Pose
- ☐ ☐ Crescent Moon Pose
- ☐ ☐ Triangle at wall
- ☐ ☐ Shoulder openers

- ☐ ☐ Provides modifications and adjustments for participants as applicable
- ☐ ☐ Does not exceed specified time guidelines

Yoga Treatment Fidelity Checklist

Segment 3: "Engaging Your Power"

Yoga Postures	25 minutes

Check the box of each pose that is taught:

Yes No
- ☐ ☐ Wall Dog Pose
- ☐ ☐ Triangle at Wall Pose
- ☐ ☐ Standing Forward Bend at Wall
- ☐ ☐ Modified Locust Pose (Both legs, if appropriate)
- ☐ ☐ Down dog
- ☐ ☐ Bridge Pose
- ☐ ☐ Child's Pose
- ☐ ☐ Extended leg stretch
- ☐ ☐ Warrior at wall
- ☐ ☐ Seated chair twist

- ☐ ☐ Provides modifications and adjustments for participants as applicable
- ☐ ☐ Does not exceed specified time guidelines

Cool-Downs	10 minutes

Check the box of each pose that is taught:

Yes No
- ☐ ☐ Supported Bridge Pose
- ☐ ☐ Knees to Chest
- ☐ ☐ Extended Leg Stretch
- ☐ ☐ Knees Together Twist
- ☐ ☐ Reclining Cobbler Pose

- ☐ ☐ Provides modifications and adjustments for participants as applicable
- ☐ ☐ Does not exceed specified time guidelines

Integrative Relaxation	5 minutes

Yes No
- ☐ ☐ Leads participants in a coordinated breathing and whole body relaxation exercise
- ☐ ☐ Conforms to specified time guidelines

Closing	2 minutes

Yes No
- ☐ ☐ Remind participants to practice each day and come to each class with the Weekly Home Practice Log and to fill out their Cost Diaries at home.

© Boston Medical Center
Program for Integrative Medicine & Health Care Disparities
Back to Health Trial

Yoga Treatment Fidelity Checklist

Segment 4: "Bringing It Home"

Observer Name:______________________________ Date:________________
Teacher being observed:_____________________ Lesson Number:__________
Assistant being observed:____________________ Location:_______________

Check in with Participants	3 minutes

Yes	No	
☐	☐	Asks participants about how their backs are feeling
☐	☐	Discusses any difficulties with home practice; allows opportunity for discussion
☐	☐	Conforms to specified time guidelines

Centering, Yoga Philosophy, and Lesson Introduction	3 minutes

Yes	No	
☐	☐	Introduces lesson and objective for the day (see above)
☐	☐	Conforms to specified time guidelines

Relaxation	3 minutes

Yes	No	
☐	☐	Leads participants in relaxation exercise
☐	☐	Conforms to specified time guidelines

Breathing Exercise	4 minutes

Yes	No	
☐	☐	Leads participants in a breathing exercise that helps them adjust breath
☐	☐	Provides instruction and support to participants throughout the exercise
☐	☐	Does not exceed specified time guidelines

Warm Ups	20 minutes

Check which poses are used for the warm-up (should choose at least three):

Yes	No	
☐	☐	Knees to Chest
☐	☐	Knees Together Twist
☐	☐	Extended Leg Stretch
☐	☐	Table Top with leg extended
☐	☐	Mountain Pose
☐	☐	Shoulder openers

☐	☐	Provides modifications and adjustments for participants as applicable
☐	☐	Does not exceed specified time guidelines

Yoga Treatment Fidelity Checklist

Segment 4: "Bringing It Home"

Yoga Postures	25 minutes

Check the box of each pose that is taught:

Yes No
- ☐ ☐ Chair Pose
- ☐ ☐ Warrior I
- ☐ ☐ Baby Dancer Pose (using wall modification)
- ☐ ☐ Standing forward bend at wall
- ☐ ☐ Downward facing dog
- ☐ ☐ Locust Pose
- ☐ ☐ Cobra Pose
- ☐ ☐ Child's Pose

- ☐ ☐ Provides modifications and adjustments for participants as applicable
- ☐ ☐ Does not exceed specified time guidelines

Cool-Downs	10 minutes

Check the box of each pose that is taught:

Yes No
- ☐ ☐ Knees to Chest
- ☐ ☐ Reclined Chest Opener
- ☐ ☐ Knees Together Twist
- ☐ ☐ Reclining Cobbler Pose

- ☐ ☐ Provides modifications and adjustments for participants as applicable
- ☐ ☐ Does not exceed specified time guidelines

Integrative Relaxation	5 minutes

Yes No
- ☐ ☐ Leads participants in a coordinated breathing and whole body relaxation exercise
- ☐ ☐ Conforms to specified time guidelines

Closing	2 minutes

Yes No
- ☐ ☐ Remind participants to practice each day and come to each class with the Weekly Home Practice Log and to fill out their Cost Diaries at home.

© Boston Medical Center

Physical Therapy Policies and Procedures

Physical Therapy Treatment Arm Flow Sheet

Step	Forms Needed
**Randomization**	• PT Randomization Phone Call Script
**Pick Up Materials**	• Welcome Packet<br>• Take home materials
**6 Week Data Collection**	• 6-week Questionnaire<br>• Survey Administration Script<br>• Survey Administration DOs and DON'Ts
**12 Week Data Collection**	• 12-week Questionnaire<br>• Survey Administration Script<br>• Survey Administration DOs and DON'Ts
**Maintenance Randomization**	• PT Maintenance Randomization Phone Call Script
**26 Week Data Collection**	• 26-week Questionnaire<br>• Survey Administration Script<br>• Survey Administration DOs and DON'Ts
**40 Week Data Collection**	• 40-week Questionnaire<br>• Survey Administration Script<br>• Survey Administration DOs and DON'Ts
**52 Week Data Collection**	• 52-week Questionnaire<br>• Survey Administration Script<br>• Survey Administration DOs and DON'Ts

END OF STUDY

Physical Therapy Randomization Script

Overview:

Once participants have completed their Baseline Survey, they will receive their treatment assignment by an unblinded staff member at least 72 hours before the start of their study cohort.

Suggested Script:

Hello, this is __________ with the Back to Health study. Is ____________ available?

If yes:

May I speak with to ______? Hello _______. I'm **[give name]** *from Back to Health study team. I'm calling you let you know that you have been randomly selected to receive physical therapy as your treatment. This means that we ask you to attend physical therapy appointments 15 times over the course of the next 12 weeks. Your physical therapy will take place the week of* **[week]** *starting with* **[start date].** *Your physical therapy will be held at* **[location]**. *We will be sending you a letter in the mail with this same information.*

If no:

When is the best time for me to call ____________ back?

(It is best if we call them, rather than the participant calling us back so that there isn't any confusion about the message being delivered)

Physical Therapy Survey Reminder Script

Overview:

Participants will be called 72 hours before the start of a data collection week to schedule survey administration. Participants will we asked to attend group survey administration sessions; however they may do the survey over the phone if their schedule does not allow them to complete the survey in person.

Suggested Script:

Hello, this is __________ with Back to Health study. Is ____________ available?

If no:

Do you know what time I could reach him/her? Thank you.

If yes:

May I speak with to ______? Hello _______. This is a reminder from Back to Health study at Boston Medical Center that you have physical therapy on **[day of week and date]** *at* **[time and location].**

This week, we will be collecting data for the study. This will take place at preset times. You can do your survey at times before your physical therapy session or 24 hours after your session. If you cannot come in person, you can do your survey over the phone. Which option would be most convenient for you?

If in-person*:*

Okay, we will meet you on **[day of week and date]** *at* **[time and location].**

If over the phone:

Okay, when would you like us to call you to complete the survey? We are available Monday through Saturday from 9:00 am to 7:00 pm.

Do you have any questions? Thank you.

© Boston Medical Center

Physical Therapy Maintenance Randomization Script

Overview:

Maintenance randomization phone calls will be conducted by unblinded study staff only. This call will be made at least one week prior to the start of the maintenance phase.

Suggested Script:

Maintenance Group:

Hello, this is __________ with the Back to Health study. Is ____________ available?

If yes:

> *May I speak with to ______? Hello _______. I'm* **[give name]** *from Back to Health study team. I'm calling you let you know that you have been randomly selected to be in the maintenance group. This means that we ask you to attend an additional five physical therapy sessions over the course of the next nine months. Your physical therapy sessions will take place the week of* **[week]** *starting with* **[start date].** *Your physical therapy appointment will be held at* **[location]**. *We will be sending you a letter in the mail with this same information.*

If no:

> *When is the best time for me to call ____________ back?*

No Maintenance Group:

Hello, this is __________ with Back to Health study. Is ____________ available?

If yes:

> *May I speak with to ______? Hello _______. I'm* **[give name]** *from Back to Health study team. I'm calling you let you know that you have been randomly selected to be in the home practice group. This means that we ask you to continue practicing physical therapy exercises at home each day. We'll call you to check in and see how everything is going. Please feel free to call us if you have any questions or if you experience any adverse events.*

If no:

> *When is the best time for me to call ____________ back?*
>
> (It is best if we call them, rather than the participant calling us back so that there isn't any confusion about the message being delivered.)

© Boston Medical Center

Physical Therapy Adverse Event Call Script

Responsibility

Adverse events will be collected by unblinded study staff. Study staff will alert the PI and the Research Fellow (Adverse Events) of any adverse events within 24 hours.

Suggested Script

Hello, this is __________ with Back to Health study. Is ____________ available?

If yes:

> *May I speak to _______? Hello _______. I'm* **[give name]** *from Back to Health study, how are you? I'm calling to check-in and see how you're doing with your physical therapy. Have you had any problems or injuries you think you experienced from your participation in physical therapy?*
>
> If yes:
>
> > *When did this problem or injury begin?*
> >
> > *How soon after your physical therapy appointment did this problem or injury begin or did this happen during your physical therapy appointment?*
>
> If participant experienced adverse event, complete an Adverse Event Form.
>
> *If you need anything, please don't hesitate to call us. Have a nice day!*

If no:

Do you know what time I could reach him/her? Thank you.

© Boston Medical Center

Physical Therapy Home Practice Call Script

Responsibility

Unblinded study staff will call physical therapy participants that were randomized to the home treatment group of the maintenance phase.

Suggested Script

Hello, this is __________ with Back to Health study. Is ____________ available?

If yes:

> *May I speak with _______? Hello _______. I'm* **[give name]** *from the Back to Health study, how are you? I'm calling to check-in and see how you're doing with your physical therapy exercises.*
>
> *How many hours have you done physical therapy exercises at home in the last three weeks?*
>
> *If you need anything, please don't hesitate to call us. Have a nice day!*

If no:

> *Do you know what time I could reach him/her? Thank you.*

Weekly Home Practice Log: Extension Exercises

First Name and Last Initial ONLY: ______________________________

Please let us know when and for how long you did physical therapy exercises at home since your last appointment.

**Date:**	**Prone Lying** 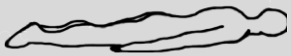	**Prone Lying on Elbows** 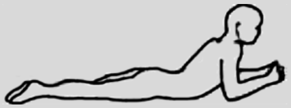	**Prone Press-ups** 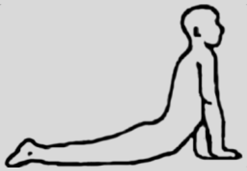	**Progressive Extension with Pillows** 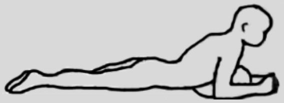	**Standing Extension** 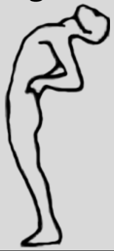
________	☐Yes ☐No<br># reps_______	☐Yes ☐No<br># reps_______	☐Yes ☐No<br># reps_______	☐Yes ☐No<br># reps_______	☐Yes ☐No<br># reps_______
________	☐Yes ☐No<br># reps_______	☐Yes ☐No<br># reps_______	☐Yes ☐No<br># reps_______	☐Yes ☐No<br># reps_______	☐Yes ☐No<br># reps_______
________	☐Yes ☐No<br># reps_______	☐Yes ☐No<br># reps_______	☐Yes ☐No<br># reps_______	☐Yes ☐No<br># reps_______	☐Yes ☐No<br># reps_______
________	☐Yes ☐No<br># reps_______	☐Yes ☐No<br># reps_______	☐Yes ☐No<br># reps_______	☐Yes ☐No<br># reps_______	☐Yes ☐No<br># reps_______
________	☐Yes ☐No<br># reps_______	☐Yes ☐No<br># reps_______	☐Yes ☐No<br># reps______	☐Yes ☐No<br># reps_______	☐Yes ☐No<br># reps_______
________	☐Yes ☐No<br># reps_______	☐Yes ☐No<br># reps_______	☐Yes ☐No<br># reps______	☐Yes ☐No<br># reps_______	☐Yes ☐No<br># reps_______
________	☐Yes ☐No<br># reps_______	☐Yes ☐No<br># reps_______	☐Yes ☐No<br># reps______	☐Yes ☐No<br># reps_______	☐Yes ☐No<br># reps_______

Comments/Notes:

© Boston Medical Center

Weekly Home Practice Log: Flexion Exercises

First Name and Last Initial ONLY: _____________________

Please let us know when and for how long you did physical therapy exercises at home since your last appointment.

**Date:**	**Pelvic tilt** 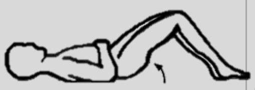	**Single Knee to chest** 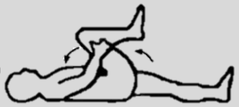	**Double knee to chest** 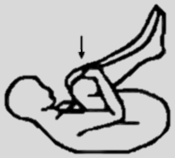	**Partial sit-up** 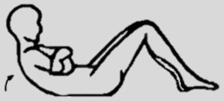	**Hamstring stretch** 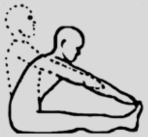	**Hip Flexor stretch** 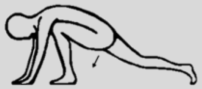	**Squat** 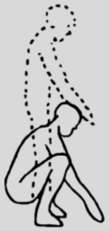
________	☐Yes ☐No # reps_______	☐Yes ☐No # reps_______	☐Yes ☐No # reps_______	☐Yes ☐No # reps_______	☐Yes ☐No # reps_______	☐Yes ☐No # reps_______	☐Yes ☐No # reps_______
________	☐Yes ☐No # reps_______	☐Yes ☐No # reps_______	☐Yes ☐No # reps_______	☐Yes ☐No # reps_______	☐Yes ☐No # reps_______	☐Yes ☐No # reps_______	☐Yes ☐No # reps_______
________	☐Yes ☐No # reps_______	☐Yes ☐No # reps_______	☐Yes ☐No # reps_______	☐Yes ☐No # reps_______	☐Yes ☐No # reps_______	☐Yes ☐No # reps_______	☐Yes ☐No # reps_______
________	☐Yes ☐No # reps_______	☐Yes ☐No # reps_______	☐Yes ☐No # reps_______	☐Yes ☐No # reps_______	☐Yes ☐No # reps_______	☐Yes ☐No # reps_______	☐Yes ☐No # reps_______
________	☐Yes ☐No # reps_______	☐Yes ☐No # reps_______	☐Yes ☐No # reps______	☐Yes ☐No # reps_______	☐Yes ☐No # reps_______	☐Yes ☐No # reps_______	☐Yes ☐No # reps_______
________	☐Yes ☐No # reps_______	☐Yes ☐No # reps_______	☐Yes ☐No # reps______	☐Yes ☐No # reps_______	☐Yes ☐No # reps_______	☐Yes ☐No # reps_______	☐Yes ☐No # reps_______
________	☐Yes ☐No # reps_______	☐Yes ☐No # reps_______	☐Yes ☐No # reps______	☐Yes ☐No # reps_______	☐Yes ☐No # reps_______	☐Yes ☐No # reps_______	☐Yes ☐No # reps_______

Comments/Notes:

© Boston Medical Center
Program for Integrative Medicine & Health Care Disparities
Back to Health Trial

Back to Health
A Study Comparing Yoga, Physical Therapy & Education for Chronic Low Back Pain

Weekly Home Practice Log: Stabilization Exercises

First Name and Last Initial ONLY: ______________________________

Please let us know when and for how long you did physical therapy exercises at home since your last appointment.

**Date:**	**Abdominal Bracing**<br>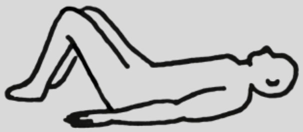<br>(8 second hold)	**Abdominal Bracing w/ Heel Slide**<br>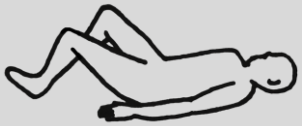<br>(6 second hold)	**Abdominal Bracing w/ Leg Lifts**<br>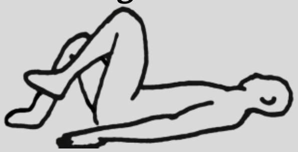<br>(6 second hold)	**Abdominal Bracing w/ Bridging**<br>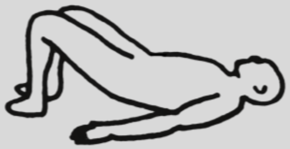<br>(8 second hold)	**Abdominal Bracing w/ Single Leg Bridging***<br>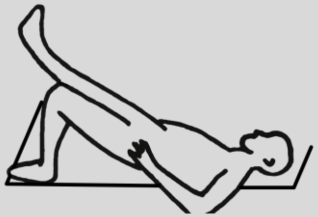<br>(8 second hold)
______	☐Yes ☐No<br># reps________/30	☐Yes ☐No<br># reps________/20	☐Yes ☐No<br># reps________/20	☐Yes ☐No<br># reps________/30	☐Yes ☐No<br># reps________/30
______	☐Yes ☐No<br># reps________/30	☐Yes ☐No<br># reps________/20	☐Yes ☐No<br># reps________/20	☐Yes ☐No<br># reps________/30	☐Yes ☐No<br># reps________/30
______	☐Yes ☐No<br># reps________/30	☐Yes ☐No<br># reps________/20	☐Yes ☐No<br># reps________/20	☐Yes ☐No<br># reps________/30	☐Yes ☐No<br># reps________/30
______	☐Yes ☐No<br># reps________/30	☐Yes ☐No<br># reps________/20	☐Yes ☐No<br># reps________/20	☐Yes ☐No<br># reps________/30	☐Yes ☐No<br># reps________/30
______	☐Yes ☐No<br># reps________/30	☐Yes ☐No<br># reps________/20	☐Yes ☐No<br># reps________/20	☐Yes ☐No<br># reps________/30	☐Yes ☐No<br># reps________/30
______	☐Yes ☐No<br># reps________/30	☐Yes ☐No<br># reps________/20	☐Yes ☐No<br># reps________/20	☐Yes ☐No<br># reps________/30	☐Yes ☐No<br># reps________/30
______	☐Yes ☐No<br># reps________/30	☐Yes ☐No<br># reps________/20	☐Yes ☐No<br># reps________/20	☐Yes ☐No<br># reps________/30	☐Yes ☐No<br># reps________/30

Comments/Notes:

© Boston Medical Center
Program for Integrative Medicine & Health Care Disparities
Back to Health Trial

First Name and Last Initial ONLY: ____________________________

Please let us know when and for how long you did physical therapy exercises at home since your last appointment.

**Date:**	**Abdominal Bracing while Standing**<br>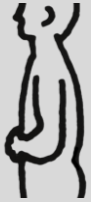<br>(8 second hold)	**Bracing w/ Standing Row**<br>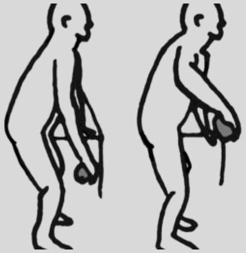<br>(6 second hold)	**Quadruped Arm Lifts w/ Abdominal Bracing**<br>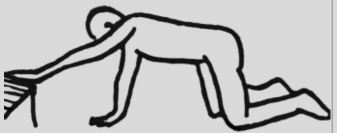<br>(8 second hold)	**Quadruped Leg Lifts w/ Abdominal Bracing**<br>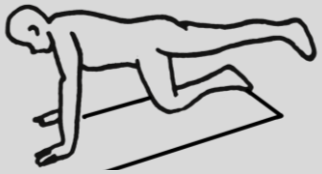<br>(8 second hold)
________	☐Yes ☐No<br># reps________/30	☐Yes ☐No<br># reps________/20	☐Yes ☐No<br># reps________/30	☐Yes ☐No<br># reps________/30
________	☐Yes ☐No<br># reps________/30	☐Yes ☐No<br># reps________/20	☐Yes ☐No<br># reps________/30	☐Yes ☐No<br># reps________/30
________	☐Yes ☐No<br># reps________/30	☐Yes ☐No<br># reps________/20	☐Yes ☐No<br># reps________/30	☐Yes ☐No<br># reps________/30
________	☐Yes ☐No<br># reps________/30	☐Yes ☐No<br># reps________/20	☐Yes ☐No<br># reps________/30	☐Yes ☐No<br># reps________/30
________	☐Yes ☐No<br># reps________/30	☐Yes ☐No<br># reps________/20	☐Yes ☐No<br># reps________/30	☐Yes ☐No<br># reps________/30
________	☐Yes ☐No<br># reps________/30	☐Yes ☐No<br># reps________/20	☐Yes ☐No<br># reps________/30	☐Yes ☐No<br># reps________/30
________	☐Yes ☐No<br># reps________/30	☐Yes ☐No<br># reps________/20	☐Yes ☐No<br># reps________/30	☐Yes ☐No<br># reps________/30

Comments/Notes:

© Boston Medical Center
Program for Integrative Medicine & Health Care Disparities
Back to Health Trial

Weekly Home Practice Log: Stabilization Exercises

First Name and Last Initial ONLY: ______________________________

Please let us know when and for how long you did physical therapy exercises at home since your last appointment.

**Date:**	**Quadruped Opposite Arm and Leg Lift w/ Abdominal Bracing**<br>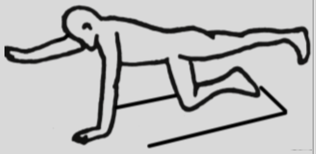<br>(8 second hold)	**Side Support w/ Knees Flexed and Abdominal Bracing**<br>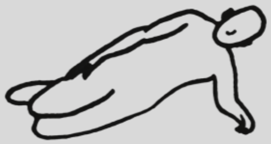<br>(8 second hold)	**Side Support w/ Knees Extended and Abdominal Bracing**<br>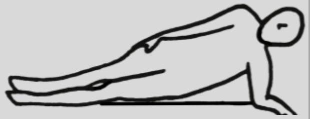<br>(8 second hold)	**Advanced Side Bridge**<br>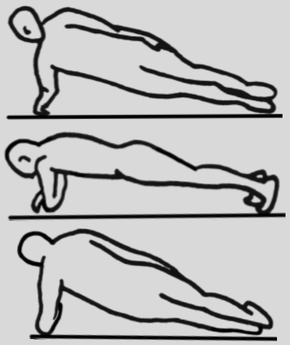
________	☐Yes ☐No<br># reps_______/30	☐Yes ☐No<br># reps_______/30	☐Yes ☐No<br># reps_______/30	☐Yes ☐No<br># reps_______/30
________	☐Yes ☐No<br># reps_______/30	☐Yes ☐No<br># reps_______/30	☐Yes ☐No<br># reps_______/30	☐Yes ☐No<br># reps_______/30
________	☐Yes ☐No<br># reps_______/30	☐Yes ☐No<br># reps_______/30	☐Yes ☐No<br># reps_______/30	☐Yes ☐No<br># reps_______/30
________	☐Yes ☐No<br># reps_______/30	☐Yes ☐No<br># reps_______/30	☐Yes ☐No<br># reps_______/30	☐Yes ☐No<br># reps_______/30
________	☐Yes ☐No<br># reps_______/30	☐Yes ☐No<br># reps_______/30	☐Yes ☐No<br># reps_______/30	☐Yes ☐No<br># reps_______/30
________	☐Yes ☐No<br># reps_______/30	☐Yes ☐No<br># reps_______/30	☐Yes ☐No<br># reps_______/30	☐Yes ☐No<br># reps_______/30
________	☐Yes ☐No<br># reps_______/30	☐Yes ☐No<br># reps_______/30	☐Yes ☐No<br># reps_______/30	☐Yes ☐No<br># reps_______/30

Comments/Notes:

© Boston Medical Center
Program for Integrative Medicine & Health Care Disparities
Back to Health Trial

Physical Therapy Weekly Attendance Reporting

Each week attendance is recorded based on completed patient visits at the PT clinic. This excel document allows the research coordinator and study team to track weekly attendance, total number of classes attended, and attendance rate (total classes attended/total classes offered). Weekly review of this tracking sheet at team meetings of unblinded study staff allow targeted efforts to help specific participants attain greater attendance rates. Attendance for the maintenance period is tracked in a similar manner.

Participant	Week 1	Week 2	Week 3	Week 4	Week 5	Week 6	Week 7	Week 8	Week 9	Week 10	Week 11	Week 12	Total Classes Attended	Total Attendance Rate
1														
2														
3														
4														
5														
6														
7														
8														
9														
10														
11														
12														
13														
14														
15														
16														
17														
18														
19														
20														
21														
22														
23														
24														
25														

© Boston Medical Center

Education Policies and Procedures

Education Treatment Arm
Flow Sheet

Step	Forms Needed
Randomization	• Education Randomization Script
Pick Up Materials	• Welcome Packet<br>• Take home supplies
3 Week Check-In	• Education Check-In Phone Call Script
6 Week Data Collection	• 6-week Questionnaire<br>• Survey Administration Script<br>• Survey Administration DOs and DON'Ts
9 Week Check-In	• Education Check-In Phone Call Script
12 Week Data Collection	• 12-week Questionnaire<br>• Survey Administration Script<br>• Survey Administration DOs and DON'Ts
26 Week Data Collection	• 26-week Questionnaire<br>• Survey Administration Script<br>• Survey Administration DOs and DON'Ts
40 Week Data Collection	• 40-week Questionnaire<br>• Survey Administration Script<br>• Survey Administration DOs and DON'Ts
52 Week Data Collection	• 52-week Questionnaire<br>• Survey Administration Script<br>• Survey Administration DOs and DON'Ts

END OF STUDY

© Boston Medical Center

Education Randomization Script

Overview:

Randomization phone calls will be conducted by unblinded study staff only. This call will be made at least one week prior to study start.

Suggested Script:

Hello, this is __________ with Back to Health study. Is ____________ available?

If yes:

May I speak with to ______? Hello _______. I'm **[name]** *from Back to Health study. I'm calling you let you know that you have been randomly selected to be in the education part of the study. You will receive The Back Pain Helpbook, which will give you strategies for back pain care. During the study, you should read the book, practice the recommended exercises, and follow the recommendations in each section. When you come to Boston Medical Center to pick up your book, we will go over this information with you. When are you available to pick up your book?*

I will meet you by the information desk by the emergency room. See you on **[date]** *at* **[time]**, *have a nice day!*

If no:

Do you know when I can call back to reach him/her? Thank you!

Education Survey Reminder Call Script

Overview:

Participants will be called 72 hours before the start of a data collection week to schedule survey administration. Participants will we asked to attend group survey administration sessions; however they may do the survey over the phone if their schedule does not allow them to complete the survey in person.

Suggested Script:

Hello, this is ___________ with Back to Health study. Is _____________ available?

If no:

Do you know what time I could reach him/her? Thank you.

If yes:

This week, we will be collecting data for the study. This will take place at preset times. If you cannot come in person, you can do your survey over the phone. Which option would be most convenient for you?

If in-person:

Okay, we will meet you on **[day of week and date]** *at* **[time and location].**

If over the phone:

Okay, when would you like us to call you to complete the survey? We are available Monday through Saturday from 9:00am to 7:00pm.

Do you have any questions? Thank you.

Education Check-in Phone Call Script

Overview:

During the treatment phase, an unblinded study staff member will call education participants to check-in every 3 weeks. During the maintenance phase, an unblinded study staff member will call participants every 6 weeks.

Suggested Script:

Hello, this is ______ with Back to Health study. Is ________ available?

If yes:

Hello _______. I'm **[name]** *from Back to Health study, how are you? I'm calling to check-in and see how you're doing with managing your low back pain.*

How is it going with the Back Pain Helpbook?

What chapters have you read so far?

Do you have any other questions for me today?

If you need anything, don't hesitate to call us. Have a nice day!

If no:

Do you know the best time for me to call back to reach him/her? Thank you!

If voicemail:

Hello ______. This is **[name]** *calling from Back to Health study. I was calling to check in and see how you're doing with managing your low back pain and using The Back Pain Helpbook. I'd love to hear back from you, so please call me back at [phone number] when you have some time. Have a nice day!*

© Boston Medical Center

Education Adverse Events Call Script

Responsibility

Adverse events will be collected by unblinded study staff. Study staff will alert the PI and the Research Fellow (Adverse Events) of any adverse events within 24 hours.

Suggested Script

Hello, this is __________ with Back to Health study. Is ____________ available?

If yes:

May I speak to _______? Hello _______. I'm **[name]** *from Back to Health study, how are you? I'm calling to check-in and see how you're doing with your yoga classes. Have you had any problems or injuries you think you experienced from your participation in our study?*

If yes:

When did this problem or injury begin?

If participant experienced adverse event, complete an Adverse Event Form.

If you need anything, please don't hesitate to call us. Have a nice day!

If no:

Do you know what time I could reach him/her? Thank you.

Health Education Assignments

We ask that you read *The Back Pain Helpbook* over the next 12 weeks. **Every 3 weeks** a member of our study staff will call you to check in and see how you are doing with the book. To help you, we've divided the book into four sections and we suggest you read the following chapters within the times mentioned below:

Weeks 1-3

- Read and complete the **Self-Assessment in Part 1, and Chapters 2-3** (Pages 3-43)

These two sections of The Back Pain Help Book are intended to introduce you to back pain self-care and increase your understanding on the problem of back pain.

Weeks 4-6

- Read **Chapters 4-11** (pages 47-110)

This section focuses on how best to manage your back pain and how you can help to ease your back pain.

Weeks 7-9

- Read **Chapters 12-17** (pages 113-174)

This section teaches you simple exercises and activities that can be done to reduce your back pain.

Weeks 10-12

- Read **Chapters 18-22** (pages 177-213)

This part of your book talks about how to increase your overall enjoyment of life.

Please note: You may read ahead of the above mentioned times if you wish to do so. This sheet is meant to serve as a guide in completing the assignment within the study period of 12 weeks.

© Boston Medical Center

Back to Health

Data Collection and Management Policies and Procedures

Data Collection Flow Sheet

Forms Needed

Telephone Eligibility Screening
- Written Verbal Consent
- In-Person Visit Schedule

↓

Eligibility Checklist
- Eligibility Checklist Form

↓

Baseline Survey
- Corresponding Week Questionnaire
- Survey Administration Script
- Survey Administration DOs and DON'Ts

↓

Randomization
- Appropriate Randomization Phone Call Script

↓

6 Week Survey

↓

12 Week Survey

↓

26 Week Survey

↓

40 Week Survey

↓

52 Week Survey

(6 to 52 Week Surveys:)
- Corresponding Week Questionnaire
- Survey Administration Script
- Survey Administration DOs and DON'Ts

END OF STUDY

© Boston Medical Center

Staff Blinding and Responsibilities

Blinding Overview

Activity	Blinded Staff	Un-blinded Staff
Eligibility Screening	✓	✓
Informed Consent	✓	✓
Baseline Survey Administration	✓	
Randomization Period		
Preparing Reminder Call Lists		✓
Reminder Calls/Attendance Tracking		✓
Schedule Paper Survey Administration		✓
Survey Administration (Phone)	✓	
Survey Administration (Paper)	✓	
Data Entry (Paper)	✓	
Double Data Entry	✓	
Home Practice Data Entry		✓
Adverse Event Reporting		✓

Blinded Staff:

- Biostatistician
- Data Analyst
- Recruitment/Data Collection Team Members
- Research Fellow

Unblinded Staff:

- Principal Investigator
- Research Coordinator
- Senior Research Assistant
- Data Research Assistant(s)
- Treatment Arm Coordinators
- Research Fellow (Adverse Events)

© Boston Medical Center

General Data Collection Guidelines

Treatment Phase Data Collection

- Paper surveys will be administered one hour prior to treatment at preset times in private rooms at a health center site or Boston Medical Center during data collection weeks, proctored by blinded staff.
- Telephone survey formats may be administered 24 hours after the participant's treatment, if necessary.

Maintenance Phase (Yoga + PT)

- Same procedure used as during the intervention phase.

Additional Data Collection for PT Arm

- In addition to all scheduled survey times, all physical therapy group participants will complete the Modified Oswestry and Fear Avoidance Belief Questionnaire every 6 weeks throughout the study. Additional time points include **18, 32, 46** weeks.
- During these 3 additional weeks, the Physical Therapy Arm coordinator and one unblinded RA will administer these surveys over the phone.

Additional Data Collection for Education Arm

- In addition to all scheduled survey times, all education group participants will receive check-in calls every 3 weeks during the 12 week intervention phase and every 6 weeks during the maintenance phase. Adherence to reading assignments will be assessed at this time.
- During these additional weeks, the Physical Therapy Arm coordinator and one unblinded RA will administer these surveys over the phone.

Randomization Procedure

Timeframe

- After all participants in a cohort have completed baseline survey, they will be randomized using permuted block randomization into one of three groups (*with block sizes of 5, 10, and 15*) in a 2:2:1 ratio.
  - Yoga
  - Physical Therapy
  - Education
- Participants will be notified of their treatment conditions (Yoga, PT, Education) at least 1 week before the start of their first appointment.
- 2-3 weeks prior to the end of the intervention phase, the Biostatistician will randomize participants in the Yoga and Physical Therapy groups into either the Maintenance or non-Maintenance phase in a 1:1 ratio.
- After the 12 week-intervention phase is complete, participants in the Yoga and Physical Therapy groups will be notified of whether or not they are in either the Yoga Maintenance or Physical Therapy Maintenance groups by unblinded research staff.

Responsibility

- Data RA 1 will complete randomization on StudyTrax and create a password protected spreadsheet with ID numbers and treatment conditions.
- All other research staff will be denied access on StudyTrax to treatment condition information.
- Only unblinded staff will notify participants of their treatment conditions by phone.

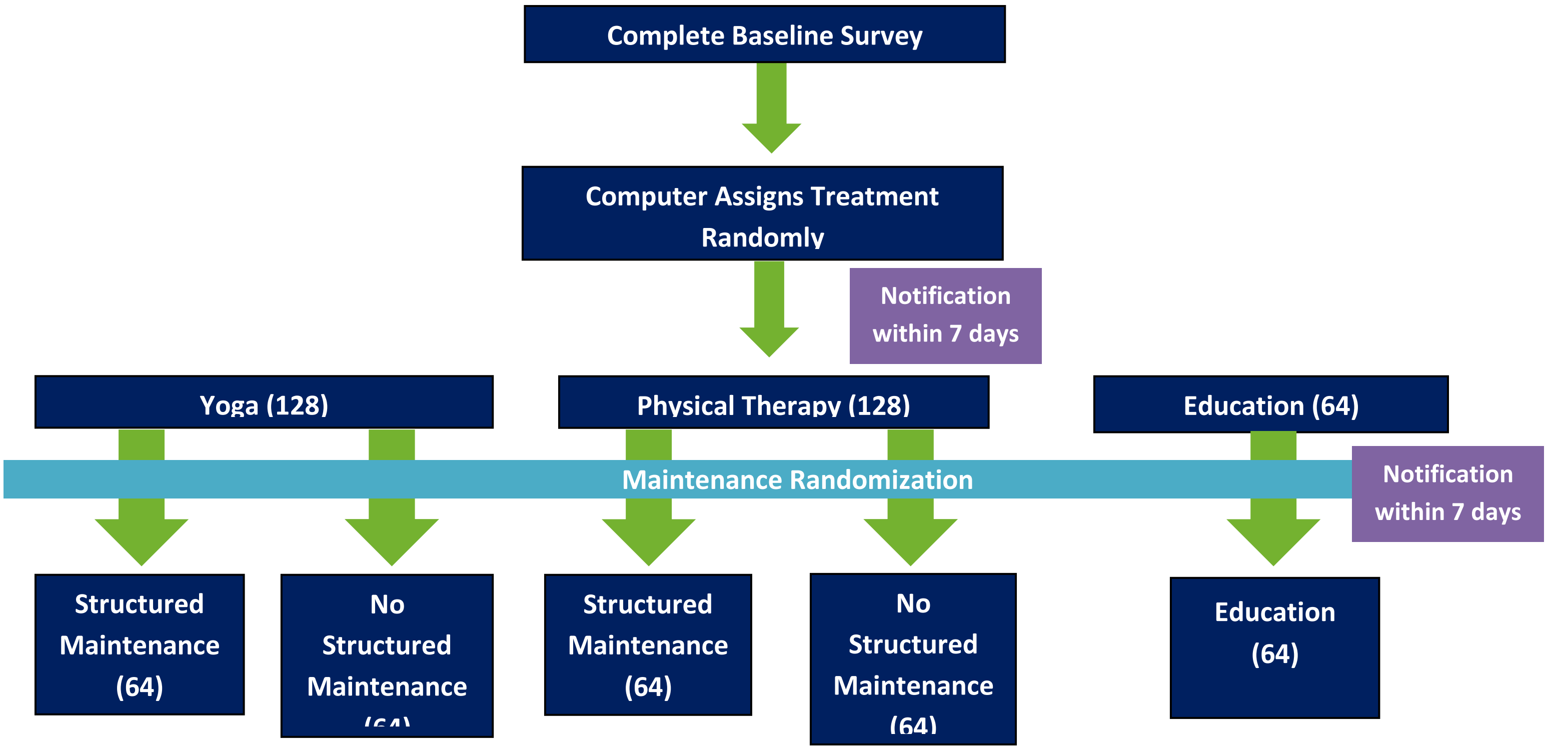

Randomization and Enrollment

Study ID#:______________________

Eligibility Verification

Date: ____/____/____

Is the participant eligible for the study based on Inclusion and Exclusion criteria?

☐Yes ☐No

If no please leave the rest of the form blank

If Yes…

1. Date Enrolled

Met all Eligibility Criteria on… Date: ____/____/____

Signature of Research Staff: ____

2. Date of Randomization

If different from date enrolled… Date: ____/____/____

Signature of Research Staff: ____

3. Not Randomized

If eligible and not randomized please indicate reason:

☐Failed to return ☐Declined participation ☐Other (specify):

Signature of Research Staff: ____

© Boston Medical Center

Survey Administration Procedures

Staff: A blinded and unblinded member of the study staff must be present

Supplies:

- Attendance sheet
- Change of contact info forms
- Gift card/survey administration schedule
- Surveys
- Lock box & pens

Opening:

Hello, I'm _____________________ from the Back to Health study. We would first like to thank all of your for joining us here today. We would like to better understand how well you are able to do your usual activities and how you rate your own health. To help us better understand these things we like to collect some information from you. This survey should take about an hour to complete.

This is not a test and there are no right or wrong answers. Choose the response that best represents the way you feel. Please answer each question as best as you can. Please remember that your answers are confidential.

Please don't tell us if you are in the yoga, physical therapy, or education group. Please ask [unblinded staff name] if you have any questions about the survey or need to reschedule your class. Thank you!

Participant Arrival:

1. As participants arrive, greet them with opening speech.
2. After opening, participants will check in with unblinded study staff who will check their name off of master attendance list and give them a survey.
3. Staff will direct participants to take a seat and begin survey. Re-emphasize that questions should be directed toward unblinded staff member.
4. If necessary, repeat opening speech on individual basis for late arrivers.

Survey Collection:

1. Flip through survey to ensure completeness
2. Staff who administered survey will print, sign, and date the survey
3. Staff will check box if a potential adverse event was identified
4. Place survey in lock box
5. Ask participant if they have any change of contact information and collect as applicable

Survey Administration Procedures

Task	Blinded Staff	Unblinded Staff
Print master attendance list		X
Check off participants from master attendance list		X
Distribute surveys		X
Administer surveys	X	
Answer survey related questions	X	
Collect surveys	X	

General Guidelines

DOs:

- Be warm and friendly
- Ask and encourage participants to complete the entire survey
- Repeat questions and answer choices verbatim
- Tell participants to answer questions based on what they think each item means

DON'Ts:

- Discuss the participant's health or emotions prior to administering the survey
- Accept incomplete surveys without first encouraging participants to respond to unanswered questions
- Change the wording or response choice of any question
- Interpret or explain questions

Common Problems and Issues

What should I do if the participant asks for clarification of a question?

- Assist the participant by rereading the question verbatim.
- If the participant asks what something means, do not offer an explanation; rather, suggest that he/she use their own interpretation of the question.
- Suggested response: "*I know that it may be hard to think this way, but which of these categories most closely expresses what you are thinking or feeling?*"
- Record participant's issue/question

© Boston Medical Center

What should I do if the participant wants to know what their answers mean?

- Simply state, "*I'm sorry, I am not trained to score or interpret the survey.*"
- For Eligibility Screening, "*The purpose of this survey is to determine your eligibility. Our system only lets us know if a potential participant is ineligible, we are not trained to interpret your answers.*"
- For other surveys, "*I'm sorry, I am not trained to score or interpret surveys.*"

What if the participant refuses to complete a survey?

- Explain that completing the survey is required in order to participate in the study.
- Explain that information that the survey provides are as important as any other type of medical information.
- Explain that they survey responses are essential to getting a complete picture of the participant's health.
- If the participant still refuses, thank them for their time and end the call.

What if the participant doesn't answer all of the questions?

- Ask the participant why he/she had difficulty responding.
- Reread the question verbatim. Do not rephrase the question.
- If the participant is still unable to answer the question, make note and accept the incomplete survey.

Date: _______________
Study ID: ____________

Survey Signature Page

For Staff ONLY

Survey Administration:

_________________________ _____________________________ _____/_____/____
Printed Name Signature Date

Initial Data Entry:

_________________________ _____________________________ _____/_____/____
Printed Name Signature Date

Double Data Entry:

_________________________ _____________________________ _____/_____/____
Printed Name Signature Date

Data Audit:

_________________________ _____________________________ _____/_____/____
Printed Name Signature Date

STAFF

Data Entry Procedure

Data Entry (Primary Data Entry)

Paper

- Paper surveys will be entered by blinded research staff.
- Data will be entered in StudyTrax within a week of receiving the paper surveys.
- After the survey is completely entered and saved, click on 'Edit Subject' and locate the 'Data Entry Sign-in' section. Write your initials and the date/time you completed data entry in the appropriate fields.
- Once completed, transfer participant folder from 'To Be Entered' into 'Primary Data Entry'.

Telephone

- Telephone data is entered in real time by blinded research staff.
- Refer to "Telephone Survey Procedures" for guidelines.

Double Data Entry (Secondary Data Entry)

- Double Data Entry will be completed for all paper surveys by blinded research staff* after the completion of Primary Data Entry.
  **Staff can enter data for one round of data entry (either Primary OR Secondary, and never both).*
- To access the Double Data Entry Forms
  1. Locate the participant by searching for their ID number on StudyTrax.
  2. Click on 'Encounters' and select the 'New' under the *Secondary Date* column for the appropriate time interval.
- Once completed, transfer participant folder from 'Primary Data Entry' to 'Secondary Data Entry.

General Tips

- Attempt to answer every field (e.g. Pain Medication). Use the codes below if any answers are missing. Note that the code depends on whether or not the variable requires a numeric response or a text response.
- If an item is single response and multiple responses are entered (i.e. both yes and no are checked off), treat as missing value. Out of range values should also be treated as missing.
- Record ID# and specific question of any responses that do not fall into the above categories.
- SAVE often.

Missing Values	Numeric	Text
Not Applicable/Never	-99	"NA"
Don't Know or left Blank for *Free Response*	-88	"REF"

Paper Survey Procedures

- Unblinded research team members are responsible for scheduling participants for paper surveys and administering paper surveys.

- Blinded research team members are responsible for proctoring paper survey administration.

- *Administration*
  1. Prepare paper surveys with ID numbers and date of administration written in on the top right corner of all pages of the forms
  2. Make sure to bring extra copies of the forms in case unexpected participant arrives
  3. Once a participant completes a survey, review the form to assure that each question is answered. If he/she has not answered a question:
     a. Ask the participant why he/she had difficulty responding
     b. Reread the question verbatim. Do not rephrase the question
     c. If the participant is still unable to answer the question, make note and accept the incomplete survey

- *Collection and Storage*
  1. Transport all surveys in lock box and file in the B2H office.
  2. The Survey drawer will be divided into 4 sections by cohort. Each Cohort section will be divided into 3 sections: 'To Be Entered', 'Primary Data Entry', and 'Secondary Data Entry'.
  3. Each participant will have their own individualized folder labeled with only their ID number and a checklist of all surveys contained therein.
  4. Place collected surveys into appropriate individual folders, check off and date which survey had been collected, and file into the 'To Be Entered' section.

- *Confidentiality*
  1. All paper surveys are identifiable by ID number and are to be stored in individual folders. Names linked to ID numbers are restricted to unblinded staff only.
  2. After the completion of a cohort, all paper surveys will be moved into a locked file cabinet in the Principal Investigators office.
  3. Paper surveys will be shredded 3 years after the completion of the study.

Telephone Survey Procedures

- Blinded research team members are responsible for calling participants to complete telephone surveys.

- *Administration*
  1. Search for the participant on StudyTrax by ID number to reach the 'Subject Overview' page.
  2. Locate the correct data collection time point and select.
  3. Complete each section, saving after each section.
  4. After the survey is complete and saved, click on 'Edit Subject' and locate the 'Data Entry Sign-in' section. Write your initials and the date/time you completed data entry in the appropriate fields.

- *Collection and Storage*
  1. All survey data are captured in real time and stored on the StudyTrax server, which is backed up nightly.

- *Confidentiality*
  1. All information on StudyTrax is password protected and each user is assigned a unique user ID and password with restricted rights.
  2. Data will be periodically exported into Excel for analysis purposes. Documents will be password protected and will never contain participant names and IDs at the same time.

Verification of Eligibility Checklist

Suggested Script:

Since it has been some time since you answered our questions about whether you are eligible to be in the study, I want to ask you a few quick questions before we begin your Baseline Survey.

Checklist:

1. In the past 3 months, have you had a period of two weeks or longer during which you had no low back pain?
   - ☐ Yes
   - ☐ No
   - ☐ Don't know/Refuse

2. In the past week how often have you had low back pain?
   - ☐ Not at all
   - ☐ Less than half the time
   - ☐ Half the time
   - ☐ More than half the time
   - ☐ All the time
   - ☐ Don't know/Refuse

3. Pick a number between 0 and 10 that best describes your low back pain in the last 7 days, where 0 is no pain and 10 is the worst possible pain. ________

4. In the next 12 months, are you planning to move out of the area or have a significant change in your schedule which would make it impossible to participate in the study?
   - ☐ Yes
   - ☐ No
   - ☐ Don't know/Refuse

5. ASK OF WOMEN LESS THAN 55 YEARS ONLY: Are you pregnant or do you suspect you're pregnant or planning to try to get pregnant in the next 6 months?
   - ☐ Yes
   - ☐ No
   - ☐ Don't know/Refuse

6. In the next 12 months, are you planning to see any of the following health care providers for your back problem?
   - ☐ Medical specials (ex. Rheumatologist or neurologist)
   - ☐ Surgeon (ex. Orthopedic surgeon or neurosurgeon)
   - ☐ Physical therapist

- ☐ Chiropractor
- ☐ Osteopath
- ☐ Massage therapist or other body worker
- ☐ Acupuncturist
- ☐ Other, please specify:______________________
- ☐ None
- ☐ Don't know/Refuse

7. Do you have an appointment already scheduled?
   - ☐ Yes
   - ☐ No

8. If yes, are you willing to postpone it until the completion of the study?
   - ☐ Yes
   - ☐ No
   - ☐ Don't know/Refuse

9. Have you practiced yoga in the past 6 months?
   - ☐ Yes
   - ☐ No

10. Have you visited a physical therapist in the past 6 months?
    - ☐ Yes
    - ☐ No

© Boston Medical Center

Survey Distribution Schedule

Measures	Baseline	6 weeks	12 weeks	26 weeks	40 weeks	52 weeks
**BASELINE INFORMATION**						
Sociodemographics	x					
Expectations	x					
Comorbidities	x					
**PRIMARY OUTCOMES**						
Low Back Pain Score	x	x	x	x	x	x
Roland Morris Disability (RMDQ)	x	x	x	x	x	x
**SECONDARY OUTCOMES**						
Pain Medication Use	x	x	x	x	x	x
Work Productivity	x	x	x	x	x	x
Health-related Quality of Life (SF-36)	x	x	x	x	x	x
Global Improvement		x	x	x	x	x
Satisfaction with Treatment	x	x	x	x	x	x
**TREATMENT-RELATED INFORMATION**						
Cost Diary		x	x	x	x	x
Adverse Events		x	x	x	x	x
Other LBP Treatments	x					x
**EXPLORATORY OUTCOMES**						
Fear Avoidance Belief (FABQ)	x	x	x	x	x	x
Back Pain & Exercise History	x	x	x	x	x	x
Alcohol, Drug, Smoking	x		x			x
Height & Weight	x		x			x
Pain Self-Efficacy (PSEQ)	x		x			x
Pittsburgh Sleep Quality (PSQI)	x		x			x
Depression (PHQ-8)	x		x			x
Anxiety (GAD-7)	x		x			x
Coping Strategies (CSQ)	x		x			x
Perceived Stress (PSS)	x		x			x

Procedure for Filing Paper Surveys

1 Immediately file paper forms that need to be entered into StudyTrax™ in locked file cabinet labeled "To Be Entered"

↓

File paper forms by cohort section (i.e. 1,2,3 or 4)

↓

File form in appropriate folder within cohort section

↓

2 Initial entry of paper form into StudyTrax™

↓

3 File form in the "For Double Data Entry" section in locked file cabinet labled "Double Data Entry"

↓

4 Second entry of paper form into StudyTrax™

↓

5 File form in the "Double Data Entry Complete" section in locked file cabinet labled "Double Data Entry"

↓

6 File form in participant's folder in locked cabinet labled "Participant Files"

© Boston Medical Center

Procedure for Filing Home Practice Logs and Adverse Events

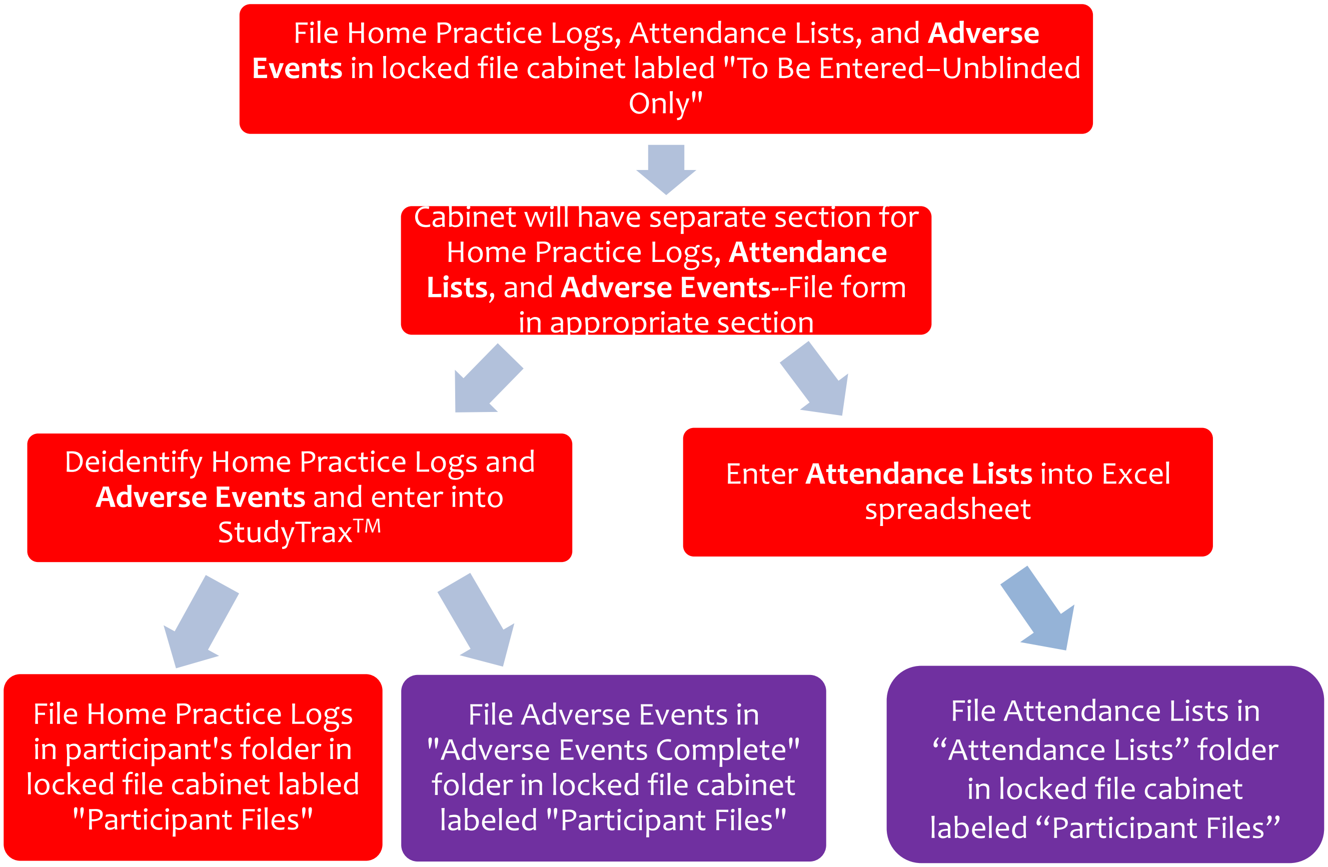

© Boston Medical Center

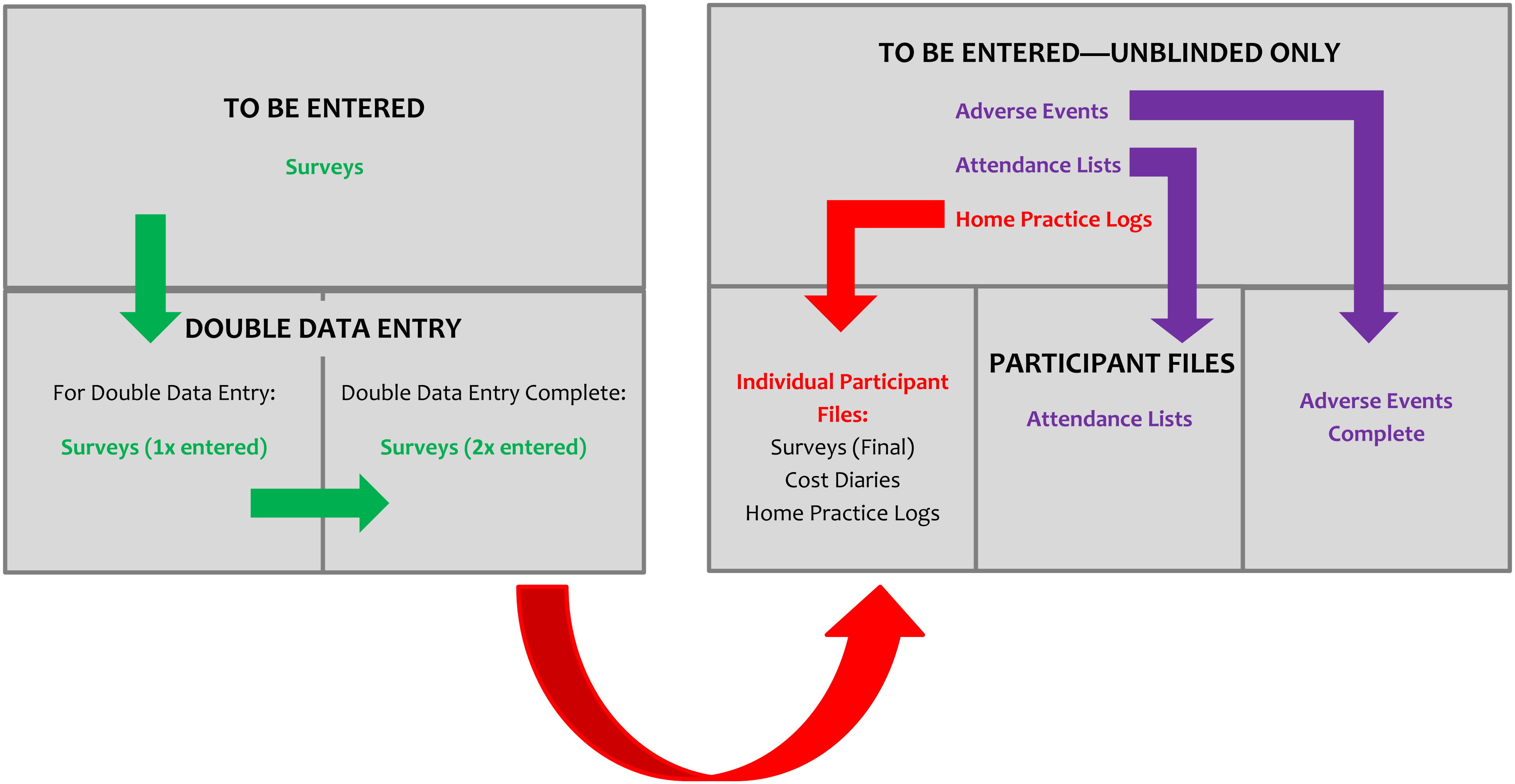
TO BE ENTERED
Surveys
DOUBLE DATA ENTRY
For Double Data Entry:
Surveys (1x entered)
Double Data Entry Complete:
Surveys (2x entered)
TO BE ENTERED—UNBLINDED ONLY
Adverse Events
Attendance Lists
Home Practice Logs
Individual Participant Files:
Surveys (Final)
Cost Diaries
Home Practice Logs
PARTICIPANT FILES
Attendance Lists
Adverse Events Complete

Qualitative Policies and Procedures

Qualitative Interview Request Script

<u>Overview:</u>

Once participants have completed the year-long participation, they will be randomly selected to participate in a one-on-one interview.

<u>Suggested Script:</u>

Hello, my name is **[name]** *calling from Back to Health. How are you today?*

This month, we are scheduling one-on-one interview sessions with randomly selected participants to learn more about your experience in our study. I'm calling you today to find out if you would like to participate. The sessions will be held for about an hour and will be located at Boston Medical Center. You will be compensated with a $25 gift card for your time.

Would you be interested in coming in to participate in the interview sessions?

If yes:

Great! The times that we have available are: [Available one-hour sessions]. Will you be able to make it to any of these?

If no:

Thank you for your time and have a great day!

© Boston Medical Center

Interview/Focus Group Guide for Participants

Introduction

Suggested Script: *I would like to thank you for coming today to share your experiences, thoughts and opinions with us. We are looking for your feedback in order to improve the way we study and treat low back pain. This is an open discussion and everything you share with me today is valuable. If there are any topics that we don't touch on, but that you feel are relevant, please feel free to let me know at the end of the interview. This discussion will be recorded; however, we will make sure to keep this recording confidential and for use by our study staff only.*

Discussion Topics

Motivations, Perceptions, and Expectations

These questions examine whether preconceived notions about yoga had an affect on peoples' interest in the study and how their perceptions of the study affected their willingness to participate.

1. **Why did you want to be in the study?**
   - Possible Follow-Up Questions:
     - *What did you see as the possible benefits of this study?*
     - *What about the study was important to you?*
     - *What did you expect to get out of this study? How did you think/hope it would impact your life?*
     - *How has low back pain has affected your life?*

2. **When you think about yoga/physical therapy/self-care, what do you think of?**
   - Possible Follow-Up Questions:
     - *Who is yoga for?*
     - *What is it about yoga that you think might be helpful to you?*
     - *What does "alternative medicine" mean to you?*
     - *How are your thoughts about yoga/PT/self-care different after participating in the study?*

3. **How did you find out about the study?**
   - Possible Follow Up Questions:
     - *What was it about the letter/brochure that sparked your interest?*
     - *How did your doctor talk to you about the study? What was it about the conversation that made you want to enroll in the study?*
     - *What part did your doctor play in your desire to be involved in the study?*

Interview/Focus Group Guide for Participants

4. **What did you think about a study of yoga/physical therapy/education for low back pain?**

5. **What were some of your thoughts and feelings before you spoke with the study staff?**

6. **What was your motivation for calling about the study?**

Yoga/Physical Therapy/Education Experience

These questions are meant to assess how satisfied or dissatisfied the subjects are with the yoga/physical therapy/education intervention.

1. **What was your experience like with yoga class/PT sessions/the book?**
   - Possible Follow-Up Questions:
     - *How did you feel about the instructor/physical therapists?*
     - *[Yoga only] How did you feel about being in a class with other participants as opposed to individual classes?*
     - *How did you feel about learning how to self-manage pain?*

2. **What did you learn from yoga class/physical therapy sessions/the book?**
   - Possible Follow-Up Questions:
     - *How do you feel about how much you learned?*
     - *If you attended yoga classes twice a week, how did the two classes compare?*
     - *How was the helpbook useful in managing your pain?*

3. **How did your back feel after yoga class/physical therapy sessions/trying activities in the book?**

4. **[Yoga only] How did yoga affect you spiritually?**

5. **How did yoga/physical therapy/self-care education affect your mood?**
   - Possible Follow-Up Questions:
     - *How was your confidence after doing yoga/physical therapy?*
     - *How did other people view your participation in the study (ex. friends or family)?*
     - *[Education only] How do you feel about your ability to self-manage pain after getting the book?*

6. **How do you think your perceptions of yoga/PT/self-care education previous to the study affected your experience?**

7. **What made home yoga/PT practice/education successful or unsuccessful for you?**
   - Possible Follow-Up Questions:
     - *What can the study do to make your home practice/education easier?*

8. **How do you plan to deal with your low back pain in the future?**
   - Possible Follow-Up Questions:
     - *What do you think your medication use will be like?*

Barriers to Attendance to Yoga Class/Physical Therapy Sessions

These questions look at the potential barriers to attendance to the yoga classes or physical therapy sessions.

1. **What about the yoga/PT experience made it easier or harder to attend class/appointments?**
   - Possible Follow-Up Questions:
     - *What made it easy or hard for you to attend your first person visit?*
     - *Did you receive a reminder call from our staff? Was it helpful?*
     - *How was the health center staff?*

2. **What factors in your life made it easier or harder to attend class/appointments?**

Barriers to Education Participation

These questions look at the potential barriers to learning from the self-care book and effectively self-managing low back pain.

1. **What made it easier or harder to learn from the book?**
   - Possible Follow-Up Questions:
     - *What made it easy or hard to try the activities the book talked about?*
     - *Did you receive a reminder call from our staff? Was it helpful?*

2. **What factors in your life made it easier or harder to read the help book?**

© Boston Medical Center

Adverse Events Procedures

Adverse Event Form

Randomization ID #:___________ **Staff Initials:** ____________

Date staff became aware of the AE: ______/_____/_________ **Serious:** ☐Yes ☐No

(If yes, notify PI immediately)

TO BE FILLED OUT BY UNBLINDED STAFF ONLY

Suggested Prompts:

When did this problem or injury begin (i.e. in the morning, after exercise; did it begin yesterday or years ago)? If you attend yoga or physical therapy sessions, did this problem occur before, during, or after your session? If you are in the education group, did this problem occur after doing a suggested activity?

Where and when do you feel the pain? Is the pain constant or does it come and go? Would you say the pain is sharp, burning, cramping, or pulling?

Does the pain spread between different parts of your body (i.e. from your thigh to your toes, across your back, down your neck)?

Please tell me how severe this problem or injury is, where **0** means *not at all severe* and **10** means *very severe*:

☺ **0** **1** **2** **3** **4** **5** **6** **7** **8** **9** **10** ☹

© Boston Medical Center

Adverse Event Form

Back to Health — A Study Comparing Yoga, Physical Therapy & Education for Chronic Low Back Pain

TO BE FILLED OUT BY STAFF CLINICIAN ONLY

PI/Clinician Signature: ________________ **Date participant contacted (if needed):** ___/_____/_____

Please check off the appropriate boxes:

A. Severity	B. Serious	C. Expected	D. Relatedness	E. Unanticipated Problem
☐ Mild<br>☐ Moderate<br>☐ Severe	☐ **Yes**<br>☐ No	☐ Yes<br>☐ **No**	☐ **Definitely related**<br>☐ **Possibly related**<br>☐ Not related	☐ Yes *(expedited report)*<br>☐ No *(progress report, weekly log)*

Clinician Notes

____

____

____

F. Step Taken	G. Follow-up Plan (if needed)
☐ Follow-up Needed *(See G.)* →<br>☐ None<br>☐ Discontinued intervention temporarily<br>☐ Discontinued intervention permanently<br>☐ Other____________________	______________________________<br>______________________________<br>______________________________<br>______________________________

H. Outcome

PI/Clinician Signature________________________ **Date of Resolution**_____/_____/______

☐ Resolved, no sequela
☐ AE still present, no treatment
☐ AE still present, being treated
☐ Residual effects present, not treated
☐ Residual effects present, treated
☐ Unknown

Outcome Notes: ______________________________

____

____

____

OFFICIAL DOCUMENTATION ON STUDYTRAX

Adverse Event Procedures

A. OVERVIEW

Responsibility

- Principal Investigator
- All unblinded study staff
- Doctoral Research Fellow
- Academic Research Fellow
- Questions: Data RA 1
- AE Contact Line: [phone number]

Materials

- Corresponding Adverse Event Call Script
- Adverse Event Form
- Adverse Event Summary Log

Inquiring about AEs

Potential adverse events (AEs) throughout the intervention will be collected in two different ways:

1. Participants are instructed in their study introduction packets to contact the study staff immediately if they experience an adverse event during their involvement in the study. All participants will have 24-hour emergency contact information for the PI and the research staff. Yoga teachers and physical therapists will check in with participants about possible AEs before the start of the session and will provide information for potential AEs (name and description). Research staff will check in with the education group every 3 weeks.
2. All data collections (e.g., 6, 12, 24, 40, 52 weeks) will include a question on whether the participant believes he/she experienced any potential AEs.

AE Definition

An AE is any untoward medical occurrence in a participant temporally associated with participation in the clinical study. An adverse finding can include a sign, symptom, or abnormal assessment (e.g., laboratory test value) or any combination of these.

AE Classification

1. **Seriousness**
   A Serious Adverse Event (SAE) is any adverse event that results in one or more of the following outcomes:
   - Death
   - A life-threatening event
   - Inpatient hospitalization or prolongation of existing hospitalization
   - A persistent or significant disability/incapacity
   - A congenital anomaly or birth defect
   - Important medical event based upon appropriate medical judgment

2. **Classification of AE Severity**
   AEs are classified based on participant history obtained and clinical judgment. AEs will be classified as mild, moderate, or severe. The severity classification is based on the AE's impact on the participant. An AE will be termed 'mild' if it has no or mild impact; 'moderate' if it causes the patient some functional limitation but does not impair activities of daily living;

© Boston Medical Center

and 'severe' if it causes a substantial disruption to the participant's function necessitating assistance with activities of daily living. (Please note that severity and seriousness are not the same. A participant can have a 'severe' event that is not a SAE and a 'moderate' event that meets the SAE definition.)

3. **AE Attribution Scale**
   AEs will be categorized according to the likelihood that they are related to the study intervention. Specifically, they will be labeled either definitely, possibly, or not related.

4. **Expected Risks**
   The hatha yoga and physical therapy interventions in this study minimal risk. However possible risks to the participant are as follows:
   a. Neck pain
   b. Worsening back pain
   c. Muscle spasm
   d. Joint pain
   e. Headache
   f. Leg pain
   g. Arm pain
   h. Numbness
   i. Tingling
   j. Weakness
   k. Dizziness
   l. Nausea
   m. Abdominal pain
   n. Chest pain
   o. Falling

NOTE: These risks are considered to be minimal and are addressed in the protocol and consent form. The risks will be minimized by multiple factors:

- The yoga and PT protocols were designed by experts specifically to minimize risk and maximize benefit for adults with chronic non-specific low back pain.
- The protocols have been piloted and refined extensively in research and clinical settings and have been found to be very safe. In our pilot study, there were no serious adverse events or moderate to severe adverse events.
- The yoga instructors and physical therapists administering the interventions have extensive training and experience working with this population.
- Yoga and PT sessions will be observed periodically by team leaders and national experts to assure the protocol is being taught appropriately.
- Participants will be continually reminded to not engage in any movement that they are not comfortable with or that causes significant pain.
- Participants will be continually encouraged and reminded to report any unusual or unexpected symptoms they experience that may be from the yoga or PT intervention. In addition to reporting adverse effects at treatment sessions, all participants will be asked

about AEs during data collection. They will also have 24-hour emergency contact information for the PI and the research staff to report any problem or event possibly related to the study.

- Our exclusion criteria will prevent individuals at greatest risk for injury from participating (e.g., low back pain due to infection, malignancy, or fracture; severe neurological deficits; serious co-morbidities precluding the safe performance of the protocol).
- To prevent potential psychological discomfort from disclosing personally sensitive information, the participants will be reminded multiple times that there is no penalty for declining to answer any question or to withdraw from the study. If a participant at any point reveals suicidal ideation, either through their answers to the surveys or otherwise, she or he will be immediately be referred to their PCP, the BMC Department of Psychiatry, and/or the BMC Emergency Room.

There are no known risks associated with the Education intervention.

B. DOCUMENTATION PROCEDURES

Follow-up

1. Once an adverse event has been identified, Part 1 of the Adverse Event Form will be completed immediately by an unblinded staff member on **StudyTRAX**. The staff member who first became aware of the AE will notify a clinician of the AE via a 'Task' on StudyTRAX and by email/text to assure completion of initial follow-up.

2. If the clinician judges the AE to be serious, he/she will notify the PI immediately, who will follow-up with the participant within 24 hours. Otherwise, non-serious AEs will be followed-up by the clinician within 1 week. At this time, Part 2 of the Adverse Event form will be completed by the clinicians on **StudyTRAX**.

3. The clinicians will instruct the unblinded staff if further follow-up is needed.

Reporting Adverse Events

1. AEs that are **unexpected, serious, and possibly or definitely related** to the study intervention are **unanticipated problems** (UPs) and will be promptly reported to the DSMB, IRB, and NCCAM within two business days of the staff becoming aware of the occurrence.

2. Non-serious AEs, SAEs that are not UPs, and AEs unrelated to the study intervention will be reported semi-annually via IRB Progress Reports in summary format.

Weekly Review of AEs

All AEs, both serious and non-serious, and UPs will be logged into an Adverse Event Log. This log contains information about classification and will be reviewed and signed by the PI weekly at team meetings.

Filing

All AEs will be kept on StudyTRAX within participant's individualized participant pages. These events will be exported from StudyTrax and included in the DSMB quarterly reports.

Adverse Event Procedures

1. **Classifying Adverse Events**

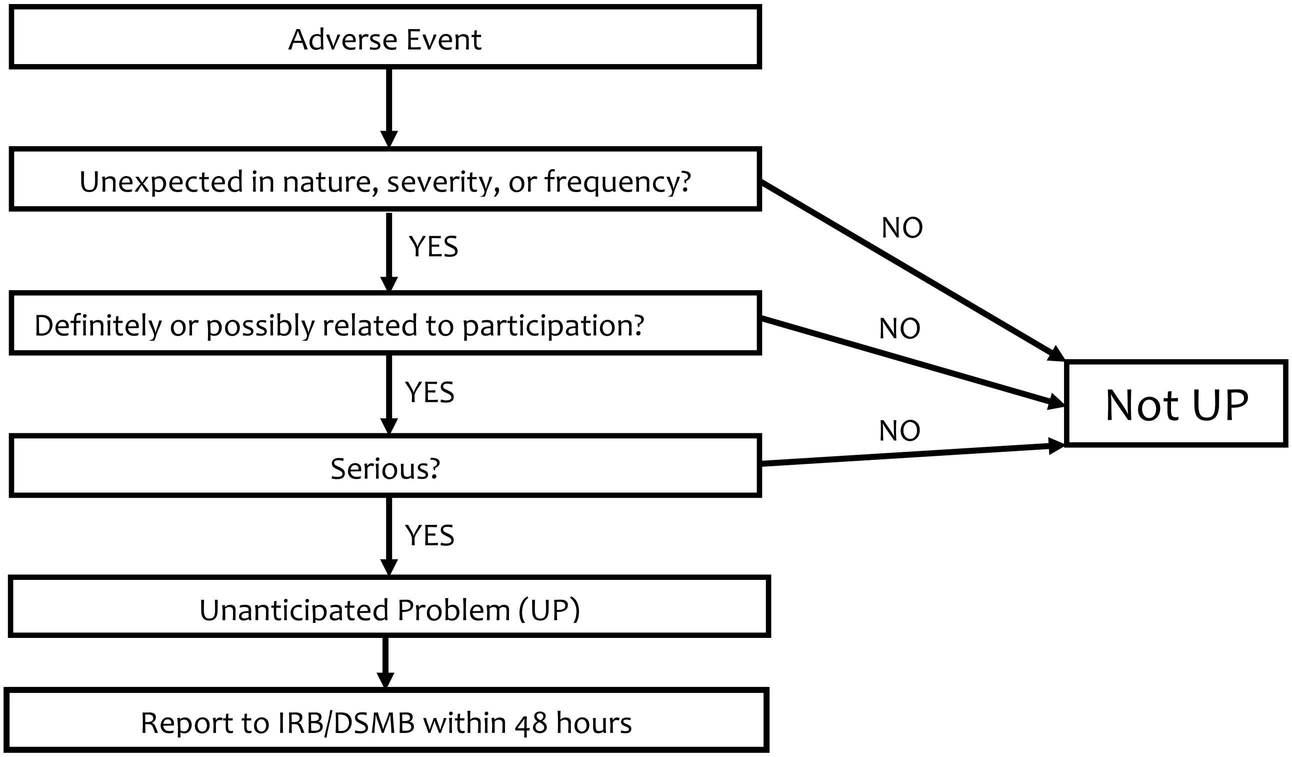

2. **Adverse Events vs. Unanticipated Problems Diagram**

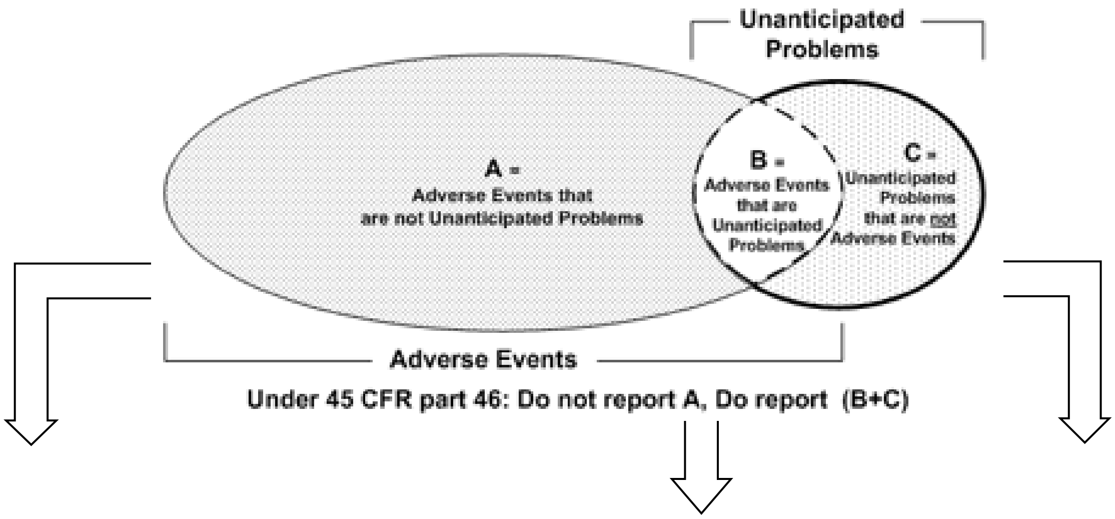

EXAMPLES

A. muscle spasms, dizziness, nausea *(which are outlined in the IRB-approved protocol and consent form)*

B. hospitalization for pinched nerve *(serious, unexpected, related)*

C. breach of confidentiality *(involves social or economic harm instead of the physical or psychological harm associated with adverse events)*

© Boston Medical Center

3. **<u>AE Collection/Reporting Flow Diagram</u>**

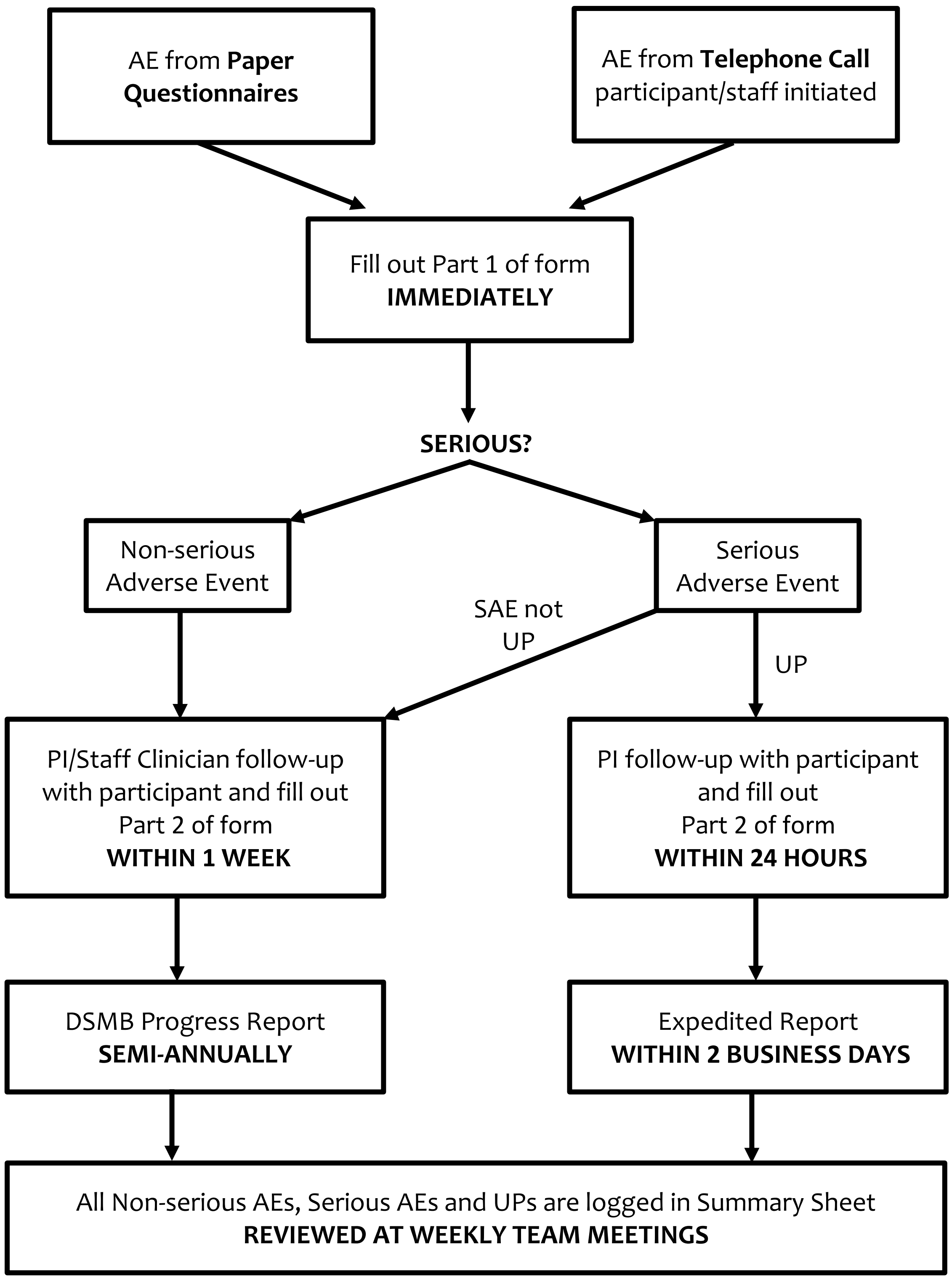

© Boston Medical Center
