# Supplementary material for: Yoga vs. physical therapy vs. education for chronic low back pain in predominantly minority populations: study protocol for a randomized controlled trial
Source: Trials. 2014 Feb 26;15:67. doi: 10.1186/1745-6215-15-67 (PMC3944007; doi:10.1186/1745-6215-15-67)
Supplement: Additional file 6 — Qualitative Interview Guides. Semi-structured interview guides used to facilitate individual qualitative interviews with participants from each treatment arm. [file 1745-6215-15-67-S6.pdf]

Interview Guide for <u>YOGA</u> Participants

Introduction

Suggested Script: *I would like to thank you for coming today to share your experiences, thoughts and opinions with us. We are looking for your feedback in order to improve the way we study and treat low back pain. This is an open discussion and everything you share with me today is valuable. If there are any topics that we don't touch on, but that you feel are relevant, please feel free to let me know at the end of the interview. This discussion will be recorded; however we will make sure to keep this recording confidential and for use by our study staff only.*

Discussion Topics

Motivations, Perceptions, and Expectations

These questions examine whether preconceived notions about yoga had an effect on peoples' interest in the study and how their perceptions of the study affected their willingness to participate.

1. **Why did you want to be in the study?**
   a. What did you expect to get out of this study?
   b. How did you expect it would impact your life? Others' lives?
   c. How do you think your expectations have changed throughout the course of the study?

2. **During your first meeting with us, we talked about how the Yoga classes are designed specifically for people with low back pain. How did you feel knowing that Yoga classes could be designed for specific conditions like yours?**
   a. When you heard this, did it change your expectations of whether this treatment would work? Why or why not?

3. **What, if anything, did you know about yoga before beginning this study?**
   a. Did you associate "yoga" with a group of people? A culture? A religion?
   b. Did you look up yoga on your own? Internet? Library?
   c. Did your friends/family have anything to say about yoga?

4. **Which treatment did you expect would be most beneficial for LBP? Why?**

5. **Why do you think yoga worked? (or didn't work?)**

Experience
These questions are meant to assess how satisfied or dissatisfied the subjects are with the yoga intervention.

1. **What was your initial experience like with yoga?**
    a. How did you feel after your first class?

2. **Describe the effects yoga had on you that are NOT related to your LBP.**
    a. Mood? (e.g., being less snappy or not as quick to react in difficult situations)
    b. Relationships?
    c. Other physical, e.g., weight loss, muscle tone, etc.?
    d. Anxiety/depression?
    e. Your overall diet?
    f. Connectedness? (with yourself or others)
    g. Mind-body relationship?
    h. Inner peace? Introspection? (Looking inside yourself?)

3. **Some people have described the sorts of effects and experiences associated with yoga as "spiritual or religious experiences." What do you think about that?**
    a. What do you mean by "spiritual?"

4. **How has Yoga influenced how you feel about LBP? How you cope with it?**
    a. How have you managed your pain?

5. **How do you plan to incorporate what you have learned in this study into your life in the future?** (LBP related and/or not)

6. **Thinking about what you got out of your yoga experience, which is more important to you—the mental effects (ex. better mood) or the pain relief? Why?**

7. **How has your perception of yoga changed over the course of this study?**
    a. How do you think your perceptions of Yoga previous to the study affected your experience?
    b. *[If participant associated yoga with religion/spirituality before study]* How did practicing Yoga confirm or deny your beliefs about Yoga in terms of spirituality/religion?

8. **What was your overall experience like with yoga?**
    a. Describe your experience/interactions with yoga teachers.
    b. What was it like being in a group with other people with LBP?

c. How would you compare a group treatment like yoga to a one-on-one treatment? Do you have a preference? How did the study influence that preference, if at all?

Barriers to Attendance

These questions are aimed at assessing any challenges participants faced in attending their yoga classes.

1. **Describe your experiences attending yoga classes.**
   a. What made going to your class easier?
   b. What made going to your class more difficult?
   Probes: Time of day- were mornings particularly difficult?
   Meeting locations
   Transportation
   Finding childcare
   Personal schedule
   Work schedule
   Group experience/yoga teacher(s)

Conclusion: Is there anything else you want to talk about that we haven't already discussed or that you think it would be helpful for us to know about?

Interview Guide for PHYSICAL THERAPY Participants

Introduction

Suggested Script: *I would like to thank you for coming today to share your experiences, thoughts and opinions with us. We are looking for your feedback in order to improve the way we study and treat low back pain. This is an open discussion and everything you share with me today is valuable. If there are any topics that we don't touch on, but that you feel are relevant, please feel free to let me know at the end of the interview. This discussion will be recorded; however we will make sure to keep this recording confidential and for use by our study staff only.*

Discussion Topics

**Motivations, Perceptions, and Expectations**<br>These questions examine whether preconceived notions about PT had an effect on peoples' interest in the study and how their perceptions of the study affected their willingness to participate.

1. **Why did you want to be in the study?**
    a. What did you expect to get out of this study?
    b. How did you expect it would impact your life? Others' lives?
    c. How do you think your expectations have changed throughout the course of the study?

2. **During your first meeting with us, we talked about how the physical therapy sessions are designed specifically for people with low back pain. How did you feel knowing that physical therapy could be designed for specific conditions like yours?**
    a. When you heard this, did it change your expectations of whether this treatment would work? Why or why not?

3. **What, if anything, did you know about physical therapy before beginning this study?**
    a. Have you done physical therapy before?
    b. Did you it look up on your own? Internet? Library?
    c. Did your friends/family have anything to say about physical therapy?

4. **Which treatment did you expect would be most beneficial for LBP? Why?**

5. **Why do you think physical therapy worked? (or didn't work?)**

Experience

These questions are meant to assess how satisfied or dissatisfied the subjects are with the physical therapy intervention.

1. **What was your initial experience like with physical therapy?**
    a. How did you feel after your first session?

2. **What was your overall experience like with physical therapy?**
    a. Describe your experience/interactions with physical therapist.
    b. What was it like being in a study with other people with LBP?
    c. How would you compare a one-on-one treatment like physical therapy to a group treatment (like yoga)? Do you have a preference? How did the study influence that preference, if at all?

3. **Describe the effects physical therapy had on you that are NOT related to your LBP.**
    a. Mood? (e.g., being less snappy or not as quick to react in difficult situations)
    b. Relationships?
    c. Other physical, e.g., weight loss, muscle tone, etc.?
    d. Anxiety/depression?
    e. Your overall diet?
    f. Connectedness? (with yourself or others)
    g. Mind-body relationship?
    h. Inner peace? Introspection? (Looking inside yourself?)

4. **How has physical therapy influenced how you feel about LBP? How you cope with it?**
    a. How have you managed your pain?

5. **How do you plan to incorporate what you have learned in this study into your life in the future?** (LBP related and/or not)

6. **Thinking about what you got out of your physical experience, which is more important to you—the mental effects (ex. better mood) or the pain relief? Why?**

7. **How has your perception of physical therapy changed over the course of this study?**
    a. How do you think your perceptions of physical therapy previous to the study affected your experience?

Barriers to Attendance

These questions are aimed at assessing any challenges participants faced in attending their physical therapy sessions.

1. **Describe your experiences attending physical therapy.**
    a. What made going to your session easier?
    b. What made going to your session more difficult?
    Probes: Time of day- were mornings particularly difficult?
    Meeting locations
    Transportation
    Finding childcare
    Personal schedule
    Work schedule
    Group experience/Yoga teacher(s)

Conclusion: Is there anything else you want to talk about that we haven't already discussed or that you think it would be helpful for us to know about?

Interview Guide for <u>EDUCATION</u> Participants

<u>Introduction</u>

<u>Suggested Script</u>: *I would like to thank you for coming today to share your experiences, thoughts and opinions with us. We are looking for your feedback in order to improve the way we study and treat low back pain. This is an open discussion and everything you share with me today is valuable. If there are any topics that we don't touch on, but that you feel are relevant, please feel free to let me know at the end of the interview. This discussion will be recorded; however we will make sure to keep this recording confidential and for use by our study staff only.*

<u>Discussion Topics</u>

**Motivations, Perceptions, and Expectations**<br>These questions examine whether preconceived notions about education had an effect on peoples' interest in the study and how their perceptions of the study affected their willingness to participate.

1. **Why did you want to be in the study?**
   a. What did you expect to get out of this study?
   b. How did you expect it would impact your life? Others' lives?
   c. How do you think your expectations have changed throughout the course of the study?

2. **During your first meeting with us, we talked about how the education book was designed specifically for people with low back pain. How did you feel knowing that there was a whole people just about back pain?**
   a. When you heard this, did it change your expectations of whether this treatment would work? Why or why not?

3. **What, if anything, did you know about chronic low back pain before beginning this study?**
   a. Did you look up chronic low back pain on your own? Internet? Library?
   b. Did you know anyone else with back pain like you?

4. **Which treatment did you expect would be most beneficial for LBP? Why?**

5. **Why do you think this treatment worked? (or didn't work?)**

Experience

These questions are meant to assess how satisfied or dissatisfied the subjects are with the education intervention.

1. **What was your initial experience like with the education treatment?**

2. **What was your overall experience like with education?**
   a. Describe your experience/interactions with staff.
   b. What was it like doing a treatment by yourself rather than with other people with LBP?
   c. How would you compare a one-on-one treatment to a group treatment? Do you have a preference? How did the study influence that preference, if at all?

3. **Describe the effects your treatment had on you that are NOT related to your LBP.**
   a. Mood? (e.g., being less snappy or not as quick to react in difficult situations)
   b. Relationships?
   c. Other physical, e.g., weight loss, muscle tone, etc.?
   d. Anxiety/depression?
   e. Your overall diet?
   f. Connectedness? (with yourself or others)
   g. Mind-body relationship?
   h. Inner peace? Introspection? (Looking inside yourself?)

4. **How has the education treatment influenced how you feel about LBP? How you cope with it?**
   a. How have you managed your pain?

5. **How do you plan to incorporate what you have learned in this study into your life in the future?** (LBP related and/or not)

6. **Thinking about what you got out of your treatment experience, which is more important to you—the mental effects (ex. better mood) or the pain relief? Why?**

7. **How has your perception of health education changed over the course of this study?**
   a. How do you think your perceptions of health education previous to the study affected your experience?

Barriers to Adherence

These questions are aimed at assessing any challenges participants faced in adhering to their treatment.

1. **Describe your experiences reading the treatment materials.**
    a. What made reading the materials easier?
    b. What made reading that materials more difficult?

Conclusion: Is there anything else you want to talk about that we haven't already discussed or that you think it would be helpful for us to know about?
